# Visible light-induced 1,2-alkoxy shift of α-diazoacetates for Wolff rearrangements – access to oxyketenes

**DOI:** 10.1039/d5sc08263c

**Published:** 2025-12-19

**Authors:** Yang Liu, Zi-Yi Xie, Lennard Kloene, Cong-Lun Xu, Jian-Peng Tai, Yu Zhu, Bao-Gui Cai, Chongqing Pan, Rene M. Koenigs, Jun Xuan

**Affiliations:** a Anhui Province Key Laboratory of Chemistry for Inorganic/Organic Hybrid Functionalized Materials, College of Chemistry & Chemical Engineering, Anhui University Hefei Anhui 230601 China xuanjun@ahu.edu.cn cqpan@ahu.edu.cn; b University of Bayreuth, Organic Chemistry II Universitätsstr. 30 95447 Bayreuth Germany rene.koenigs@uni-bayreuth.de; c Key Laboratory of Structure and Functional Regulation of Hybrid Materials (Anhui University), Ministry of Education Hefei Anhui 230601 China

## Abstract

The Wolff rearrangement is an important transformation to access ketenes *via* 1,2-alkyl or aryl migrations of α-diazoketones. In contrast, alkoxy group migration from free singlet carbenes has remained elusive owing to the intrinsically low migratory aptitude of alkoxy groups. Here we report a visible-light-induced 1,2-alkoxy/aryloxy shift of α-diazoacetates that generates oxy-substituted ketenes. These intermediates undergo efficient [2 + 2] cycloadditions with imines and nucleophilic addition reactions with amines. The method tolerates a wide substrate range, operates on gram scale, and is supported by mechanistic and computational studies. This work expands the scope of Wolff rearrangements and opens new chemical space for oxyketene reactivity.

## Abstract 2

A visible-light-induced 1,2-alkoxy shift to generate oxy-substituted ketenes *via* Wolff rearrangement from α-diazoacetates is developed. This approach enables efficient [2 + 2] cycloadditions with imines and nucleophilic additions with amines.

## Graphical abstract

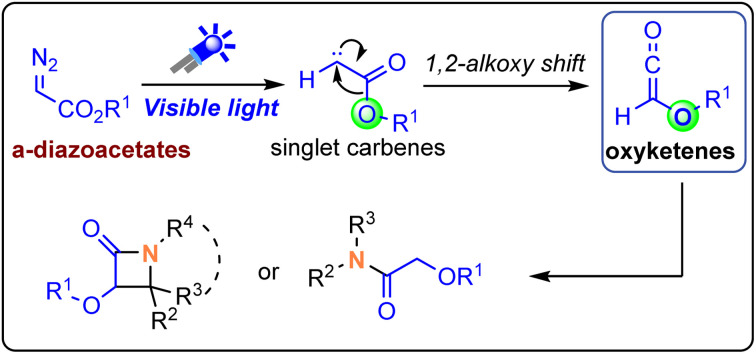

## Introduction

**Scheme 1:**
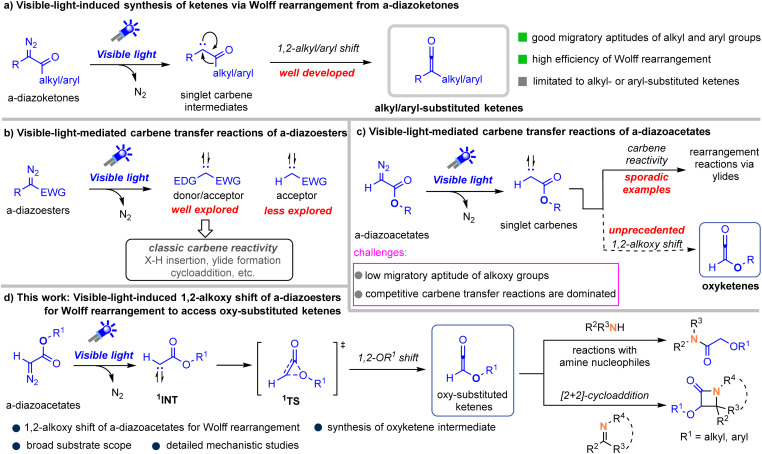
Visible-light-induced generation of ketenes *via* Wolff rearrangement.

The Wolff rearrangement is one of the most accessible and straightforward methods for generating ketene intermediates and was first discovered in 1902.^1^ It is commonly promoted by thermolysis, photolysis, or through the action of metal catalysts with diazo compounds, with the latter two strategies being more prevalently utilized.^2^ Notably, recent advances have demonstrated that Wolff rearrangements can be efficiently promoted even under visible-light irradiation, offering a mild and clean alternative to traditional activation modes.^3^ This process starts with the visible-light-mediated extrusion of dinitrogen to generate a free singlet carbene intermediate, followed by a 1,2-alkyl or aryl migration to access ketenes (Scheme 1a).

In the past few years, such visible-light-promoted reactions of diazoalkanes have witnessed significant developments with a main focus on carbene transfer reactions (Scheme 1b).^4,5^ Since 2018, such visible-light-driven, metal-free carbene transfer reactions have focused primarily on the use of donor/acceptor diazo compounds and are today an important strategy for green and sustainable carbene transfer reactions due to the strong absorption of donor/acceptor diazo compounds in the visible light region.^6^ In sharp contrast, carbene-transfer reactions employing acceptor-only diazoalkanes remain challenging. Their weak visible-light absorption and inherent structural constraints have resulted in only sporadic successful examples, in which ether or nitrile solvents are usually required to trap the free carbene to form ylide intermediates that themselves undergo rearrangement.^7^

We therefore hypothesized that a detailed exploration of visible-light-driven transformations of acceptor-only diazoalkanes could reveal distinct reactivity patterns diverging from those typical of donor/acceptor carbene intermediates. In this regard, we envisioned this free acceptor-only carbene species might undergo a 1,2-alkoxy shift to access previously underexplored oxy-ketene intermediates (Scheme 1c).^8^ Although this strategy seems feasible, the success to our designed pathway faces several challenges, such as (1) low migratory aptitude of alkoxy groups relative to common alkyl- and aryl groups,^2^ (2) the dominance of competitive carbene transfer reactions. Herein, we disclose the hypothesis of 1,2-alkoxy migration for Wolff rearrangement from α-diazoacetates, and the presence of oxy-substituted ketene intermediates can be verified by [2 + 2] cycloadditions with imines and nucleophilic addition reactions with amines (Scheme 1d).

## Results and discussion

### Reaction development

**Fig. 1:**
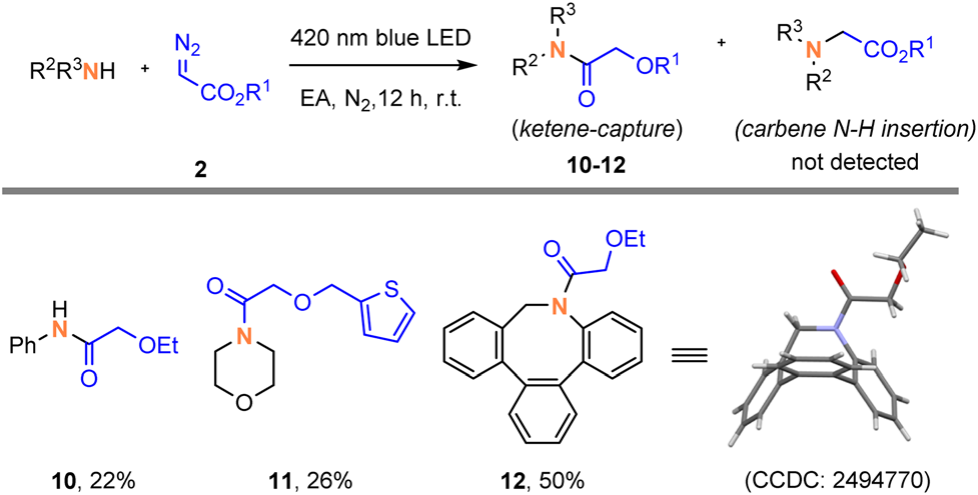
Initial reactions of α-diazoacetate with amine nucleophiles.

To probe the feasibility of the 1,2-alkoxy shifts in photochemical Wolff rearrangements, we initially studied the reactions of α-diazoacetates with several amine nucleophiles. To our delight, these oxyketene intermediates could be trapped by amines under 420 nm blue LED irradiation in ethyl acetate (EA) as a solvent, giving the corresponding amide derivative 10–12, respectively. We unambiguously confirmed the structure of ketene-captured adduct 12 by X-ray diffraction. To be noted, no competitive N–H insertion products were detected in these reactions (Fig. 1). Besides, the ketene-captured adduct 15 can also be obtained using benzyl mercaptan as the nucleophile (Scheme S1, page S17). These preliminary results indicate that the acceptor-only diazoalkanes exhibits distinct reactivity from donor–acceptor diazoalkanes.^6*a*,9^

Encouraged by this result, we then attempted to explore the [2 + 2] cycloaddition reactions of α-diazoacetates with imines. First, we selected *N*-methyl quinoxalin-2(1*H*)-one (1a) and ethyl diazoacetate (2a) as model substrates to optimize the reaction conditions (Table 1). To our delight, target [2 + 2] cycloaddition product *trans*-3a was obtained in 25% yield with excellent diastereoselectivity (>19 : 1 dr) under 455 nm blue LED irradiation in DCM (entry 1). Further reaction optimization revealed ethyl acetate as the best solvent (entries 2–3). The reaction yield is highly influenced by the wavelength of the light source (entries 3−5), and shorter wavelength irradiation (420 nm) increased the yield of *trans*-3a to 79% (entry 4). Replacing the ethyl substituent of the α-diazoacetate with 1-adamantyl further raised the yield to 95% under 420 nm blue LED irradiation (entry 7). Importantly, in the absence of light no reaction occurred, and the starting materials were fully recovered (entry 9).

**Table 1:** Reaction optimization^a^

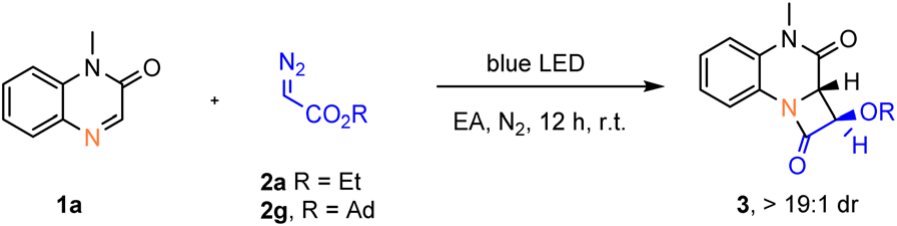				
Entry	Diazoacetate	Wavelength	Solvent	Yield^b^ %
1	2a	455 nm	DCM	25
2	2a	455 nm	Toluene	33
3	2a	455 nm	EA	51
4	2a	420 nm	EA	79
5	2a	390 nm	EA	66
6	2g	455 nm	EA	86
7	2g	420 nm	EA	95
8	2g	390 nm	EA	94
9^c^	2g	—	EA	N.R.

a Reaction conditions: 1a (0.2 mmol), 2 (0.6 mmol, 3.0 equiv.), in solvent (1.0 mL) at rt under blue LED irradiation for 12 h under nitrogen atmosphere. The diastereoisomeric ratio of product 3a or 3g is determined to be >19 : 1 by ^1^H NMR.

b Isolated yield.

c In the dark. EA = ethyl acetate. N.R. = no reaction.

### Evaluation of substrate scope

**Scheme 2:**
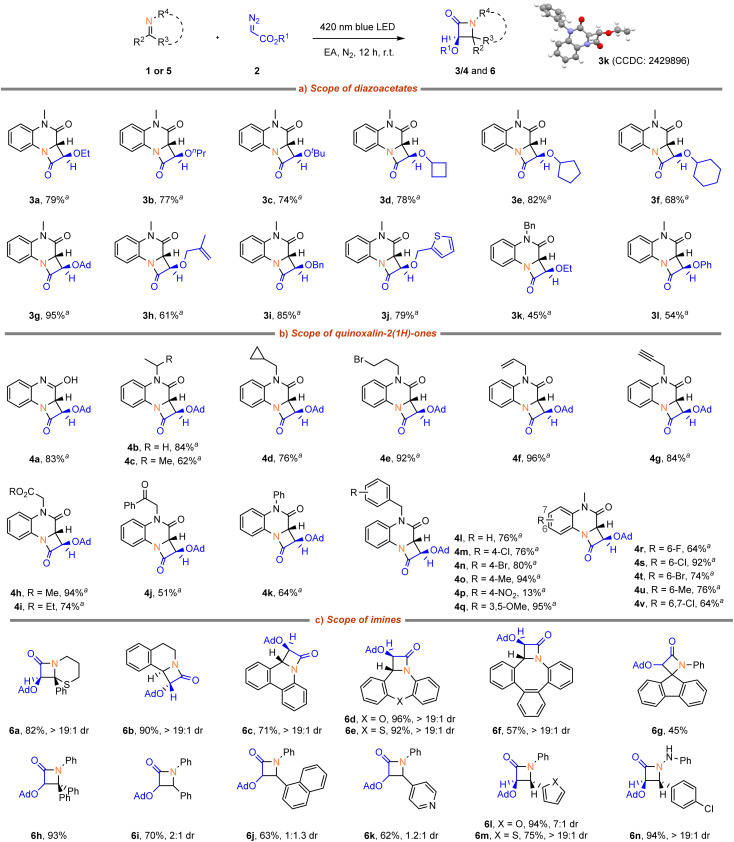
Scope of α-diazoacetate and imine substrates. ^*a*^dr >19 : 1.

With the optimized reaction conditions in hand, the scope of α-diazoacetates 2 was first investigated (Scheme 2a). It was found that various α-diazoacetates proved to be highly effective substrates for this reaction, delivering good yields with excellent diastereoselectivity. Specifically, alkyl diazoacetates smoothly underwent the transformation and provided the desired cycloaddition products (3a–f) in high yields. Notably, olefinic-, benzyl-, heteroaryl-, and phenyloxy-substituted diazoacetates were also tolerated in this reaction, affording the corresponding products (3h–j, 3l) in good yields. Importantly, no byproducts from intramolecular carbene transfer reactions, *e.g.* cyclopropanation for 3h or ylide formation for 3j, was observed. The structure of 3k was confirmed by X-ray diffraction.

Next, the scope of quinoxalin-2(1*H*)-ones 1 was examined (Scheme 2b). Quinoxalin-2(1*H*)-ones bearing H, alkyl, allyl, propargyl and phenyl functional groups at *N*-position were well tolerated (4a–k, 62–96% yields). *N*-benzyl derivatives also reacted smoothly and afforded products (4l–q) in 13–95% yields. Among them, benzyl groups with electron-donating substituents or weak electron-withdrawing groups, such as fluorine, chlorine, and bromine, exhibited good tolerance. However, the presence of strong electron-withdrawing nitro group (product 4p) had a significant negative impact on the reaction. Moreover, the benzene ring of quinoxalin-2(1*H*)-ones demonstrated tolerance towards different substituents, where the corresponding products (4r–v) were obtained in 64–92% yields.

Subsequently, we turned our attention to other heterocycles as imine coupling partners (Scheme 2c). 2-Phenyl-5,6-dihydro-4*H*-1,3-thiazine underwent cycloaddition efficiently, giving access to the product (6a) in 82% yield with excellent diastereoselecitivty. 3,4-Dihydroisoquinoline, phenanthridine, dibenzoxazepine, dibenzothiazepine, and saddle-shaped eight-membered azaheterocycle all underwent cycloaddition efficiently, giving access to the products (6b–f) in 57–92% yields with excellent diastereoselectivity. Moreover, *N*-phenyl ketimines also proved to be a viable substrate, giving the products (6g and 6h) with moderate to excellent yields. Acyclic aldimines led to poor diastereoselectivity (6i–k), while excellent diastereomeric ratios were observed in the cases of 6l–n.

### Synthetic applications

**Scheme 3:**
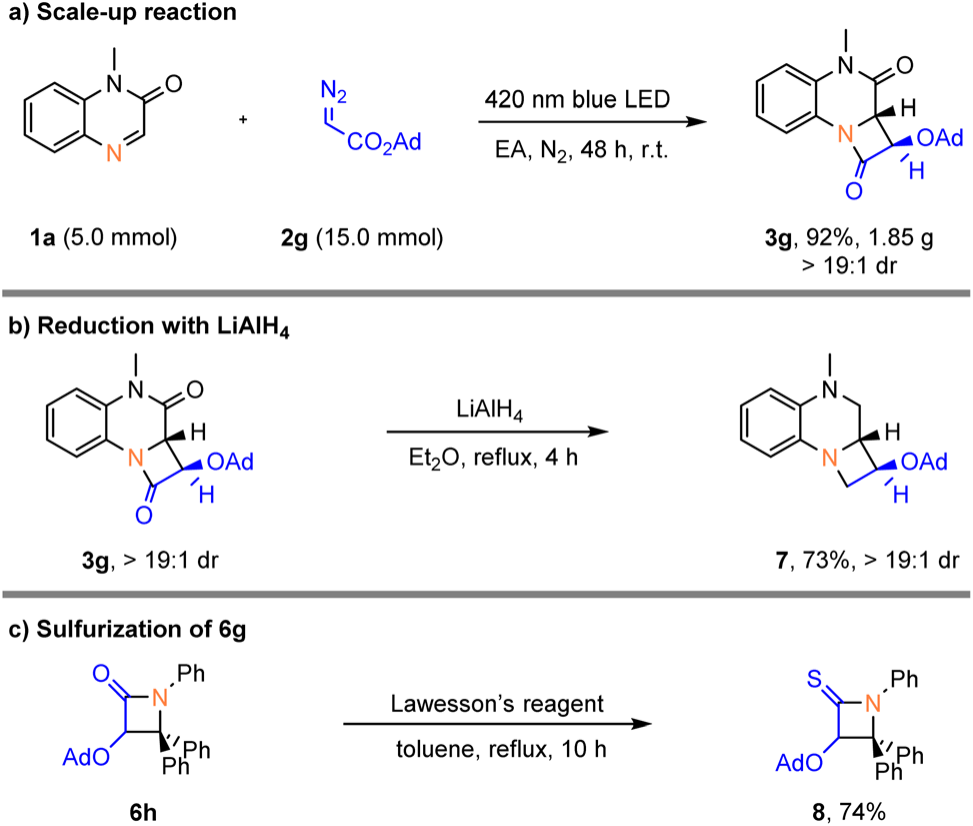
Gram-scale reaction and derivatization reactions.

In order to prove the synthetic practicality of this protocol, the reaction was scaled up to 5.0 mmol, giving 3g (1.85 g) in 92% yield with >19 : 1 dr after 48 h (Scheme 3a). The β-lactam 3g was then reduced efficiently by lithium aluminium hydride to give the azetidine 7 in good yield (Scheme 3b). Notably, the β-lactam 6g can smoothly react with Lawesson's reagent to afford 8 in 74% yield (Scheme 3c).

### Mechanistic studies

**Scheme 4:**
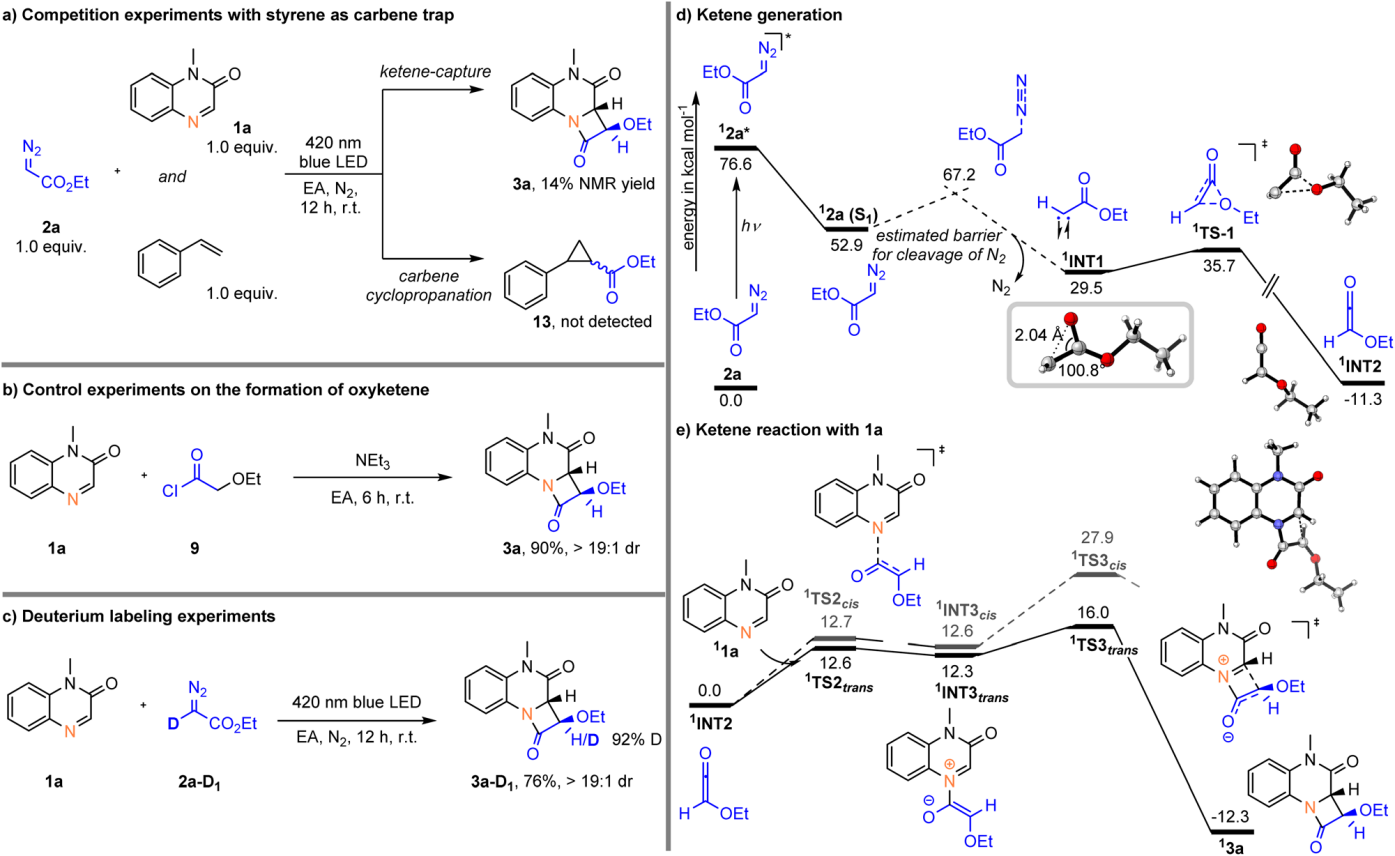
Combined experimental and computational mechanistic studies.

To gain some information about the mechanism of this photochemical reaction, a series of mechanistic studies were performed in Scheme 4. First, competition experiments using styrene as a carbene trap showed a 14% NMR yield for 3a, and no cyclopropanation product (13) was detected (Scheme 4a). In addition, we conducted a similar [2 + 2] cycloaddition reaction using acyl chloride (9) as the ketene precursor in the presence of Et_3_N, affording the target product 3a in 90% yield (Scheme 4b). Next, we conducted a D-labeling experiment to elucidate the α-hydrogen transfer of the diazo substrate during entire process. When the reaction was carried out with deuterated α-diazo ester 2a-D_1_, more than 90% deuteration incorporation was observed at the C3 position of the β-lactam ring of 3a, indicating that the hydrogen source came from ethyl diazoacetate substrate (Scheme 4c). Summarizing all the above results, it is believed that the ethoxyketene (2-ethoxyethen-1-one) intermediate is generated from ethyl diazoacetate (2a) under visible light irradiation.

In order to enhance our understanding of the mechanism, we complemented our experimental studies with DFT calculations. In line with previous findings on the photochemical Wolff rearrangement reactions,^10–12^ we analyzed photoexcitation of α-diazoacetate 2a. Photoexcitation of 2a results in a stepwise reaction mechanism – initial formation of the first excited state of 2a enables the loss of molecular nitrogen with an estimated energy barrier of 14.3 kcal mol^−1^. The structure of the formed singlet carbene intermediate already suggest the participation of oxirene-like bonding character. The interaction of the oxygen lone pair with the unoccupied orbital of the carbene result in a C–C–O bond angle of 100.8°. Next, the formed singlet carbene intermediate ^1^INT1 undergoes Wolff rearrangement to give the ethoxyketene intermediate ^1^INT2, which is in line with the performed control experiments (Scheme 4d).

In a next step, ketene ^1^INT2 reacts in a stepwise manner with 1a. Initial formation of the C–N bond proceeds *via* low-lying ^1^TS2*_trans_* with an activation energy of 12.6 kcal mol^−1^ to furnish zwitterionic intermediate ^1^INT3*_trans_*. Notably, the formation of the zwitterionic intermediate is highly reversible as ^1^INT3*_trans_* is only slightly stabilized compared to ^1^TS2*_cis_*. Subsequently, the final C–C bond formation occurs to give the observed 3a with excellent diasteroselectivity. The origin of the diastereoselectivity can be reasoned by the initial nucleophilic attack of ketene ^1^INT2. While the energy difference for the two possible transition states (^1^TS2*_trans_* and ^1^TS2*_cis_*) is nearly the same, the formation of the C–C bond *via*^1^TS3*_trans_* or ^1^TS3*_cis_* significantly differs and the transition state leading to the *trans*-β-lactam is favored by 11.9 kcal mol^−1^ (Scheme 4e).

Calculations exploring a carbene transfer reaction to 1a result in energetically more demanding reaction pathways compared to the observed intramolecular Wolff rearrangement. Therefore, the participation of a carbene transfer reaction pathways can be excluded (for details please see SI Fig. S1–S5).

## Conclusions

In summary, we have developed a visible-light-induced Wolff rearrangement reaction from α-diazoacetates for the generation of oxy-substituted ketenes *via* a 1,2-alkoxy/aryloxy shift. This approach enables highly efficient [2 + 2] cycloadditions with imines, affording structurally diverse β-lactams. Diverse fused nitrogen heterocycles, aldimines, ketimines, and hydrazones were found to be compatible with good yields and diastereoselectivity. The oxyketene intermediates also could react with several amine nucleophiles. We believe that this strategy can provide new chemical space for the visible-light-mediated 1,2-alkoxy/aryloxy shift of Wolff rearrangement from acceptor-only diazoalkanes.

## Author contributions

C. P., R. M. K. and J. X. conceptualised the work. Y. L. and Z.-Y. X. developed the methodology and performed the majority of the experiments. C.-L. X., J.-P. T., Y. Z. prepared some substrates. L. K. and R. M. K. performed and analysed the theoretical calculations. Y. L., Z.-Y. X., B.-G. C., C. P., R. M. K. and J. X. prepared the manuscript. All authors have given approval to the final version of the manuscript.

## Conflicts of interest

There are no conflicts to declare.

## Supplementary Material

SC-017-D5SC08263C-s001

SC-017-D5SC08263C-s002

## Data Availability

CCDC 2429896 and 2494770 contain the supplementary crystallographic data for this paper.^13*a*,*b*^ The data supporting this article have been included as part of the supplementary information (SI). Supplementary information: experimental details and characterization data for all products. See DOI: https://doi.org/10.1039/d5sc08263c.
