# Supplementary material for: Visible light-induced 1,2-alkoxy shift of α-diazoacetates for Wolff rearrangements – access to oxyketenes
Source: Chem Sci. 2025 Dec 19;17(7):3587–92. doi: 10.1039/d5sc08263c (PMC12757782; doi:10.1039/d5sc08263c)

Supplementary Information (SI) for Chemical Science.
This journal is © The Royal Society of Chemistry 2025

Visible Light-Induced 1,2-Alkoxy Shift of α-Diazoacetates for Wolff Rearrangements – Access to Oxyketenes

Yang Liu,†[a] Zi-Yi Xie,†[a] Lennard Kloene,[b] Cong-Lun Xu,[a] Jian-Peng Tai,[a] Yu Zhu,[a] Bao-Gui Cai,[a] Chongqing Pan,*[a] Rene M. Koenigs,*[b] Jun Xuan*[a]

[a] Anhui Province Key Laboratory of Chemistry for Inorganic/Organic Hybrid Functionalized Materials, College of Chemistry & Chemical Engineering, Anhui University, Hefei, Anhui 230601, China; Key Laboratory of Structure and Functional Regulation of Hybrid Materials (Anhui University), Ministry of Education, Hefei, Anhui 230601, China.

[b] University of Bayreuth, Organic Chemistry II, Universitätsstr. 30, 95447 Bayreuth, Germany

Table of contents

1. General Information 2

2. Preparation and Spectral Data of Starting Materials 3

3. The Condition Optimization 4

4.General Procedure and Spectral Data of Products 5

5. Synthetic applications and gram-scale Synthesis 18

6. Reactions with amine nucleophiles 19

7. Mechanism studies 20

8. DFT calculations 23

9. Crystal data 49

10. References 52

11. Copies of ^{1}H NMR, ^{13}C NMR and ^{19}F NMR Spectra 53

1. General Information

All reactions involving air- or moisture-sensitive reagents or intermediates were carried out in pre-heated glassware under an argon atmosphere using standard Schlenk techniques. All other solvents and reagents were purified according to standard procedures or were used as received from chemical suppliers. The starting materials were synthesized according to literature procedures. The light employed in this work was bought from ahkemi: model KL 100-420 nm, 40 W blue LEDs, λ = 420 nm. All photo-reactions were performed in borosilicate glass irradiation vessel at a distance of ~3 cm from light source. All reactions involving heating are carried out in an oil bath.

Standardized photoreactors:

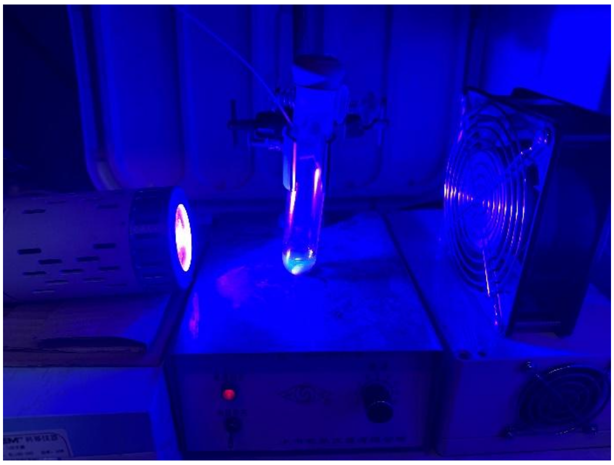

Chromatography: Analytical thin layer chromatography was performed using Qingdao Puke Parting Materials Co. silica gel plates (Silica gel 60 F254). Visualisation was by ultraviolet fluorescence (λ = 254 nm) and/or staining with phosphomolybdic acid or potassium permanganate ($KMnO_4$). Flash column chromatography was performed using 200-300 mesh silica gel.

^{1}H NMR, ^{13}C NMR, and ^{19}F NMR spectra were recorded on a JEOL JNM ECZ400R and ECZ600R at 300 K. Spectra were calibrated relative to solvent's residual proton and carbon chemical shift: $CDCl_3$ (δ = 7.26 ppm for ^{1}H NMR and δ = 77.0 ppm for ^{13}C NMR), CD_3CN (δ = 1.94 ppm for ^{1}H NMR, and δ = 1.3 ppm and 118.3 ppm for ^{13}C NMR). Data are reported as follows: chemical shift δ/ppm, integration (^{1}H only), multiplicity (s = singlet, d = doublet, t = triplet, q = quartet, dd= doublet of doublets, sept = septet, m = multiplet or combinations thereof; ^{13}C signals are singlets unless otherwise stated), coupling constants *J* in Hz, assignment.

High Resolution Mass Spectrometry (HRMS): All were recorded on Thermo Fisher Scientific LTQ Orbitrap XL using an atmospheric-pressure chemical ionization ($APCI^{+}$) or positive electrospray ionization (ESI^{+}). Measured values are reported to 4 decimal places of the calculated value. The calculated values are based on the most abundant isotope.

X-ray Crystallography were collected at 100 K on a Rigaku Oxford Diffraction Supernova Dual Source, Cu at Zero equipped with an AtlasS2 CCD using Cu Kα radiation. The data were collected and processed using CrysAlisPro.

2. Preparation and Spectral Data of Starting Materials

All quinoxalin-2(1*H*)-ones **1** and diazo compounds **2** are prepared according to the known procedures.[1], [2]

Other types of imines, including *N*-heterocycles (**5a-5f**), linear ketimines (**5g-5h**), linear aldimines (**5i-5m**), and hydrazones (**5n**), are prepared by the literature reports.[3-5]

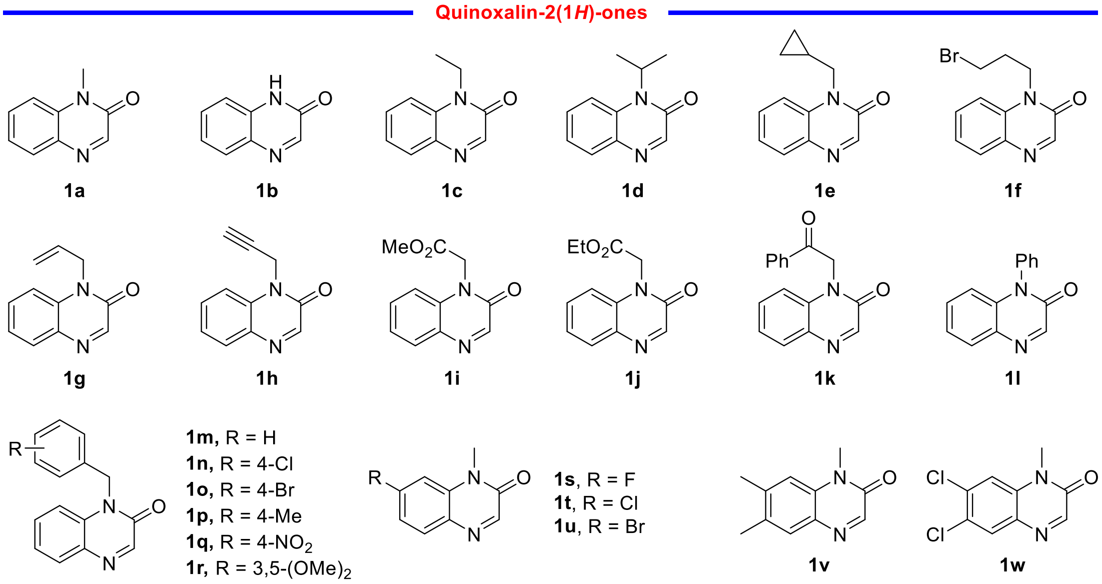

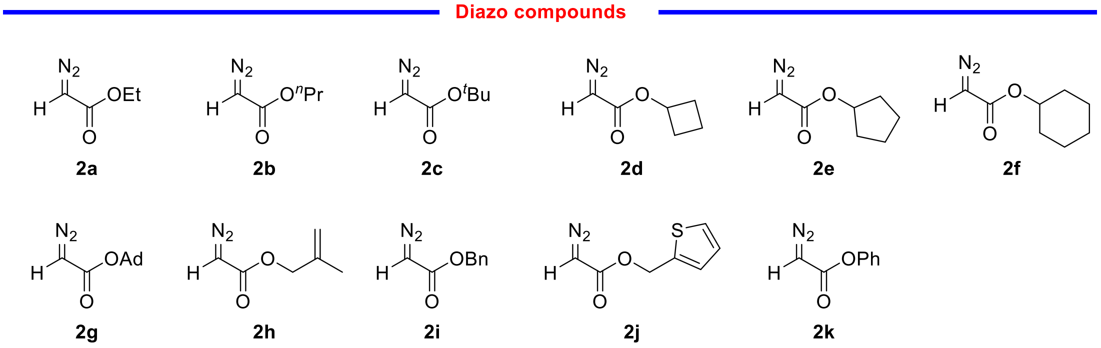

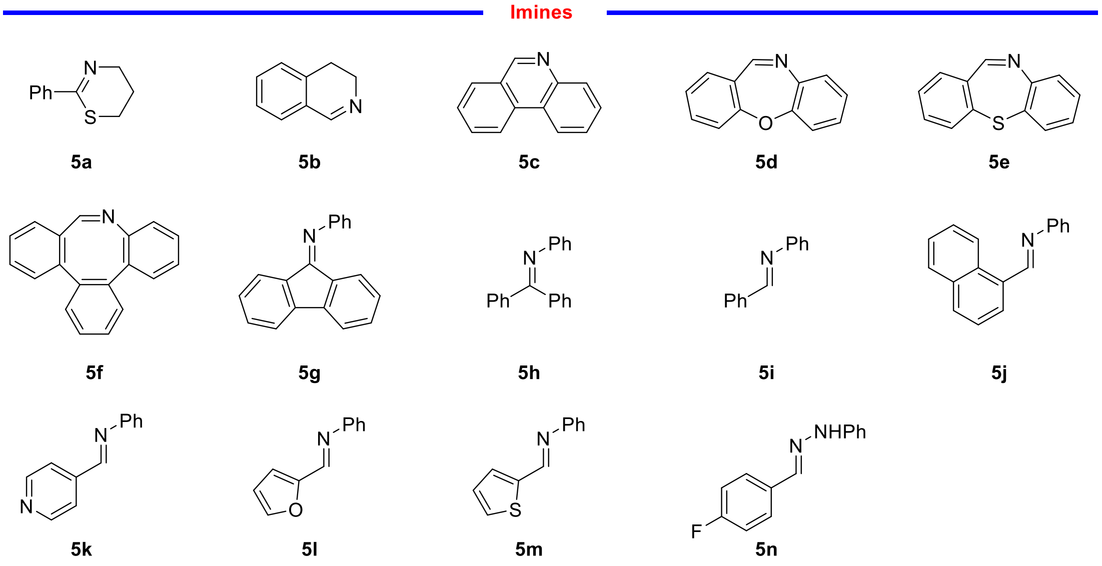

3. The Condition Optimization[a]

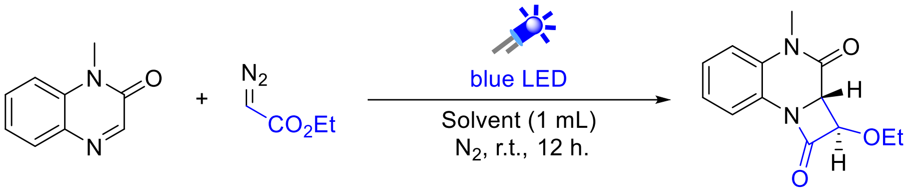

1a 2a 3a

Entry	Light source	Solvent	Molar of 1a	Molar of 2a	T	Yield[b]
1	455 nm	EA	0.2 mmol	0.6 mmol	R.T.	51%
2	455 nm	DCM	0.2 mmol	0.6 mmol	R.T.	25%
3	455 nm	toluene	0.2 mmol	0.6 mmol	R.T.	33%
4	420 nm	EA	0.2 mmol	0.6 mmol	R.T.	79%
5	390 nm	EA	0.2 mmol	0.6 mmol	R.T.	66%
6	480 nm	EA	0.2 mmol	0.6 mmol	R.T.	44%
7	420 nm	EA	0.2 mmol	0.4 mmol	R.T.	61%
8	420 nm	EA	0.2 mmol	0.6 mmol	50 °C	78%
9	No light	EA	0.2 mmol	0.6 mmol	R.T.	N.D.

[a]Reaction conditions: **1a** (0.2 mmol), **2a** (x equiv) in solvent (1.0 mL), with Blue LEDs irradiation at r.t. for 12 h under an N_2 atmosphere. [b] Isolated yield.

4.General Procedure and Spectral Data of Products

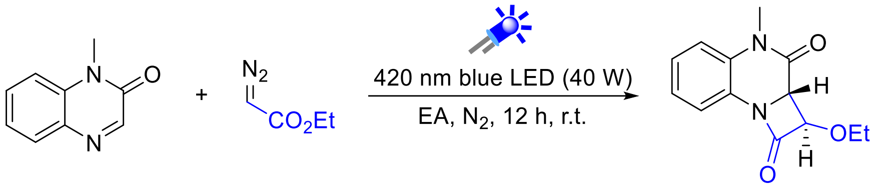

1a **2a** **3a**

General procedure: To a 10 mL Schlenk flask equipped with a magnetic stir bar was added **1a** (32.0 mg, 0.2 mmol, 1.0 equiv.), **2a** (68.5 mg, 0.6 mmol, 3.0 equiv.) and dry EA (1.0 mL). After the resulting mixture was degassed via ‘freeze-pump-thaw’ procedure (3 times), the solution was stirred at room temperature and irradiated with blue LED (λ = 420 nm, 40 W) for 12 h. The solvent was removed by vacuum and the crude product was purified by flash chromatography on silica gel silica: 200~300; eluant: petroleum ether/ethyl acetate (5:1 to 3:1) to provide pure product **3a**.

(±)-*trans*-2-ethoxy-4-methyl-2,2a-dihydro-1*H*-azeto[1,2-*a*]quinoxaline-1,3(4*H*)-dione (3a)

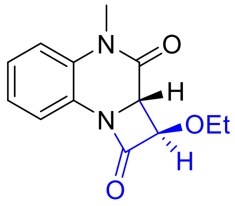

According to ***GP*** with **1a** (32.0 mg, 0.2 mmol, 1.0 equiv.), **2a** (68.5 mg, 0.6 mmol, 3.0 equiv.) in 1.0 mL dry EA for 12 h. Purification by silica gel chromatography afforded the desired **3a** (38.8 mg, 79% yield, dr > 19:1, yellow oil). **^{1}H NMR** (400 MHz, $CDCl_3$, 300K): δ (ppm) = 7.42 (dd, *J* = 7.6, 1.6 Hz, 1H), 7.28 – 7.19 (m, 1H), 7.11 (td, *J* = 7.6, 1.2 Hz, 1H), 7.03 (dd, *J* = 8.4, 1.2 Hz, 1H), 4.95 (d, *J* = 2.0 Hz, 1H), 4.27 (d, *J* = 1.2 Hz, 1H), 3.83 (q, *J* = 6.8 Hz, 2H), 3.40 (s, 3H), 1.32 (t, *J* = 6.8 Hz, 3H). **^{13}C NMR** (100 MHz, $CDCl_3$, 300K): δ (ppm) = 164.8, 164.3, 133.0, 126.2, 123.8, 123.5, 120.7, 115.5, 89.2, 67.2, 57.1, 29.1, 14.9. **HRMS** (ESI) m/z: $[M+H]^+$ Calcd for $C_{13}H_{15}N_2O_3^+$: 247.1077; Found: 247.1078.

(±)-*trans*-4-methyl-2-propoxy-2,2a-dihydro-1*H*-azeto[1,2-*a*]quinoxaline-1,3(4*H*-dione (3b)

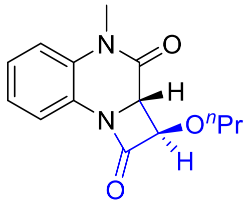

According to ***GP*** with **1a** (32.0 mg, 0.2 mmol, 1.0 equiv.), **2b** (76.9 mg, 0.6 mmol, 3.0 equiv.) in 1.0 mL dry EA for 12 h. Purification by silica gel chromatography afforded the desired **3b** (40.2 mg, 77% yield, dr > 19:1, yellow solid). **^{1}H NMR** (600 MHz, $CDCl_3$, 300K): δ (ppm) = 7.44 (d, *J* = 7.8 Hz, 1H), 7.23 (t, *J* = 7.8 Hz, 1H), 7.12 (t, *J* = 7.8 Hz, 1H), 7.04 (d, *J* = 7.8 Hz, 1H), 4.97 (s, 1H), 4.28 (s, 1H), 3.74 (t, *J* = 7.2 Hz, 2H), 3.41 (s, 3H), 1.76 – 1.70 (m, 2H), 0.97 (t, *J* = 7.2 Hz, 3H). **^{13}C NMR** (150 MHz, $CDCl_3$, 300K): δ (ppm) = 164.9, 164.4, 133.0, 126.3, 123.9, 123.6, 120.8, 115.5, 89.5, 73.3, 57.2, 29.1, 22.6, 10.3. **HRMS** (ESI) m/z: $[M+H]^+$ Calcd for $C_{14}H_{17}N_2O_3^+$: 261.1234; Found: 261.1233.

(±)-*trans*-2-(tert-butoxy)-4-methyl-2,2a-dihydro-1*H*-azeto[1,2-*a*]quinoxaline-1,3(4*H*)-dione (3c)

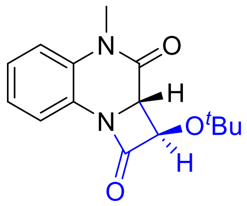

According to ***GP*** with **1a** (32.0 mg, 0.2 mmol, 1.0 equiv.), **2c** (85.3 mg, 0.6 mmol, 3.0 equiv.) in 1.0 mL dry EA for 12 h. Purification by silica gel chromatography afforded the desired **3c** (40.8 mg, 74% yield, dr > 19:1, yellow oil). **^{1}H NMR** (600 MHz, $CDCl_3$, 300K): δ (ppm) = 7.43 (d, *J* = 7.8 Hz, 1H), 7.21 (t, *J* = 7.8 Hz, 1H), 7.11 (t, *J* = 7.8 Hz, 1H), 7.03 (d, *J* = 7.8 Hz, 1H), 5.15 (s, 1H), 4.21 (s, 1H), 3.39 (s, 3H), 1.38 (s, 9H). **^{13}C NMR** (150 MHz, $CDCl_3$, 300K): δ (ppm) = 166.3, 164.7, 133.0, 126.1, 123.8, 123.8, 120.7, 115.4, 83.5, 77.1, 58.6, 29.0, 27.9. **HRMS** (ESI) m/z: $[M+H]^+$ Calcd for $C_{15}H_{19}N_2O_3^+$: 275.1390; Found: 275.1394.

(±)-*trans*-2-cyclobutoxy-4-methyl-2,2a-dihydro-1*H*-azeto[1,2-*a*]quinoxaline-1,3(4*H*)-dione (3d)

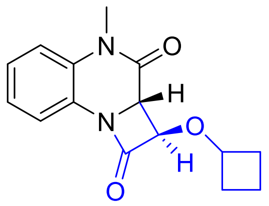

According to ***GP*** with **1a** (32.0 mg, 0.2 mmol, 1.0 equiv.), **2d** (84.1 mg, 0.6 mmol, 3.0 equiv.) in 1.0 mL dry EA for 12 h. Purification by silica gel chromatography afforded the desired **3d** (42.7 mg, 78% yield, dr > 19:1, yellow oil). ^{1}**H NMR** (600 MHz, $CDCl_3$, 300K): δ (ppm) = 7.43 (dd, *J* = 7.8, 1.8 Hz, 1H), 7.23 (td, *J* = 7.8, 1.2 Hz, 1H), 7.12 (td, *J* = 7.8, 1.2 Hz, 1H), 7.04 (dd, *J* = 8.4, 1.2 Hz, 1H), 4.92 (d, *J* = 1.2 Hz, 1H), 4.33 – 4.28 (m, 1H), 4.26 (d, *J* = 1.2 Hz, 1H), 3.40 (s, 3H), 2.37 – 2.30 (m, 2H), 2.14 – 2.08 (m, 2H), 1.79 – 1.73 (m, 1H), 1.59 – 1.50 (m, 1H). ^{13}**C NMR** (150 MHz, $CDCl_3$, 300K): δ (ppm) = 164.9, 164.3, 133.0, 126.3, 123.9, 123.6, 120.8, 115.5, 87.0, 74.3, 57.2, 30.3, 30.3, 29.1, 12.4. **HRMS** (ESI) m/z: $[M+H]^+$ Calcd for $C_{15}H_{17}N_2O_3^+$: 273.1234; Found: 273.1236.

(±)-*trans*-2-(cyclopentyloxy)-4-methyl-2,2a-dihydro-1*H*-azeto[1,2-*a*]quinoxaline-1,3(4*H*)-dione (3e)

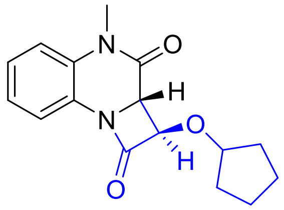

According to ***GP*** with **1a** (32.0 mg, 0.2 mmol, 1.0 equiv.), **2e** (92.5 mg, 0.6 mmol, 3.0 equiv.) in 1.0 mL dry EA for 12 h. Purification by silica gel chromatography afforded the desired **3e** (47.2 mg, 82% yield, dr > 19:1, yellow oil). ^{1}**H NMR** (600 MHz, $CDCl_3$, 300K): δ (ppm) = 7.44 (dd, *J* = 7.8, 1.2 Hz, 1H), 7.23 (td, *J* = 7.8, 1.2 Hz, 1H), 7.12 (dd, *J* = 7.8, 1.2 Hz, 1H), 7.04 (dd, *J* = 7.8, 1.2 Hz, 1H), 4.97 (d, *J* = 1.8 Hz, 1H), 4.33 – 4.29 (m, 1H), 4.26 (d, *J* = 1.2 Hz, 1H), 3.41 (s, 3H), 1.94 – 1.89 (m, 1H), 1.87 – 1.81 (m, 3H), 1.78 – 1.72 (m, 2H), 1.62 – 1.55 (m, 2H). ^{13}**C NMR** (150 MHz, $CDCl_3$, 300K): δ (ppm) = 165.4, 164.6, 133.0, 126.2, 123.9, 123.7, 120.8, 115.5, 88.4, 84.0, 57.9, 32.5, 32.5, 29.1, 23.4, 23.3. **HRMS** (ESI) m/z: $[M+H]^+$ Calcd for $C_{16}H_{19}N_2O_3^+$: 287.1390; Found: 287.1395.

(±)-*trans*-2-(cyclohexyloxy)-4-methyl-2,2a-dihydro-1*H*-azeto[1,2-*a*]quinoxaline-1,3(4*H*)-dione (3f)

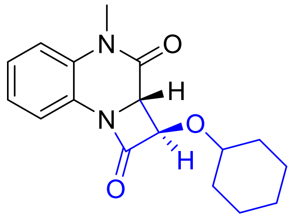

According to ***GP*** with **1a** (32.0 mg, 0.2 mmol, 1.0 equiv.), **2f** (100.9 mg, 0.6 mmol, 3.0 equiv.) in 1.0 mL dry EA for 12 h. Purification by silica gel chromatography afforded the desired **3f** (52.1 mg, 68% yield, dr > 19:1, pale yellow oil). ^{1}**H NMR** (400 MHz, $CDCl_3$, 300K): δ (ppm) = 7.44 (dd, *J* = 7.6, 1.6 Hz, 1H), 7.23 (td, *J* = 7.6, 1.6 Hz, 1H), 7.12 (td, *J* = 7.6, 1.3 Hz, 1H), 7.04 (dd, *J* = 8.4, 1.2 Hz, 1H), 5.04 (d, *J* = 1.6 Hz, 1H), 4.26 (d, *J* = 1.6 Hz, 1H), 3.72 – 3.61 (m, 1H), 3.41 (s, 3H), 2.18 – 2.08 (m, 1H), 2.07 – 1.97 (m, 1H), 1.84 – 1.72 (m, 2H), 1.59 – 1.41 (m, 3H), 1.38 – 1.20 (m, 3H). ^{13}**C NMR** (100 MHz, $CDCl_3$, 300K): δ (ppm) = 165.6, 164.6, 133.0, 126.2, 123.9, 123.7, 120.8, 115.5, 88.0, 80.2, 58.2, 32.2, 32.1, 29.1, 25.5, 23.8, 23.8. **HRMS** (ESI) m/z: $[M+H]^+$ Calcd for $C_{17}H_{21}N_2O_3^+$: 301.1547; Found: 301.1550.

(±)-*trans*-2-((3-adamantan-1-yl)oxy)-4-methyl-2,2a-dihydro-1*H*-azeto[1,2-*a*]quinoxaline-1,3(4*H*)-dione (3g)

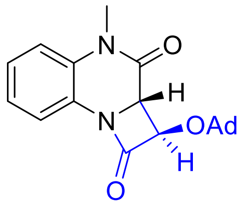

According to ***GP*** with **1a** (32.0 mg, 0.2 mmol, 1.0 equiv.), **2g** (132.2 mg, 0.6 mmol, 3.0 equiv.) in 1.0 mL dry EA for 12 h. Purification by silica gel chromatography afforded the desired **3g** (66.9 mg, 95% yield, dr > 19:1, yellow oil).^{1}**H NMR** (400 MHz, $CDCl_3$, 300K): δ (ppm) = 7.44 (dd, *J* = 7.6, 1.6 Hz, 1H), 7.22 (td, *J* = 7.6, 1.6 Hz, 1H), 7.12 (td, *J* = 7.6, 1.2 Hz, 1H), 7.04 (d, *J* = 8.4 Hz, 1H), 5.30 (d, *J* = 1.6 Hz, 1H), 4.22 (d, *J* = 1.6 Hz, 1H), 3.40 (s, 3H), 2.22 (s, 3H), 2.01 – 1.85 (m, 6H), 1.66 (m, 6H). ^{13}**C NMR** (100 MHz, $CDCl_3$, 300K): δ (ppm) = 166.6, 164.8, 133.0, 126.1, 123.8, 120.8, 115.4, 81.9, 76.3, 58.7, 41.7, 36.1, 30.7, 29.0. **HRMS** (ESI) m/z: $[M+H]^+$ Calcd for $C_{21}H_{25}N_2O_3^+$: 353.1860; Found: 353.1862.

(±)-*trans*-4-methyl-2-((2-methylallyl)oxy)-2,2a-dihydro-1*H*-azeto[1,2-*a*]quinoxaline-1,3(4*H*)-dione (3h)

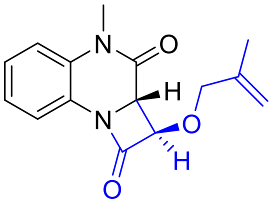

According to ***GP*** with **1a** (32.0 mg, 0.2 mmol, 1.0 equiv.), **2h** (84.1 mg, 0.6 mmol, 3.0 equiv.) in 1.0 mL dry EA for 12 h. Purification by silica gel chromatography afforded the desired **3h** (33.6 mg, 61% yield, dr > 19:1, orange oil). **^{1}H NMR** (600 MHz, $CDCl_3$, 300K): δ (ppm) = 7.44 (dd, J = 7.8, 1.2 Hz, 1H), 7.23 (td, J = 7.8, 1.2 Hz, 1H), 7.13 (td, J = 7.8, 1.2 Hz, 1H), 7.04 (dd, J = 8.2, 1.2 Hz, 1H), 5.14 – 5.13 (m, 1H), 5.11 (d, J = 2.4 Hz, 1H), 5.01 – 5.00 (m, 1H), 4.31 (d, J = 1.2 Hz, 1H), 4.24 – 4.18 (m, 2H), 3.41 (s, 3H), 1.81 (s, 3H) **^{13}C NMR** (150 MHz, $CDCl_3$, 300K): δ (ppm) = 164.8, 164.3, 140.3, 133.0, 126.3, 123.9, 123.6, 120.8, 115.5, 114.1, 88.3, 74.9, 57.2, 29.1, 19.4. **HRMS** (ESI) m/z: $[M+H]^+$ Calcd for $C_{15}H_{17}N_2O_3^+$: 273.1234; Found: 273.1238.

(±)-*trans*-2-(benzyloxy)-4-methyl-2,2a-dihydro-1*H*-azeto[1,2-*a*]quinoxaline-1,3(4*H*)-dione (3i)

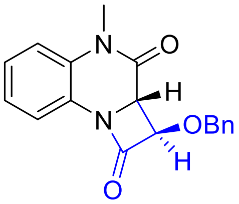

According to ***GP*** with **1a** (32.0 mg, 0.2 mmol, 1.0 equiv.), **2i** (105.7 mg, 0.6 mmol, 3.0 equiv.) in 1.0 mL dry EA for 12 h. Purification by silica gel chromatography afforded the desired **3i** (52.6 mg, 85% yield, dr > 19:1, pale yellow oil).**^{1}H NMR** (400 MHz, $CDCl_3$, 300K): δ (ppm) = 7.50 – 7.42 (m, 3H), 7.42 – 7.30 (m, 3H), 7.24 (td, J = 7.6, 1.2 Hz, 1H),, 7.13 (td, J = 7.6, 1.2 Hz, 1H), 7.05 (dd, J = 8.4, 1.2 Hz, 1H), 5.08 (d, J = 1.6 Hz, 1H), 4.89 – 4.81 (m, 2H), 4.34 (d, J = 1.6 Hz, 1H), 3.41 (s, 3H). **^{13}C NMR** (100 MHz, $CDCl_3$, 300K): δ (ppm) = 164.5, 164.2, 136.1, 133.0, 128.6, 128.3, 128.3, 126.3, 123.9, 123.6, 120.8, 115.6, 88.4, 73.0, 57.2, 29.1. **HRMS** (ESI) m/z: $[M+H]^+$ Calcd for $C_{18}H_{17}N_2O_3^+$: 309.1234; Found: 309.1237.

(±)-*trans*-4-methyl-2-(thiophen-2-ylmethoxy)-2,2a-dihydro-1*H*-azeto[1,2-*a*]quinoxaline-1,3(4*H*)-dione (3j)

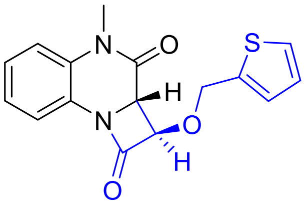

According to ***GP*** with **1a** (32.0 mg, 0.2 mmol, 1.0 equiv.), **2j** (109.3 mg, 0.6 mmol, 3.0 equiv.) in 1.0 mL dry EA for 12 h. Purification by silica gel chromatography afforded the desired **3j** (47.4 mg, 79% yield, dr > 19:1, yellow oil). **^{1}H NMR** (400 MHz, $CDCl_3$, 300K): δ (ppm) = 7.44 (dd, J = 8.0, 1.6 Hz, 1H), 7.35 (dd, J = 5.2, 1.2 Hz, 1H), 7.26 – 7.18 (m, 2H), 7.13 (td, J = 7.6, 1.2 Hz, 1H), 7.04 (dd, J = 8.0, 1.2 Hz, 1H), 7.01 (dd, J = 5.2, 3.6 Hz, 1H), 5.09 (d, J = 2.0 Hz, 1H), 5.02 (d, J = 12.4 Hz, 1H), 4.98 (d, J = 12.4 Hz, 1H), 4.32 (d, J = 2.0 Hz, 1H), 3.41 (s, 3H). **^{13}C NMR** (100 MHz, $CDCl_3$, 300K): δ (ppm) = 164.3, 164.1, 138.2, 133.0, 128.5, 127.0, 126.9, 126.4, 123.9, 123.5, 120.8, 115.6, 87.6, 67.3, 57.2, 29.1. **HRMS** (ESI) m/z: $[M+H]^+$ Calcd for $C_{16}H_{15}N_2O_3S^+$: 315.0798; Found: 315.0799.

(±)-*trans*-4-benzyl-2-ethoxy-2,2a-dihydro-1*H*-azeto[1,2-*a*]quinoxaline-1,3(4*H*)-dione (3k)

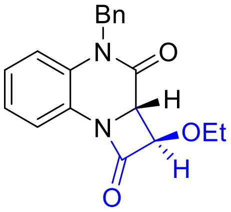

According to ***GP*** with **1m** (47.3 mg, 0.2 mmol, 1.0 equiv.), **2a** (68.5 mg, 0.6 mmol, 3.0 equiv.) in 1.0 mL dry EA for 12 h. Purification by silica gel chromatography afforded the desired **3k** (29.2 mg, 45% yield, dr > 19:1, yellow oil). **^{1}H NMR** (400 MHz, $CDCl_3$, 300 K) δ (ppm) = 7.46 – 7.40 (m, 1H), 7.34 – 7.28 (m, 2H), 7.25 – 7.16 (m, 3H), 7.08 – 7.03 (m, 2H), 6.95 – 6.90 (m, 1H), 5.49 (d, J = 16.4 Hz, 1H), 5.03 (d, J = 2.0 Hz, 1H), 4.87 (d, J = 16.0 Hz, 1H), 4.41 (d, J = 2.0 Hz, 1H), 3.85 (q, J = 6.8 Hz, 2H), 1.33 (t, J = 6.8 Hz, 3H). **^{13}C NMR** (100 MHz, $CDCl_3$, 300 K) δ (ppm) = 164.7, 164.6, 135.6, 132.3, 129.0, 127.6, 126.3, 126.2, 124.0, 123.7, 120.8, 116.4, 89.4, 67.3, 57.3, 45.9, 14.9. **HRMS** (ESI) m/z: $[M+H]^+$ Calcd for $C_{19}H_{19}N_2O_3^+$: 323.1390; Found: 323.1389.

(±)-*trans*-4-methyl-2-phenoxy-2,2a-dihydro-1*H*-azeto[1,2-*a*]quinoxaline-1,3(4*H*)-dione (3l)

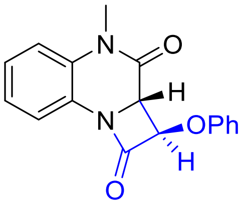

According to ***GP*** with **1a** (32.0 mg, 0.2 mmol, 1.0 equiv.), **2k** (97.3 mg, 0.6 mmol, 3.0 equiv.) in 1.0 mL dry EA for 12 h. Purification by silica gel chromatography afforded the desired **3l** (31.7 mg, 54% yield, dr > 19:1, yellow oil). **^{1}H NMR** (600 MHz, $CDCl_3$, 300 K) δ (ppm) = 7.50 (dd, J = 7.8, 1.2 Hz, 1H), 7.39 – 7.35 (m, 4H), 7.28 (td, J = 7.8, 1.8 Hz, 1H), 7.17 (td, J = 7.8, 1.2 Hz, 1H), 7.11 – 7.06 (m, 2H), 5.59 (d, J = 1.8 Hz, 1H), 4.45 (d, J = 1.8 Hz, 1H), 3.47 (s, 3H). **^{13}C NMR** (150 MHz, $CDCl_3$, 300 K) δ

(ppm) = 163.9, 162.9, 157.3, 133.0, 129.8, 126.7, 124.0, 123.4, 122.7, 121.0, 115.7, 115.6, 86.5, 57.1, 29.3. **HRMS** (ESI) m/z: $[M+H]^+$ Calcd for $C_{17}H_{15}N_2O_3^+$: 295.1077; Found: 295.1076.

(±)-*trans*-2-((3-adamantan-1-yl)oxy)-3-hydroxy-2,2a-dihydro-1*H*-azeto[1,2-*a*]quinoxalin-1-one (4a)

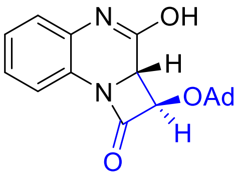

According to ***GP*** with **1b** (29.2 mg, 0.2 mmol, 1.0 equiv.), **2g** (132.2 mg, 0.6 mmol, 3.0 equiv.) in 1.0 mL dry EA for 12 h. Purification by silica gel chromatography afforded the desired **4a** (56.4 mg, 83% yield, dr > 19:1, white solid). **^{1}H NMR** (400 MHz, $CDCl_3$, 300 K) δ (ppm) = 8.89 (s, 1H), 7.42 (dd, J = 7.6, 1.6 Hz, 1H), 7.15 – 7.06 (m, 2H), 6.88 (dd, J = 7.6, 1.6 Hz, 1H), 5.32 (d, J = 1.6 Hz, 1H), 4.27 (d, J = 1.6 Hz, 1H), 2.22 (s, 3H), 2.00 – 1.85 (m, 6H), 1.69 – 1.60 (s, 6H). **^{13}C NMR** (100 MHz, $CDCl_3$, 300 K) δ (ppm) = 167.3, 165.8, 130.0, 126.1, 124.2, 122.7, 120.7, 115.8, 81.3, 76.3, 59.1, 41.7, 36.1, 30.7. **HRMS** (ESI) m/z: $[M+H]^+$ Calcd for $C_{20}H_{23}N_2O_3^+$: 339.1703; Found: 339.1705.

(±)-*trans*-2-((3-adamantan-1-yl)oxy)-4-ethyl-2,2a-dihydro-1H-azeto[1,2-a]quinoxaline-1,3(4H)-dione (4b)

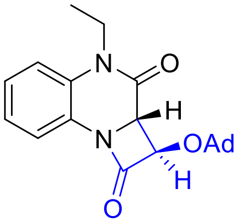

According to ***GP*** with **1c** (34.8 mg, 0.2 mmol, 1.0 equiv.), **2g** (132.2 mg, 0.6 mmol, 3.0 equiv.) in 1.0 mL dry EA for 12 h. Purification by silica gel chromatography afforded the desired **4b** (61.5 mg, 84% yield, dr > 19:1, white solid). **^{1}H NMR** (400 MHz, $CDCl_3$, 300 K): δ (ppm) = 7.44 (dd, J = 7.6, 1.6 Hz, 1H), 7.21 (td, J = 8.0, 1.6 Hz, 1H), 7.10 (td, J = 8.0, 1.2 Hz, 1H), 7.44 (dd, J = 8.4, 1.2 Hz, 1H), 5.29 (d, J = 2.0 Hz, 1H), 4.19 (d, J = 1.6 Hz, 1H), 4.17 – 4.08 (m, 1H), 3.97 – 3.88 (m, 1H), 2.22 (s, 3H), 2.01 – 1.84 (m, 6H), 1.68 – 1.62 (m, 6H), 1.28 (t, J = 7.2 Hz, 3H). **^{13}C NMR** (100 MHz, $CDCl_3$, 300 K): δ (ppm) = 166.6, 164.2, 131.8, 126.1, 124.0, 123.7, 121.1, 115.2, 81.9, 76.3, 58.6, 41.7, 37.2, 36.1, 30.7, 12.5. **HRMS** (ESI) m/z: $[M+H]^+$ Calcd for $C_{22}H_{27}N_2O_3^+$: 367.2016; Found: 367.2011.

(±)-*trans*-2-((3-adamantan-1-yl)oxy)-4-isopropyl-2,2a-dihydro-1*H*-azeto[1,2-*a*]quinoxaline-1,3(4*H*)-dione (4c)

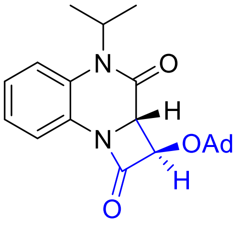

According to ***GP*** with **1d** (37.6 mg, 0.2 mmol, 1.0 equiv.), **2g** (132.2 mg, 0.6 mmol, 3.0 equiv.) in 1.0 mL dry EA for 12 h. Purification by silica gel chromatography afforded the desired **4c** (47.1 mg, 62% yield, dr > 19:1, colorless oil). **^{1}H NMR** (600 MHz, $CDCl_3$, 300 K): δ (ppm) = 7.32 (dd, J = 7.2, 2.4 Hz, 1H), 7.20 (dd, J = 7.2, 2.4 Hz, 1H), 7.14 – 7.09 (m, 2H), 5.41 (sept, J = 6.0 Hz, 1H), 5.17 (d, J = 1.8 Hz, 1H), 4.11 (d, J = 1.8 Hz, 1H), 2.22 (s, 3H), 1.95 – 1.84 (m, 6H), 1.71 – 1.60 (m, 6H), 1.38(d, J = 6.0 Hz, 3H), 1.36(d, J = 6.0 Hz, 3H). **^{13}C NMR** (150 MHz, $CDCl_3$, 300 K): δ (ppm) = 169.2, 158.5, 136.0, 126.0, 125.8, 125.6, 125.2, 119.7, 81.7, 75.9, 69.5, 55.3, 41.7, 36.2, 30.7, 22.0, 21.7. **HRMS** (ESI) m/z: $[M+H]^+$ Calcd for $C_{23}H_{29}N_2O_3^+$: 381.2173; Found: 381.2173.

(±)-*trans*-2-((3-adamantan-1-yl)oxy)-4-(cyclopropylmethyl)-2,2a-dihydro-1*H*-azeto[1,2-*a*]quinoxaline-1,3(4*H*)-dione (4d)

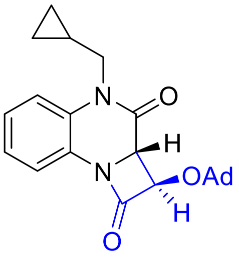

According to ***GP*** with **1e** (40.0 mg, 0.2 mmol, 1.0 equiv.), **2g** (132.2 mg, 0.6 mmol, 3.0 equiv.) in 1.0 mL dry EA for 12 h. Purification by silica gel chromatography afforded the desired **4d** (59.4 mg, 76% yield, dr > 19:1, colorless oil) **^{1}H NMR** (400 MHz, $CDCl_3$, 300 K): δ (ppm) = 7.44 (dd, J = 7.6, 1.6 Hz, 1H), 7.23 – 7.15 (m, 2H), 7.10 (td, J = 7.6, 2.0 Hz, 1H), 5.28 (d, J = 1.6 Hz, 1H), 4.21 (d, J = 1.6 Hz, 1H), 3.97 (dd, J = 14.4, 6.8 Hz, 1H), 3.81 (dd, J = 14.4, 6.8 Hz, 1H), 2.21 (s, 3H), 2.00 – 1.85 (m, 6H), 1.65 (s, 6H), 1.19 – 1.08 (m, 1H), 0.58 – 0.39 (m, 4H). **^{13}C NMR** (100 MHz, $CDCl_3$, 300 K): δ (ppm) = 166.6, 164.5, 132.3, 126.0, 123.9, 123.6, 121.0, 115.7, 81.9, 76.2, 58.5, 46.0, 41.7, 36.1, 30.7, 9.5, 4.3, 4.0. **HRMS** (ESI) m/z: $[M+H]^+$ Calcd for $C_{24}H_{29}N_2O_3^+$: 393.2173; Found: 393.2167.

(±)-*trans*-2-((3-adamantan-1-yl)oxy)-4-(3-bromopropyl)-2,2a-dihydro-1*H*-azeto[1,2-*a*]quinoxaline-1,3(4*H*)-dione (4e)

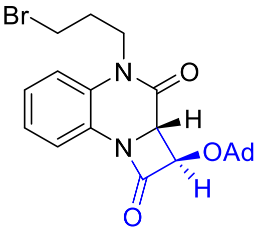

According to ***GP*** with **1f** (53.4 mg, 0.2 mmol, 1.0 equiv.), **2g** (132.2 mg, 0.6 mmol, 3.0 equiv.) in 1.0 mL dry EA for 12 h. Purification by silica gel chromatography afforded the desired **4e** (84.2 mg, 92% yield, dr > 19:1, yellow oil) **^{1}H NMR** (400 MHz, $CDCl_3$, 300 K): δ (ppm) = 7.44 (dd, *J* = 8.0, 1.6 Hz, 1H), 7.22 (td, *J* = 8.0, 1.6 Hz, 1H), 7.14 – 7.09 (m, 2H), 5.30 (d, *J* = 2.0 Hz, 1H), 4.21 (d, *J* = 1.6 Hz, 1H), 4.20 – 3.99 (m, 2H), 3.55 – 3.43 (m, 2H), 2.31 – 2.16 (m, 5H), 1.99 – 1.83 (m, 6H), 1.65 (s, 6H). **^{13}C NMR** (100 MHz, $CDCl_3$, 300 K): δ (ppm) = 166.4, 164.7, 131.8, 126.3, 124.0, 123.9, 121.1, 115.1, 82.0, 76.3, 58.6, 41.6, 41.2, 36.1, 30.7, 30.4, 29.8. **HRMS** (ESI) m/z: $[M+H]^+$ Calcd for $C_{23}H_{28}BrN_2O_3^+$: 459.1278; Found: 459.1273.

(±)-*trans*-2-((3-adamantan-1-yl)oxy)-4-allyl-2,2a-dihydro-1*H*-azeto[1,2-a]quinoxaline-1,3 (4*H*)-dione (4f)

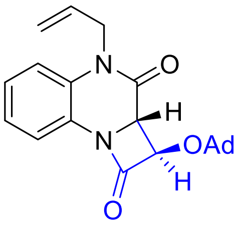

According to ***GP*** with **1g** (37.2 mg, 0.2 mmol, 1.0 equiv.), **2g** (132.2 mg, 0.6 mmol, 3.0 equiv.) in 1.0 mL dry EA for 12 h. Purification by silica gel chromatography afforded the desired **4f** (84.0 mg, 96% yield, dr > 19:1, pale yellow oil). **^{1}H NMR** (400 MHz, $CDCl_3$, 300 K): δ (ppm) = 7.43 (dd, *J* = 7.6, 1.6 Hz, 1H), 7.17 (td, *J* = 7.6, 1.6 Hz, 1H), 7.09 (td, *J* = 7.6, 1.2 Hz, 1H), 7.02 (dd, *J* = 8.0, 1.2 Hz, 1H), 5.93 – 5.81 (m, 1H), 5.31 (d, *J* = 2.0 Hz, 1H), 5.27 – 5.17 (m, 2H), 4.85 (dd, *J* = 16.8, 4.8 Hz, 1H), 4.33 (dd, *J* = 16.8, 5.2 Hz, 1H), 4.24 (d, *J* = 2.0 Hz, 1H), 2.21 (s, 3H), 2.00 – 1.85 (m, 6H), 1.64 (s, 6H). **^{13}C NMR** (100 MHz, $CDCl_3$, 300 K): δ (ppm) = 166.4, 164.5, 132.2, 131.4, 126.1, 123.8, 120.8, 117.3, 116.0, 81.9, 76.3, 58.6, 44.6, 41.6, 36.1, 30.6. **HRMS** (ESI) m/z: $[M+H]^+$ Calcd for $C_{23}H_{27}N_2O_3^+$: 379.2016; Found: 379.2011.

(±)-*trans*-2-((3-adamantan-1-yl)oxy)-4-(prop-2-yn-1-yl)-2,2a-dihydro-1*H*-azeto[1,2-*a*] quinoxaline-1,3(4*H*)-dione (4g)

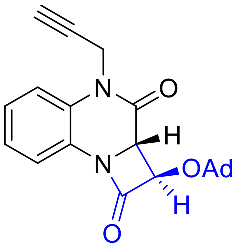

According to ***GP*** with **1h** (36.8 mg, 0.2 mmol, 1.0 equiv.), **2g** (132.2 mg, 0.6 mmol, 3.0 equiv.) in 1.0 mL dry EA for 12 h. Purification by silica gel chromatography afforded the desired **4g** (66.3 mg, 84% yield, dr > 19:1, colorless oil). **^{1}H NMR** (400 MHz, $CDCl_3$, 300 K) δ (ppm) = 7.47 (d, *J* = 7.6 Hz, 1H), 7.29 – 7.24 (m, 2H), 7.19 – 7.14 (m, 1H), 5.33 (d, *J* = 2.0 Hz, 1H), 5.00 (dd, *J* = 17.6, 2.4 Hz, 1H), 4.51 (dd, *J* = 17.6, 2.4 Hz, 1H), 4.27 (d, *J* = 1.6 Hz, 1H), 2.29 (t, *J* = 2.4 Hz, 1H), 2.24 (s, 3H), 2.02 - 1.87 (m, 6H), 1.70 – 1.65 (m, 6H). **^{13}C NMR** (100 MHz, $CDCl_3$, 300 K) δ (ppm) = 166.3, 164.3, 131.5, 126.2, 124.3, 123.9, 121.0, 115.9, 81.9, 77.5, 76.4, 72.7, 58.8, 41.6, 36.1, 31.6, 30.7. **HRMS** (ESI) m/z: $[M+H]^+$ Calcd for $C_{23}H_{25}N_2O_3^+$: 377.1860; Found: 377.1861.

(±)-*trans*-methyl 2-(2-((3-adamantan-1-yl)oxy)-1,3-dioxo-1,2,2a,3-tetrahydro-4*H*-azeto[1,2-*a*] quinoxalin-4-yl)acetate (4h)

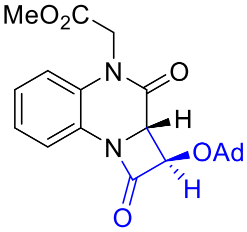

According to ***GP*** with **1i** (43.6 mg, 0.2 mmol, 1.0 equiv.), **2g** (132.2 mg, 0.6 mmol, 3.0 equiv.) in 1.0 mL dry EA for 12 h. Purification by silica gel chromatography afforded the desired **4h** (77.1 mg, 94% yield, dr > 19:1, white solid). **^{1}H NMR** (400 MHz, $CDCl_3$, 300 K) δ (ppm) = 7.46 (dd, *J* = 7.6, 1.6 Hz, 1H), 7.20 – 7.10 (m, 2H), 6.78 (dd, *J* = 8.0, 1.2 Hz, 1H), 5.31 (d, *J* = 1.6 Hz, 1H), 5.00 (d, *J* = 17.6 Hz, 1H), 4.42 (d, *J* = 17.6 Hz, 1H), 4.30 (d, *J* = 1.6 Hz, 1H), 3.77 (s, 3H), 2.21 (s, 3H), 2.00 – 1.84 (m, 6H), 1.64 (s, 6H). **^{13}C NMR** (100 MHz, $CDCl_3$, 300 K) δ (ppm) = 168.2, 166.2, 165.2, 132.1, 126.2, 124.2, 123.8, 121.1, 114.8, 81.9, 76.4, 58.7, 52.7, 43.5, 41.6, 41.1, 36.1, 36.0, 36.0, 30.8, 30.6. **HRMS** (ESI) m/z: $[M+H]^+$ Calcd for $C_{23}H_{27}N_2O_5^+$: 411.1914; Found: 411.1902.

(±)-*trans*-ethyl 2-(-2-((3-adamantan-1-yl)oxy)-1,3-dioxo-1,2,2a,3-tetrahydro-4*H*-azeto[1,2-a]

quinoxalin-4-yl)acetate (4i)

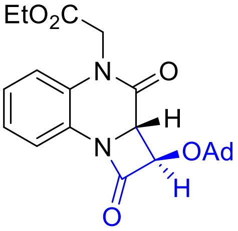

According to ***GP*** with **1j** (46.4 mg, 0.2 mmol, 1.0 equiv.), **2g** (132.2 mg, 0.6 mmol, 3.0 equiv.) in 1.0 mL dry EA for 12 h. Purification by silica gel chromatography afforded the desired **4i** (62.6 mg, 74% yield, dr > 19:1, white solid). **^{1}H NMR** (400 MHz, $CDCl_3$, 300 K) δ (ppm) = 7.45 (dd, *J* = 8.0, 1.6 Hz, 1H), 7.20 – 7.09 (m, 2H), 6.78 (dd, *J* = 8.0, 1.2 Hz, 1H), 5.31 (d, *J* = 1.6 Hz, 1H), 4.99 (d, *J* = 17.6 Hz, 1H), 4.40 (d, *J* = 17.6 Hz, 1H), 4.30 (d, *J* = 1.6 Hz, 1H), 4.23 (q, *J* = 7.2 Hz, 2H), 2.21 (s, 3H), 2.00 – 1.83 (m, 6H), 1.64 (s, 6H), 1.27 (t, *J* = 7.2 Hz, 3H). **^{13}C NMR** (100 MHz, $CDCl_3$, 300 K) δ (ppm) = 167.7, 166.2, 165.2, 132.1, 126.2, 124.2, 123.8, 121.1, 114.9, 81.9, 76.4, 61.9, 58.6, 43.7, 41.6, 36.1, 30.6, 14.1. **HRMS** (ESI) m/z: $[M+H]^+$ Calcd for $C_{24}H_{29}N_2O_5^+$: 425.2071; Found: 425.2069.

(±)-*trans*-2-((3-adamantan-1-yl)oxy)-4-(2-oxo-2-phenylethyl)-2,2a-dihydro-1*H*-azeto[1,2-*a*] quinoxaline-1,3(4*H*)-dione (4j)

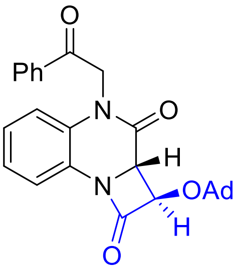

According to ***GP*** with **1k** (52.9 mg, 0.2 mmol, 1.0 equiv.), **2g** (132.2 mg, 0.6 mmol, 3.0 equiv.) in 1.0 mL dry EA for 12 h. Purification by silica gel chromatography afforded the desired **4j** (46.9 mg, 51% yield, dr > 19:1, white solid). **^{1}H NMR** (400 MHz, $CDCl_3$, 300 K) δ (ppm) = 8.05 – 8.01 (m, 2H), 7.69 – 7.63 (m, 1H), 7.57 – 7.44 (m, 3H), 7.14 – 7.06 (m, 2H), 6.68 – 6.60 (m, 1H), 5.76 (d, *J* = 18.0 Hz, 1H), 5.33 (d, *J* = 1.6 Hz, 1H), 5.05 (d, *J* = 18.0 Hz, 1H), 4.37 (d, *J* = 1.6 Hz, 1H), 2.20 (s, 3H), 2.00 – 1.85 (m, 6H), 1.67 – 1.61 (s, 6H). **^{13}C NMR** (100 MHz, $CDCl_3$, 300 K) δ (ppm) = 191.7, 166.3 165.5, 134.4, 134.2, 132.3, 129.0, 128.0, 126.2, 124.0, 123.9, 121.1, 115.2, 81.9, 76.4, 58.7, 48.8, 41.6, 36.1, 30.6. **HRMS** (ESI) m/z: $[M+H]^+$ Calcd for $C_{28}H_{29}N_2O_4^+$: 457.2122; Found: 457.2118.

(±)-*trans*-2-((3-adamantan-1-yl)oxy)-4-phenyl-2,2a-dihydro-1*H*-azeto[1,2-*a*]quinoxaline-1,3 (4*H*)-dione (4k)

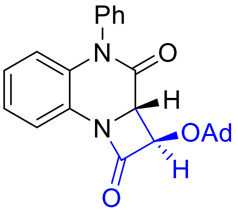

According to ***GP*** with **1l** (44.4 mg, 0.2 mmol, 1.0 equiv.), **2g** (132.2 mg, 0.6 mmol, 3.0 equiv.) in 1.0 mL dry EA for 12 h. Purification by silica gel chromatography afforded the desired **4k** (53.5 mg, 64% yield, dr > 19:1, white solid). **^{1}H NMR** (400 MHz, $CDCl_3$, 300 K) δ (ppm) = 7.61 – 7.46 (m, 4H), 7.36 – 7.27 (m, 1H), 7.25 – 7.12 (m, 1H), 7.25 – 7.05 (m, 1H), 7.00 (dd, *J* = 8.0, 1.6 Hz, 1H), 6.36 (d, *J* = 8.4 Hz, 1H), 5.41 (d, *J* = 1.6 Hz, 1H), 4.42 (d, *J* = 1.6 Hz, 1H), 2.19 (s, 3H), 2.01 – 1.85 (m, 6H), 1.65 – 1.60 (s, 6H). **^{13}C NMR** (100 MHz, $CDCl_3$, 300 K) δ (ppm) = 166.5, 164.7, 136.6, 134.1, 130.3, 129.0, 125.8, 124.0, 123.4, 120.7, 117.4, 82.0, 76.5, 59.4, 41.6, 36.0, 30.7. **HRMS** (ESI) m/z: $[M+H]^+$ Calcd for $C_{26}H_{27}N_2O_3^+$: 415.2016; Found: 415.2013.

(±)-*trans*-2-((3-adamantan-1-yl)oxy)-4-benzyl-2,2a-dihydro-1*H*-azeto[1,2-*a*]quinoxaline-1,3 (4*H*)-dione (4l)

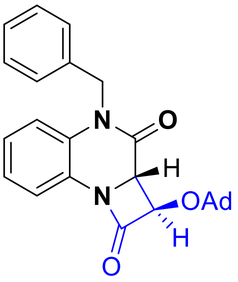

According to ***GP*** with **1m** (47.3 mg, 0.2 mmol, 1.0 equiv.), **2g** (132.2 mg, 0.6 mmol, 3.0 equiv.) in 1.0 mL dry EA for 12 h. Purification by silica gel chromatography afforded the desired **4l** (38.8 mg, 76% yield, dr > 19:1, white solid). **^{1}H NMR** (400 MHz, $CDCl_3$, 300 K) δ (ppm) = 7.49 – 7.43 (m, 1H), 7.36 – 7.31 (m, 2H), 7.29 – 7.20 (m, 3H), 7.09 – 7.05 (m, 2H), 6.95 – 6.90 (m, 1H), 5.52 (d, *J* = 16.0 Hz, 1H), 5.38 (d, *J* = 1.6 Hz, 1H), 4.88 (d, *J* = 16.4 Hz, 1H), 4.35 (d, *J* = 1.6 Hz, 1H), 2.23 (s, 3H), 2.03 – 1.88 (m, 6H), 1.69 – 1.63 (s, 6H). **^{13}C NMR** (100 MHz, $CDCl_3$, 300 K) δ (ppm) = 166.4, 165.0, 135.7, 132.4, 128.9, 127.5, 126.2, 126.1, 123.9, 120.8, 116.3, 82.1, 76.4, 58.7, 45.9, 41.7, 36.1, 30.7. **HRMS** (ESI) m/z: $[M+H]^+$ Calcd for $C_{27}H_{29}N_2O_3^+$: 429.2173; Found: 429.2169.

(±)-*trans*-2-((3-adamantan-1-yl)oxy)-4-(4-chlorobenzyl)-2,2a-dihydro-1*H*-azeto[1,2-*a*] quinoxaline-1,3(4*H*)-dione (4m)

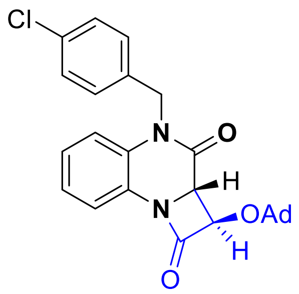

According to ***GP*** with **1n** (54.1 mg, 0.2 mmol, 1.0 equiv.), **2g** (132.2 mg, 0.6 mmol, 3.0 equiv.) in 1.0 mL dry EA for 12 h. Purification by silica gel chromatography afforded the desired **4m** (70.1 mg, 76% yield, dr > 19:1, white solid). **^{1}H NMR** (400 MHz, $CDCl_3$, 300 K) δ (ppm) = 7.48 – 7.44 (m, 1H), 7.31 (d, *J* = 8.4 Hz, 2H), 7.16 (d, *J* = 8.4 Hz, 2H), 7.11 – 7.07 (m, 2H), 6.90 – 6.86 (m, 1H), 5.46 (d, *J* = 16.0 Hz, 1H), 5.37 (d, *J* = 2.0 Hz, 1H), 4.86 (d, *J* = 16.4 Hz, 1H), 4.34 (d, *J* = 1.6 Hz, 1H), 2.22 (s, 3H), 2.01 – 1.87 (m, 6H), 1.69 – 1.63 (s, 6H). **^{13}C NMR** (100 MHz, $CDCl_3$, 300 K) δ (ppm) = 166.3, 165.0, 134.2, 133.4, 132.2, 129.1, 127.7, 126.1, 124.1, 124.0, 121.0, 116.0, 82.1, 76.4, 58.7, 45.4, 41.7, 36.1, 30.7. **HRMS** (ESI) m/z: $[M+H]^+$ Calcd for $C_{27}H_{28}ClN_2O_3^+$: 463.1783; Found: 463.1782.

(±)-*trans*-2-(((3*R*)-adamantan-1-yl)oxy)-4-(4-bromobenzyl)-2,2a-dihydro-1*H*-azeto[1,2-*a*]quinoxaline-1,3(4*H*)-dione (4n)

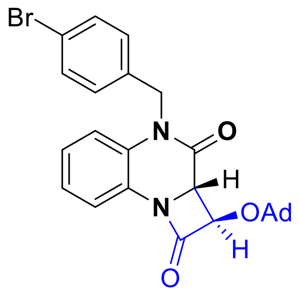

According to ***GP*** with **1o** (63.0 mg, 0.2 mmol, 1.0 equiv.), **2g** (132.2 mg, 0.6 mmol, 3.0 equiv.) in 1.0 mL dry EA for 12 h. Purification by silica gel chromatography afforded the desired **4n** (81.1 mg, 80% yield, dr > 19:1, white solid). **^{1}H NMR** (400 MHz, $CDCl_3$, 300 K) δ (ppm) = 7.48 – 7.43 (m, 3H), 7.12 – 7.07 (m, 4H), 6.89 – 6.84 (m, 1H), 5.44 (d, *J* = 16.4 Hz, 1H), 5.37 (d, *J* = 2.0 Hz, 1H), 4.84 (d, *J* = 16.4 Hz, 1H), 4.34 (d, *J* = 1.6 Hz, 1H), 2.22 (s, 3H), 2.00 – 1.87 (m, 6H), 1.69 – 1.63 (s, 6H). **^{13}C NMR** (100 MHz, $CDCl_3$, 300 K) δ (ppm) = 166.3, 165.0, 134.8, 132.1, 132.1, 128.1, 126.1, 124.1, 124.0, 121.4, 121.0, 116.0, 82.1, 76.4, 58.7, 45.5, 41.7, 36.1, 30.7. **HRMS** (ESI) m/z: $[M+H]^+$ Calcd for $C_{27}H_{28}BrN_2O_3^+$: 507.1278; Found: 507.1274.

(±)-*trans*-2-((3-adamantan-1-yl)oxy)-4-(4-methylbenzyl)-2,2a-dihydro-1*H*-azeto[1,2-*a*] quinoxaline-1,3(4*H*)-dione (4o)

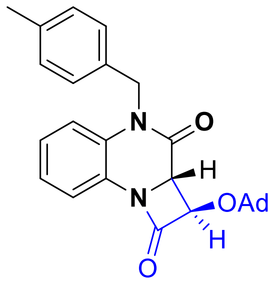

According to ***GP*** with **1p** (50.1 mg, 0.2 mmol, 1.0 equiv.), **2g** (132.2 mg, 0.6 mmol, 3.0 equiv.) in 1.0 mL dry EA for 12 h. Purification by silica gel chromatography afforded the desired **4o** (82.9 mg, 94% yield, dr > 19:1, colorless oil). **^{1}H NMR** (400 MHz, $CDCl_3$, 300 K) δ (ppm) = 7.47 – 7.40 (m, 1H), 7.15 – 7.09 (m, 4H), 7.08 – 7.04 (m, 2H), 6.99 – 6.92 (m, 1H), 5.47 (d, *J* = 16.0 Hz, 1H), 5.37 (d, *J* = 2.0 Hz, 1H), 4.84 (d, *J* = 16.0 Hz, 1H), 4.34 (d, *J* = 1.6 Hz, 1H), 2.32 (s, 3H), 2.22 (s, 3H), 2.02 – 1.88 (m, 6H), 1.69 – 1.62 (s, 6H). **^{13}C NMR** (100 MHz, $CDCl_3$, 300 K) δ (ppm) = 166.4, 164.9, 137.1, 132.6, 132.4, 129.6, 126.2, 126.0, 123.9, 123.8, 120.7, 116.3, 82.0, 76.3, 58.7, 45.7, 41.6, 36.1, 30.6, 21.0. **HRMS** (ESI) m/z: $[M+Na]^+$ Calcd for $C_{28}H_{30}N_2O_3Na^+$: 465.2149; Found: 465.2142.

(±)-*trans*-2-((3-adamantan-1-yl)oxy)-4-(4-nitrobenzyl)-2,2a-dihydro-1H-azeto[1,2-a] quinoxaline-1,3(4H)-dione (4p)

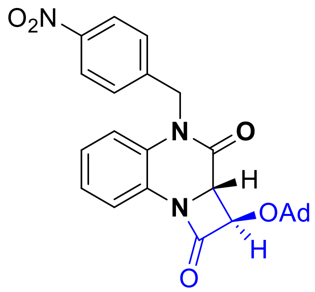

According to ***GP*** with **1q** (56.3 mg, 0.2 mmol, 1.0 equiv.), **2g** (132.2 mg, 0.6 mmol, 3.0 equiv.) in 1.0 mL dry EA for 12 h. Purification by silica gel chromatography afforded the desired **4p** (12.2 mg, 13% yield, dr > 19:1, white solid). **^{1}H NMR** (400 MHz, $CDCl_3$, 300 K) δ (ppm) = 8.21 (d, *J* = 8.8 Hz, 2H), 7.49 (dd, *J* = 7.2, 2.0 Hz,1H), 7.39 (d, *J* = 8.8 Hz, 2H), 7.15 – 7.05 (m, 2H), 6.79 (dd, *J* = 7.2, 2.0 Hz,1H), 5.58 (d, *J* = 16.8 Hz, 1H), 5.39 (d, *J* = 1.6 Hz, 1H), 4.99 (d, *J* = 16.8 Hz, 1H), 4.38 (d, *J* = 1.6 Hz, 1H), 2.23 (s, 3H), 2.00 – 1.87 (m, 6H), 1.69 – 1.63 (m, 6H). **^{13}C NMR** (100 MHz, $CDCl_3$, 300 K) δ (ppm) = 166.3, 165.1, 147.5, 143.2, 131.9, 127.2, 126.2, 124.5, 124.3, 124.0, 121.2, 115.7, 82.2, 76.5, 58.8, 45.6, 41.7, 36.1, 30.7. HRMS (ESI) m/z: $[M+Na]^+$ Calcd for $C_{27}H_{28}N_3O_5Na^+$: 474.2023; Found: 474.2030.

(±)-*trans*-2-((3-adamantan-1-yl)oxy)-4-(3,5-dimethoxybenzyl)-2,2a-dihydro-1H-azeto[1,2-*a*]

quinoxaline-1,3(4*H*)-dione (4q)

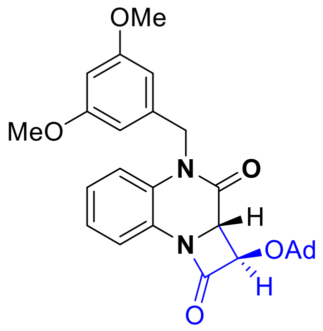

According to ***GP*** with **1r** (59.3 mg, 0.2 mmol, 1.0 equiv.), **2g** (132.2 mg, 0.6 mmol, 3.0 equiv.) in 1.0 mL dry EA for 12 h. Purification by silica gel chromatography afforded the desired **4q** (93.2 mg, 95% yield, dr > 19:1, colorless oil). **^{1}H NMR** (400 MHz, $CDCl_3$, 300 K) δ (ppm) = 7.46 – 7.41 (m, 1H), 7.10 – 7.05 (m, 2H), 6.96 – 6.91 (m, 1H), 6.34 (s, 3H), 5.52 (d, *J* = 16.4 Hz, 1H), 5.36 (d, *J* = 2.0 Hz, 1H), 4.71 (d, *J* = 16.4 Hz, 1H), 4.34 (d, *J* = 1.6 Hz, 1H), 3.76 (s, 6H), 2.22 (s, 3H), 2.01 – 1.87(m, 6H), 1.65 (s, 6H). **^{13}C NMR** (100 MHz, $CDCl_3$, 300 K) δ (ppm) = 166.3, 164.9, 161.3, 138.2, 132.5, 126.2, 124.0, 123.9, 120.8, 116.3, 104.4, 98.7, 82.0, 76.4, 58.7, 55.3, 46.1, 41.7, 36.1, 30.7. **HRMS** (ESI) m/z: $[M+H]^+$ Calcd for $C_{29}H_{33}N_2O_5^+$: 489.2384; Found: 489.2384.

(±)-*trans*-2-((3-adamantan-1-yl)oxy)-6-fluoro-4-methyl-2,2a-dihydro-1*H*-azeto[1,2-*a*] quinoxaline-1,3(4*H*)-dione (4r)

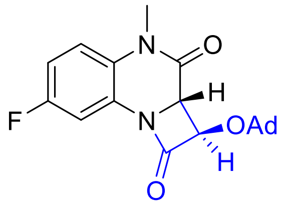

According to ***GP*** with **1s** (35.6 mg, 0.2 mmol, 1.0 equiv.), **2g** (132.2 mg, 0.6 mmol, 3.0 equiv.) in 1.0 mL dry EA for 12 h. Purification by silica gel chromatography afforded the desired **4r** (47.6 mg, 64% yield, dr > 19:1, colorless oil). **^{1}H NMR** (400 MHz, $CDCl_3$, 300 K): δ (ppm) = 7.17 (dd, *J* = 8.0, 2.8 Hz, 1H), 6.97 (dd, *J* = 8.8, 4.8 Hz, 1H), 6.91 (td, *J* = 7.6, 2.8 Hz, 1H), 5.30 (d, *J* = 2.0 Hz, 1H), 4.22 (d, *J* = 2.0 Hz, 1H), 3.39 (s, 3H), 2.22 (s, 3H), 2.00 – 1.84 (m, 6H), 1.65 (s, 6H). **^{13}C NMR** (100 MHz, $CDCl_3$, 300 K): δ (ppm) = 166.5, 164.1, 158.6 (d, *J* = 246.3 Hz), 129.3, 124.5 (d, *J* = 10.6 Hz), 116.4 (d, *J* = 9.2 Hz), 112.5 (d, *J* = 22.8 Hz), 108.4 (d, *J* = 26.1 Hz), 82.1, 76.4, 58.5, 41.6, 36.1, 30.7, 29.2. **HRMS** (ESI) m/z: $[M+H]^+$ Calcd for $C_{21}H_{24}FN_2O_3^+$: 371.1765; Found: 371.1761.

(±)-*trans*-2-((3-adamantan-1-yl)oxy)-6-chloro-4-methyl-2,2a-dihydro-1*H*-azeto[1,2-*a*] quinoxaline-1,3(4*H*)-dione (4s)

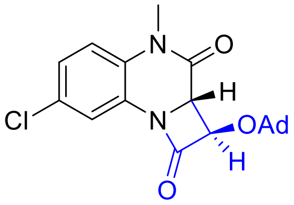

According to ***GP*** with **1t** (38.9 mg, 0.2 mmol, 1.0 equiv.), **2g** (132.2 mg, 0.6 mmol, 3.0 equiv.) in 1.0 mL dry EA for 12 h. Purification by silica gel chromatography afforded the desired **4s** (70.8 mg, 92% yield, dr > 19:1, colorless oil). **^{1}H NMR** (400 MHz, $CDCl_3$, 300 K) δ (ppm) = 7.42 (d, *J* = 2.4 Hz, 1H), 7.17 (dd, *J* = 8.8, 2.4 Hz, 1H), 6.94 (d, *J* = 8.8 Hz, 1H), 5.30 (d, *J* = 2.0 Hz, 1H), 4.21 (d, *J* = 2.0 Hz, 1H), 3.38 (s, 3H), 2.22 (s, 3H), 1.98 – 1.84 (m, 6H), 1.69 – 1.60 (s, 6H). **^{13}C NMR** (100 MHz, $CDCl_3$, 300 K) δ (ppm) = 166.5, 164.3, 131.6, 129.1, 125.9, 124.5, 120.7, 116.4, 82.1, 76.5, 58.5, 41.6, 36.1, 30.7, 29.1. **HRMS** (ESI) m/z: $[M+H]^+$ Calcd for $C_{21}H_{24}ClN_2O_3^+$: 387.1470; Found: 387.1470.

(±)-*trans*-2-((3-adamantan-1-yl)oxy)-6-bromo-4-methyl-2,2a-dihydro-1*H*-azeto[1,2-*a*] quinoxaline-1,3(4*H*)-dione (4t)

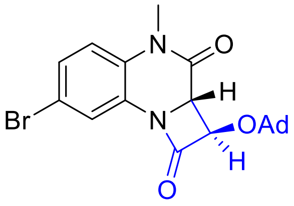

According to ***GP*** with **1u** (47.8 mg, 0.2 mmol, 1.0 equiv.), **2g** (132.2 mg, 0.6 mmol, 3.0 equiv.) in 1.0 mL dry EA for 12 h. Purification by silica gel chromatography afforded the desired **4t** (63.4 mg, 74% yield, dr > 19:1, colorless oil). **^{1}H NMR** (400 MHz, $CDCl_3$, 300 K) δ (ppm) = 7.57 (d, *J* = 2.0 Hz, 1H), 7.32 (dd, *J* = 8.8, 2.0 Hz, 1H), 6.89 (d, *J* = 8.8 Hz, 1H), 5.30 (d, *J* = 2.0 Hz, 1H), 4.21 (d, *J* = 1.6 Hz, 1H), 3.38 (s, 3H), 2.22 (s, 3H), 1.99 – 1.84 (m, 6H), 1.70 – 1.61 (m, 6H). **^{13}C NMR** (100 MHz, $CDCl_3$, 300 K) δ (ppm) = 166.5, 164.3, 132.1, 128.9, 124.7, 123.5, 116.7, 116.3, 82.1, 76.5, 58.5, 41.7, 36.1, 30.7, 29.1. **HRMS** (ESI) m/z: $[M+H]^+$ Calcd for $C_{21}H_{24}BrN_2O_3^+$: 431.0965; Found: 431.0966.

(±)-*trans*-2-((3-adamantan-1-yl)oxy)-4,6,7-trimethyl-2,2a-dihydro-1*H*-azeto[1,2-*a*] quinoxaline-1,3(4*H*)-dione (4u)

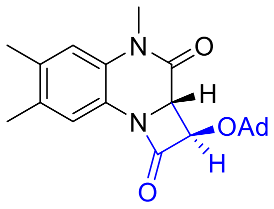

According to ***GP*** with **1v** (37.6 mg, 0.2 mmol, 1.0 equiv.), **2g** (132.2 mg, 0.6 mmol, 3.0 equiv.) in 1.0 mL dry EA for 12 h. Purification by silica gel chromatography afforded the desired **4u** (57.8 mg, 76% yield, dr > 19:1, yellow oil). **^{1}H NMR** (400 MHz, $CDCl_3$, 300 K): δ (ppm) = 7.20 (s, 1H), 6.79 (s, 1H), 5.27 (d, *J* = 2.0 Hz, 1H), 4.17 (d, *J* = 2.0 Hz, 1H), 3.37 (s, 3H), 2.27 (s, 3H), 2.25 – 2.19 (m, 6H), 2.01 – 1.84 (m, 6H), 1.68 – 1.63 (s, 6H). **^{13}C NMR** (100 MHz, $CDCl_3$, 300 K): δ (ppm) = 166.5, 164.8, 134.6, 132.3, 130.7, 121.7, 121.5, 116.6, 81.7, 76.2, 58.8, 41.7, 36.1, 30.7, 28.9, 20.0, 19.0. **HRMS** (ESI) m/z: $[M+H]^+$ Calcd for $C_{23}H_{29}N_2O_3^+$: 381.2173; Found: 381.2166

(±)-*trans*-ethyl 6-cyano-5-phenyl-2,5-dihydropyridazine-3-carboxylate (4v)

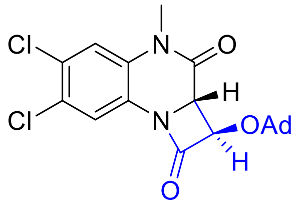

According to ***GP*** with **1w** (45.8 mg, 0.2 mmol, 1.0 equiv.), **2g** (132.2 mg, 0.6 mmol, 3.0 equiv.) in 1.0 mL dry EA for 12 h. Purification by silica gel chromatography afforded the desired **4v** (54.1 mg, 64% yield, dr > 19:1, yellow oil). **^{1}H NMR** (400 MHz, $CDCl_3$, 300 K): δ (ppm) = 7.50 (s, 1H), 7.10 (s, 1H), 5.30 (d, *J* = 2.0 Hz, 1H), 4.22 (d, *J* = 1.6 Hz, 1H), 3.37 (s, 3H), 2.22 (s, 3H), 1.98 – 1.83 (m, 6H), 1.70 – 1.62 (s, 6H). **^{13}C NMR** (100 MHz, $CDCl_3$, 300 K): δ (ppm) = 166.4, 164.1, 132.5, 129.6, 127.2, 122.9, 121.9, 117.0, 82.3, 76.6, 58.5, 41.6, 36.0, 30.7, 29.2. **HRMS** (ESI) m/z: $[M+H]^+$ Calcd for $C_{21}H_{23}Cl_2N_2O_3^+$: 421.1080; Found: 421.1072.

(±)-*trans*-7-((3-adamantan-1-yl)oxy)-6-phenyl-5-thia-1-azabicyclo[4.2.0]octan-8-one (6a)

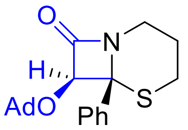

According to ***GP*** with **5a** (35.5 mg, 0.2 mmol, 1.0 equiv.), **2g** (132.2 mg, 0.6 mmol, 3.0 equiv.) in 1.0 mL dry EA for 12 h. Purification by silica gel chromatography afforded the desired **6a** (60.3 mg, 82% yield, dr > 19:1, white solid). **^{1}H NMR** (400 MHz, $CDCl_3$, 300 K): δ (ppm) = 7.63 – 7.59 (m, 2H), 7.41 – 7.36 (m, 2H), 7.34 – 7.29 (m, 1H), 5.05 (s, 1H), 4.07 – 4.01 (m, 1H), 2.93 – 2.84 (m, 1H), 2.70 (dd, *J* = 7.6, 4.0 Hz, 2H), 2.01 (s, 3H), 1.89 – 1.73 (m, 2H), 1.55 – 1.33 (m, 12H). **^{13}C NMR** (100 MHz, $CDCl_3$, 300 K): δ (ppm) = 165.6, 135.8, 129.2, 128.0, 127.9, 87.2, 74.7, 71.3, 41.5, 37.3, 35.9, 30.4, 26.1, 23.9. **HRMS** (ESI) m/z: $[M+H]^+$ Calcd for $C_{22}H_{28}NO_2S^+$: 370.1835; Found: 370.1834.

(±)-*trans*-1-((3-adamantan-1-yl)oxy)-1,4,5,9b-tetrahydro-2*H*-azeto[2,1-*a*]isoquinolin-2-one (6b)

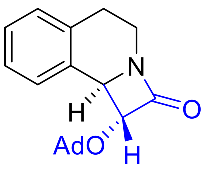

According to ***GP*** with **5b** (26.2 mg, 0.2 mmol, 1.0 equiv.), **2g** (132.2 mg, 0.6 mmol, 3.0 equiv.) in 1.0 mL dry EA for 12 h. Purification by silica gel chromatography afforded the desired **6b** (66.7 mg, 90% yield, dr > 19:1, pale yellow oil). **^{1}H NMR** (600 MHz, $CDCl_3$, 300 K): δ (ppm) = 7.31 – 7.24 (m, 3H), 7.17 (d, *J* = 7.5 Hz, 1H), 4.69 (s, 1H), 4.54 (s, 1H), 4.03 – 3.98 (m, 1H), 3.17 – 3.11 (m, 1H), 3.05 – 2.98 (m, 1H), 2.78 – 2.72 (m, 1H), 2.22 (s, 3H), 1.92 – 1.83 (m, 6H), 1.71 – 1.61 (m, 6H). **^{13}C NMR** (150 MHz, $CDCl_3$, 300 K): δ (ppm) = 168.9, 134.0, 133.9, 129.6, 127.3, 126.9, 125.6, 81.4, 74.5, 67.9, 59.0, 45.2, 37.1, 36.1, 36.0, 30.6, 30.5, 28.3. **HRMS** (ESI) m/z: $[M+H]^+$ Calcd for $C_{21}H_{26}NO_2^+$: 324.1958; Found: 324.1962.

(±)-*trans*-1-((3-adamantan-1-yl)oxy)-1,11b-dihydro-2*H*-azeto[1,2-*f*]phenanthridin-2-one (6c)

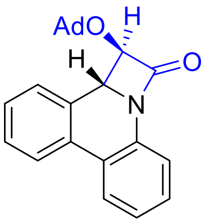

According to ***GP*** with **5c** (35.8 mg, 0.2 mmol, 1.0 equiv.), **2g** (132.2 mg, 0.6 mmol, 3.0 equiv.) in 1.0 mL dry EA for 12 h. Purification by silica gel chromatography afforded the desired **6c** (52.5 mg, 71% yield, dr > 19:1, yellow oil). **^{1}H NMR** (400 MHz, $CDCl_3$, 300 K): δ (ppm) = 7.84 (d, *J* = 8.8 Hz, 2H), 7.49 (dd, *J* = 8.0, 1.2 Hz, 1H), 7.39 – 7.29 (m, 4H), 7.21 (td, *J* = 8.0, 1.2 Hz, 1H), 5.15 (d, *J* = 2.0 Hz, 1H), 4.94 (d, *J* = 2.0 Hz, 1H), 2.25 (s, 3H), 2.04 – 1.87 (m, 6H), 1.73 – 1.63 (s, 6H). **^{13}C NMR** (100 MHz, $CDCl_3$, 300 K): δ (ppm) = 168.5, 132.6, 132.4, 130.2, 129.0, 128.6, 128.2, 125.2, 125.1, 124.9, 123.8, 123.5, 120.7, 83.7, 75.2, 58.4, 42.2, 36.1, 30.7 **HRMS** (ESI) m/z: $[M+H]^+$ Calcd for $C_{25}H_{26}NO_2^+$: 372.1958; Found: 372.1952.

(±)-*trans*-1-((3-adamantan-1-yl)oxy)-1,12b-dihydro-2*H*-azeto[1,2-*d*]dibenzo[*b,f*][1,4] oxazepin-2-one (6d)

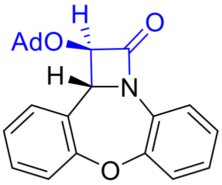

According to ***GP*** with **5d** (39.0 mg, 0.2 mmol, 1.0 equiv.), **2g** (132.2 mg, 0.6 mmol, 3.0 equiv.) in 1.0 mL dry EA for 12 h. Purification by silica gel chromatography afforded the desired **6d** (74.5 mg, 96% yield, dr > 19:1, pale yellow oil). **^{1}H NMR** (400 MHz, $CDCl_3$, 300 K): δ (ppm) = 8.06 (dd, *J* = 8.0, 1.6 Hz, 1H), 7.37 – 7.31 (m, 2H), 7.25 – 7.19 (m, 3H), 7.07 (td, *J* = 7.6, 1.6 Hz, 1H), 7.00 (td, *J* = 7.6, 1.6 Hz, 1H), 5.53 (d, *J* = 2.4 Hz, 1H), 5.30 (d, *J* = 2.4 Hz, 1H), 2.25 (s, 3H), 2.01 – 1.89 (m, 6H), 1.74 – 1.62 (m, 6H). **^{13}C NMR** (100 MHz, $CDCl_3$, 300 K): δ (ppm) = 164.6, 158.7, 144.2, 130.2, 129.9, 129.7, 125.7, 125.2, 125.2, 124.5, 121.8, 121.5, 120.2, 77.0, 75.1, 61.8, 42.2, 36.1, 30.6. **HRMS** (ESI) m/z: $[M+H]^+$ Calcd for $C_{25}H_{26}NO_3^+$: 388.1907; Found: 388.1907.

(±)-*trans*-1-((3-adamantan-1-yl)oxy)-1,12b-dihydro-2*H*-azeto[1,2-*d*]dibenzo[*b,f*][1,4] thiazepine -2-one (6e)

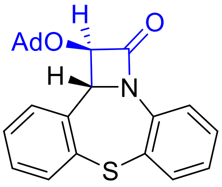

According to ***GP*** with **5e** (42.3 mg, 0.2 mmol, 1.0 equiv.), **2g** (132.2 mg, 0.6 mmol, 3.0 equiv.) in 1.0 mL dry EA for 12 h. Purification by silica gel chromatography afforded the desired **6e** in (74.2 mg, 92% yield, dr > 19:1, colorless oil). **^{1}H NMR** (600 MHz, $CDCl_3$, 300 K): δ (ppm) = 8.18 (d, *J* = 7.8 Hz, 1H), 7.63 (d, *J* = 7.8 Hz, 1H), 7.45 – 7.42 (m, 2H), 7.37 (d, *J* = 7.8 Hz, 1H), 7.35 – 7.32 (m, 1H), 7.18 (t, *J* = 7.8 Hz, 1H), 6.92 (t, *J* = 7.8 Hz, 1H), 6.10 (d, *J* = 1.8 Hz, 1H), 5.32 (d, *J* = 2.4 Hz, 1H), 2.23 (s, 3H), 1.97 – 1.89 (m, 6H), 1.72 – 1.62 (m, 6H). **^{13}C NMR** (150 MHz, $CDCl_3$, 300 K): δ (ppm) = 165.7, 139.0, 138.5, 137.6, 133.5, 132.5, 129.5, 129.4, 129.2, 126.1, 124.0, 120.6, 119.1, 75.0, 74.7, 65.3, 42.2, 36.2, 30.6. **HRMS** (ESI) m/z: $[M+H]^+$ Calcd for $C_{25}H_{26}NO_2S^+$: 404.1679; Found: 404.1679.

(±)-*trans*-1-((3-adamantan-1-yl)oxy)-1,15b-dihydro-2H-azeto[1,2-a]tribenzo[c,e,g]azocin -2-one (6f)

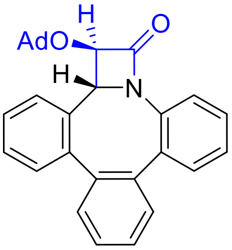

According to ***GP*** with **5f** (25.5 mg, 0.1 mmol, 1.0 equiv.), **2g** (66.1 mg, 0.3 mmol, 3.0 equiv.) in 0.5 mL dry EA for 12 h. Purification by silica gel chromatography afforded the desired **6f** (25.5 mg, 57% yield, dr > 19:1, colorless oil). **^{1}H NMR** (600 MHz, CD_3CN, 300 K): δ (ppm) = 7.54 – 7.47 (m, 2H), 7.42 – 7.32 (m, 5H), 7.31 – 7.25 (m, 4H), 7.01 (d, *J* = 5.4 Hz, 1H), 4.75 (d, *J* = 2.4 Hz, 1H), 4.61 (d, *J* = 2.4 Hz, 1H), 2.06 (s, 3H), 1.69 – 1.56 (m, 6H), 1.55 – 1.47 (m, 6H). **^{13}C NMR** (150 MHz, CD_3CN, 300 K): δ (ppm) = 167.9, 143.4, 142.0, 141.0, 138.3, 135.0, 134.1, 134.0 132.2, 131.3, 131.3, 130.6, 130.0, 129.6, 129.5, 129.4, 129.0, 128.8, 81.2, 75.2, 72.4, 42.7, 36.7, 31.5. **HRMS** (ESI) m/z: $[M+H]^+$ Calcd for $C_{31}H_{30}NO_2^+$: 448.2271; Found: 448.2271.

(±)-3-((3-adamantan-1-yl)oxy)-1-phenylspiro[azetidine-2,9'-fluoren]-4-one (6g)

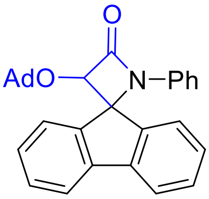

According to ***GP*** with **5g** (51.1 mg, 0.2 mmol, 1.0 equiv.), **2g** (132.2 mg, 0.6 mmol, 3.0 equiv.) in 1.0 mL dry EA for 12 h. Purification by silica gel chromatography afforded the desired **6g** (40.3 mg, 45% yield, yellow solid). **^{1}H NMR** (400 MHz, $CDCl_3$, 300 K): δ (ppm) = 7.78 (t, *J* = 6.6 Hz, 2H), 7.61 (d, *J* = 7.2 Hz, 1H), 7.50 – 7.41 (m, 3H), 7.31 (t, *J* = 7.2 Hz, 2H), 7.05 (t, *J* = 7.8 Hz, 2H), 6.99 (d, *J* = 8.4 Hz, 2H), 6.91 (t, *J* = 7.2 Hz, 1H), 5.29 (s, 1H), 1.88 (s, 3H), 1.47 – 1.38 (m, 3H), 1.34 – 1.27 (m, 3H), 1.26 – 1.17 (m, 6H). **^{13}C NMR** (100 MHz, $CDCl_3$, 300 K): δ (ppm) = 166.0, 141.8, 140.7, 140.1, 139.8, 136.8, 129.4, 129.4, 128.8, 128.1, 127.3, 127.2, 124.0, 122.7, 120.5, 120.0, 117.4, 83.0, 74.8, 73.8, 41.1, 35.8, 30.3. **HRMS** (ESI) m/z: $[M+H]^+$ Calcd for $C_{31}H_{30}NO_2^+$: 448.2271; Found: 448.2271.

(±)-3-((3-adamantan-1-yl)oxy)-1,4,4-triphenylazetidin-2-one (6h)

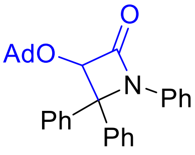

According to ***GP*** with **5h** (51.5 mg, 0.2 mmol, 1.0 equiv.), **2g** (132.2 mg, 0.6 mmol, 3.0 equiv.) in 1.0 mL dry EA for 12 h. Purification by silica gel chromatography afforded the desired **6h** (83.5 mg, 93% yield, yellow oil). **^{1}H NMR** (400 MHz, $CDCl_3$, 300 K) δ (ppm) = 7.47 – 7.40 (m, 4H), 7.40 – 7.30 (m, 8H), 7.16 (t, *J* = 8.0 Hz, 2H), 7.00 (t, *J* = 7.6 Hz, 1H), 5.16 (s, 1H), 2.11

(s, 3H), 1.74 – 1.64 (m, 6H), 1.63 – 1.50 (m, 6H). **^{13}C NMR** (100 MHz, $CDCl_3$, 300 K) δ (ppm) = 167.7, 139.7, 137.8, 135.6, 130.0, 128.7, 128.6, 127.9, 127.6, 127.4, 127.4, 123.8, 118.5, 85.5, 75.0, 73.9, 42.1, 36.1, 30.6. **HRMS** (ESI) m/z: $[M+H]^+$ Calcd for $C_{31}H_{32}NO_2^+$: 450.2428; Found: 450.2428.

(±)-3-((3-adamantan-1-yl)oxy)-1,4-diphenylazetidin-2-one (6i)
According to ***GP*** with **5i** (36.2 mg, 0.2 mmol, 1.0 equiv.), **2g** (132.2 mg, 0.6 mmol, 3.0 equiv.) in 1.0 mL dry EA for 12 h. Purification by silica gel chromatography afforded the desired **6i** (52.0 mg, 70% yield, dr = 2:1, white soild) (the dr value was determined by crude ^{1}H NMR, and coupling constant of *cis* isomer is bigger than that of *trans* isomer).

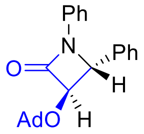

***trans*-6i**

^{1}H NMR (400 MHz, $CDCl_3$, 300 K, major) δ (ppm) = 7.42 – 7.30 (m, 5H), 7.29 – 7.25 (m, 2H), 7.24 – 7.18 (m, 2H), 7.06 – 7.00 (m, 1H), 4.78 (d, *J* = 1.6 Hz, 1H), 4.70 (d, *J* = 1.6 Hz, 1H), 2.14 (s, 3H), 1.81 – 1.65 (m, 6H), 1.62 – 1.54 (m, 6H). **^{13}C NMR** (100 MHz, $CDCl_3$, 300 K, major) δ (ppm) = 166.1, 137.4, 136.4, 129.1, 129.0, 128.5, 126.1, 124.0, 117.5, 83.0, 74.9, 65.8, 42.1 36.0, 30.6.

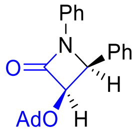

***cis*-6i**

^{1}H NMR (400 MHz, $CDCl_3$, 300 K, minor) δ (ppm) = 7.37 – 7.31 (m, 7H), 7.25 – 7.20 (m, 2H), 7.06 – 7.00 (m, 1H), 5.24 (d, *J* = 5.2 Hz, 1H), 5.10 (d, *J* = 5.2 Hz, 1H), 2.05 (s, 3H), 1.56 (s, 6H), 1.51 – 1.46 (m, 6H). **^{13}C NMR** (100 MHz, $CDCl_3$, 300 K, minor) δ (ppm) = 166.7, 137.4, 134.4, 129.0, 128.7, 128.3, 128.1, 124.0, 117.5, 76.0, 74.7, 63.5, 41.7, 36.1, 30.5. **HRMS** (ESI) m/z: $[M+H]^+$ Calcd for $C_{25}H_{28}NO_2^+$: 374.2115; Found: 374.2116.

(±)-3-((3-adamantan-1-yl)oxy)-4-(naphthalen-1-yl)-1-phenylazetidin-2-one (6j)
According to ***GP*** with **5c** (46.3 mg, 0.2 mmol, 1.0 equiv.), **5j** (132.2 mg, 0.6 mmol, 3.0 equiv.) in 1.0 mL dry EA for 12 h. Purification by silica gel chromatography afforded the desired **6j** (53.0 mg, 63% yield, (***trans*-6j**, colorless oil): (***cis*-6j,** white solid) = 1: 1.3) (dr value was determined by crude ^{1}H NMR, and coupling constant of *cis* isomer is bigger than that of *trans* isomer).

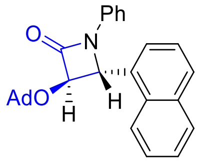

***trans*-6j**

^{1}H NMR (400 MHz, $CDCl_3$, 300 K) δ (ppm) = 8.20 (d, *J* = 8.0 Hz, 1H), 7.88 (d, *J* = 7.6 Hz, 1H), 7.76 (d, *J* = 8.4 Hz, 1H), 7.60 – 7.50 (m, 2H), 7.35 – 7.28 (m, 3H), 7.25 – 7.17 (m, 3H), 7.02 (t, *J* = 7.6 Hz, 1H), 5.61 (d, *J* = 2.0 Hz, 1H), 4.72 (d, *J* = 2.0 Hz, 1H), 2.07 (s, 3H), 1.79 – 1.68 (m, 6H), 1.58 – 1.46 (m, 6H). **^{13}C NMR** (100 MHz, $CDCl_3$, 300 K) δ (ppm) = 166.7, 137.8, 133.9, 132.2, 130.5, 129.0, 128.9, 128.4, 126.5, 126.2, 125.4, 124.0, 123.5, 122.8, 117.6, 82.3, 75.3, 61.5, 42.2, 36.0, 30.6.

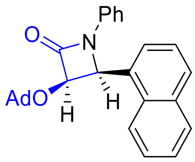

***cis*-6j**

^{1}H NMR (400 MHz, $CDCl_3$, 300 K) δ (ppm) = 7.99 (d, *J* = 8.0 Hz, 1H), 7.92 (d, *J* = 7.6 Hz, 1H), 7.80 (d, *J* = 8.0 Hz, 1H), 7.60 – 7.50 (m, 2H), 7.45 – 7.35 (m, 4H), 7.26 – 7.21 (m, 2H), 7.06 (t, *J* = 7.2 Hz, 1H), 6.00 (d, *J* = 5.6 Hz, 1H), 5.42 (d, *J* = 5.2 Hz, 1H), 1.98 (s, 3H), 1.54 – 1.43 (m, 6H) 1.43 – 1.35 (m, 6H). **^{13}C NMR** (100 MHz, $CDCl_3$, 300 K) δ (ppm) = 167.1, 137.5, 133.6, 131.5, 129.5, 129.1, 129.0, 128.2, 125.9, 125.5, 125.3, 124.1, 122.8, 117.5, 75.9, 74.2, 59.0, 41.6, 36.0, 30.4. **HRMS** (ESI) m/z: $[M+H]^+$ Calcd for $C_{29}H_{30}NO_2^+$: 424.2271; Found: 424.2269.

(±)-3-((3-adamantan-1-yl)oxy)-1-phenyl-4-(pyridin-4-yl)azetidin-2-one (6k)
According to ***GP*** with **5k** (37.5 mg, 0.2 mmol, 1.0 equiv.), **2g** (132.2 mg, 0.6 mmol, 3.0 equiv.) in 1.0 mL dry EA for 12 h. Purification by silica gel chromatography afforded the desired **6k** (46.4 mg, 62% yield, (***trans*-6k**): (***cis*-6k**) = 1.2:1, yellow oil) (the dr value was determined by crude ^{1}H NMR, and coupling constant of *cis* isomer is bigger than that of *trans* isomer).

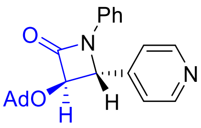

***trans*-6k**

^{1}**H NMR** (400 MHz, $CDCl_3$, 300 K): δ (ppm) = 8.64 (d, *J* = 4.8 Hz, 2H), 7.28 – 7.20 (m, 6H), 7.10 – 7.04 (m, 1H), 4.77 (d, *J* = 2.0 Hz, 1H), 4.69 (d, *J* = 2.0 Hz, 1H), 2.16 (s, 3H), 1.80 – 1.72 (m, 3H), 1.72 – 1.52 (m, 9H). ^{13}**C NMR** (100 MHz, $CDCl_3$, 300 K): δ (ppm) = 165.4, 150.7, 145.6, 136.9, 129.2, 124.5, 120.9, 117.3, 82.8, 75.3, 64.4, 42.0, 36.0, 30.5. **HRMS** (ESI) m/z: $[M+H]^+$ Calcd for $C_{24}H_{27}N_2O_2^+$: 375.2067; Found: 375.2067.

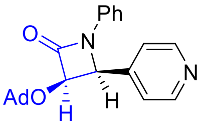

***cis*-6k**

^{1}**H NMR** (400 MHz, $CDCl_3$, 300 K): δ (ppm) = 8.61 (d, *J* = 5.2 Hz, 2H), 7.31 – 7.23 (m, 6H), 7.10 – 7.05 (m, 1H), 5.30 (d, *J* = 5.2 Hz, 1H), 5.10 (d, *J* = 4.8 Hz, 1H), 2.08 (s, 3H), 1.62 – 1.44 (m, 12H). ^{13}**C NMR** (100 MHz, $CDCl_3$, 300 K): δ (ppm) = 166.1, 149.7, 143.9, 137.0, 129.2, 124.5, 123.3, 117.2, 76.0, 75.1, 62.0, 41.8, 36.0, 30.5.

(±)-*trans*-3-((3-adamantan-1-yl)oxy)-4-(furan-2-yl)-1-phenylazetidin-2-one (6l)

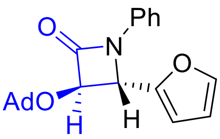

According to ***GP*** with **5l** (34.2 mg, 0.2 mmol, 1.0 equiv.), **2g** (132.2 mg, 0.6 mmol, 3.0 equiv.) in 1.0 mL dry EA for 12 h. Purification by silica gel chromatography afforded the desired **6l** (68.3 mg, 94% yield, dr = 7:1, yellow solid) (the dr value was determined by crude ^{1}H NMR, and coupling constant of *trans* isomer is smaller than that of *cis* isomer). ^{1}**H NMR** (400 MHz, $CDCl_3$, 300 K) δ (ppm) = 7.41 (d, *J* = 1.6 Hz, 1H), 7.35 – 7.31 (m, 2H), 7.27 – 7.21 (m, 2H), 7.07 – 7.02 (m, 1H), 6.45 (d, *J* = 3.2 Hz, 1H), 6.38 (dd, *J* = 3.2, 1.6 Hz, 1H), 5.05 (d, *J* = 2.0 Hz, 1H), 4.81 (d, *J* = 2.0 Hz, 1H), 2.16 (s, 3H), 1.84 – 1.54 (m, 12H). ^{13}**C NMR** (100 MHz, $CDCl_3$, 300 K) δ (ppm) = 165.5, 149.3, 143.3, 137.5, 128.9, 124.2, 117.2, 110.7, 109.5, 80.0, 75.1, 59.1, 41.9, 36.0, 30.5. **HRMS** (ESI) m/z: $[M+H]^+$ Calcd for $C_{23}H_{26}NO_3^+$: 364.1907; Found: 364.1907.

(±)-*trans*-3-((3-adamantan-1-yl)oxy)-1-phenyl-4-(thiophen-2-yl)azetidin-2-one (6m)

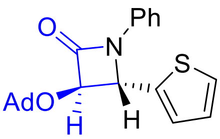

According to ***GP*** with **5m** (37.5 mg, 0.2 mmol, 1.0 equiv.), **2g** (132.2 mg, 0.6 mmol, 3.0 equiv.) in 1.0 mL dry EA for 12 h. Purification by silica gel chromatography afforded the desired ***trans*-6m** (56.6 mg, 75% yield, dr > 19:1 yellow solid, ***cis*-6m** was not observed according to the coupling constant of crude ^{1}H NMR Spectrum). ^{1}**H NMR** (600 MHz, $CDCl_3$, 300 K) δ (ppm) = 7.33 (d, *J* = 8.4 Hz, 2H), 7.30 (d, *J* = 5.4 Hz, 1H), 7.26 – 7.22 (m, 2H), 7.11 (d, *J* = 3.6 Hz, 1H), 7.05 (t, *J* = 7.2 Hz, 1H), 7.01 (t, *J* = 4.2 Hz, 1H), 5.04 (d, *J* = 1.8 Hz, 1H), 4.83 (d, *J* = 1.8 Hz, 1H), 2.16 (s, 3H), 1.83 – 1.69 (m, 6H), 1.66 – 1.54 (m, 6H). ^{13}**C NMR** (150 MHz, $CDCl_3$, 300 K) δ (ppm) = 165.6, 140.1, 137.2, 129.0, 127.3, 125.9, 125.8, 124.3, 117.5, 83.8, 75.1, 61.8, 42.1, 36.0, 30.6. **HRMS** (ESI) m/z: $[M+H]^+$ Calcd for $C_{23}H_{26}NO_2S^+$: 380.1679; Found: 380.1680.

(±)-*trans*-3-((3-adamantan-1-yl)oxy)-4-(4-chlorophenyl)-1-(phenylamino)azetidine-2-one (6n)

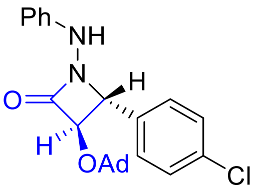

According to ***GP*** with **5n** (42.8 mg, 0.1 mmol, 1.0 equiv.), **2g** (132.2 mg, 0.3 mmol, 3.0 equiv.) in 0.5 mL dry EA for 12 h. Purification by silica gel chromatography afforded the desired **6n** (39.5 mg, 94%, dr > 19:1, colorless oil). ^{1}**H NMR** (400 MHz, $CDCl_3$, 300 K): δ (ppm) = 7.38 (d, *J* = 8.4 Hz, 2H), 7.25 – 7.17 (m, 4H), 6.93 (t, *J* = 7.6 Hz, 1H), 6.79 (d, *J* = 8.0 Hz, 2H), 5.77 (s, 1H), 4.69 (d, *J* = 1.6 Hz, 1H), 4.64 (d, *J* = 1.6 Hz, 1H), 2.13 (s, 3H), 1.80 – 1.71 (m, 3H), 1.71 – 1.51 (m, 9H). ^{13}**C NMR** (100 MHz, $CDCl_3$, 300 K): δ (ppm) = 168.9, 144.4, 134.8, 133.8, 129.5, 129.4, 128.3, 121.7, 114.0, 80.7, 75.1, 68.0, 42.0, 36.0, 30.5. **HRMS** (ESI) m/z: $[M+H]^+$ Calcd for $C_{25}H_{28}ClN_2O_2^+$: 423.1834; Found: 423.1834.

With the optimal reaction conditions, we have evaluated several oxygen or sulfur nucleophiles, including phenol, benzyl alcohol, 4-methylbenzenethiol and benzyl mercaptan. As presented in the following Scheme S1, both phenol and benzyl alcohol proved unreactive under our standard reaction conditions, and the starting materials were fully recovered. Interestingly, 4-methylbenzenethiol led

to the competitive carbene S-H insertion product (**14**) with 36% yield, while benzyl mercaptan yielded the desired nucleophilic addition product (**15**) with 41% yield.

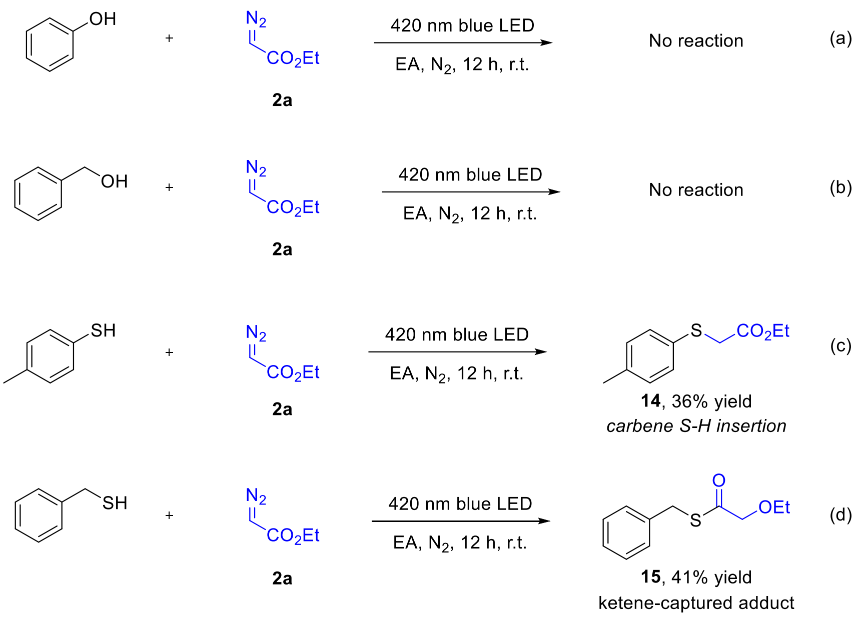

Scheme S1. Investigation of oxygen or sulfur nucleophiles.

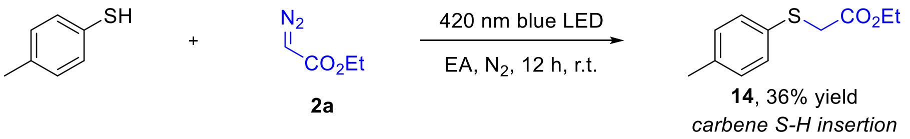

To a 10 mL Schlenk flask equipped with a magnetic stir bar was added **2a** (68.5 mg, 0.6 mmol, 3.0 equiv.), 4-methylbenzenethiol (24.8 mg, 0.2 mmol, 1.0 equiv.) and dry EA (1.0 mL). After the resulting mixture was degassed via 'freeze-pump-thaw' procedure (3 times), the solution was stirred at room temperature and irradiated with blue LED (λ = 420 nm) for 12 h. The solvent was removed by vacuum and the crude product was purified by flash chromatography on silica gel silica: 200~300; eluant: petroleum ether/ethyl acetate (10:1) to provide pure product **14** as yellow oil in 36% yield (15 mg). **^{1}H NMR** (400 MHz, $CDCl_3$, 300 K) δ (ppm) = 7.33 (d, J = 7.6 Hz, 2H), 7.11 (d, J = 7.6 Hz, 2H), 4.15 (q, J = 7.2 Hz, 2H), 3.58 (s, 2H), 2.32 (s, 3H), 1.22 (t, J = 7.2 Hz, 3H). **^{13}C NMR** (100 MHz, $CDCl_3$, 300 K) δ (ppm) = 169.8, 137.3, 131.1, 130.9, 129.8, 61.5, 37.4, 21.1, 14.1. **HRMS** (ESI) m/z: $[M+H]^+$ Calcd for $C_{11}H_{15}O_2S^+$: 211.0787; Found: 211.0787.

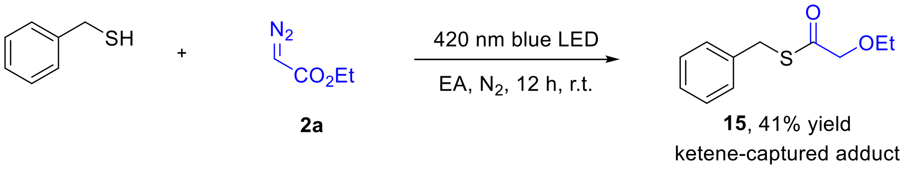

To a 10 mL Schlenk flask equipped with a magnetic stir bar was added **2a** (114.17 mg, 1.0 mmol, 5.0 equiv.), benzyl mercaptan (24.8 mg, 0.2 mmol, 1.0 equiv.) and dry EA (1.0 mL). After the resulting mixture was degassed via 'freeze-pump-thaw' procedure (3 times), the solution was stirred at room temperature and irradiated with blue LED (λ = 420 nm) for 12 h. The solvent was removed by vacuum and the crude product was purified by flash chromatography on silica gel silica: 200~300; eluant: petroleum ether/ethyl acetate (10:1) to provide pure product **15** as colorless oil in 41% yield

(17.2 mg). **^{1}H NMR** (600 MHz, $CDCl_3$, 300 K) δ (ppm) = 7.32 – 7.27 (m, 4H), 7.26 – 7.21 (m, 1H), 4.14 (s, 2H), 4.13 (s, 2H), 3.62 (q, 7.2 Hz, 2H), 1.26 (t, J = 7.2 Hz, 3H). **^{13}C NMR** (150 MHz, $CDCl_3$, 300 K) δ (ppm) = 199.9, 137.4, 128.9, 128.6, 127.3, 75.5, 68.1, 32.2, 15.0. **HRMS** (ESI) m/z: $[M+H]^+$ Calcd for $C_{11}H_{15}O_2S^+$: 211.0787; Found: 211.0787.

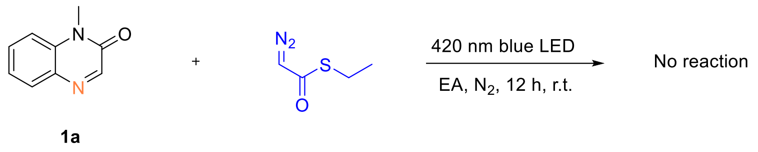

Scheme S2. Investigation of the α-diazo thioester substrate in this [2+2] cycloaddition.

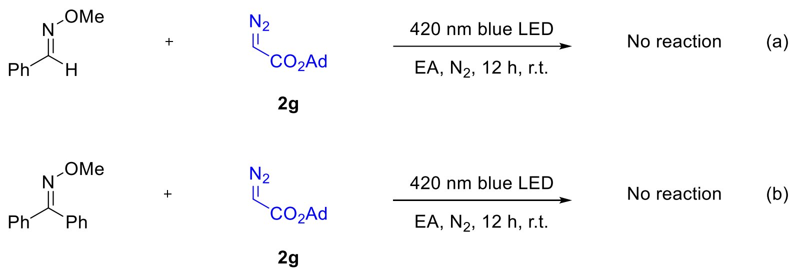

Scheme S3. Investigation of imine substrates in [2+2] cycloadditions.

5. Synthetic applications and gram-scale Synthesis

5.1 Scale-up reaction

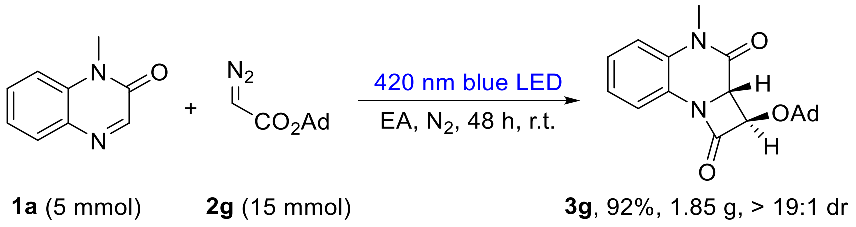

To a 10 mL Schlenk flask equipped with a magnetic stir bar was added **1a** (0.8 g, 5 mmol, 1.0 equiv.), **2a** (3.30 g, 15 mmol, 3.0 equiv.), dry EA (25.0 mL). After the resulting mixture was degassed via 'freeze-pump-thaw' procedure (3 times), the solution was stirred at room temperature and irradiated with blue LED (λ = 420 nm) for 48 h. The solvent was removed by vacuum and the crude product was purified by flash chromatography on silica gel silica: 200~300; eluant: petroleum ether/ethyl acetate (10:1 to 3:1) to provide pure product **3a** as a white solid in 92% yield (1.85 g).

5.2 Reduction of 3g by $LiAlH_4$

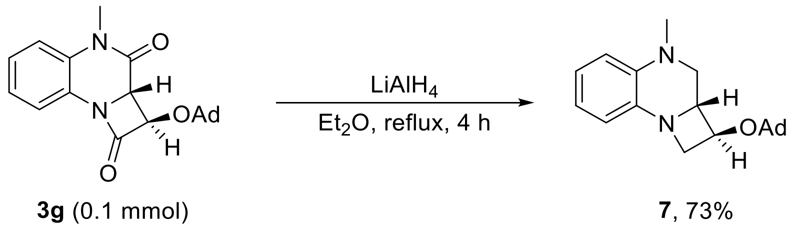

To a 10 mL Schlenk flask equipped with a magnetic stir bar was added **3g** (35.2 mg, 0.1 mmol, 1.0 equiv.), $LiAlH_4$ (15.2 mg, 0.4 mmol, 4.0 equiv.), and Et_2O (1.0 mL). The solution was refluxed for

4 h. The solvent was removed by vacuum and the crude product was purified by flash chromatography on silica gel silica: 200~300; eluant: petroleum ether/ethyl acetate (10:1 to 3:1) to provide pure product **7** as pale yellow oil in 73% yield (23.8 mg). **^{1}H NMR** (400 MHz, $CDCl_3$, 300 K) δ (ppm) = 6.70 – 6.58 (m, 3H), 6.56 – 6.48 (m, 2H), 3.85 – 3.80 (m, 1H), 3.73 – 3.61 (m, 3H), 3.23 (d, *J* = 12.8 Hz, 1H), 3.08 (d, *J* = 11.2 Hz, 1H), 2.85 (s, 3H), 2.17 (s, 3H), 1.84 – 1.73 (m, 6H), 1.69 – 1.56 (m, 6H). **^{13}C NMR** (100 MHz, $CDCl_3$, 300 K) δ (ppm) = 135.5, 133.9, 119.3, 118.0, 113.2, 112.0, 73.8, 70.6, 63.1, 53.2, 50.4, 42.7, 39.1, 36.2, 30.6. **HRMS** (ESI) m/z: $[M+H]^+$ Calcd for $C_{21}H_{29}N_2O^+$: 325.2274; Found: 325.2271.

5.3 Thionation of β-lactam 6g[6]

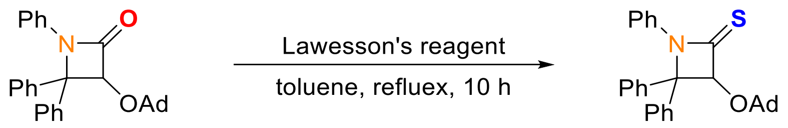

6g (0.1 mmol) **8**, 74%

To a 10 mL Schlenk flask equipped with a magnetic stir bar was added **6g** (44.9 mg, 0.1 mmol, 1.0 equiv.), Lawesson's reagent (15.2 mg, 0.0315 mmol, 0.375 equiv.) and toluene (1.0 mL). The solution was refluxed for 10 h. The solvent was removed by vacuum and the crude product was purified by flash chromatography on silica gel silica: 200~300; eluant: petroleum ether/ethyl acetate (10:1 to 3:1) to provide pure product **8** as yellow oil in 74% yield (34.3 mg). **^{1}H NMR** (600 MHz, $CDCl_3$, 300 K) δ (ppm) = 7.87 (d, *J* = 8.4 Hz, 2H), 7.47 – 7.42 (m, 2H), 7.42 – 7.32 (m, 8H), 7.20 (t, *J* = 6.6 Hz, 2H), 7.11 (t, *J* = 7.2 Hz, 1H), 4.97 (s, 1H), 2.11 (s, 3H), 1.70 (s, 6H), 1.62-1.50 (m, 6H). **^{13}C NMR** (150 MHz, $CDCl_3$, 300 K) δ (ppm) = 201.8, 138.3, 138.3, 135.0, 129.8, 128.7, 128.6, 128.3, 127.9, 127.6, 127.4, 125.9, 120.1, 84.1, 83.7, 75.2, 42.6, 36.1, 30.6. **HRMS** (ESI) m/z: $[M+H]^+$ Calcd for $C_{31}H_{32}NOS^+$: 466.2199; Found: 466.2220.

6. Reactions with amine nucleophiles

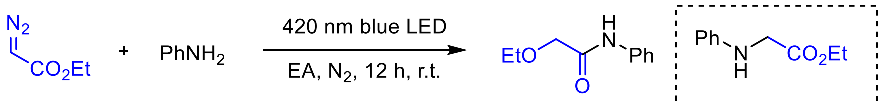

2a

10, 22% (*ketene reactivity*) **10'**, not detected (*carbene reactivity*)

To a 10 mL Schlenk flask equipped with a magnetic stir bar was added **2a** (34.8 mg, 0.3 mmol, 1.0 equiv.), aniline (28.0 mg, 0.3 mmol, 1.0 equiv.) and dry EA (1.0 mL). After the resulting mixture was degassed via 'freeze-pump-thaw' procedure (3 times), the solution was stirred at room temperature and irradiated with blue LED (λ = 420 nm) for 12 h. The solvent was removed by vacuum and the crude product was purified by flash chromatography on silica gel silica: 200~300; eluant: petroleum ether/ethyl acetate (10:1 to 5:1) to provide pure product **10** as yellow oil in 22% yield (11.8 mg). **^{1}H NMR** (600 MHz, $CDCl_3$, 300 K) δ (ppm) = 8.30 (s, 1H), 7.57 (d, *J* = 7.2 Hz, 2H), 7.35 – 7.32 (m, 2H), 7.12 (t, *J* = 7.2 Hz, 1H), 4.05 (s, 2H), 3.66 (q, *J* = 7.2 Hz, 2H), 1.31 (t, *J* = 7.2 Hz, 3H). **^{13}C NMR** (150 MHz, $CDCl_3$, 300 K) δ (ppm) = 167.8, 137.1, 129.0, 124.4, 119.7, 70.0, 67.3, 15.0. **HRMS** (ESI) m/z: $[M+H]^+$ Calcd for $C_{10}H_{14}NO_2^+$: 180.1019; Found: 180.1017.

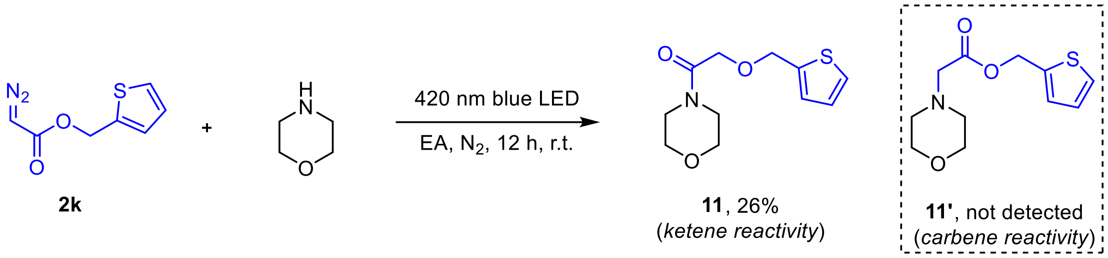

To a 10 mL Schlenk flask equipped with a magnetic stir bar was added **2k** (91.1 mg, 0.5 mmol, 1.0 equiv.), aniline (21.8 mg, 0.25 mmol, 1.0 equiv.) and dry EA (1.0 mL). After the resulting mixture was degassed via 'freeze-pump-thaw' procedure (3 times), the solution was stirred at room temperature and irradiated with blue LED (λ = 420 nm) for 12 h. The solvent was removed by vacuum and the crude product was purified by flash chromatography on silica gel silica: 200~300; eluant: petroleum ether/ethyl acetate (5:1 to 3:1) to provide pure product **11** as yellow oil in 26% yield (15.7 mg). **^{1}H NMR** (600 MHz, $CDCl_3$, 300 K) δ (ppm) = 7.31 (dd, J = 5.4, 1.2 Hz, 1H), 7.03 (dd, J = 3.6, 1.3 Hz, 1H), 6.98 (dd, J = 4.8, 3.6 Hz, 1H), 4.76 (s, 2H), 4.17 (s, 2H), 3.68 (t, J = 4.8 Hz, 2H), 3.65 (t, J = 4.8 Hz, 2H), 3.60 (t, J = 4.8 Hz, 2H), 3.49 (t, J = 4.8 Hz, 2H). **^{13}C NMR** (150 MHz, $CDCl_3$, 300 K) δ (ppm) = 167.6, 139.5, 127.3, 126.8, 126.4, 68.9, 67.4, 66.8, 66.7, 45.7, 42.1. **HRMS** (ESI) m/z: $[M+Na]^+$ Calcd for $C_{11}H_{15}NO_3SNa^+$: 264.0665; Found: 264.0672.

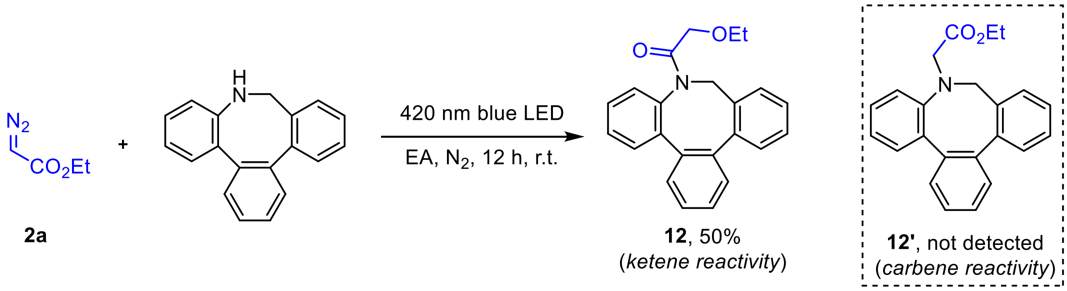

To a 10 mL Schlenk flask equipped with a magnetic stir bar was added **2a** (34.8 mg, 0.3 mmol, 1.0 equiv.), amine (25.7 mg, 0.1 mmol, 1.0 equiv.) and dry EA (1.0 mL). After the resulting mixture was degassed via 'freeze-pump-thaw' procedure (3 times), the solution was stirred at room temperature and irradiated with blue LED (λ = 420 nm) for 12 h. The solvent was removed by vacuum and the crude product was purified by flash chromatography on silica gel silica: 200~300; eluant: petroleum ether/ethyl acetate (10:1 to 5:1) to provide pure product **12** as yellow oil in 50% yield (17.2 mg). **^{1}H NMR** (600 MHz, $CDCl_3$, 300 K) δ (ppm) = δ 7.49 (td, J = 7.8, 1.8 Hz, 1H), 7.45 (td, J = 7.8, 1.8 Hz, 1H), 7.37 (dd, J = 7.8, 1.8 Hz, 1H), 7.32 – 7.26 (m, 3H), 7.23 – 7.19 (m, 2H), 7.18 – 7.12 (m, 3H), 7.00 (d, J = 7.8 Hz, 1H), 5.15 (d, J = 14.4 Hz, 1H), 4.50 (d, J = 14.4 Hz, 1H), 3.77 (d, J = 15.6 Hz, 1H), 3.41 (d, J = 15.0 Hz, 1H), 3.37 (q, J = 7.2 Hz, 2H), 1.15 (t, J = 7.2 Hz, 3H). **^{13}C NMR** (150 MHz, $CDCl_3$, 300 K) δ (ppm) = 168.7, 142.3, 141.9, 140.7, 139.0, 137.5, 133.1, 130.3, 129.9, 129.7, 128.9, 128.84, 128.77, 128.6, 128.2, 128.0, 127.8, 127.6, 127.5, 69.3, 66.9, 52.9, 14.9. **HRMS** (ESI) m/z: $[M+H]^+$ Calcd for $C_{23}H_{22}NO_2^+$: 344.1645; Found: 344.1651.

7. Mechanism studies

7.1 Competition experiments with styrene as carbene trap

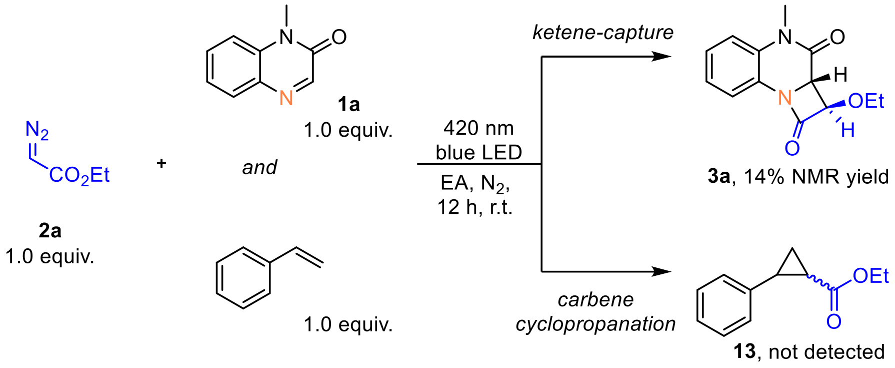

To a 10 mL Schlenk flask equipped with a magnetic stir bar was added **1a** (32.0 mg, 0.2 mmol, 1.0 equiv.), **2a** (22.8 mg, 0.2 mmol, 1.0 equiv.), styrene (23.0 uL, 0.2 mmol, 1.0 equiv.), and dry EA (1.0 mL). After the resulting mixture was degassed via 'freeze-pump-thaw' procedure (3 times), the solution was stirred at room temperature and irradiated with blue LED (λ = 420 nm) for 12 h. The solvent was removed by vacuum and the crude product was analyzed by ^{1}H NMR using 1,3,5-trimethoxybenzene as an internal standard. Competition experiments using styrene as a carbene trap showed a 14% NMR yield for **3a**, and no cyclopropanation product (**13**) was detected.

7.2 Control experiment

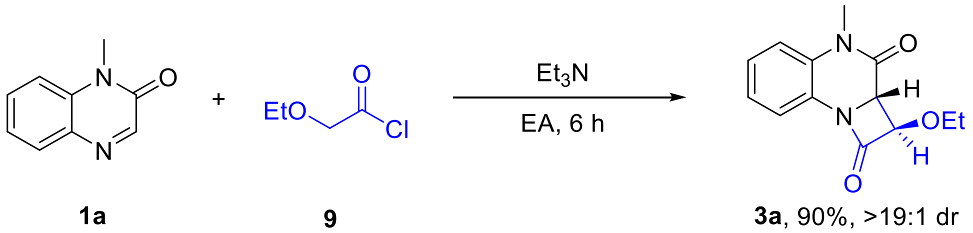

To a 10 mL Schlenk flask equipped with a magnetic stir bar was added **1a** (32.0 mg, 0.2 mmol, 1.0 equiv.), **9** (73.5 mg, 0.6 mmol, 3.0 equiv.), Et_3N (40.5 mg, 0.4 mmol, 2.0 equiv.) and dry EA (1.0 mL). The solution was stirred at room temperature for 6 h. The solvent was removed by vacuum and the crude product was purified by flash chromatography on silica gel silica: 200~300; eluant: petroleum ether/ethyl acetate (5:1 to 3:1) to provide pure product **3a** as a white solid in 90% yield (44.5 mg).

7.3 D-labeling experiment

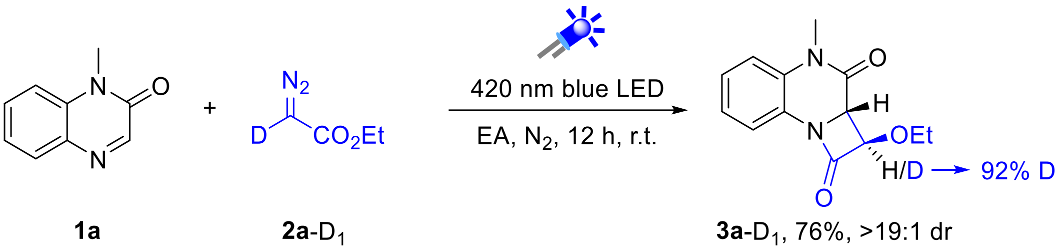

To a 10 mL Schlenk flask equipped with a magnetic stir bar was added **1a** (32.0 mg, 0.2 mmol, 1.0 equiv.), **2a-D$_1$** (69.1 mg, 0.6 mmol, 3.0 equiv.) and dry EA (1.0 mL). After the resulting mixture was degassed via 'freeze-pump-thaw' procedure (3 times), the solution was stirred at room temperature and irradiated with blue LED (λ = 420 nm) for 12 h. The solvent was removed by vacuum and the crude product was purified by flash chromatography on silica gel silica: 200~300; eluant: petroleum

ether/ethyl acetate (5:1 to 3:1) to provide pure product **3a-D$_1$** as a white solid in 76% yield (37.6 mg).

8. DFT calculations

8.1 Computational Details

General calculations were performed using the *Gaussian 16* program.[7] Structure Optimizations were performed using the B3LYP functional,[8] with Grimme's dispersion Correction (denoted B3LYP-GD3)[9] and the def2tzvpp basis set[10] for all atoms. Harmonic vibrational frequencies were calculated at the same level of theory for all stationary points to confirm them as local minima and for all transition-states to confirm that they possess one imaginary frequency. Key transition-state structures were confirmed to be connected to the reactants and the product by intrinsic reaction coordinate (IRC) calculations.[11] For excited states, TD-DFT as implemented in *Gaussian 16* was used.[12] To ensure, that the optimal geometries are used for the calculations, conformer screenings with *ORCA* were performed using the GOAT algorithm at important points.[13] In addition, the solvent effects of ethyl acetate ($\varepsilon = 5.99$) were considered using SMD solvation for all calculations.[14] The given Gibbs free energies in ethyl acetate were calculated in accordance with the equation: $G_{sol} = TCG + E_{sol}$. The enthalpies in ethyl acetate were calculated equivalently using: $H_{sol} = TCH + E_{sol}$. The minimum energy crossing points for changes between multiplicities were calculated based on the *sobMECP* software.[15] To generate images of the 3D structures, the *CYL View* software was used.[16]

8.2 Schemes of Computed Reaction Pathways

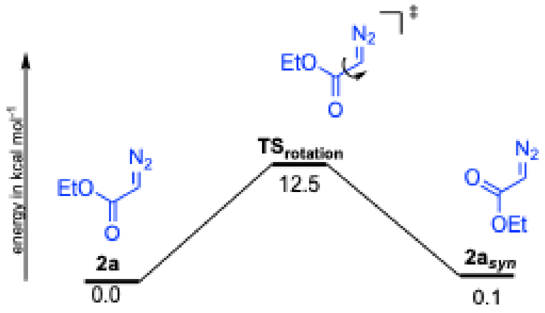

Figure S1: *anti-to-syn* rotation of ethyl diazoacetate **2a**.

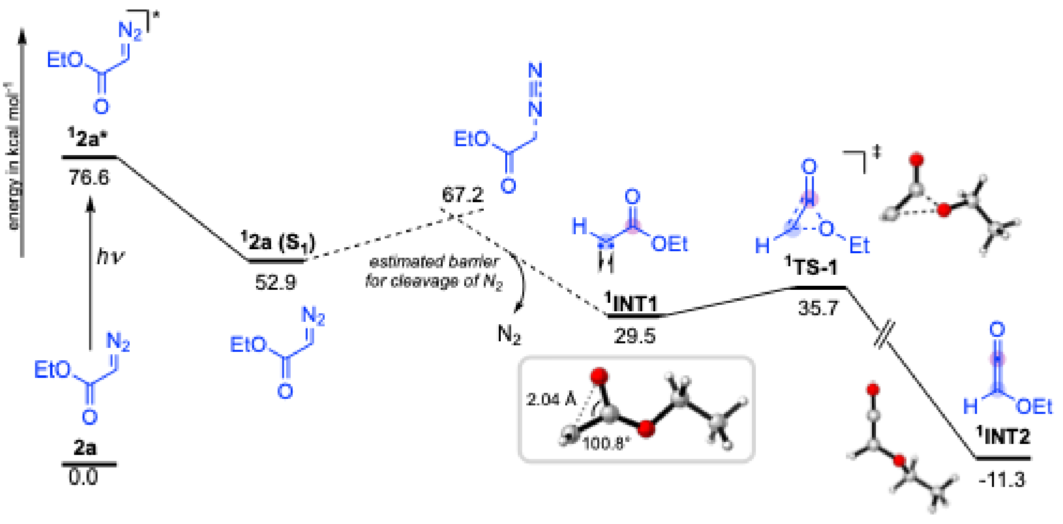

Figure S2: Photoexcitation of the ethyl diazoacetate **2a**, generating singlet carbene **INT1** and subsequent Wolff-rearrangement via **TS1** to form ketene intermediate **INT2**.

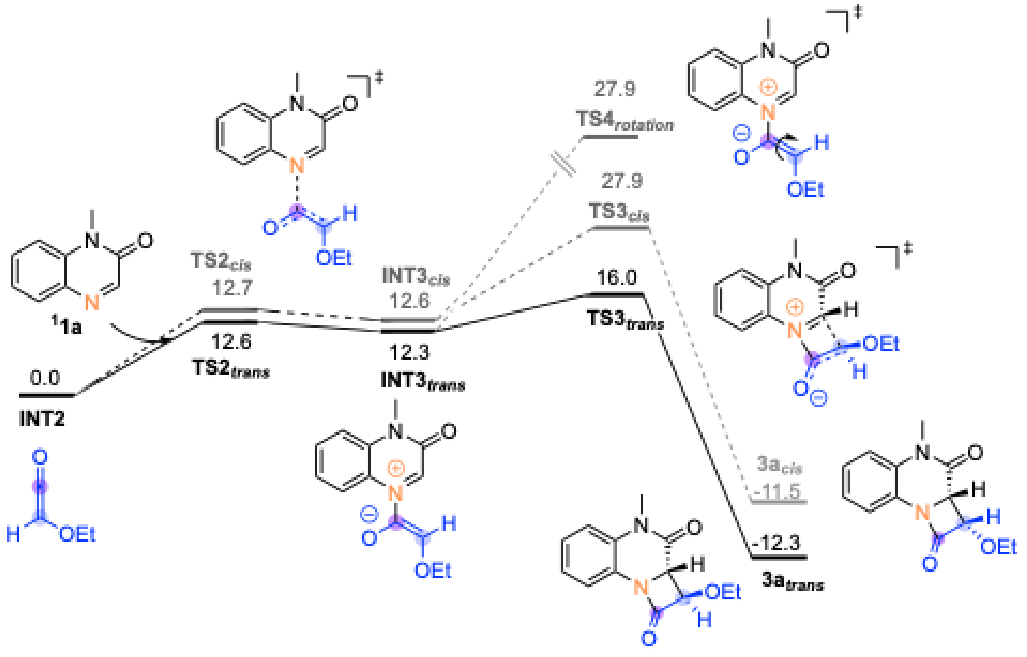

Figure S3: Cycloaddition pathway to form **3a**$_{trans}$ and **3a**$_{cis}$ product originating from ketene intermediate **INT2**.

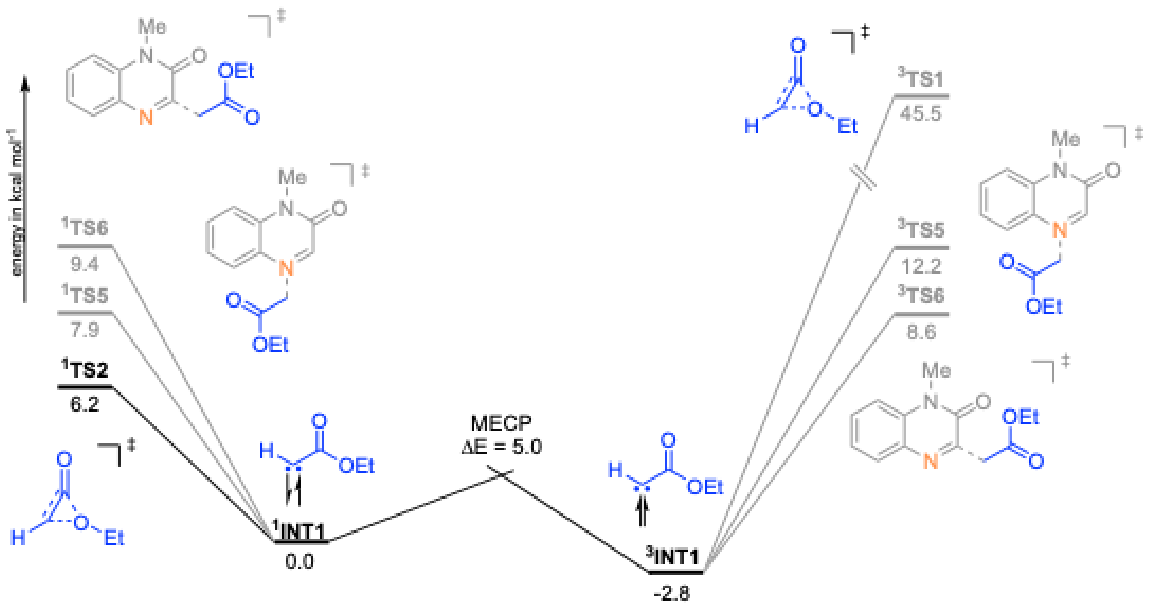

Figure S4: Possible reaction pathways from singlet carbene intermediate ^{1}**INT1**.

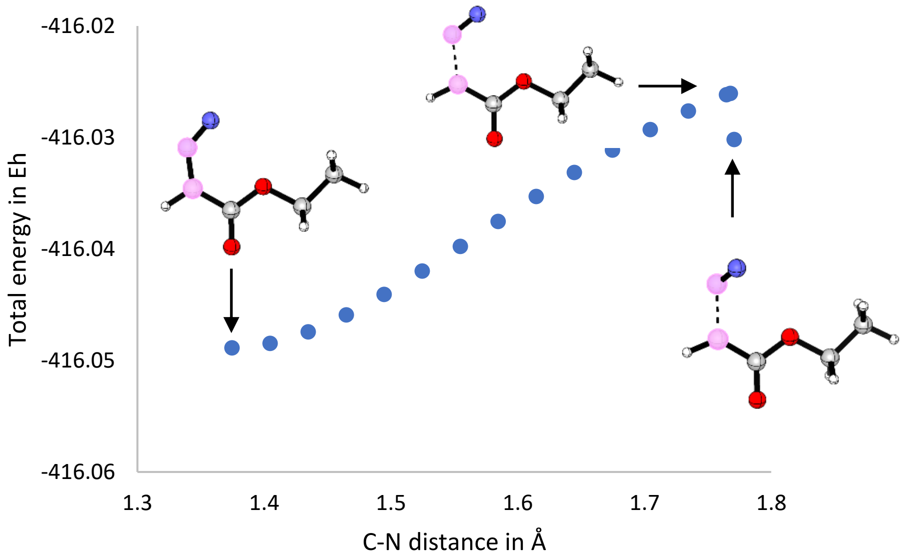

Figure S5: C-N-bond scan for **2a** (**S$_1$**).

8.3 Computed Energies of all stationary points

Table S1. Calculated Energies of all stationary point. Thermal correction to Gibbs free energies (***TCG***, in Hartree) and thermal correction to the enthalpies (***TCH***, in Hartree) at the B3LYP-GD3 / def2tzvpp level of theory in ethyl acetate using the SMD solvation model ($\boldsymbol{E}_{\mathbf{sol}}$, in Hartree) as well as the sum of the single point energy and thermal free energy correction ($\boldsymbol{G}_{\mathbf{sol}}$, in Hartree) or thermal enthalpy correction ($\boldsymbol{H}_{\mathbf{sol}}$, in Hartree). Excited state barriers were estimated using a relaxed scan.

Name	*TCG* /Hartree	*TCH* /Hartree	E_{sol} /Hartree	G_{sol} /Hartree	H_{sol} /Hartree
2a	0.070567	0.113812	-416.1349	-416.0643	-416.0211
$TS_{rotation}$	0.07012	0.112417	-416.1145	-416.0444	-416.0021
$2a_{syn}$	0.070708	0.113855	-416.1350	-416.0643	-416.0211
2a*			-416.0128		
2a (S_1)	0.068741	0.112461	-416.0489	-415.9801	-415.9364
^{1}INT1	0.061054	0.100035	-306.4974	-306.4363	-306.3973
N_2	-0.012828	0.0089	-109.5682	-109.58103	-109.5593
^{1}TS1	0.060044	0.098215	-306.4864	-306.4264	-306.3882
INT2	0.062156	0.101372	-306.5635	-306.5013	-306.4621
1a	0.122313	0.166565	-532.75042	-532.62811	-532.58385
$TS2_{trans}$	0.205531	0.269397	-839.3149	-839.1094	-839.0455
$TS2_{cis}$	0.206331	0.26937	-839.3155	-839.1091	-839.0461
$INT3_{trans}$	0.208449	0.27107	-839.3182	-839.1098	-839.0472
$INT3_{cis}$	0.206758	0.270719	-839.3162	-839.1094	-839.0454
$TS3_{trans}$	0.21066	0.270284	-839.3145	-839.1039	-839.0442
$TS3_{cis}$	0.207984	0.269364	-839.2930	-839.0850	-839.0236
$TS4_{rotation}$	0.208143	0.268385	-839.26626	-839.05811	-838.99787
$3a_{trans}$	0.212209	0.272842	-839.3613	-839.1491	-839.0885
$3a_{cis}$	0.211442	0.272704	-839.3592	-839.1478	-839.0865
^{1}TS5	0.198677	0.267311	-839.2505	-839.0518	-838.9832
^{1}TS6	0.202016	0.267404	-839.2515	-839.0495	-838.9841
MECP			-306.48934		
^{3}INT1	0.06059	0.099921	-306.5014	-306.4408	-306.4015
^{3}TS1	0.050425	0.093788	-306.4142	-306.3638	-306.3204
^{3}TS5	0.20072	0.266949	-839.2457	-839.0450	-838.9788
^{3}TS6	0.201381	0.267088	-839.2521	-839.0507	-838.9850

3D Structure and Coordinates of all Stationary Points2a

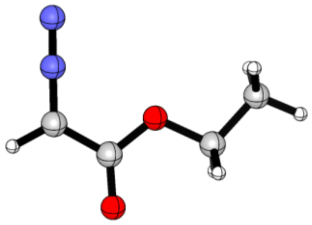

Charge: 0

Spin: 1

C	-1.69015200	0.61613100	-0.00011400
H	-2.46654500	1.36290700	-0.00096200
C	-0.27942800	0.95858800	0.00008300
N	-2.06408600	-0.63236200	-0.00006300
N	-2.37188200	-1.71287000	-0.00024300
O	0.12495600	2.10278800	-0.00090200
O	0.51343500	-0.12955500	0.00191300
C	1.94478700	0.10522900	0.00079400
H	2.20203600	0.69143400	-0.88210000
H	2.20368500	0.68944300	0.88452600
C	2.62393700	-1.24245300	-0.00117700
H	2.35187500	-1.81655700	-0.88788600
H	3.70616100	-1.10272400	-0.00139000
H	2.35257200	-1.81871600	0.88435900

$TS_{rotation}$

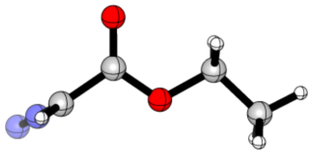

Charge: 0

Spin: 1

C	-1.49389900	0.17111100	0.76337000
H	-1.74799700	0.36677100	1.79321400
C	-0.22061200	0.67129600	0.14482200
N	-2.33952400	-0.46776000	0.03615800
N	-3.07624200	-1.02919800	-0.61752900
O	-0.10303700	1.75192800	-0.37799300
O	0.76487600	-0.21664800	0.27581700
C	2.06996900	0.16520800	-0.25377500
H	1.95922700	0.37414400	-1.31793000
H	2.38608600	1.08177200	0.24459200
C	3.01622900	-0.97979900	0.00627600
H	2.67697200	-1.88896000	-0.49135200
H	4.00430400	-0.72555500	-0.38039200

H	3.10694500	-1.17860200	1.07471900

2a$_{syn}$

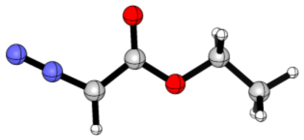

Charge: 0
Spin: 1

C	1.29218000	-0.79971700	-0.00009300
H	1.15195700	-1.86797500	0.00007700
C	0.22684200	0.18791100	-0.00010300
N	2.51823700	-0.35896900	-0.00012000
N	3.56613500	0.04366200	0.00008300
O	0.38476400	1.39242900	-0.00020800
O	-0.96990800	-0.42523100	0.00001000
C	-2.13773200	0.43533100	0.00002600
H	-2.10138000	1.07429200	0.88303200
H	-2.10159300	1.07403100	-0.88317800
C	-3.35617500	-0.45529000	0.00030200
H	-3.37640800	-1.09085400	0.88651800
H	-4.25610800	0.16179800	0.00030700
H	-3.37660800	-1.09113200	-0.88571000

2a (S_1)

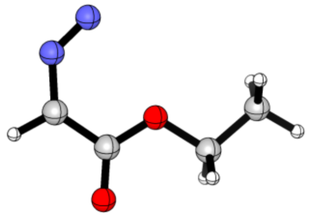

Charge: 0
Spin: 1

C	1.78256000	0.51180100	0.00021300
H	2.52074500	1.29707500	0.00044500
C	0.37458700	0.90819600	0.00002300
N	2.28058400	-0.76956600	0.00006600
N	1.74129100	-1.82298900	-0.00023800
O	0.06673700	2.08674300	-0.00007900
O	-0.48411300	-0.10853600	-0.00000600
C	-1.89928100	0.23005400	-0.00021200
H	-2.10782900	0.83462200	0.88310600
H	-2.10770600	0.83398500	-0.88399800
C	-2.67297200	-1.06417900	0.00020700

H	-2.44358500	-1.65669700	0.88637200
H	-3.74165900	-0.84463700	0.00004800
H	-2.44345300	-1.65735600	-0.88548300

[1]INT1

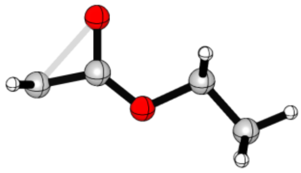

Charge: 0
Spin: 1

C	-2.27445900	-0.80854200	-0.15488900
H	-2.76243800	-0.96384500	0.81273000
C	-1.06973600	-0.10590100	-0.00539400
O	-1.48177300	1.06527700	-0.00169000
O	0.15930400	-0.53789100	0.00903100
C	1.22463500	0.47847300	0.01483500
H	1.09156500	1.10901000	-0.86326200
H	1.10540700	1.08685000	0.91043300
C	2.53907700	-0.25652600	-0.00358800
H	2.64298500	-0.89560200	0.87368500
H	2.63187700	-0.87019600	-0.89994700
H	3.35325600	0.46966000	0.00184800

N_2

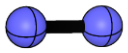

Charge: 0
Spin: 1

N	0.00000000	0.00000000	0.54522200
N	0.00000000	0.00000000	-0.54522200

TS1

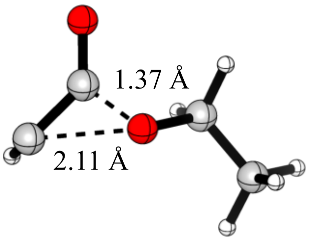

Charge: 0
Spin: 1

C	-1.71733400	1.28741800	0.05192400

H	-1.69929400	1.60417200	1.10211800
C	-1.20677100	-0.03052100	-0.08309900
O	-1.68232900	-1.13293400	-0.00000300
O	0.08786700	0.29019600	-0.41584500
C	1.14298000	-0.46895300	0.26584700
H	0.91668000	-0.48990700	1.33335000
H	1.11241100	-1.48627600	-0.12212000
C	2.45599100	0.21059900	-0.02140100
H	2.65336500	0.23949400	-1.09329000
H	2.46282100	1.22994400	0.36565000
H	3.26051400	-0.34678500	0.46146000

INT2

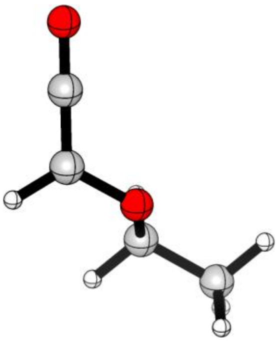

Charge: 0

Spin: 1

C	-1.86134400	0.01774100	0.02219600
O	0.40323900	0.20414400	-0.51945800
C	1.41358000	0.11594800	0.50780700
H	1.58714100	1.11377500	0.92541900
H	1.04809500	-0.52953700	1.31291000
C	2.66898700	-0.44076500	-0.11950500
H	3.02529500	0.21066000	-0.91869700
H	3.45415000	-0.52182100	0.63409700
H	2.48729900	-1.43308800	-0.53470200
O	-2.83014700	-0.62798200	0.07145400
C	-0.77969100	0.76294900	-0.07906400
H	-0.83590800	1.81547300	0.17640200

1a

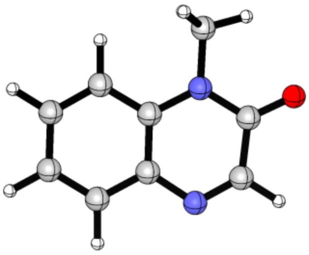

Charge: 0

Spin: 1

C	2.58348800	1.61570600	0.00000000
C	2.42079800	0.24511800	0.00000000
C	1.14003500	-0.32231800	0.00000000
C	0.00000000	0.51454700	0.00000000
C	0.17714800	1.90561100	0.00000000
C	1.45417100	2.44000600	0.00000000
H	3.57417600	2.04899800	0.00000000
H	3.26976700	-0.42546200	0.00000000
H	-0.67484900	2.56722700	0.00000000
H	1.57287300	3.51551000	0.00000000
C	-1.41312300	-1.44683400	0.00000000
C	-0.15580900	-2.20827400	0.00000000
H	-0.26805800	-3.28736100	0.00000000
N	-1.25860500	-0.07056300	0.00000000
N	1.02261400	-1.69845200	0.00000000
O	-2.50729800	-2.00115700	0.00000000
C	-2.45566100	0.76694000	0.00000000
H	-2.47359000	1.39652900	0.88912700
H	-2.47359000	1.39652900	-0.88912700
H	-3.32268400	0.11738000	0.00000000

TS2$_{trans}$

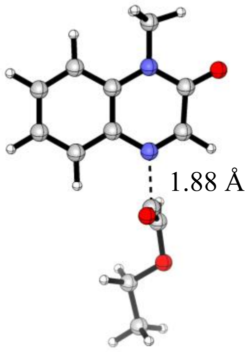

Charge: 0

Spin: 1

C	-1.70250200	-0.44061800	0.82568400
O	-3.77014400	-0.80926100	-0.22714300
C	-4.45635900	0.37292700	-0.66443500
H	-4.15762200	1.21847300	-0.03446700
H	-4.16214400	0.60488100	-1.69545300
C	-5.94472500	0.12534100	-0.57047800
H	-6.49423100	1.01025200	-0.89678200
H	-6.23796800	-0.71420100	-1.20290200
H	-6.23354200	-0.09951100	0.45740700
O	-1.72684300	-0.19247500	1.99751000
C	-2.38950400	-0.71166400	-0.29730600
H	-1.90848500	-0.86709400	-1.25170100
C	0.96398600	3.02694000	0.06274700
C	0.21423400	1.87778400	0.18935300
C	0.83322400	0.62450100	0.11007700
C	2.22633300	0.52405200	-0.10584000
C	2.97208000	1.70440800	-0.23626500
C	2.34297400	2.93230500	-0.14987100
H	0.48746300	3.99510800	0.12499200
H	-0.85319700	1.92100700	0.34687000
H	4.03583800	1.66385200	-0.40786400
H	2.93387500	3.83266900	-0.25226600
C	2.06712500	-1.89243400	-0.09250200
C	0.63399200	-1.68653600	0.14413600
H	0.01962200	-2.57039000	0.25359000
N	2.81257700	-0.73054200	-0.19097500
N	0.08286800	-0.52978100	0.23358800
O	2.54279400	-3.01620500	-0.18412700
C	4.25589400	-0.85141400	-0.39668700
H	4.50965900	-1.90439000	-0.39961600
H	4.53660800	-0.40827500	-1.35140500
H	4.78904300	-0.35014100	0.40969900

TS2*cis*

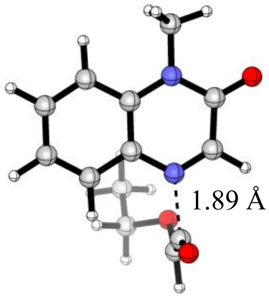

Charge: 0

Spin: 1

C	1.99239000	-1.00532200	-1.43991200
O	2.50126000	-0.58142800	0.87174800
C	3.00778700	0.66324000	1.37124000
H	4.10425900	0.63613600	1.37540500
H	2.69795300	1.47652000	0.70646800
C	2.46742400	0.86803500	2.76787300
H	2.77026300	0.05033000	3.42375500
H	2.84773400	1.80220500	3.18470200
H	1.37717800	0.91515000	2.75566800
O	1.83847800	-1.27375500	-2.59971500
C	2.86569800	-0.85494000	-0.43133400
H	3.91803800	-1.00809900	-0.64776900
C	-0.25261600	2.85961500	-0.99423500
C	0.36282500	1.62703800	-1.03945900
C	-0.31274700	0.48763700	-0.58794500
C	-1.63142200	0.58630800	-0.08859900
C	-2.24077400	1.84825300	-0.04787400
C	-1.55427200	2.96249400	-0.49448200
H	0.26887400	3.73990000	-1.34296700
H	1.36814300	1.51598200	-1.41824500
H	-3.24471100	1.95987700	0.32940200
H	-2.04050800	3.92809000	-0.45578200
C	-1.67352300	-1.80320200	0.30143500
C	-0.30447400	-1.80079800	-0.22328700
H	0.20858300	-2.75275800	-0.26204400
N	-2.28083300	-0.55990200	0.34527100
N	0.30671600	-0.74567600	-0.62167900
O	-2.21427800	-2.83675500	0.67218200
C	-3.64520800	-0.47386800	0.86486300
H	-3.67124000	0.17438900	1.73986300
H	-4.31184800	-0.07936600	0.09922100
H	-3.96411600	-1.47074200	1.14374600

INT3$_{trans}$

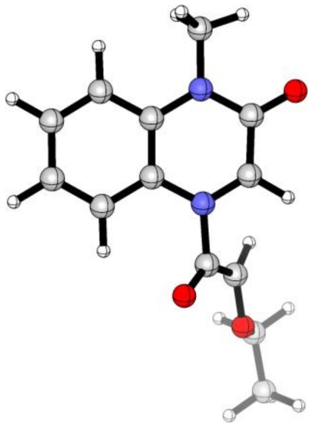

Charge: 0

Spin: 1

C	1.42579600	-0.35309500	0.87433000
O	3.62263400	0.01485000	0.10642000
C	4.38572800	0.34825600	-1.06392100
H	4.00719400	-0.22918000	-1.91373500
H	4.25142100	1.41217400	-1.28916600
C	5.83638600	0.03076600	-0.79078800
H	6.43942000	0.27477700	-1.66689000
H	6.20719700	0.61103200	0.05518400
H	5.96544900	-1.02907900	-0.56780500
O	1.59389300	-1.24279800	1.72139500
C	2.30514300	0.30819600	0.03756500
H	1.97713400	1.00692400	-0.72004200
C	-1.79391300	-2.98187600	-0.23081600
C	-0.77446400	-2.14182100	0.16725900
C	-0.98160900	-0.75928200	0.20843800
C	-2.22719300	-0.20999600	-0.16916600
C	-3.24951500	-1.08554600	-0.56325600
C	-3.03279800	-2.45017000	-0.59131300
H	-1.62914800	-4.05002300	-0.25400400
H	0.18062500	-2.53599500	0.47451900
H	-4.21255300	-0.69752400	-0.85287400
H	-3.83726400	-3.10596200	-0.89600400
C	-1.41941700	2.04185900	0.22776300
C	-0.19109600	1.41776300	0.67603200
H	0.56161700	2.04426100	1.12248900
N	-2.41732200	1.16778600	-0.17423500
N	0.04085000	0.12333400	0.58281500
O	-1.55582400	3.26128600	0.24020800
C	-3.70119400	1.72932000	-0.58707200
H	-3.63687200	2.80771700	-0.50754500

H	-3.91719000	1.45462400	-1.61904200
H	-4.49848000	1.36548100	0.06033800

INT3*cis*

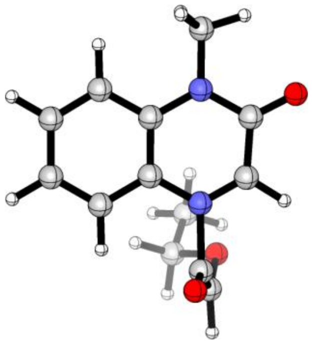

Charge: 0
Spin: 1

C	-1.79609400	0.17075300	-1.62862100
O	-2.62247400	0.74368200	0.55428900
C	-2.94644400	-0.27722900	1.50598200
H	-4.00486400	-0.54769400	1.40733100
H	-2.35438800	-1.17441300	1.29185800
C	-2.65138700	0.24990100	2.89203000
H	-3.24056500	1.14455300	3.09948900
H	-2.89566100	-0.50442200	3.64207800
H	-1.59435200	0.50344400	2.99098700
O	-1.62791200	-0.11555700	-2.81179900
C	-2.81993100	0.36820800	-0.76691800
H	-3.84195600	0.27349500	-1.11684400
C	0.65257800	-3.06122100	-0.19427500
C	-0.11060300	-1.98918600	-0.60072500
C	0.38675600	-0.68882300	-0.45889900
C	1.66476900	-0.45761000	0.09491600
C	2.42356500	-1.56345400	0.50523600
C	1.91814600	-2.84091400	0.35939200
H	0.27291800	-4.06722900	-0.30316300
H	-1.08918700	-2.13053200	-1.03318600
H	3.40210600	-1.42437500	0.93569400
H	2.51790500	-3.68206800	0.68018500
C	1.37897800	1.93287700	-0.17152900
C	0.06281000	1.60717400	-0.72191500
H	-0.56984900	2.42558900	-1.03383100
N	2.13194000	0.84157000	0.22386900
N	-0.37456600	0.40301000	-0.84542500
O	1.75461500	3.09285300	-0.07631900

C	3.45637300	1.08891400	0.79476000
H	3.49801200	0.70968500	1.81477900
H	4.21888600	0.59956700	0.19056500
H	3.62844900	2.15819000	0.79898100

TS3*trans*

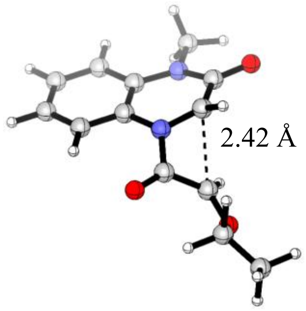

Charge: 0

Spin: 1

C	-1.38034300	-0.76973700	-0.10611900
O	-3.38303500	0.27068400	0.83728200
C	-4.28328300	-0.31430900	-0.14851700
H	-3.96075200	0.01156600	-1.13901300
H	-4.19389100	-1.39730000	-0.09438500
C	-5.67704800	0.16356700	0.17259800
H	-5.74460900	1.25040500	0.11090800
H	-6.37996800	-0.26690600	-0.54244300
H	-5.97417700	-0.14946400	1.17428300
O	-1.76775000	-1.89979600	-0.40792600
C	-2.08216100	0.26537200	0.60470300
H	-1.52922900	0.88487100	1.29767400
C	2.52956200	-2.77293800	0.02289800
C	1.28237000	-2.21551900	-0.21477700
C	1.13077800	-0.83076400	-0.20571600
C	2.22675500	0.01983600	0.04780800
C	3.47744100	-0.56567900	0.27132200
C	3.62260000	-1.94602200	0.26037700
H	2.64700400	-3.84781200	0.01304100
H	0.42027100	-2.83208000	-0.41503300
H	4.34110300	0.05272900	0.45767200
H	4.60051300	-2.37402100	0.43587300
C	0.87173400	2.01505500	-0.35092400
C	-0.20984400	1.11291700	-0.68828800
H	-0.95747400	1.45733800	-1.37913900
N	2.04134500	1.40711700	0.09828500
N	-0.11094000	-0.21533500	-0.40638800
O	0.76545600	3.23298300	-0.47021200
C	3.15797400	2.26678100	0.47585200
H	3.56476900	1.94520100	1.43348000
H	3.94677800	2.23823500	-0.27792900
H	2.79023700	3.28243800	0.56127200

TS3$_{cis}$

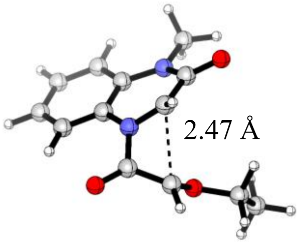

Charge: 0

Spin: 1

C	-0.88308700	-1.93681800	-0.66180800
O	-0.63326200	-3.12322400	-0.86433300
C	-2.18870200	-1.37825400	-0.38155400
C	3.25731100	-1.60289100	0.84901900
C	2.04029800	-1.81171800	0.21814100
C	1.27212300	-0.71889600	-0.17493700
C	1.69982400	0.60243500	0.07047200
C	2.93752600	0.78748000	0.69427300
C	3.70378900	-0.30505700	1.07856300
H	3.85759200	-2.45025500	1.15068900
H	1.67578300	-2.80693200	0.01310700
H	3.30674000	1.78272000	0.88473500
H	4.65779500	-0.13583100	1.55966700
C	-0.23079700	1.53348000	-1.11748600
C	-0.58491400	0.16798400	-1.43950400
H	-1.10682100	-0.02401400	-2.35961000
N	0.87256400	1.67969500	-0.27715400
N	0.02220100	-0.86043700	-0.77475100
O	-0.84905100	2.49636300	-1.56565200
C	1.26372500	3.03214000	0.10545400
H	1.44709300	3.07381000	1.17816200
H	2.16386200	3.34536400	-0.42643800
H	0.45325500	3.70464800	-0.14951000
O	-2.34593700	-0.58383200	0.68512200
C	-3.58175300	0.16286300	0.73403100
H	-3.55405500	0.90459900	-0.06912100
H	-4.41567500	-0.52057200	0.54671700
C	-3.68726600	0.81376500	2.09004900
H	-2.84623300	1.48601200	2.26312500
H	-4.60833100	1.39595300	2.14344400
H	-3.70675300	0.06430600	2.88208300
H	-3.07007900	-1.94815000	-0.68308300

TS4$_{\textit{rotation}}$

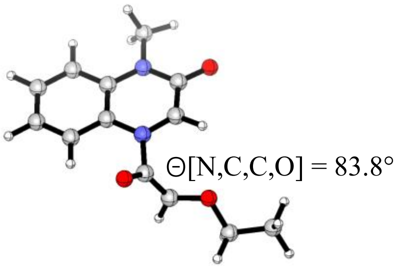

Charge: 0

Spin: 1

C	0.92524300	-2.05153400	0.63131500
O	2.43476200	-0.93131400	-0.68985100
C	2.43020500	0.13518600	-1.67595300
H	2.42256600	-0.32137700	-2.66975300
H	1.51393700	0.71822100	-1.55159100
C	3.65989300	0.97958600	-1.45945500
H	3.68802400	1.77832200	-2.20238400
H	3.64936200	1.43197800	-0.46760900
H	4.56568800	0.38141000	-1.56406100
O	1.26122800	-2.82991000	1.46486900
C	1.36269700	-1.75921100	-0.72066500
H	0.62141100	-1.59663400	-1.49240200
C	-3.17593500	-1.55629800	-0.69454900
C	-1.98727700	-1.76126700	-0.01089100
C	-1.17302900	-0.67930500	0.30884200
C	-1.53892900	0.63623200	-0.03128200
C	-2.75108400	0.82544900	-0.70635700
C	-3.55076500	-0.26048600	-1.03726500
H	-3.80807600	-2.39688900	-0.94427100
H	-1.68515700	-2.75803100	0.28316800
H	-3.07027200	1.81899400	-0.97968400
H	-4.48135100	-0.08718700	-1.56113700
C	0.36774500	1.51942500	1.20698000
C	0.67199900	0.17059300	1.56144800
H	1.49236900	-0.02835700	2.23048400
N	-0.68626600	1.68828600	0.28859600
N	0.04499500	-0.87918600	0.98256100
O	0.98407900	2.48812600	1.65564500
C	-1.00921800	3.03956600	-0.14791300
H	-0.17490000	3.68416500	0.10326900
H	-1.16912100	3.05512300	-1.22526200
H	-1.90539200	3.41372800	0.35230700

3a$_{trans}$

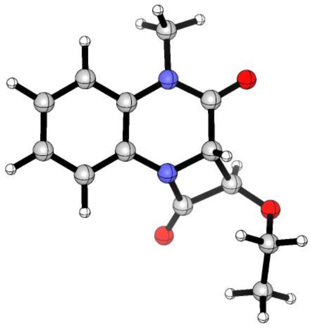

Charge: 0

Spin: 1

C	-1.31226400	-0.83852700	0.74644300
O	-3.20294200	0.80949000	0.14697600
C	-3.70757000	-0.02465300	-0.91417500
H	-4.25791200	0.63923000	-1.58229900
H	-2.88417600	-0.45697300	-1.48893700
C	-4.61222700	-1.11093600	-0.36711700
H	-5.44033500	-0.67088700	0.19011500
H	-5.02567100	-1.69962000	-1.18909400
H	-4.06356400	-1.77905000	0.29650800
O	-1.59178600	-1.82896000	1.37026300
C	-1.90085900	0.59383600	0.55303900
H	-1.74743700	1.16838800	1.46751400
C	2.84242200	-2.56388900	-0.25477100
C	1.48992200	-2.24743400	-0.20680600
C	1.09466900	-0.91876400	-0.13915500
C	2.03832600	0.12572400	-0.10759500
C	3.39076500	-0.20987800	-0.18484400
C	3.78432600	-1.54324600	-0.25508200
H	3.15330300	-3.59845400	-0.30345900
H	0.73265500	-3.01853600	-0.22313300
H	4.14738200	0.55886500	-0.17767000
H	4.83926400	-1.77688900	-0.30775000
C	0.30711400	1.88473200	-0.11225100
C	-0.69514800	0.80705700	-0.43452200
H	-1.00275700	0.90304900	-1.47777100
N	1.61282500	1.46896000	0.04828300
N	-0.24408100	-0.53259600	-0.07821800
O	-0.04171300	3.05048300	-0.02321200
C	2.62214400	2.49114000	0.32217800
H	3.23438000	2.18896000	1.17028900
H	3.26158700	2.64981100	-0.54716700
H	2.11388400	3.41848900	0.55813200

3a$_{cis}$

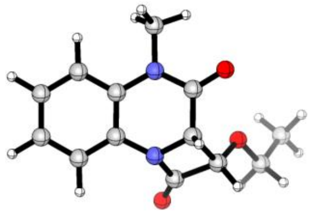

Charge: 0

Spin: 1

C	-0.89330400	-1.65111900	-0.28189000
O	-0.99181500	-2.64648700	0.38714700
C	-1.86755800	-0.59872100	-0.89396000
C	3.67792200	-1.59766400	0.35518500
C	2.40107700	-1.83194000	-0.14249200
C	1.50873900	-0.77862500	-0.27454000
C	1.86198200	0.53284900	0.09337900
C	3.15768200	0.75547300	0.56238900
C	4.05156700	-0.30378700	0.69392000
H	4.37440700	-2.41755500	0.46496700
H	2.08574000	-2.82471000	-0.43169800
H	3.47748900	1.74745700	0.84026000
H	5.04757900	-0.10614200	1.06715000
C	-0.26449500	1.49736000	-0.71135000
C	-0.56892400	0.15210800	-1.32633100
H	-0.45129700	0.23958200	-2.40820600
N	0.90782900	1.57766000	0.01637400
N	0.20261700	-0.94689400	-0.74350300
O	-0.99670400	2.45030400	-0.90368600
C	1.23983700	2.86914600	0.61556200
H	1.57646000	2.72244600	1.64028400
H	2.02036600	3.37815200	0.04786100
H	0.34895900	3.48598700	0.61538000
O	-2.62951600	0.08330800	0.04966200
C	-3.83065300	-0.60522300	0.42902500
H	-4.44048900	-0.77944100	-0.46580000
H	-3.57868800	-1.57822800	0.86074700
C	-4.56647300	0.25604300	1.42818700
H	-4.81342600	1.22604900	0.99462900
H	-5.49329100	-0.23484200	1.72958500
H	-3.95818800	0.41877600	2.31893000
H	-2.46887000	-0.96529800	-1.73167600

[1]TS5

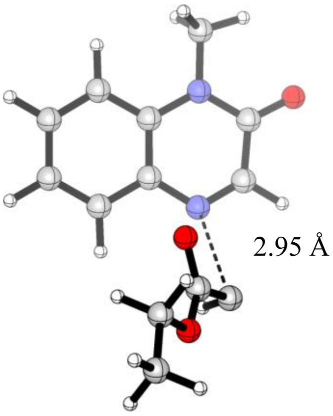

Charge: 0

Spin: 1

C	2.14307800	-1.46959900	1.23876400
H	1.77522800	-0.86243200	2.06918800
C	2.75547600	-0.68533800	0.22430500
O	1.93695500	-0.32852400	-0.61986500
O	4.06025100	-0.48604100	0.20192500
C	4.60750600	0.20483300	-0.96828600
H	4.31486000	-0.34845800	-1.86023400
H	4.16800400	1.20071800	-1.01514500
C	6.10448500	0.25439400	-0.79784100
H	6.52501500	-0.75003700	-0.74160800
H	6.54644100	0.76463900	-1.65506600
H	6.37845100	0.80077200	0.10526700
C	-0.89663000	2.94681200	0.70956800
C	-0.48801600	1.71678800	1.18289600
C	-1.14390200	0.54534600	0.78639100
C	-2.23451200	0.61789100	-0.10988600
C	-2.64141700	1.87292500	-0.58493200
C	-1.97615100	3.01579300	-0.17599700
H	-0.38637200	3.84894900	1.01794000
H	0.34532200	1.62357200	1.86614000
H	-3.47073000	1.95920000	-1.26941600
H	-2.30297100	3.97607300	-0.55273500
C	-2.45501300	-1.78759600	-0.00354300
C	-1.30554600	-1.73571000	0.91114300
H	-0.95931400	-2.69421500	1.28145200
N	-2.86738000	-0.55813000	-0.48897800
N	-0.69991500	-0.66528300	1.27730600
O	-2.99473000	-2.84638900	-0.30689200
C	-3.99516400	-0.51742000	-1.41666300
H	-4.32769200	-1.53415900	-1.58856300

H	-4.81238000	0.06371400	-0.99047100
H	-3.68776300	-0.07152000	-2.36190500

[1]TS6

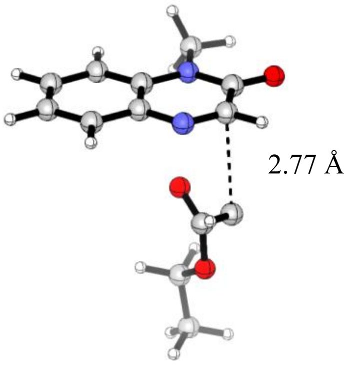

Charge: 0

Spin: 1

C	-1.67097600	-0.92165500	1.66529600
H	-1.35087100	-0.25722100	2.47427100
C	-2.12397900	-0.19629300	0.51926400
O	-1.26550900	0.03584900	-0.32413500
O	-3.41573000	0.07672500	0.42650600
C	-3.88036100	0.68967900	-0.81944900
H	-3.35851400	1.63691500	-0.95165200
H	-3.61699600	0.02803500	-1.64426700
C	-5.37113300	0.87678400	-0.69649100
H	-5.61633500	1.53056200	0.14113900
H	-5.75187600	1.33364900	-1.61131300
H	-5.87527200	-0.07956600	-0.55458700
C	2.28274100	2.85413300	0.45472800
C	1.88614300	1.89300900	1.36051200
C	1.65899500	0.57233700	0.94860500
C	1.83949100	0.21651900	-0.40899800
C	2.24572000	1.20196000	-1.31974200
C	2.46042400	2.49850300	-0.88658500
H	2.45494000	3.87224100	0.77575800
H	1.73728600	2.12709100	2.40626600
H	2.38786900	0.96113600	-2.36146300
H	2.77104100	3.24644600	-1.60427300
C	1.18136300	-2.04616800	0.11289000
C	1.02314100	-1.55773200	1.49206100
H	0.72763500	-2.30530900	2.21729300
N	1.60781700	-1.09292200	-0.79446400
N	1.25732800	-0.35125100	1.88580500
O	0.95950900	-3.21295600	-0.18922000
C	1.76763100	-1.48420600	-2.19182100
H	1.08190700	-0.91573800	-2.81961800
H	2.79157000	-1.30698400	-2.51877000
H	1.54024500	-2.54022200	-2.27499700

MECP

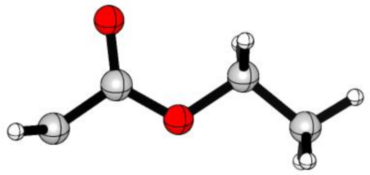

Charge: 0

Spin: 1

C	-2.16903700	-0.97673400	-0.10845300
H	-2.87829100	-1.17315400	0.68931700
C	-1.11697700	-0.01180900	-0.01614100
O	-1.41958000	1.17360000	-0.00232500
O	0.12097400	-0.49373500	-0.02959400
C	1.21298900	0.47439600	0.02380700
H	1.12830500	1.13799200	-0.83660600
H	1.10427600	1.07030400	0.93037200
C	2.49880000	-0.31241600	0.00964600
H	2.55376000	-0.98070800	0.86936300
H	2.57980300	-0.90734300	-0.90069000
H	3.34634700	0.37337200	0.05044400

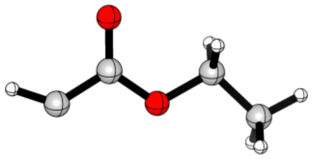

^{3}INT1

Charge: 0

Spin: 3

C	2.14170500	-1.01355300	0.00023900
H	3.21697900	-0.90528600	0.00090100
C	1.12156900	-0.01245400	-0.00038200
O	1.39789300	1.17895500	0.00031800
O	-0.12284400	-0.50359600	-0.00054300
C	-1.20746000	0.46449400	-0.00030200
H	-1.10828800	1.09625700	0.88280500
H	-1.10904400	1.09577800	-0.88384400
C	-2.50424600	-0.30667300	0.00044900
H	-2.58476600	-0.93728100	-0.88525800
H	-2.58404800	-0.93674500	0.88660200
H	-3.34062700	0.39352600	0.00057300

[3]TS1

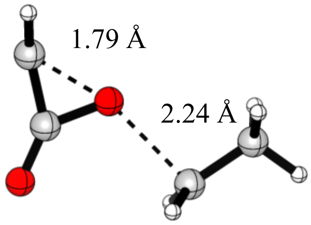

Charge: 0

Spin: 3

C	-2.16329700	0.97391100	-0.08664000
H	-2.77672200	1.63311300	0.50293200
C	-1.13875400	-0.03334500	-0.01957300
O	-1.37698500	-1.22728100	0.01180000
O	0.09918400	0.48482400	-0.02574400
C	1.20450400	-0.46042900	0.02448500
H	1.11014100	-1.05666400	0.93270400
H	1.12392900	-1.13096200	-0.83191500
C	2.48492900	0.33691200	0.00160600
H	2.55939400	0.93170000	-0.90944600
H	2.54547500	1.00531700	0.86124800
H	3.33589800	-0.34514300	0.03676400

[3]TS5

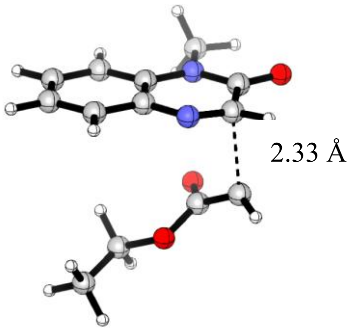

Charge: 0

Spin: 3

C	-0.19408500	-2.40501900	0.82091000
H	-0.51442500	-2.89315200	1.72863000
C	-0.92466300	-1.81703800	-0.26236900
O	-0.46591400	-1.61495400	-1.37468900
O	-2.17732100	-1.47538800	0.10711900
C	-2.97790200	-0.77505400	-0.88154700
H	-2.41473400	0.08986000	-1.23380600
H	-3.14556300	-1.44018400	-1.72974700
C	-4.26940400	-0.37052500	-0.21458100

H	-4.07946300	0.29405100	0.62907500
H	-4.89865200	0.15747300	-0.93298800
H	-4.81649600	-1.24321000	0.14408100
C	-0.82937200	3.06526600	0.62092200
C	-0.49444700	2.05029600	1.48884400
C	0.41553500	1.04794100	1.10750600
C	1.00753800	1.09226600	-0.18393700
C	0.65953400	2.13461100	-1.05377000
C	-0.24741200	3.09967800	-0.65247800
H	-1.53566300	3.82859200	0.91687500
H	-0.92583300	1.98422900	2.47861400
H	1.08797000	2.19052800	-2.04194400
H	-0.50721600	3.89323300	-1.34060300
C	2.21712600	-0.93586200	0.31577600
C	1.44615300	-0.94121800	1.57812900
H	1.74758000	-1.69910200	2.29099200
N	1.91769100	0.11047900	-0.53809400
N	0.69306900	0.04410200	1.99237600
O	3.05640900	-1.78985100	0.06710100
C	2.56467100	0.14661600	-1.84621600
H	1.81308400	0.09075400	-2.63237100
H	3.14330900	1.06304300	-1.95699300
H	3.22575500	-0.70838400	-1.91916400

[3]TS6

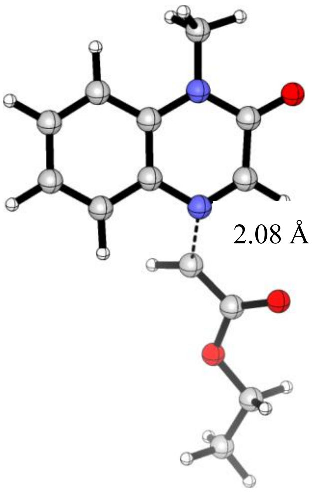

Charge: 0
Spin: 3

C	1.51336200	0.26308600	0.57194900
H	1.42057200	1.11121000	1.23570100
C	2.70249800	-0.38713100	0.11351600
O	2.72715500	-1.46147400	-0.46799200

O	3.81757100	0.33669000	0.38117200
C	5.07733300	-0.22281300	-0.06732200
H	5.21638800	-1.19644300	0.40496100
H	5.02918200	-0.37707600	-1.14633800
C	6.16887800	0.74771500	0.31377500
H	6.20211500	0.89473600	1.39407300
H	7.13459300	0.35478000	-0.00856300
H	6.01615800	1.71599800	-0.16470900
C	-1.82701700	3.08141600	-0.27461400
C	-0.86487700	2.11289400	-0.48025100
C	-1.17422200	0.75819700	-0.32493000
C	-2.48121900	0.36604900	0.03705800
C	-3.44942800	1.36039400	0.24353000
C	-3.11916400	2.69561000	0.09031000
H	-1.58302700	4.12763100	-0.39686800
H	0.14453500	2.37615600	-0.76584000
H	-4.45691100	1.09270300	0.52061200
H	-3.88031900	3.44701500	0.25383800
C	-1.82025600	-1.95759600	-0.10911700
C	-0.50375000	-1.45220600	-0.48022500
H	0.26797300	-2.18600300	-0.66948300
N	-2.77280600	-0.98468800	0.16629700
N	-0.18507000	-0.19241000	-0.55149200
O	-2.06156000	-3.16007500	-0.05197500
C	-4.11199300	-1.41077000	0.56131300
H	-4.11294700	-2.49148500	0.63869700
H	-4.37500700	-0.97734200	1.52560500
H	-4.84437400	-1.10240100	-0.18491400

9. Crystal data

Method for single crystals cultivation: the single crystal for compound **3k** (CCDC: 2429896) or **12** (CCDC: 2494770) were prepared from a mixture solvent of DCM and PE (v/v = 1:1). A pure solid sample (10–20 mg) was dissolved in DCM (2 mL) in a vial at room temperature, and PE (2-3 mL) was added into the above solution slowly while keeping the sample completely dissolved. The vial was properly sealed with parafilm and kept at room temperature to allow the slow evaporation of the solvents until a single crystal was obtained.

(1) Crystal data of 3k (Thermal ellipsoids are shown with 50% probability.)

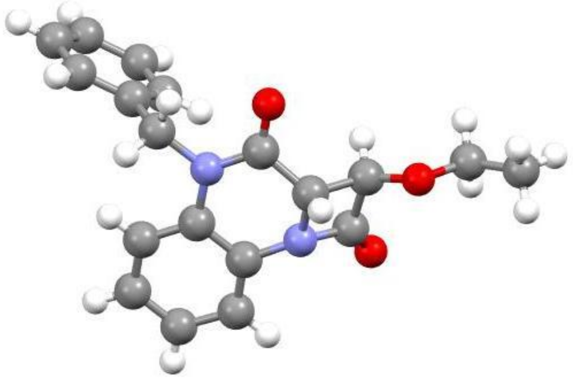

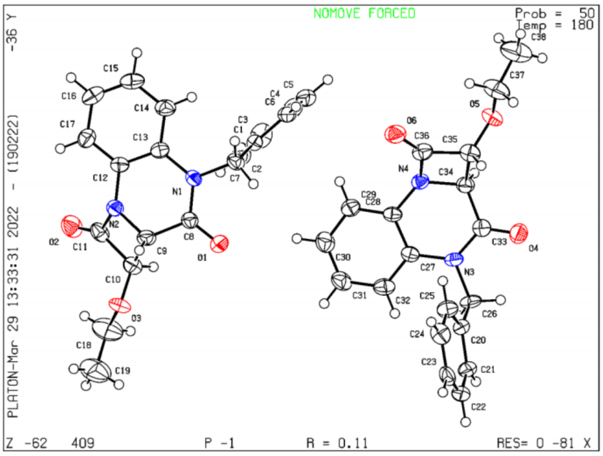

Table S2 Crystal data and structure refinement for 3k.

Identification code	**3k**
Empirical formula	$C_{19}H_{18}N_2O_3$
Formula weight	322.35
Temperature/K	179.99(10)
Crystal system	triclinic
Space group	P-1
a/Å	9.5496(7)
b/Å	10.1080(8)
c/Å	17.3620(16)
α/°	90.483(7)
β/°	97.956(7)
γ/°	100.119(6)

Volume/Å^3	1633.0(2)
Z	4
ρ_{calc}g/cm^3	1.311
μ/mm^{-1}	0.090
F(000)	680.0
Crystal size/mm^3	0.14 × 0.13 × 0.12
Radiation	Mo Kα (λ = 0.71073)
2Θ range for data collection/°	4.096 to 50.078
Index ranges	$-11 \leq h \leq 11$, $-12 \leq k \leq 12$, $-5 \leq l \leq 20$
Reflections collected	5743
Independent reflections	5743 [R_{int} = ?, R_{sigma} = 0.1252]
Data/restraints/parameters	5743/0/436
Goodness-of-fit on F^2	1.080
Final R indexes [I>=2σ (I)]	R_1 = 0.1126, wR_2 = 0.2851
Final R indexes [all data]	R_1 = 0.1432, wR_2 = 0.3080
Largest diff. peak/hole / e Å^{-3}	0.60/-0.52

(2) Crystal data of 12 (Thermal ellipsoids are shown with 50% probability.)

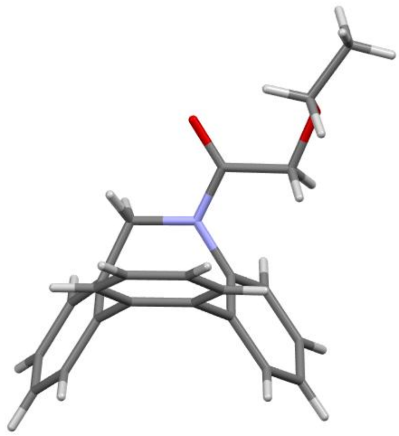

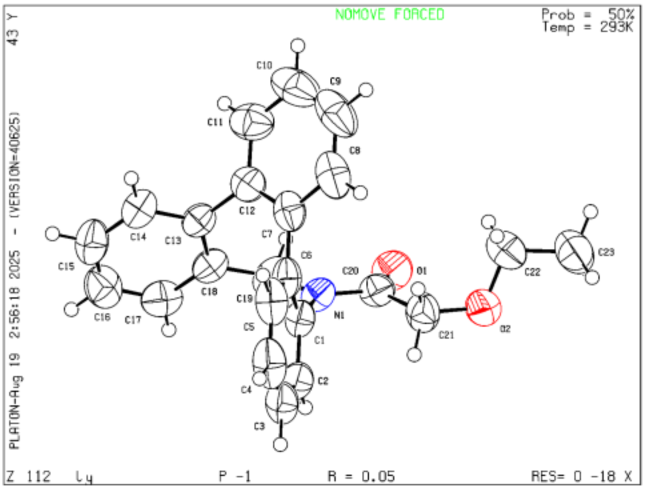

Table S3 Crystal data and structure refinement for 12.

Identification code	**12**
Empirical formula	$C_{23}H_{21}NO_2$
Formula weight	343.41
Temperature/K	293(2)
Crystal system	triclinic
Space group	P-1
a/Å	8.5050(3)
b/Å	9.0705(2)
c/Å	13.1678(4)
α/°	84.919(2)
β/°	71.480(3)
γ/°	75.360(2)
Volume/Å^3	931.91(5)
Z	2
ρ_{calc}g/cm^3	1.224
μ/mm^{-1}	0.614
F(000)	364.0
Crystal size/mm^3	0.23 × 0.18 × 0.15
Radiation	Cu Kα (λ = 1.54184)
2Θ range for data collection/°	7.08 to 133.196
Index ranges	-10 ≤ h ≤ 9, -10 ≤ k ≤ 10, -15 ≤ l ≤ 15
Reflections collected	13882
Independent reflections	3281 [R_{int} = 0.0350, R_{sigma} = 0.0277]
Data/restraints/parameters	3281/3/236
Goodness-of-fit on F^2	1.045
Final R indexes [I>=2σ (I)]	R_1 = 0.0542, wR_2 = 0.1537
Final R indexes [all data]	R_1 = 0.0671, wR_2 = 0.1643
Largest diff. peak/hole / e Å^{-3}	0.28/-0.27

10. References

[1] M. Gao, Y. Li, L.-J. Xie, R. Chauvin, X.-L. Cui, *Chem. Commun.*, 2016, **52**, 2846-2849.

[2] (a) X.-B. Qi, J. M. Ready, *Angew. Chem.*, *Int. Ed*., 2007, **46**, 3242-3244; (b) J. S. Baum, D. A. Shook, H. M. L. Davies, H. D. Smith, *Synth. Commun*., 1987, **17**, 1709-1716; (c) A. Audic, R. Oriez, J. Prunet, *Tetrahedron*, 2021, **79**, 131843; (d) H.-Y. Guo, S. Zhang, X.-Q. Yu, X.-J. Feng, Y. Yamamoto, M. Bao, *ACS Catal.*, 2021, **11**, 10789-10795.

[3] (a) J.-H. Ye, P. Bellotti, T. O. Paulisch, C. G. Daniliuc, F. Glorius, *Angew. Chem.*, *Int. Ed.* 2021, **60**, 13671-13676; (b) R. Wang, M.-Y. Ma, X. Gong, G. B. Panetti, X.-Y. Fan, P. J. Walsh, *Org. Lett.*, 2018, **20**, 2433–2436; (c) D. C. Elliott, A. Marti, P. Mauleón, A. Pfaltz, *Chem. Eur. J.*, 2019, **25**, 1918-1922.

[4] K. Livingstone, S. Bertrand, A. R. Kennedy, C. Jamieson, *Chem. Eur. J.*, 2020, **26**, 10591-10597.

[5] (a) D. Zhang, J. Zhou, T. Qin, X. Yang, *Chem Catal.*, 2024, **4**, 100827; (b) U. Pathak, S. Bhattacharyya, V. Dhruwansh, L. K. Pandey, R. Tank, M. V. S. Suryanarayana. *Green Chem*., 2011, **13**, 1648; (c) A. F. Garrido-Castro, A. Gini, M. C. Maestro, J. Alemán, *Chem. Commun*., 2020, **56**, 3769-3772.

[6] M. C. Mollo, J. A. Bisceglia, N. B. Kilimciler, M. Mancinelli, L. R. Orelli, *Synthesis*, 2020, **52**, 1666−1679.

[7] M. J. Frisch, *et al. Gaussian 16, RevisionC.01*, Gaussian, Inc., Wallingford CT, **2019**.

[8] (a) A. D. Becke, *Phys. Rev. A* **1988**, *38*, 3098; (b) A. D. Becke, *J. Chem. Phys.* **1993,** *98,* 5648.

[9] S. Grimme, J. Antony, S. Ehrlich, H. Krieg, *J. Chem. Phys.* **2010**, *132*, 154104.

[10] (a) F. Weigend, R. Ahlrichs, R. *Phys. Chem. Chem. Phys.* **2005**, *18*, 3297-3305; (b) F. Weigend, *Phys. Chem. Chem. Phys.* **2006**, *8*, 1057-1065.

[11] (a) K. Fukui, *J. Phys. Chem.* **1970**, *74*, 4161-4163; (b) K. Fukui, *Acc. Chem. Res*. **1981**, *14*, 363-368; (c) H. P. Hratchian, H. B. Schlegel, In Theory and Applications of Computational Chemistry: The First 40 Years, Ed. C. E. Dykstra, G. Frenking, K. S. Kim, G. Scuseria (Elsevier, Amsterdam, **2005**) 195-249.

[12] F. Trani, G. Scalmani, G. S. Zheng, I. Carnimeo, M. J. Frisch, V. Barone, *J. Chem. Theory Comput.* **2011**, *7*, 3304-3313.

[13] (a) C. Bannwarth, S. Ehlert, S. Grimme, *J. Chem. Theory. Comput.* **2019**, *15*, 1652-1671; (b) F. Neese, *Comput. Molec. Sci.* **2022**, *12*, e1606; (c) C. Bannwarth, E. Caldeweyher, S. Ehlert, A. Hansen, P. Pracht, J. Seibert, S. Spicher, S. Grimme, *Comput. Molec. Sci.* **2020**, *11*, e1493.

[14] (a) S. Miertuš, E. Scrocco, J. Tomasi C*hem. Phys.* **1981**, *55*, 117; (b) R. Cammi, J. Tomasi, *J. Comp. Chem.* **1995**, *16*, 1449; (c) M. Cossi, V. Barone, R. Cammi, J. Tomasi *Chem. Phys. Lett.* **1996**, *255*, 327; (d) C. Amovilli, V. Barone, R. Cammi, E. Cancès, M. Cossi, B. Mennucci, C. S. Pomelli, J. Tomasi, *Adv. Quantum. Chem.* **1998**, *32*, 227.

[15] T. Lu, sobMECP program; http://sobereva.com/286.

[16] CYLview20; C. Y. Legault, Universitéde Sherbrooke, **2020**, (http://www.cylview.org).

11. Copies of ^{1}H NMR, ^{13}C NMR and ^{19}F NMR Spectra

^{1}H NMR (400 MHz) Spectrum of 3a in $CDCl_3$

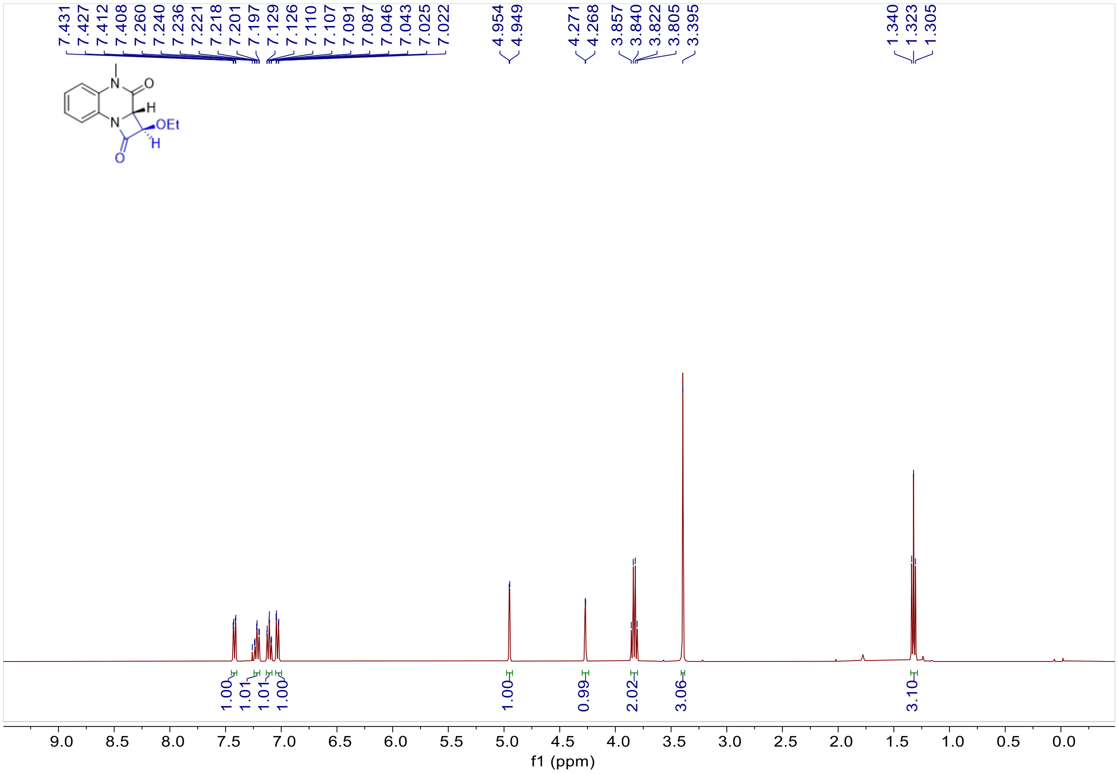

^{13}C NMR (100 MHz) Spectrum of 3a in $CDCl_3$

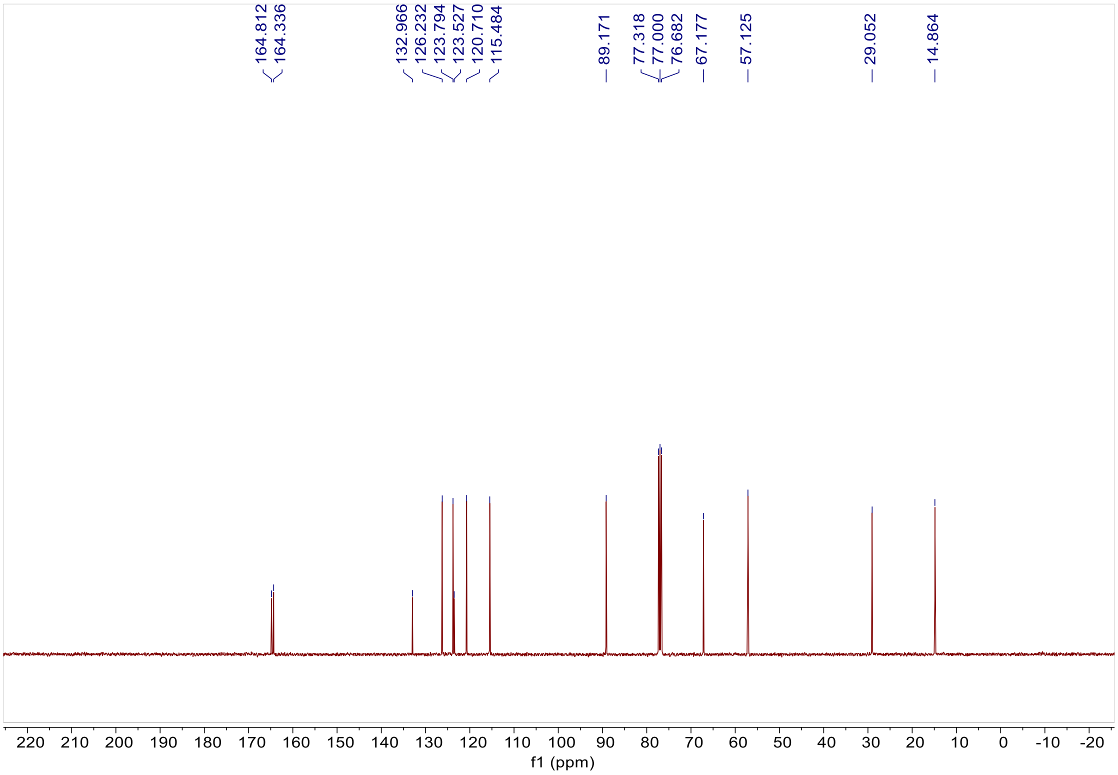

^{1}H NMR (600 MHz) Spectrum of 3b in $CDCl_3$

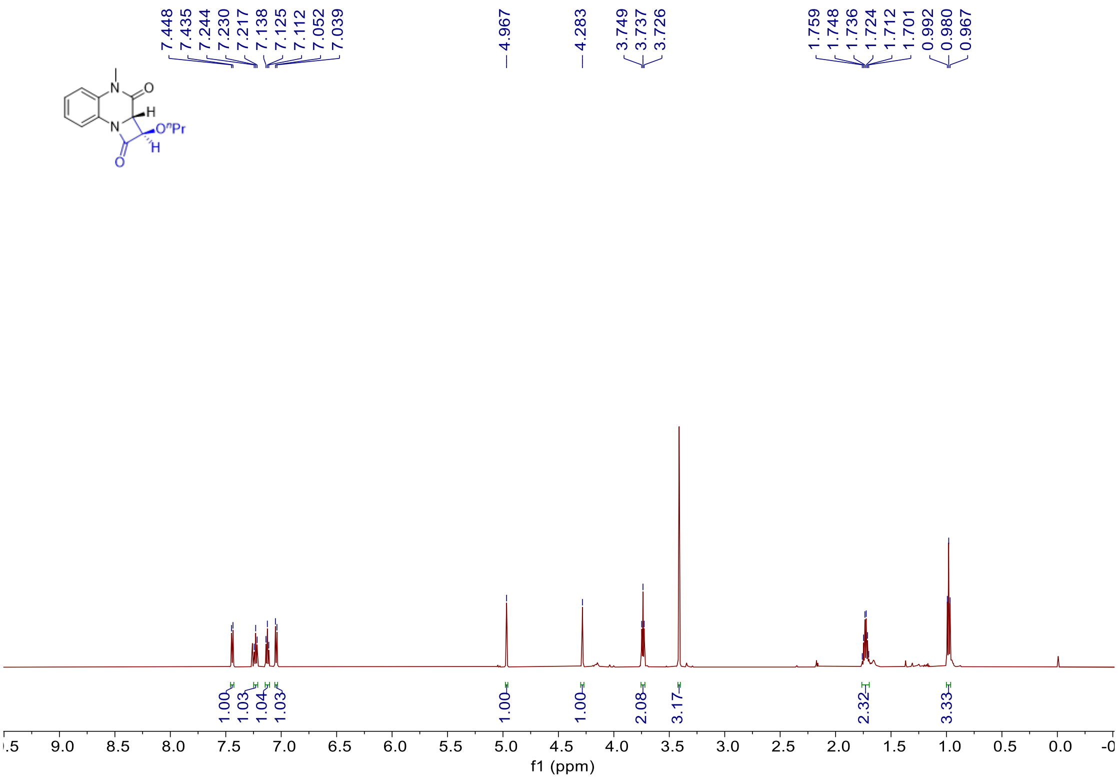

^{13}C NMR (150 MHz) Spectrum of 3b in $CDCl_3$

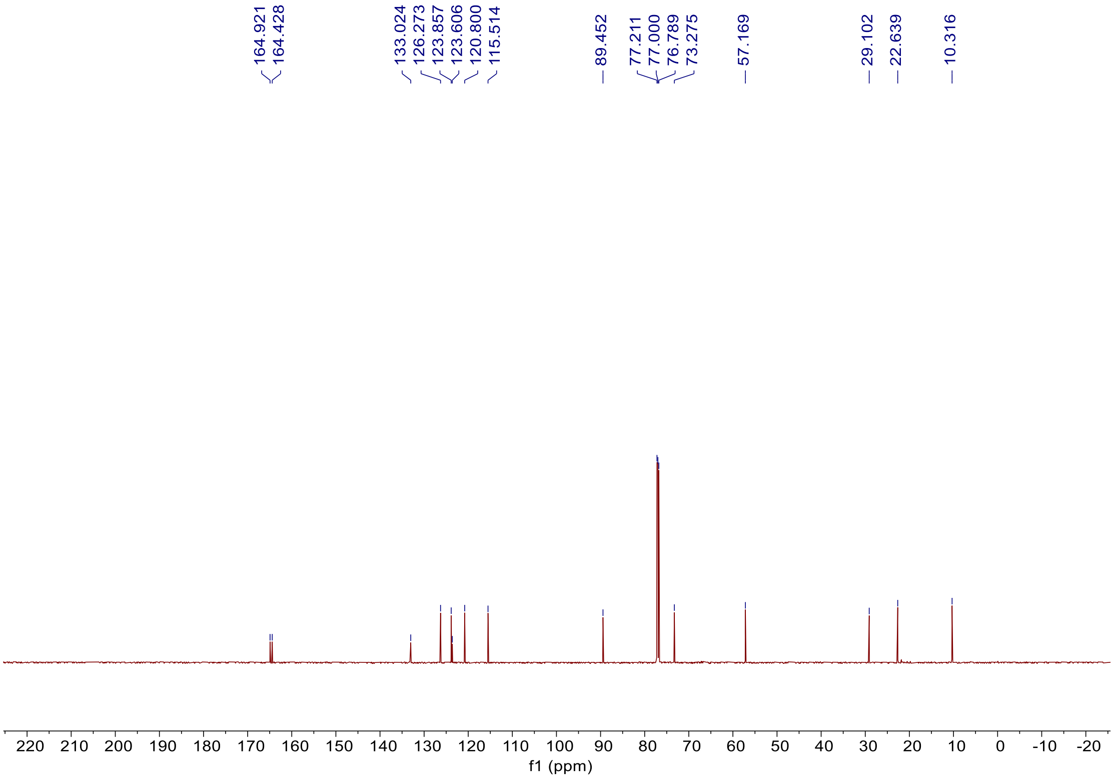

^{1}H NMR (600 MHz) Spectrum of 3c in $CDCl_3$

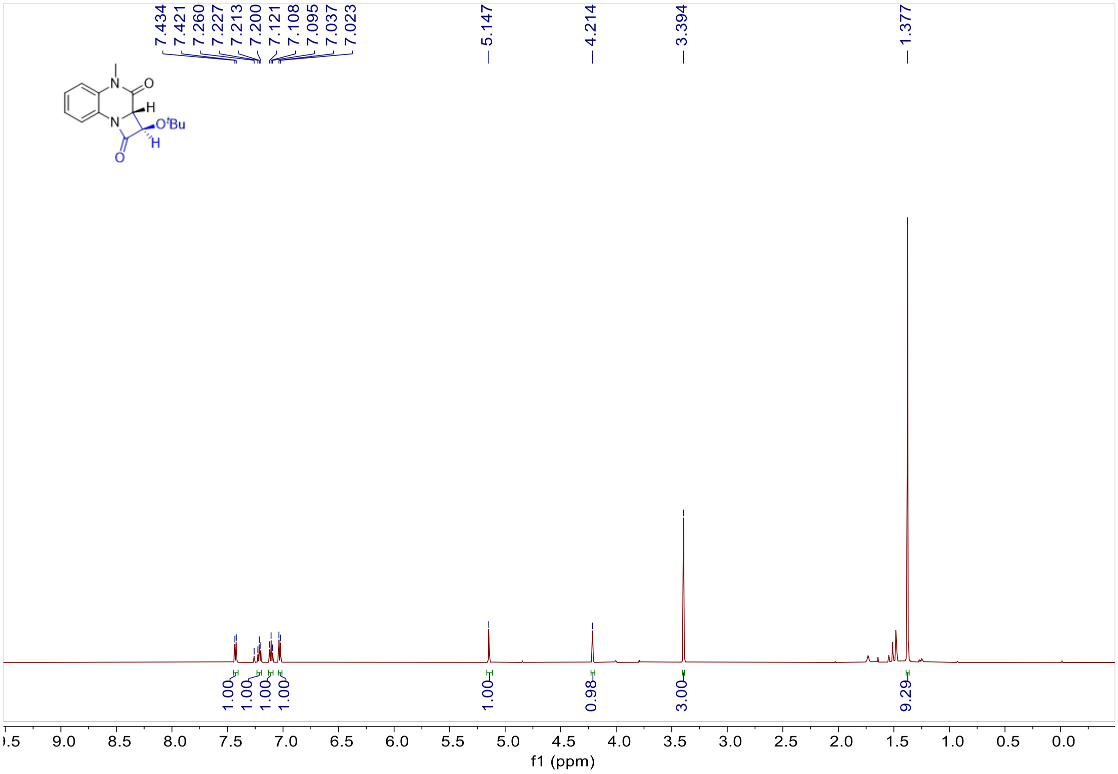

^{13}C NMR (150 MHz) Spectrum of 3c in $CDCl_3$

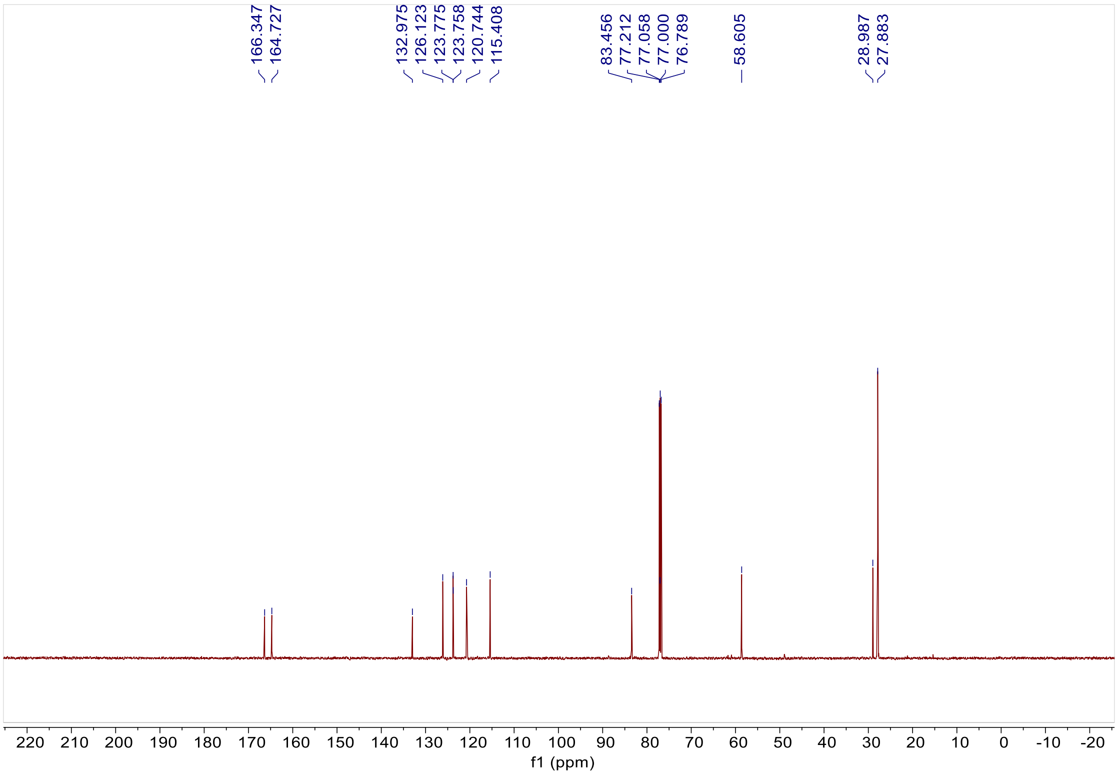

^{1}H NMR (600 MHz) Spectrum of 3d in CDCl₃

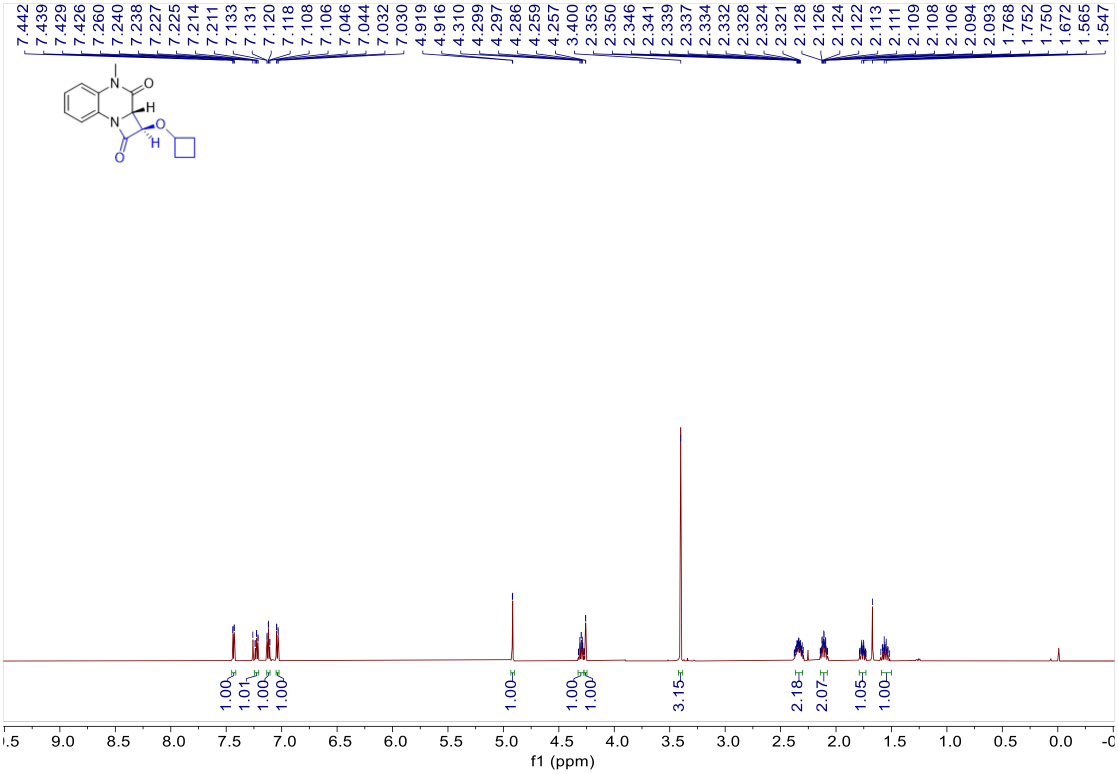

^{13}C NMR (150 MHz) Spectrum of 3d in CDCl₃

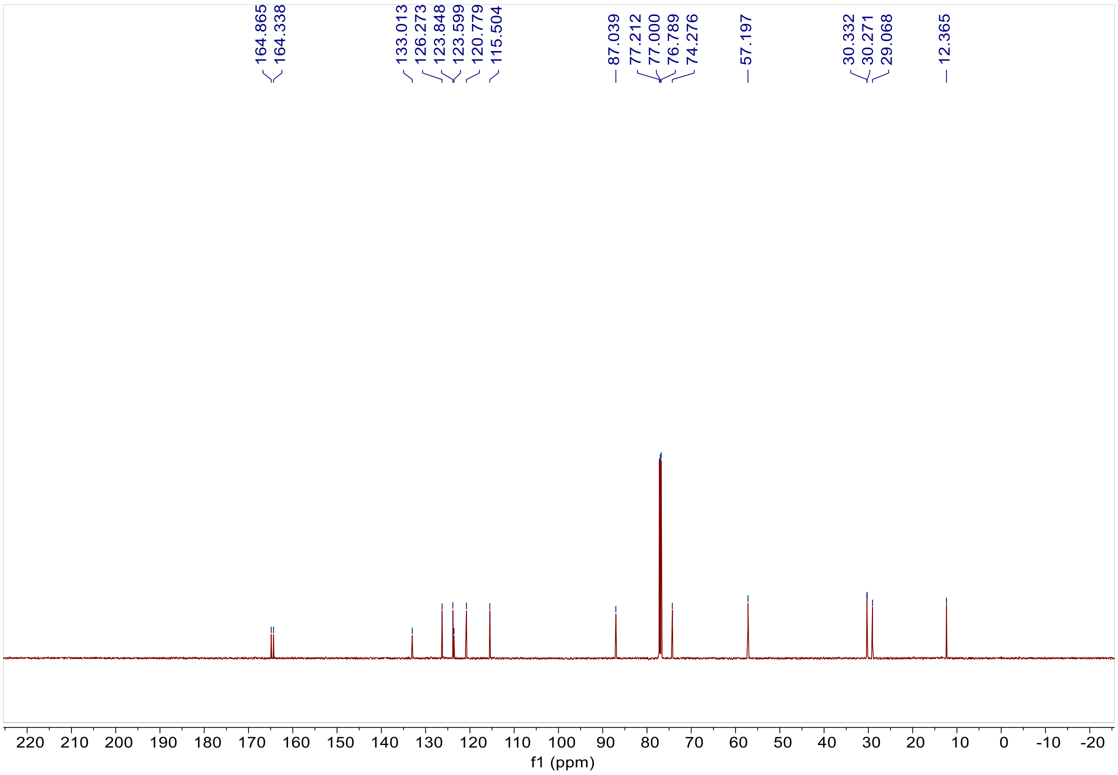

^{1}H NMR (600 MHz) Spectrum of 3e in $CDCl_3$

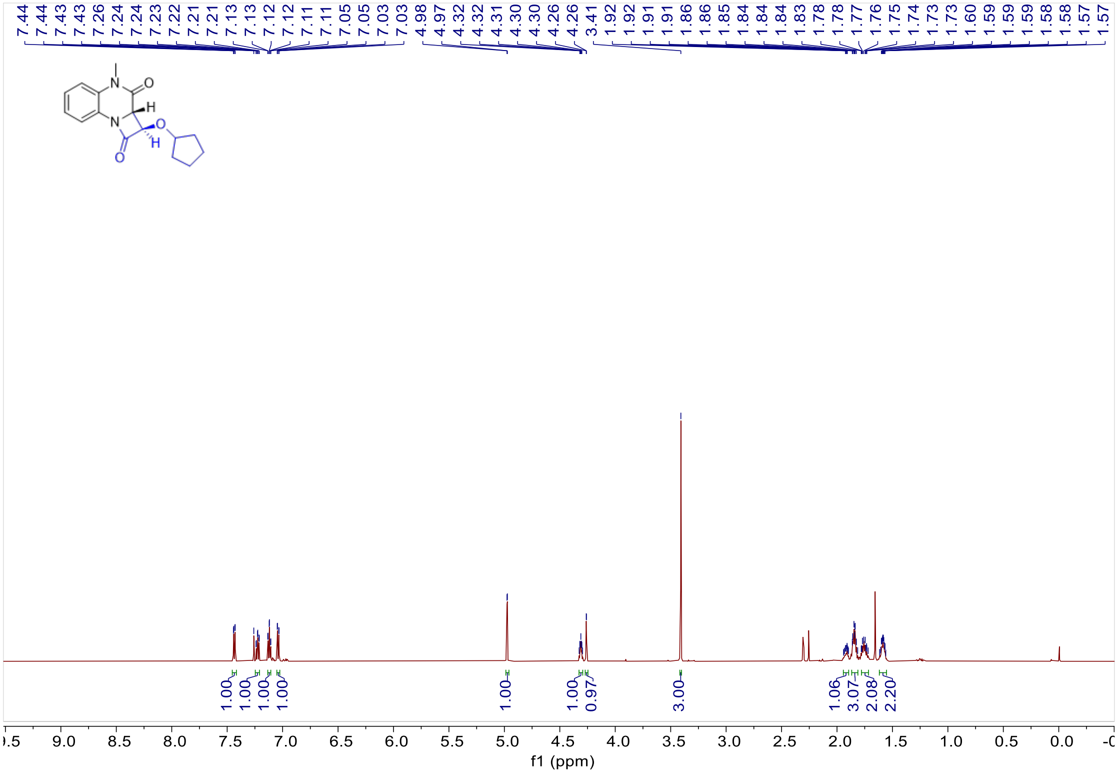

^{13}C NMR (150 MHz) Spectrum of 3e in $CDCl_3$

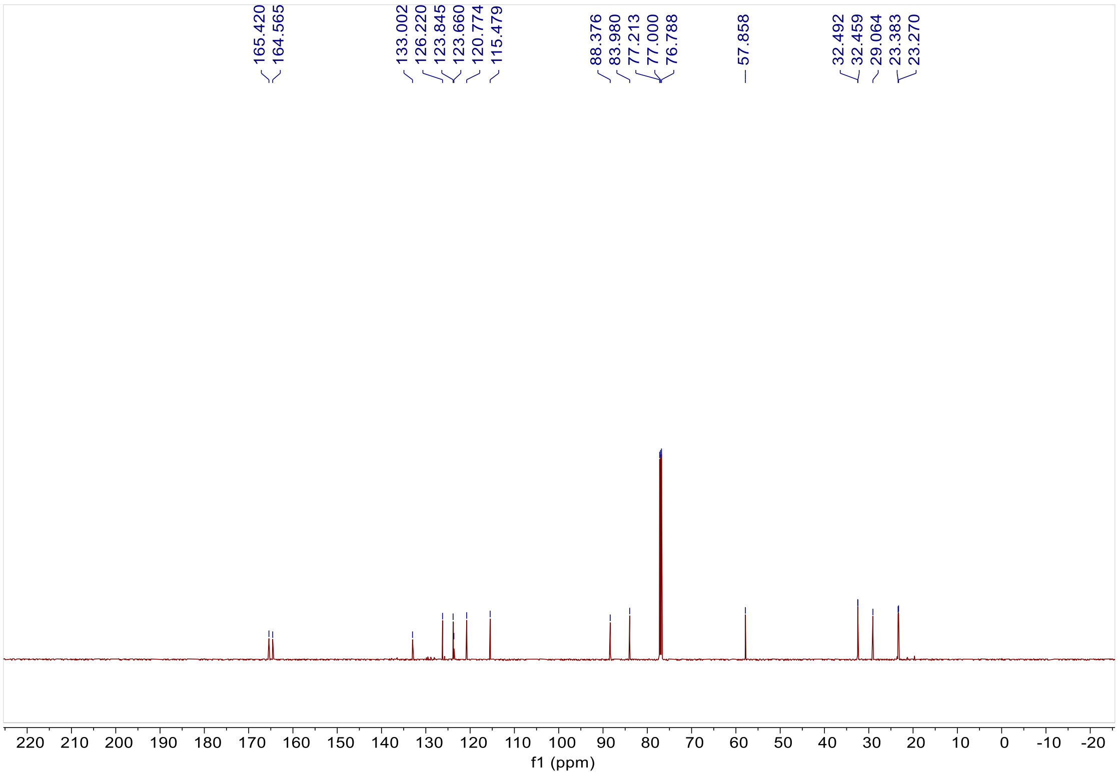

^{1}H NMR (400 MHz) Spectrum of 3f in CDCl$_3$

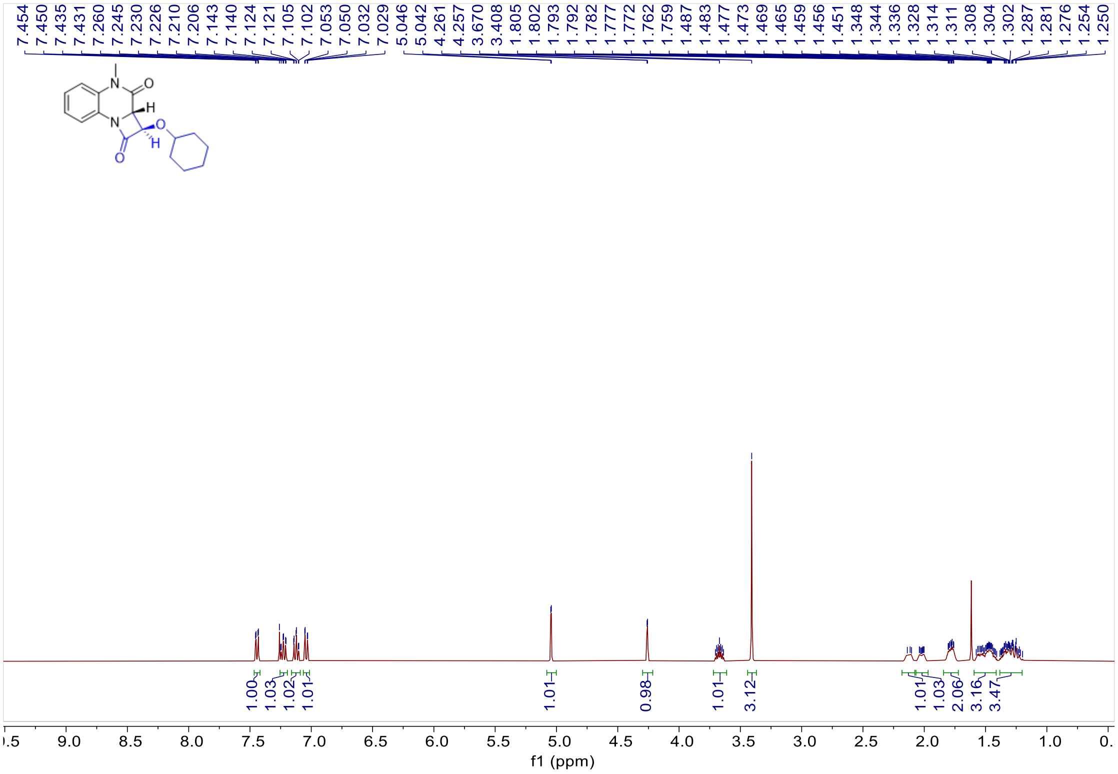

^{13}C NMR (100 MHz) Spectrum of 3f in CDCl$_3$

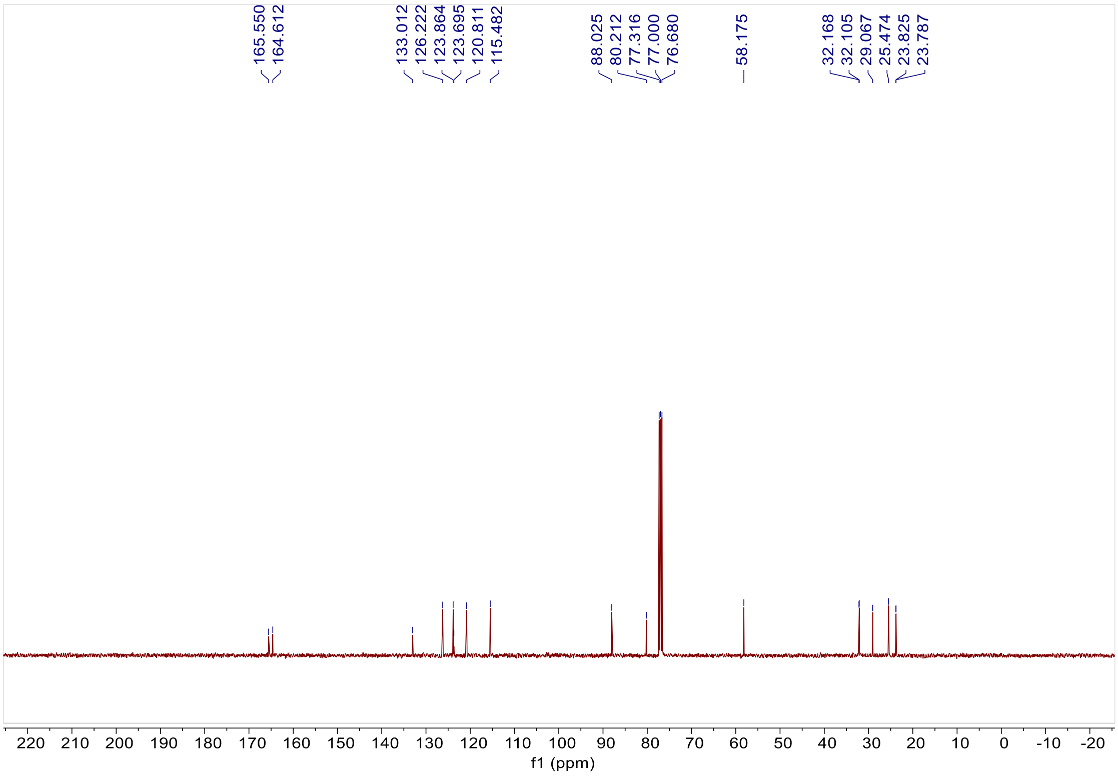

^{1}H NMR (400 MHz) Spectrum of 3g in $CDCl_3$

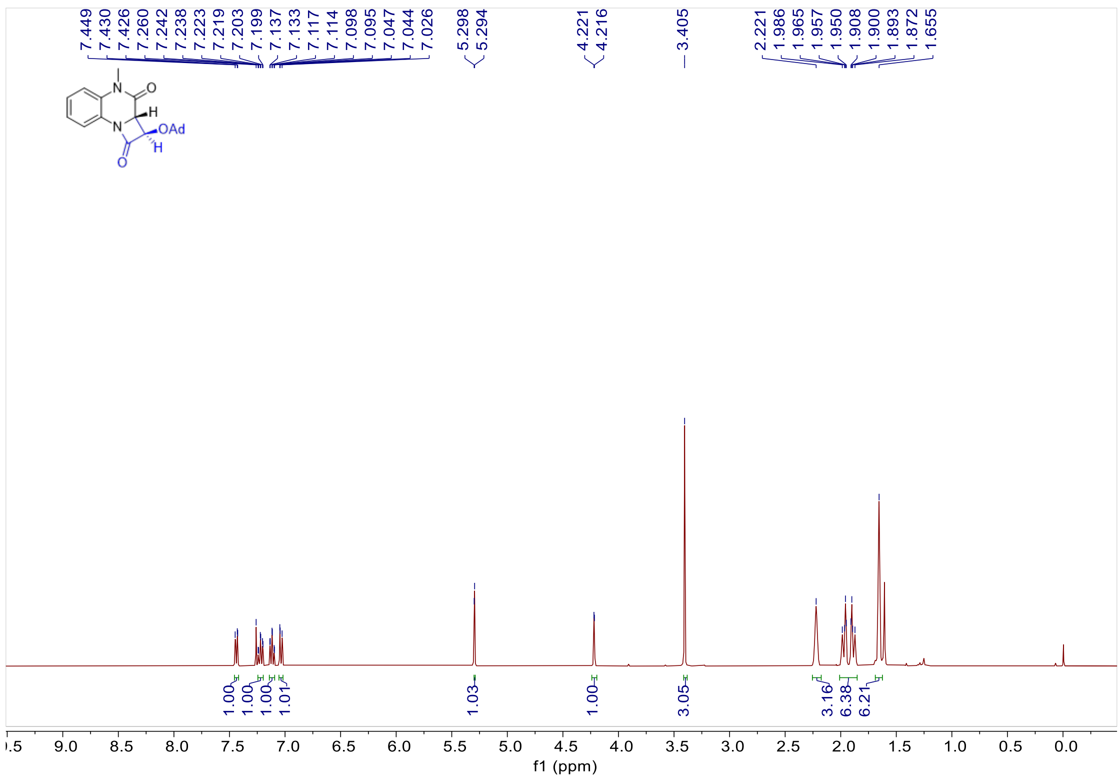

^{13}C NMR (100 MHz) Spectrum of 3g in $CDCl_3$

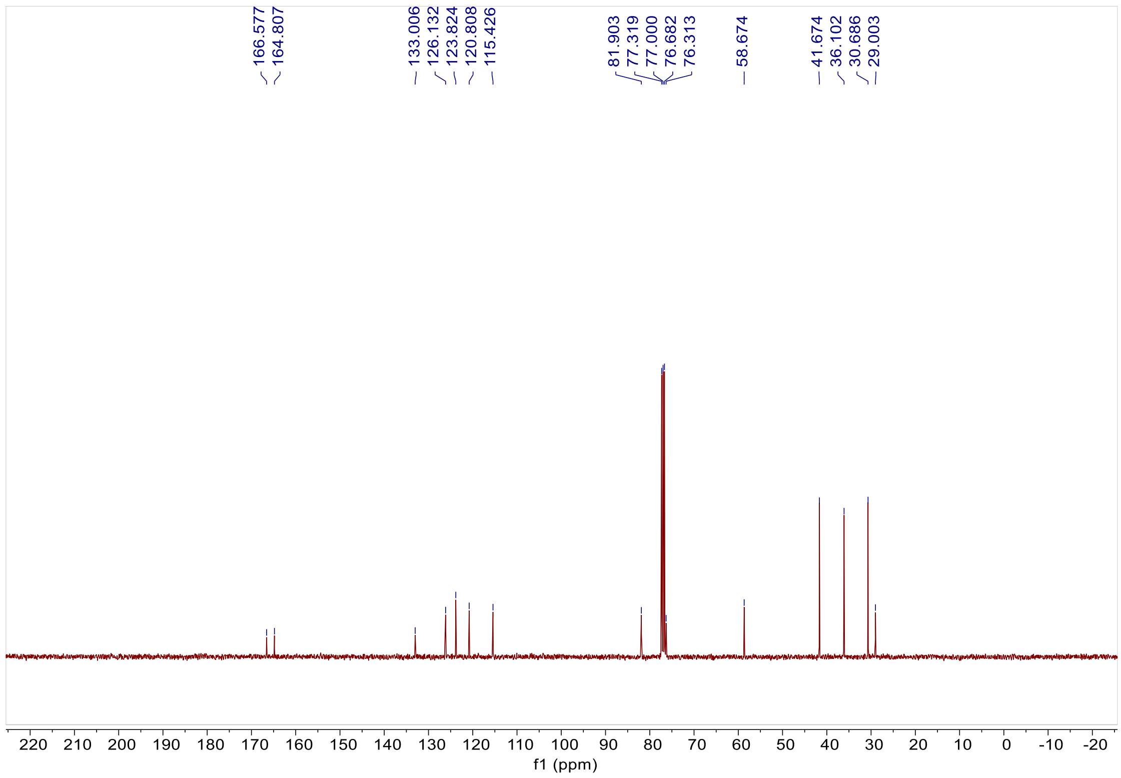

^{1}H NMR (600 MHz) Spectrum of 3h in $CDCl_3$

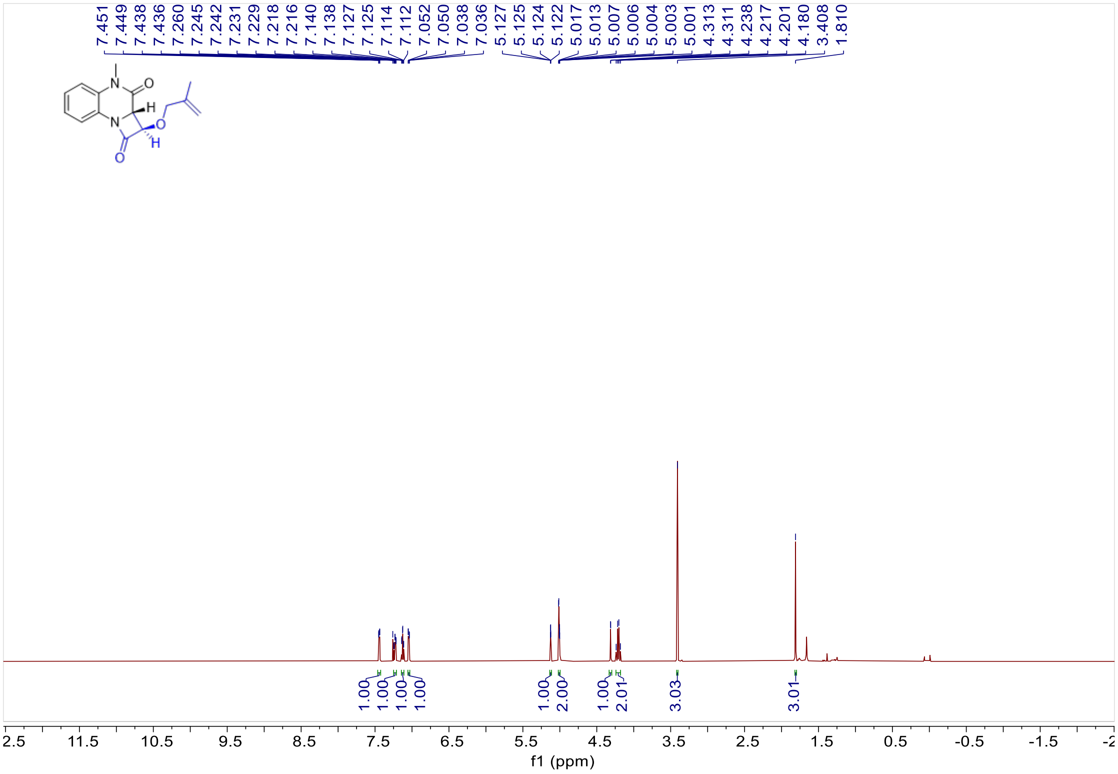

^{13}C NMR (150 MHz) Spectrum of 3h in $CDCl_3$

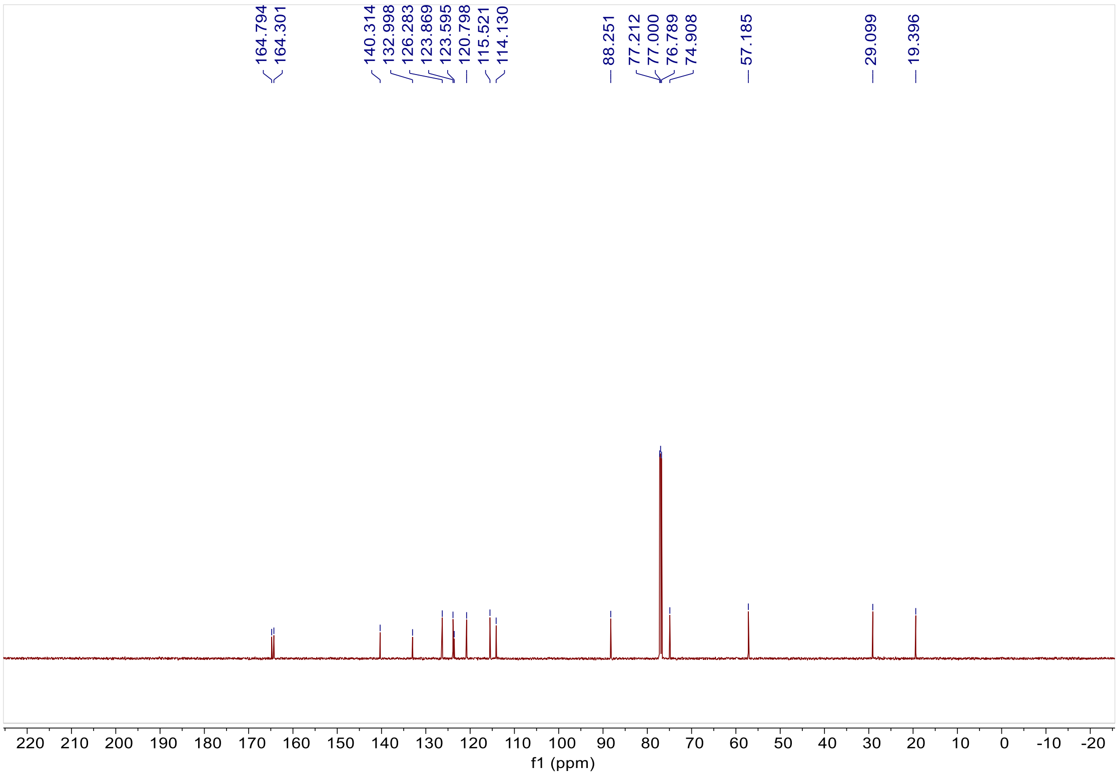

^{1}H NMR (400 MHz) Spectrum of 3i in $CDCl_3$

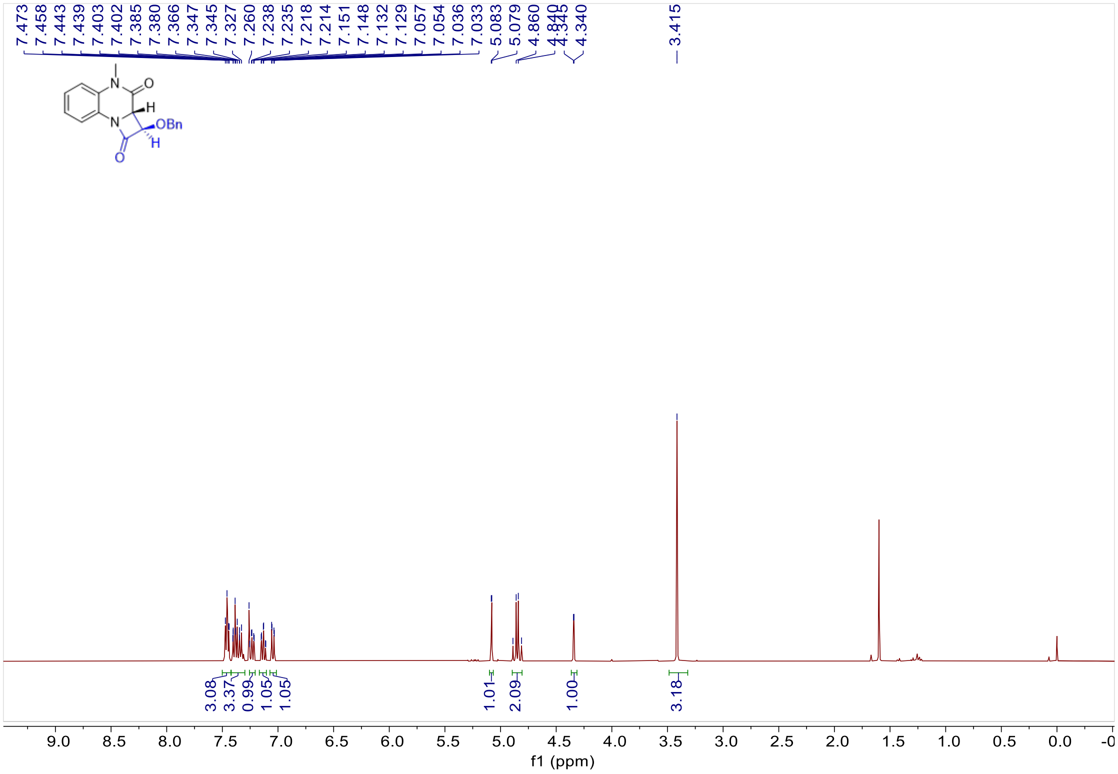

^{13}C NMR (100 MHz) Spectrum of 3i in $CDCl_3$

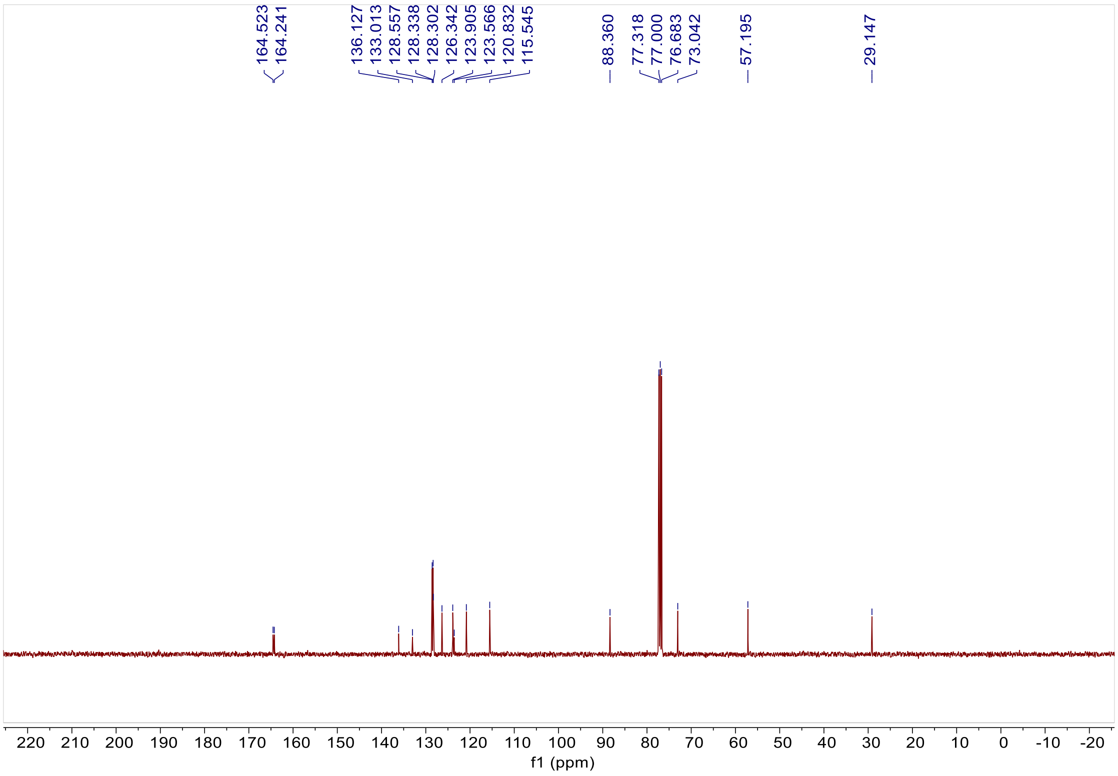

^{1}H NMR (400 MHz) Spectrum of 3j in CDCl$_3$

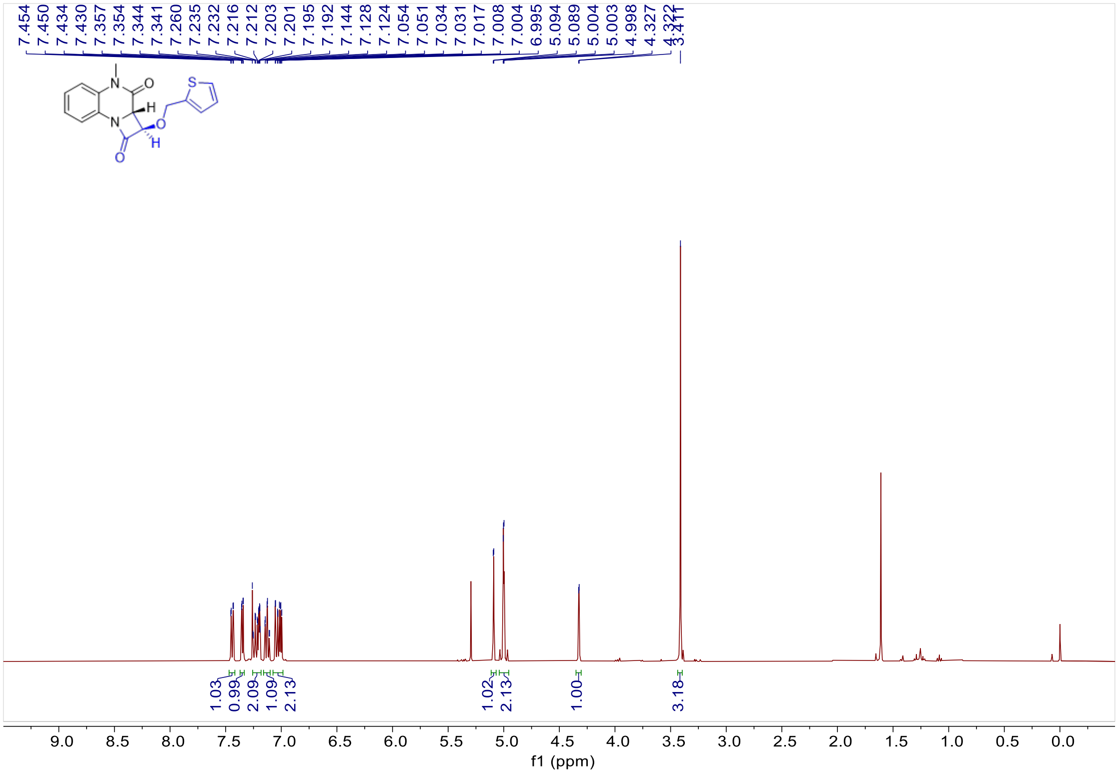

^{13}C NMR (100 MHz) Spectrum of 3j in CDCl$_3$

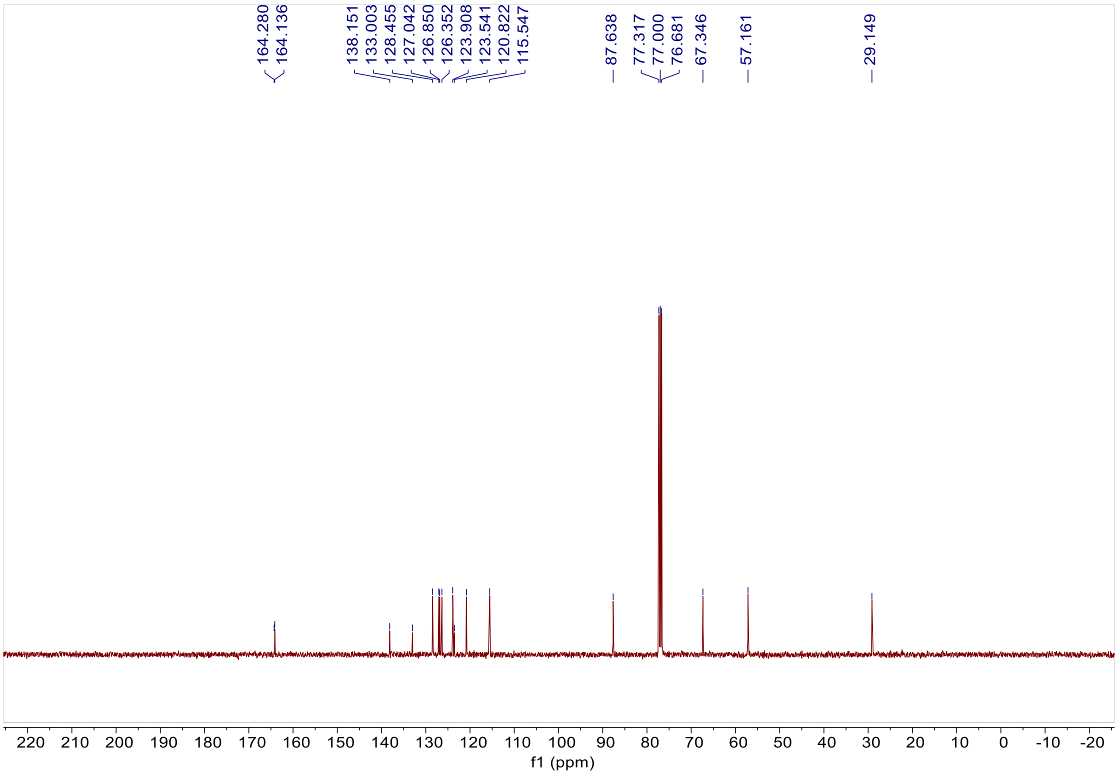

^{1}H NMR (400 MHz) Spectrum of 3k in CDCl$_3$

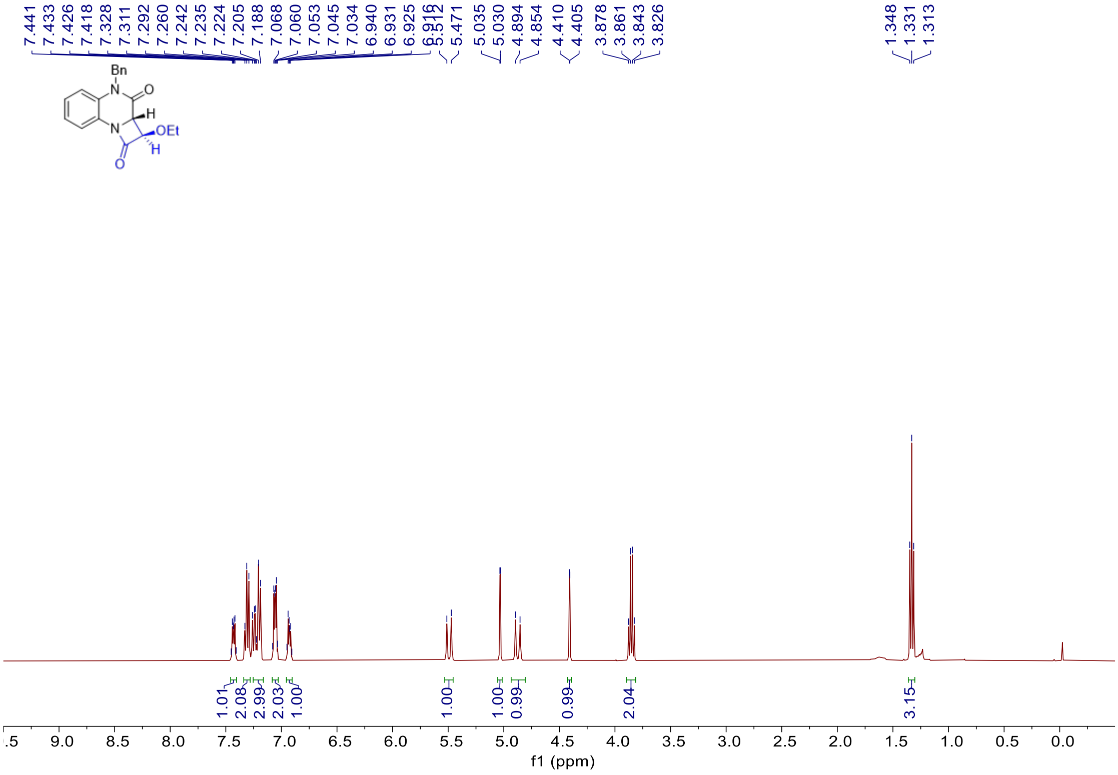

^{13}C NMR (100 MHz) Spectrum of 3k in CDCl$_3$

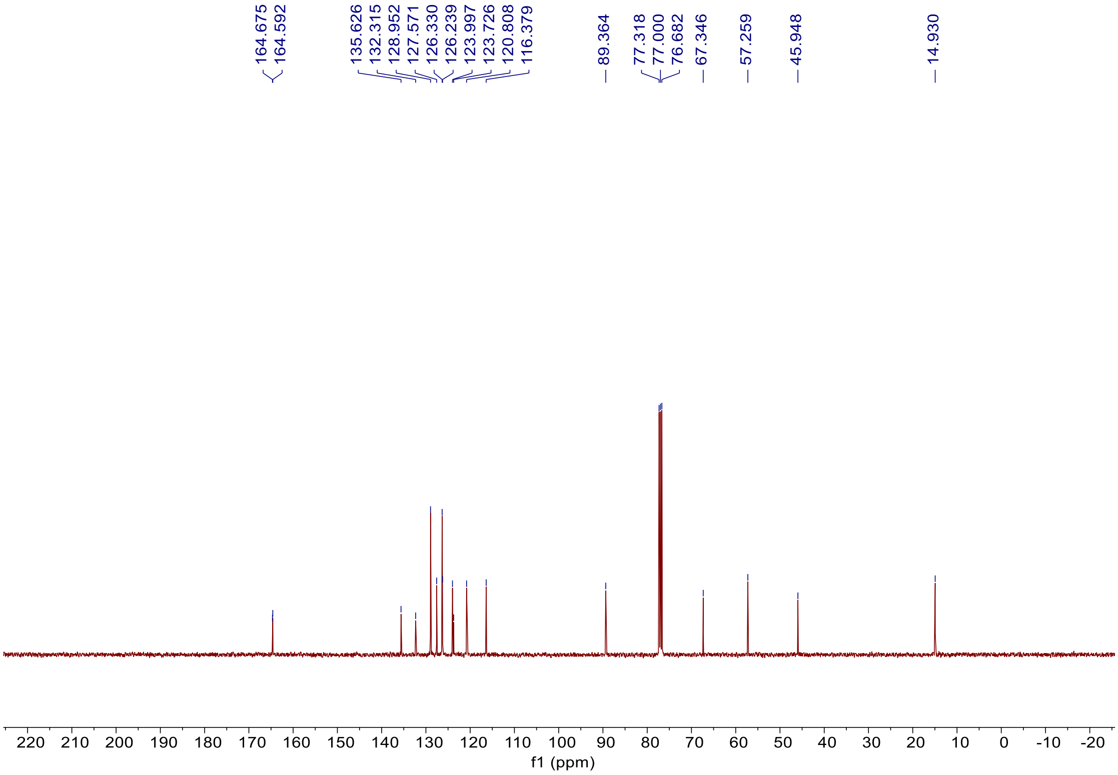

^{1}H NMR (600 MHz) Spectrum of 3l in CDCl$_3$

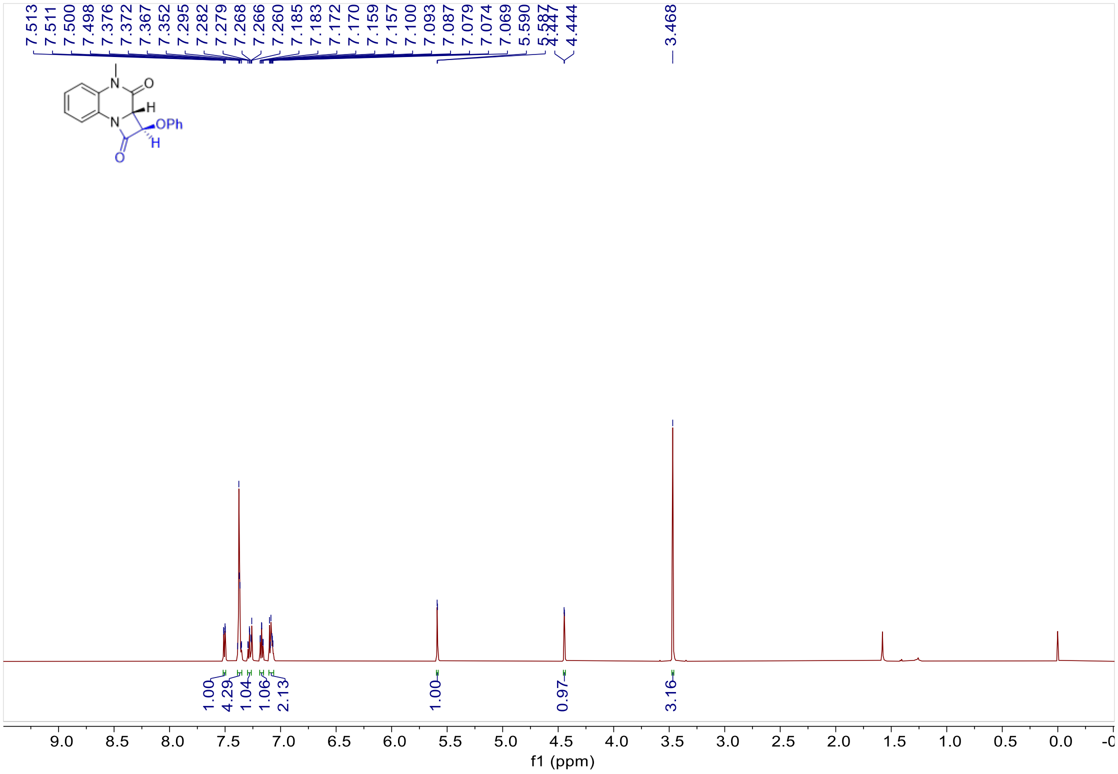

^{13}C NMR (150 MHz) Spectrum of 3l in CDCl$_3$

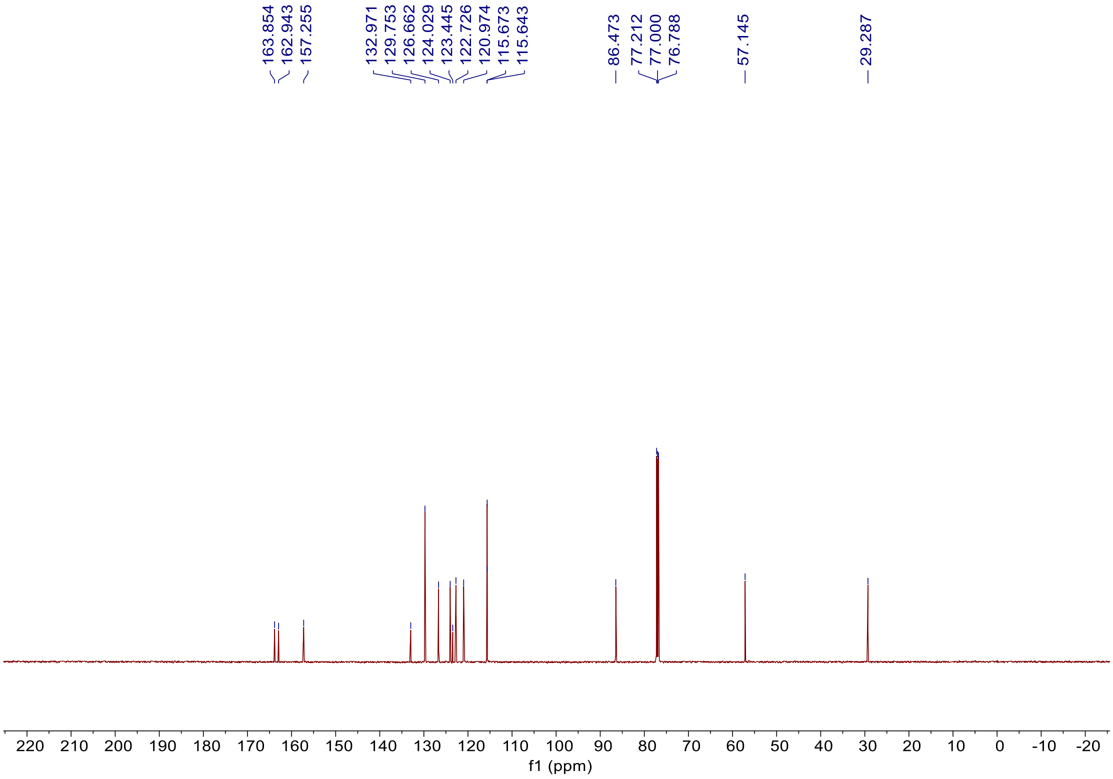

^{1}H NMR (400 MHz) Spectrum of 4a in $CDCl_3$

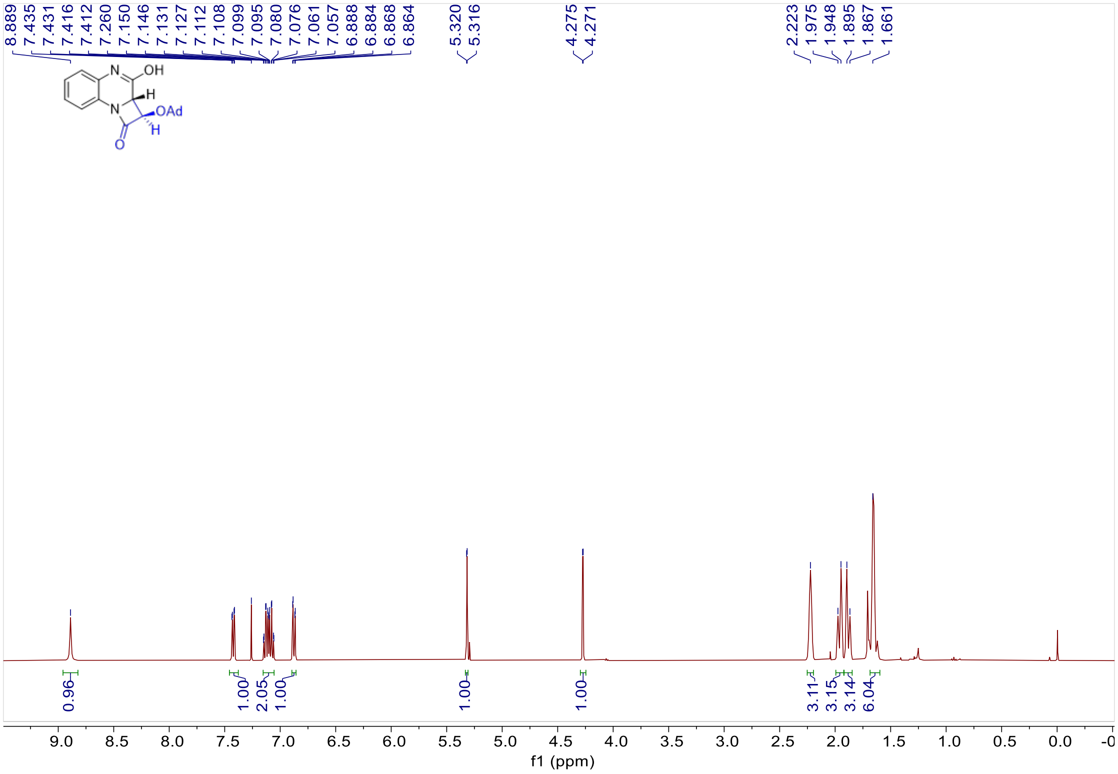

^{13}C NMR (100 MHz) Spectrum of 4a in $CDCl_3$

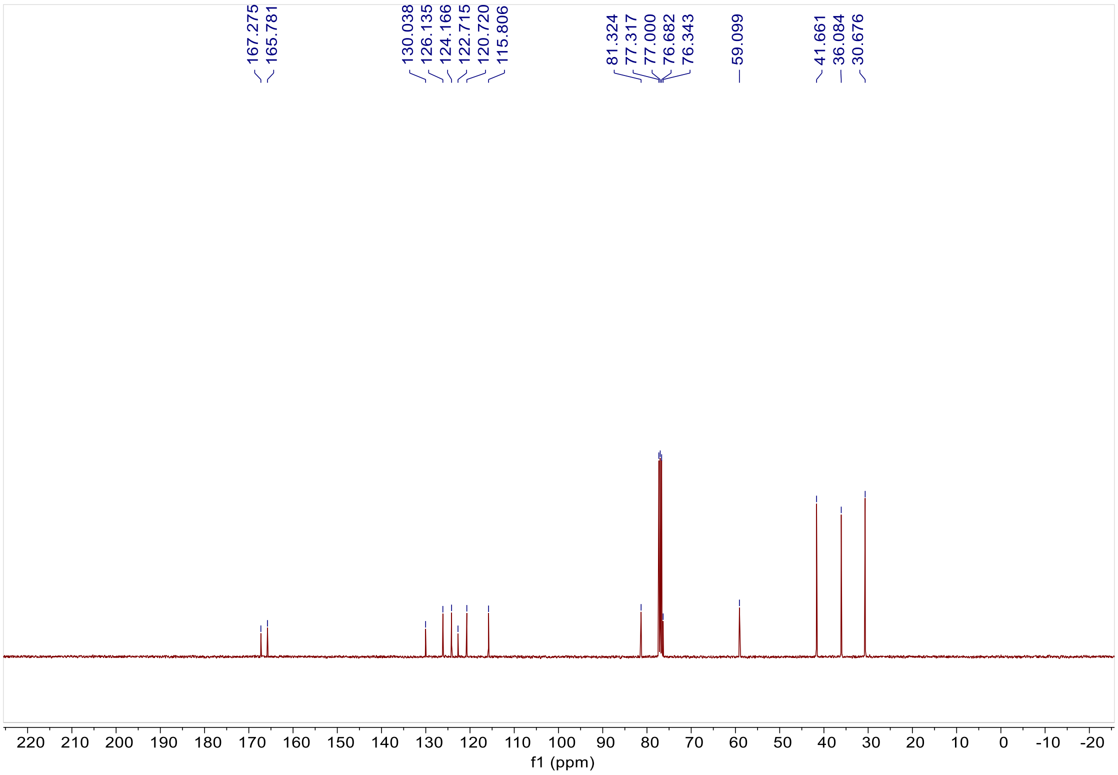

^{1}H NMR (400 MHz) Spectrum of 4b in $CDCl_3$

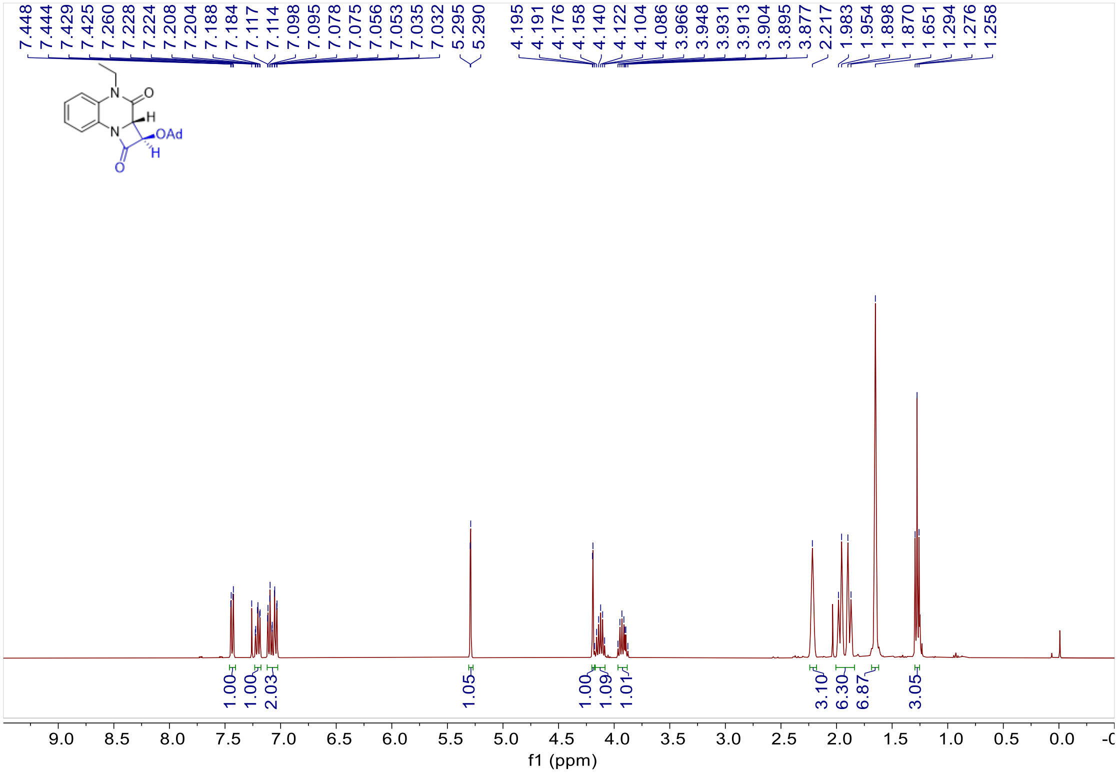

^{13}C NMR (100 MHz) Spectrum of 4b in $CDCl_3$

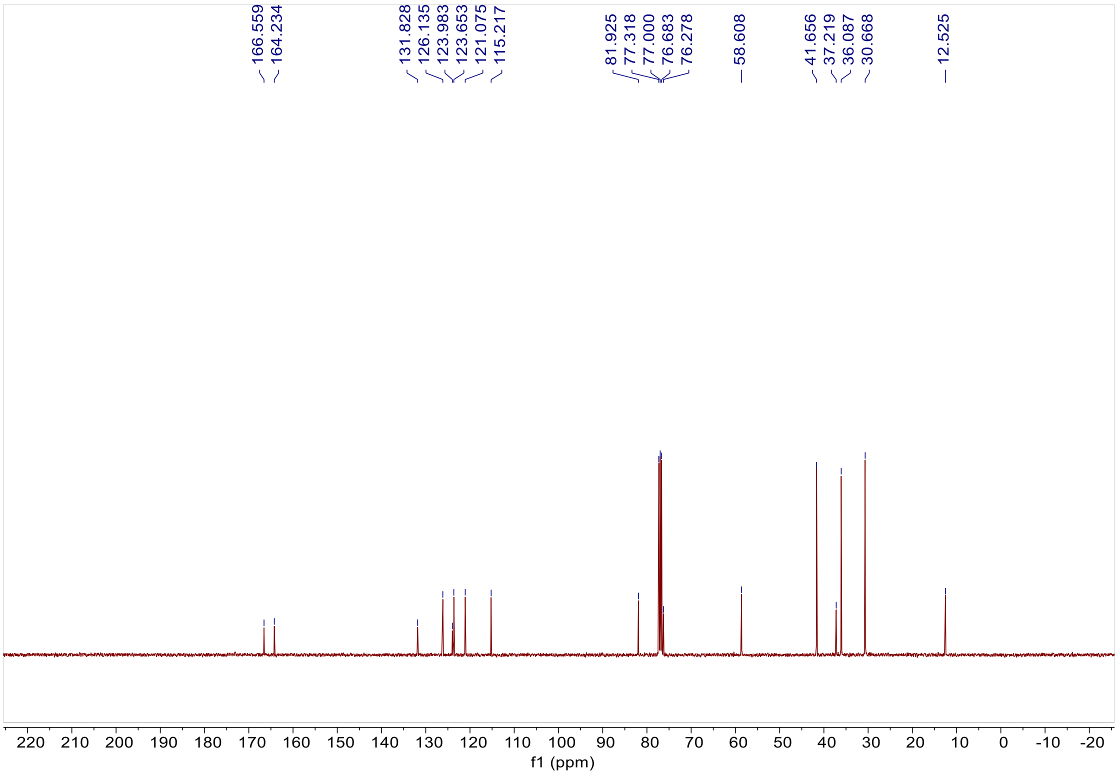

^{1}H NMR (600 MHz) Spectrum of 4c in CDCl$_3$

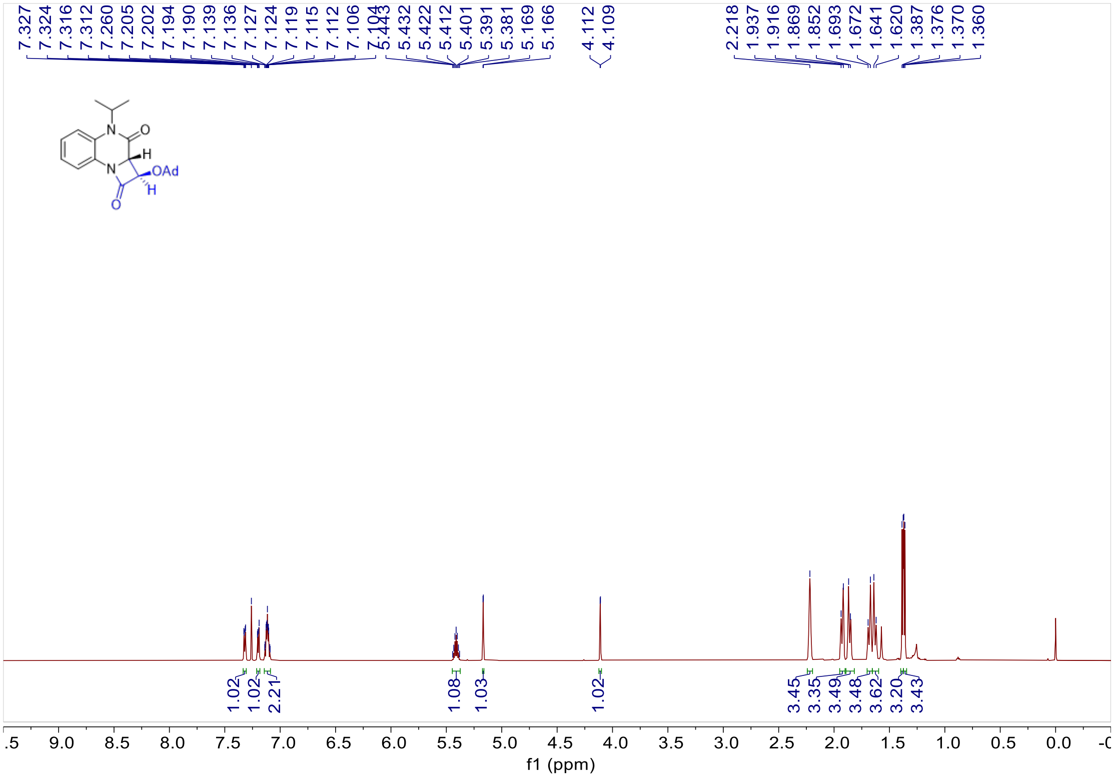

^{13}C NMR (150 MHz) Spectrum of 4c in CDCl$_3$

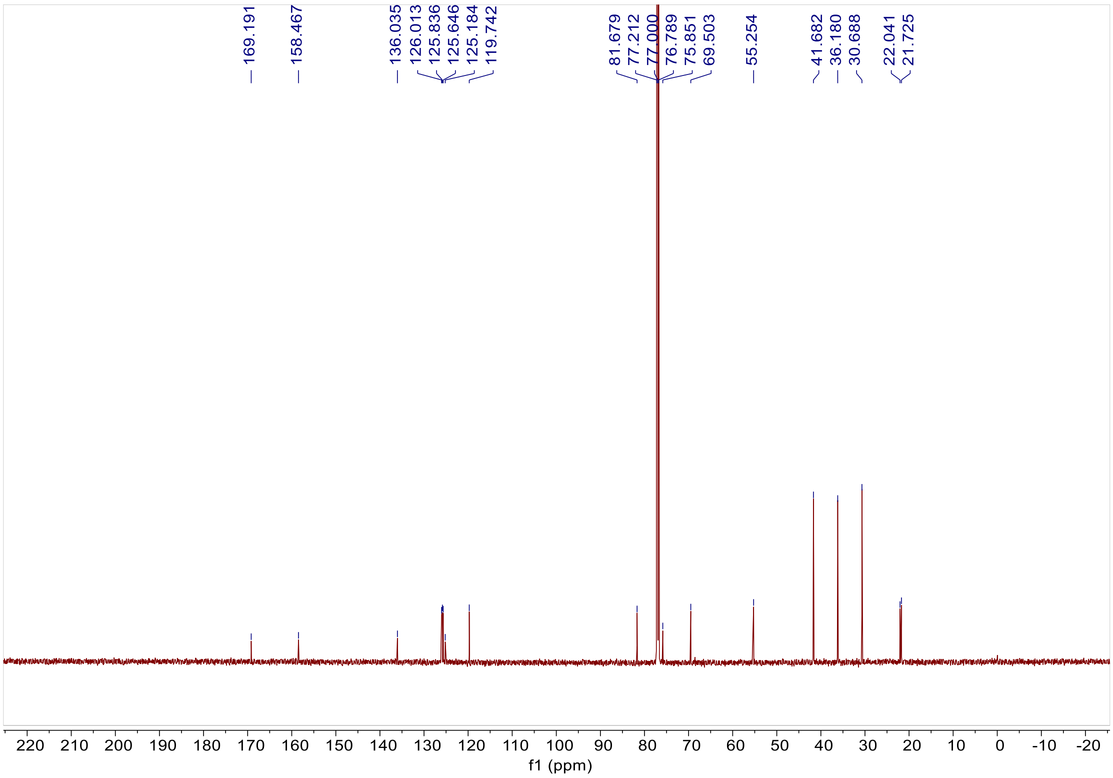

^{1}H NMR (400 MHz) Spectrum of 4d in $CDCl_3$

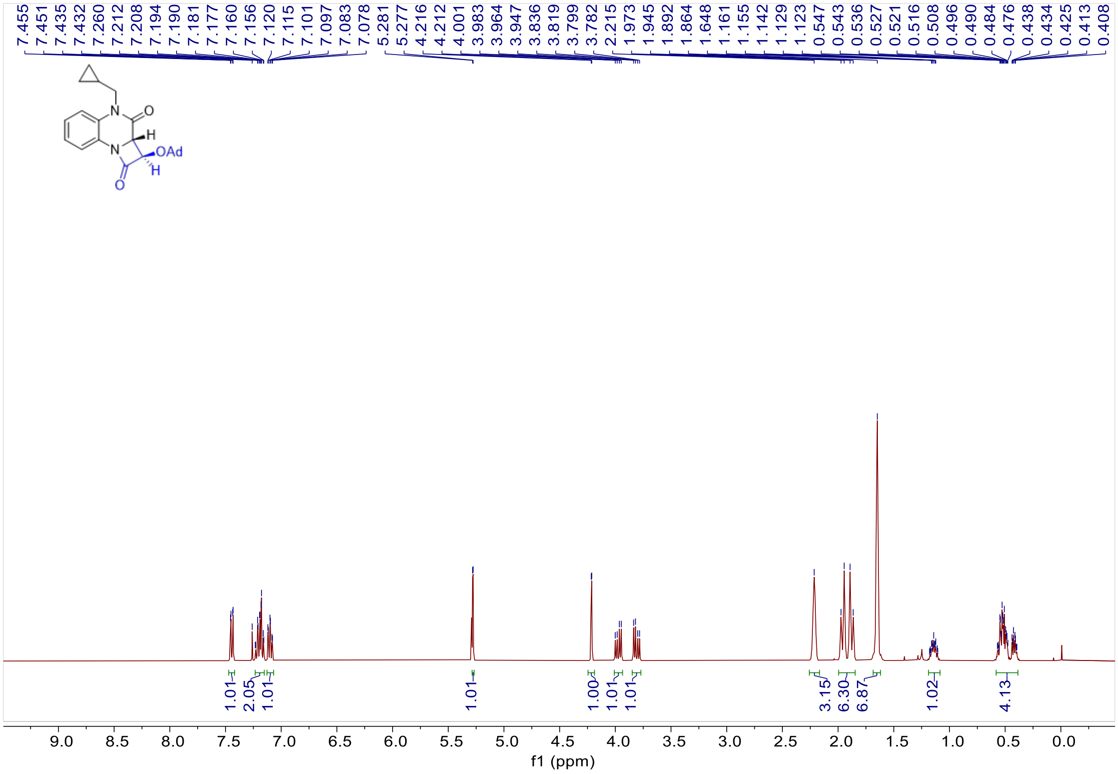

^{13}C NMR (100 MHz) Spectrum of 4d in $CDCl_3$

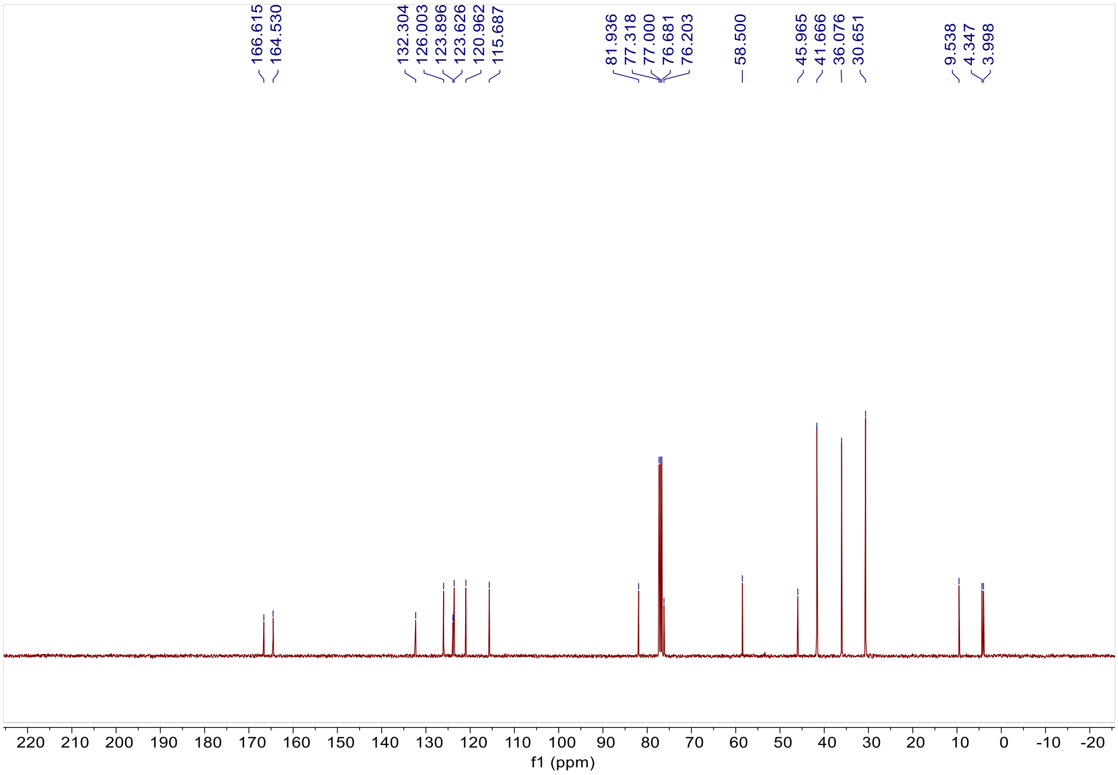

^{1}H NMR (400 MHz) Spectrum of 4e in $CDCl_3$

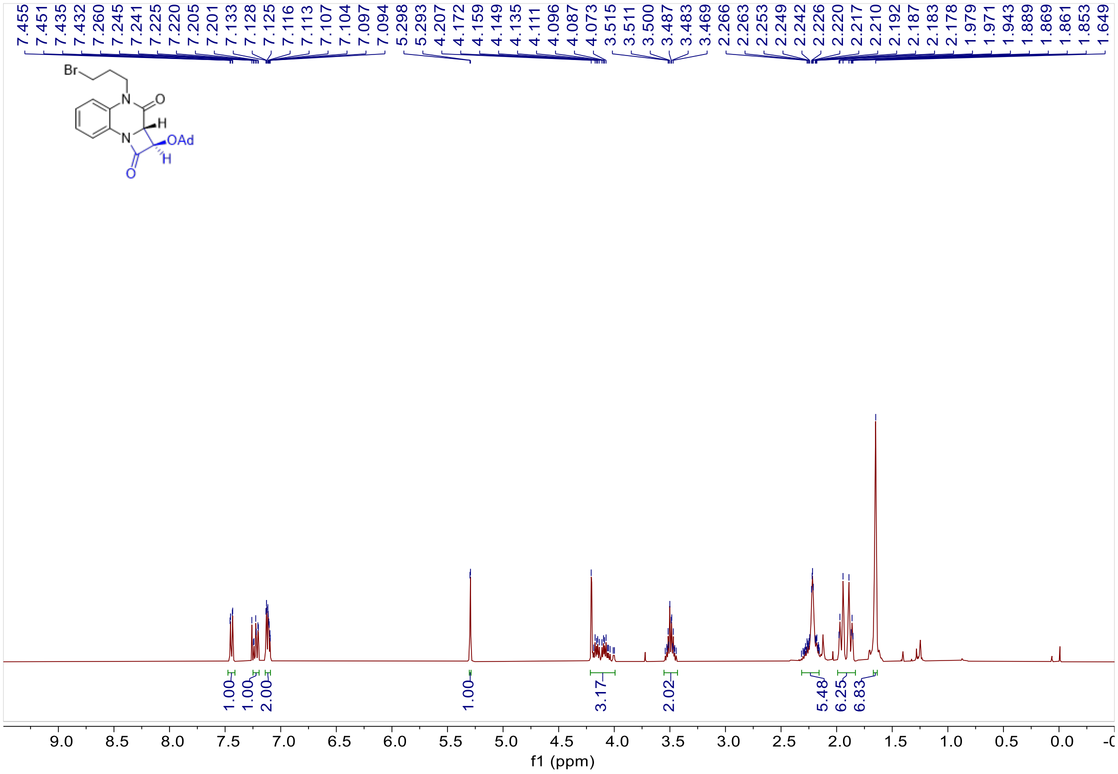

^{13}C NMR (100 MHz) Spectrum of 4e in $CDCl_3$

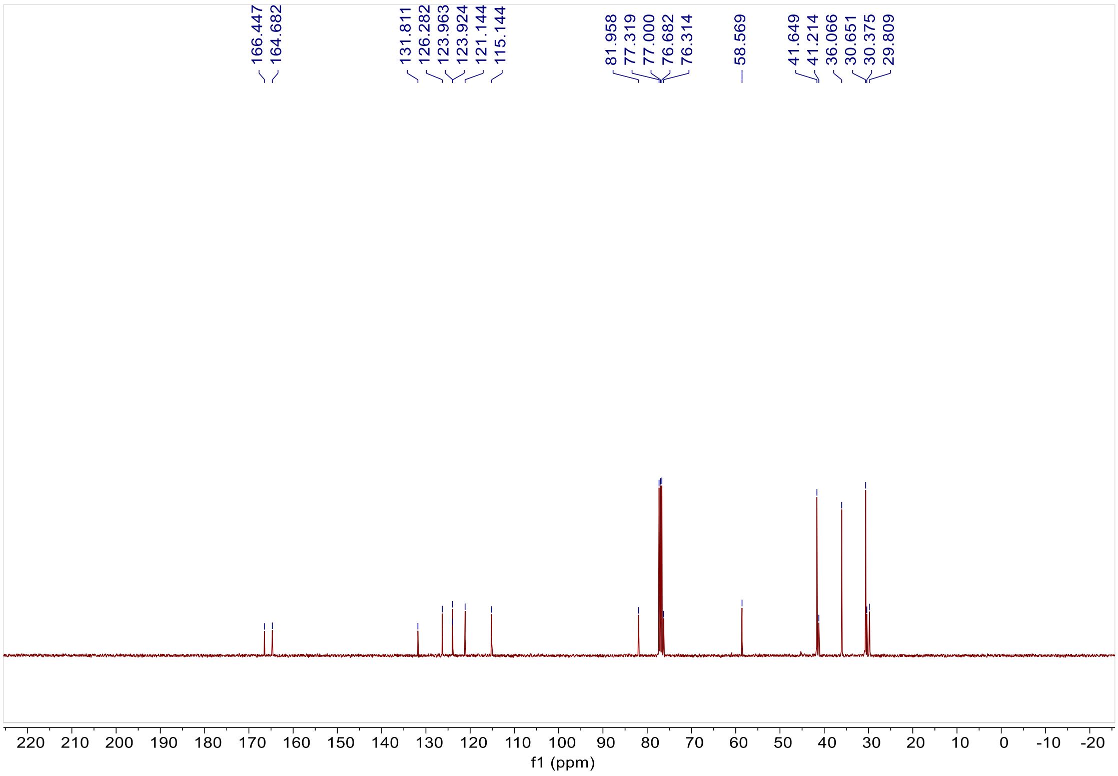

^{1}H NMR (400 MHz) Spectrum of 4f in $CDCl_3$

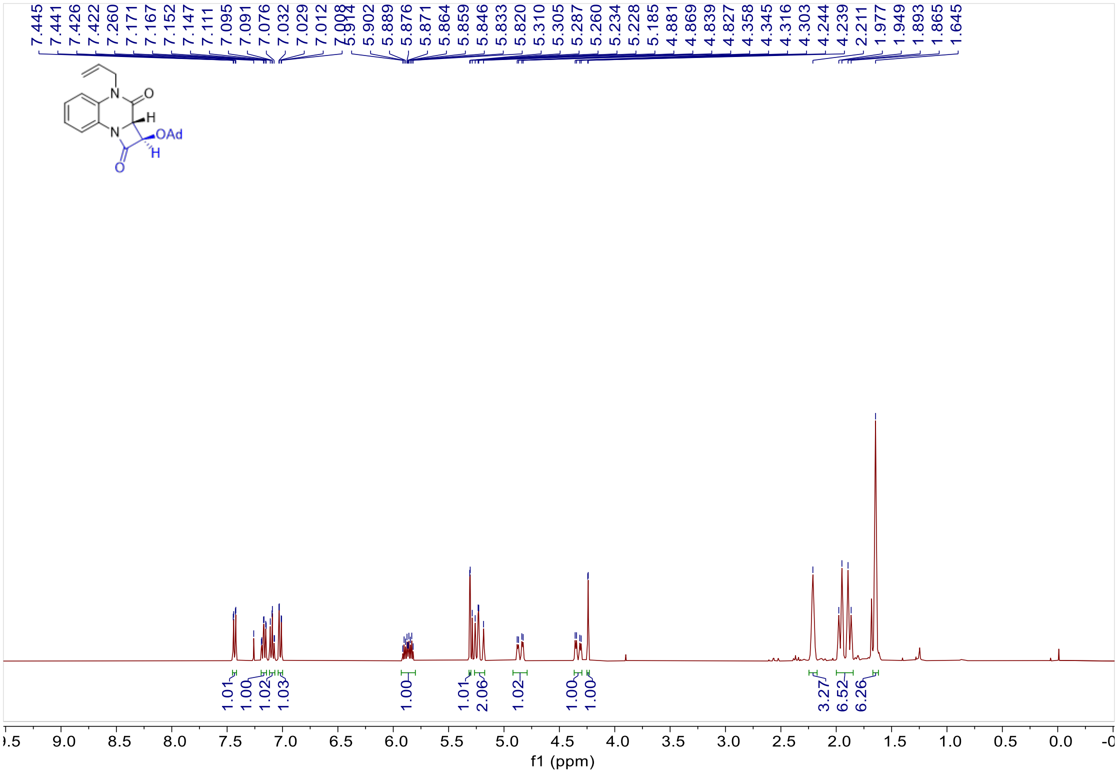

^{13}C NMR (100 MHz) Spectrum of 4f in $CDCl_3$

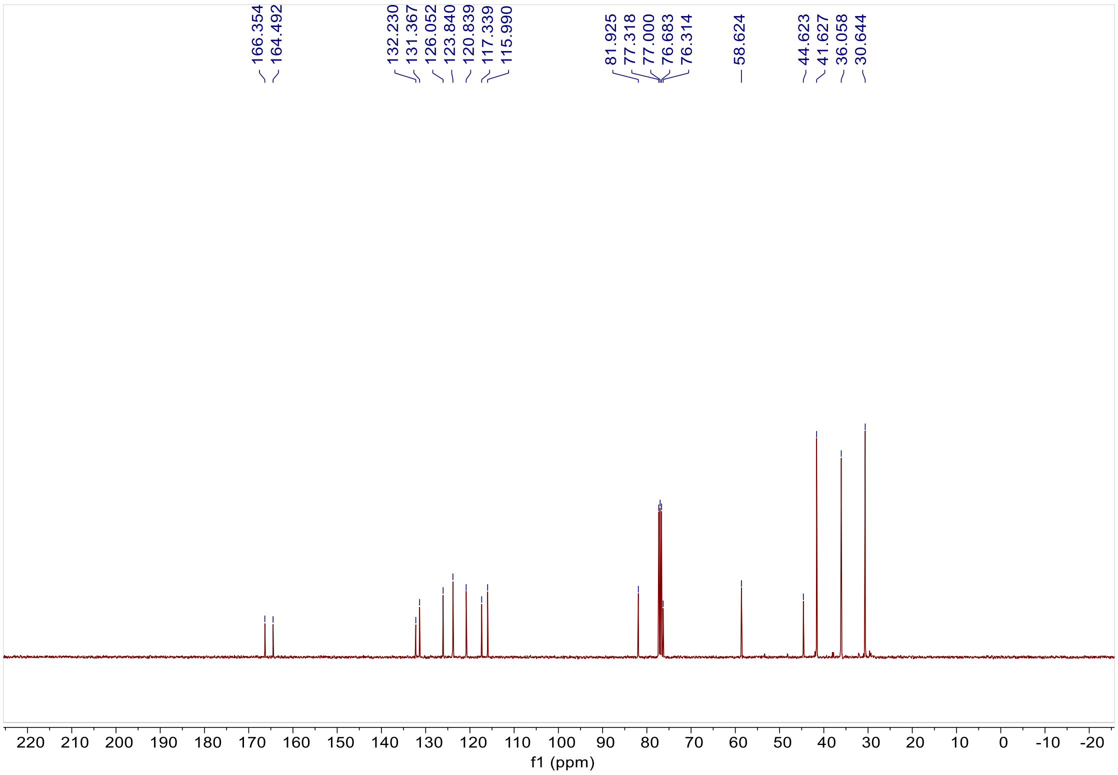

^{1}H NMR (400 MHz) Spectrum of 4g in $CDCl_3$

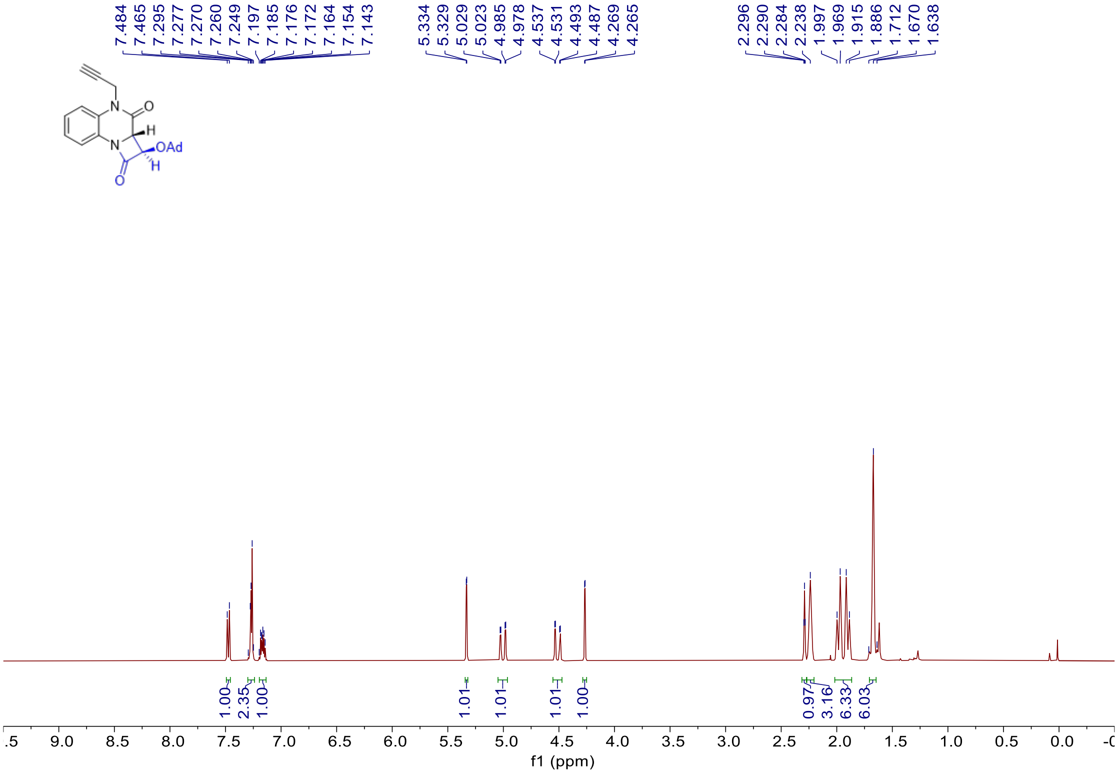

^{13}C NMR (100 MHz) Spectrum of 4g in $CDCl_3$

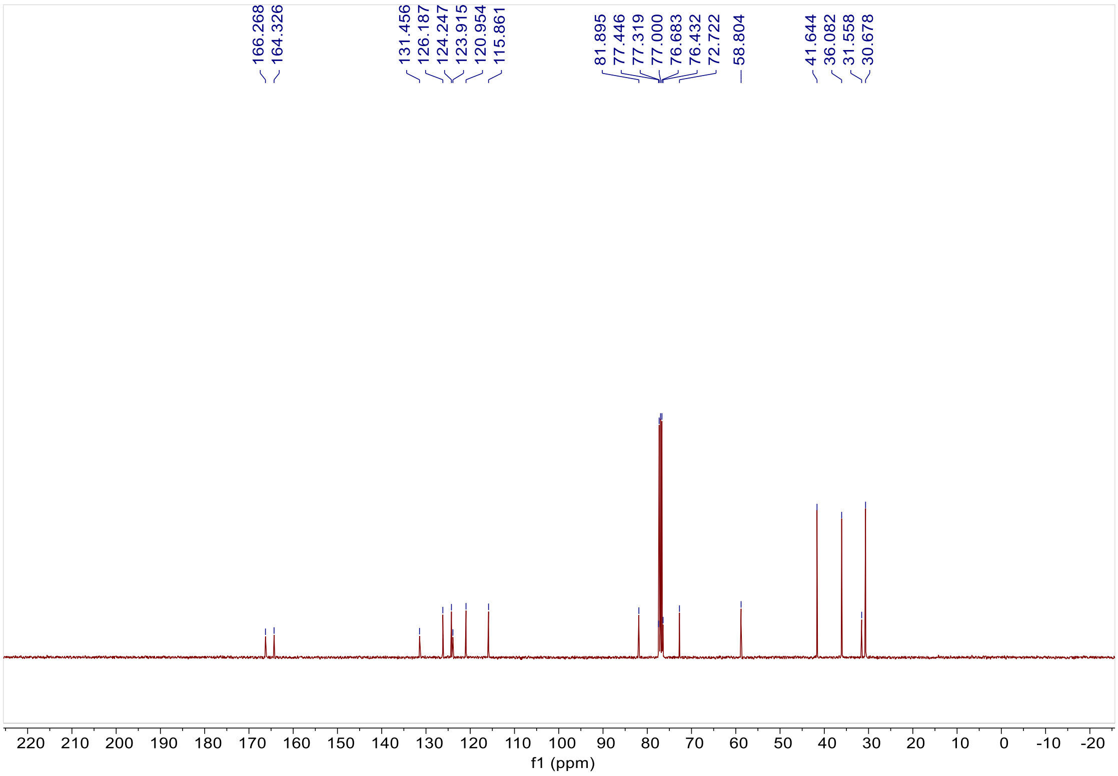

^{1}H NMR (400 MHz) Spectrum of 4h in $CDCl_3$

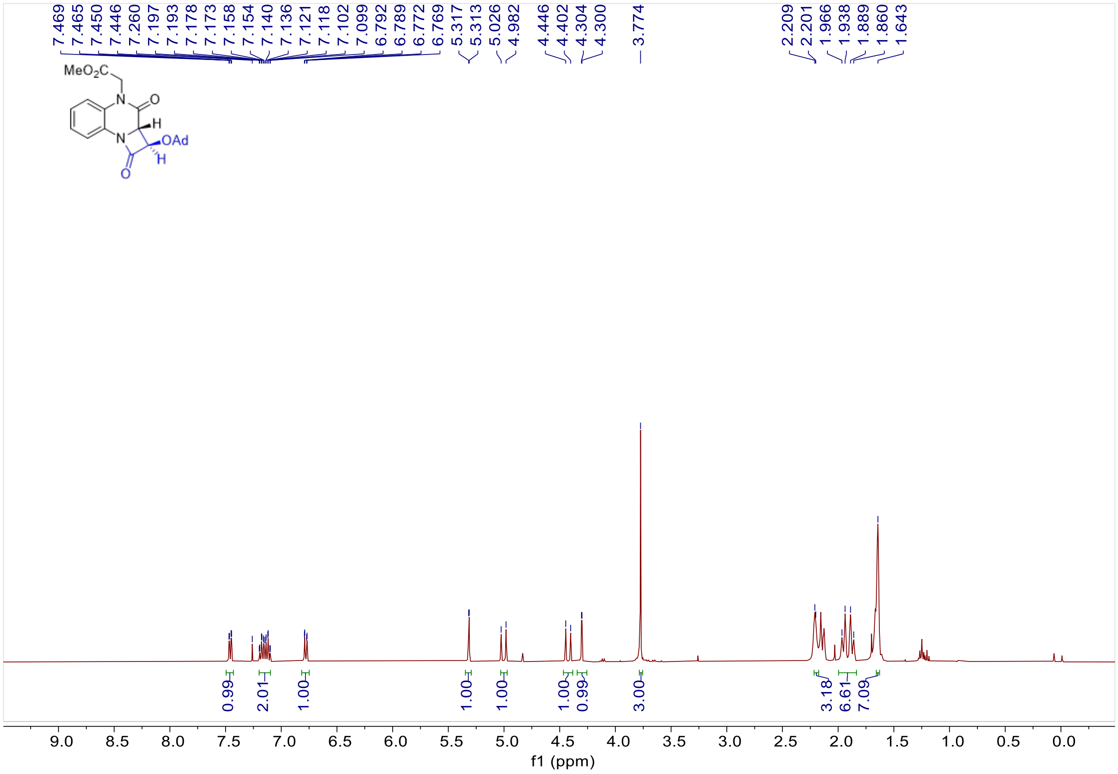

^{13}C NMR (100 MHz) Spectrum of 4h in $CDCl_3$

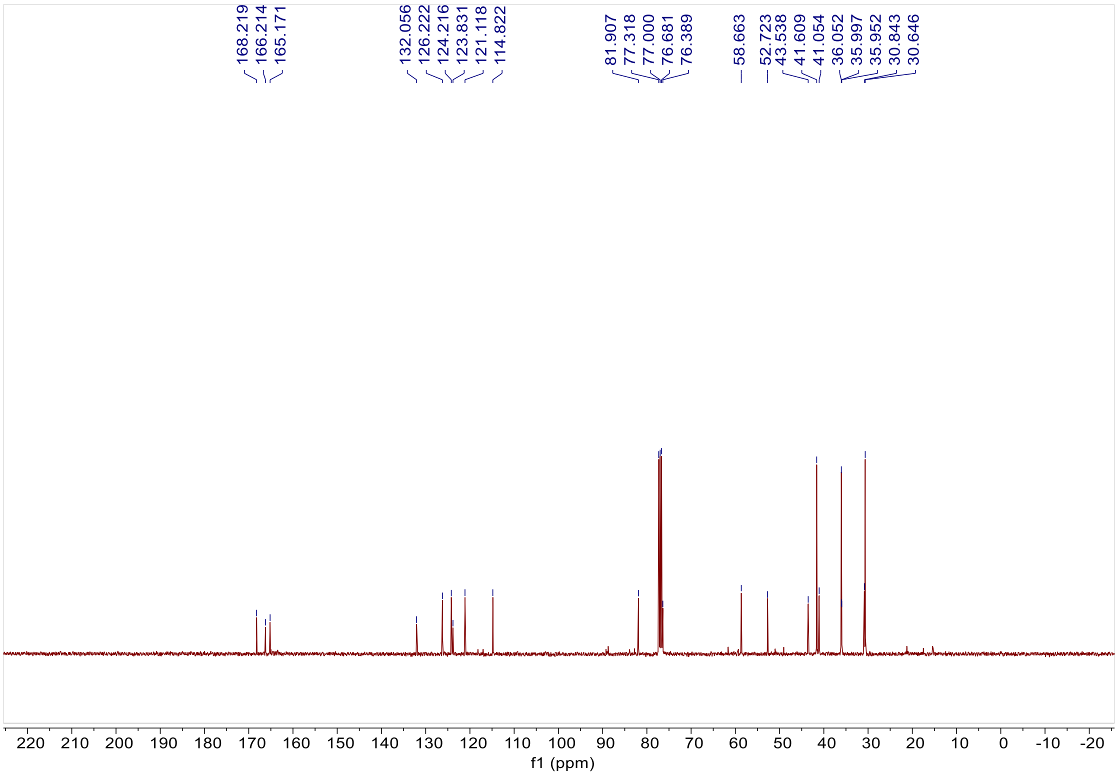

^{1}H NMR (400 MHz) Spectrum of 4i in $CDCl_3$

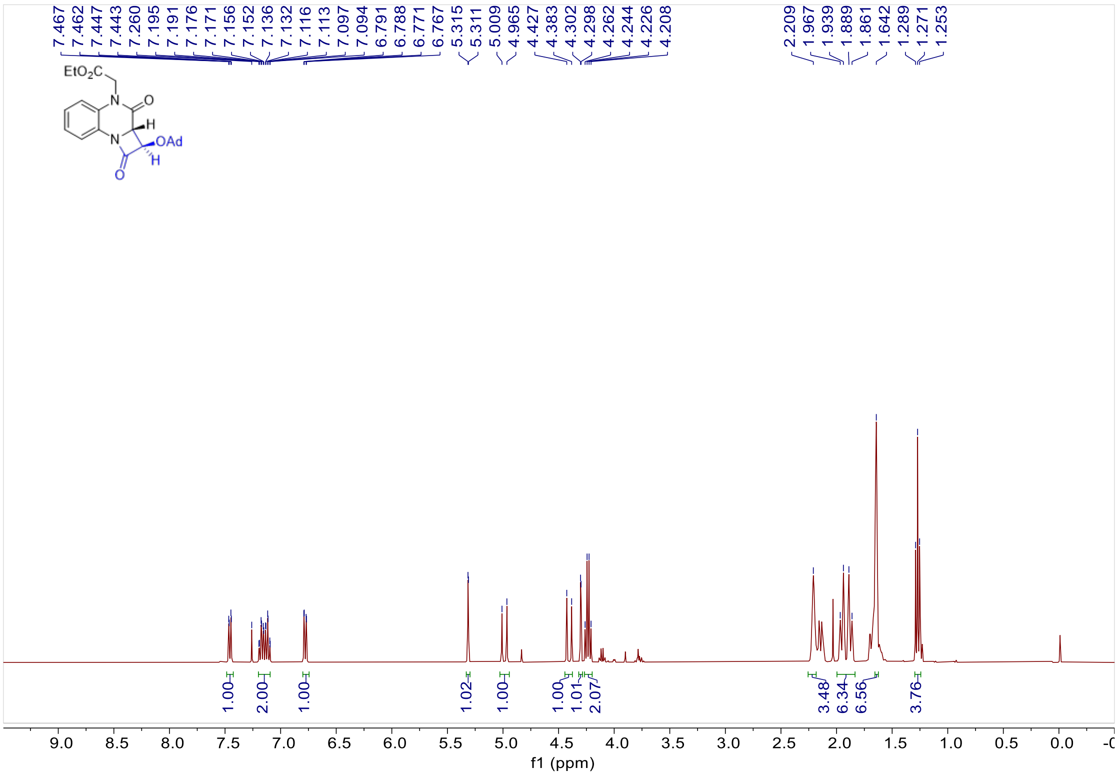

^{13}C NMR (100 MHz) Spectrum of 4i in $CDCl_3$

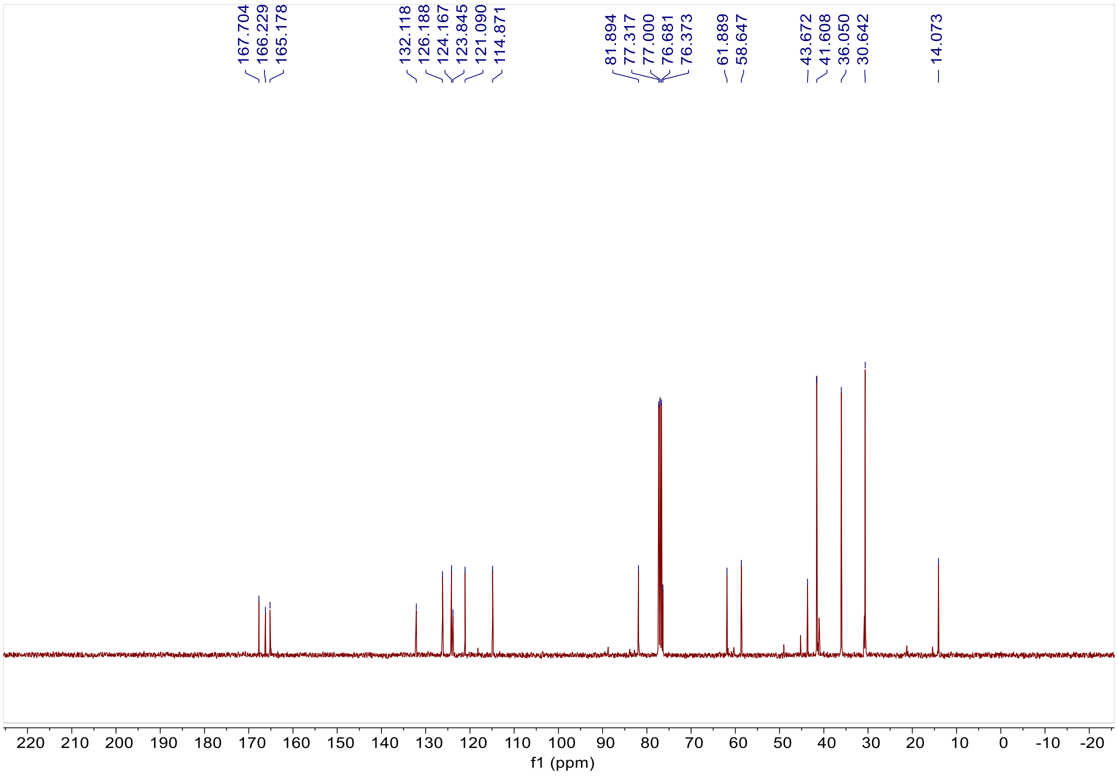

^{1}H NMR (400 MHz) Spectrum of 4j in $CDCl_3$

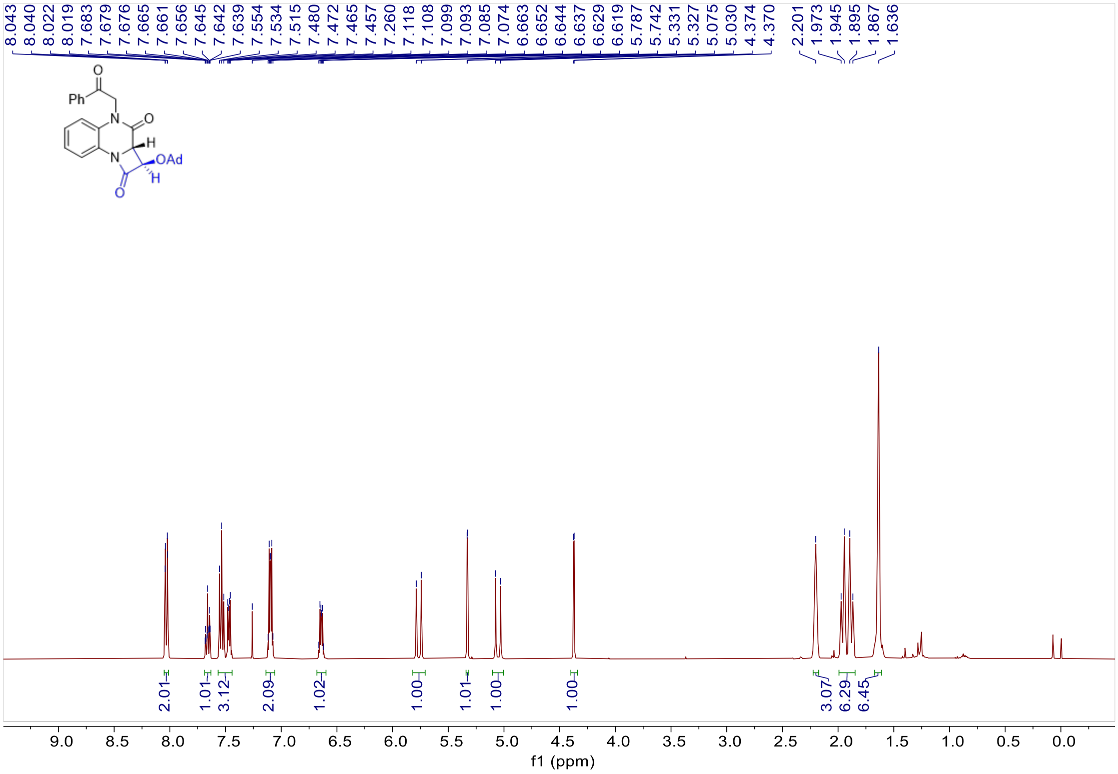

^{13}C NMR (100 MHz) Spectrum of 4j in $CDCl_3$

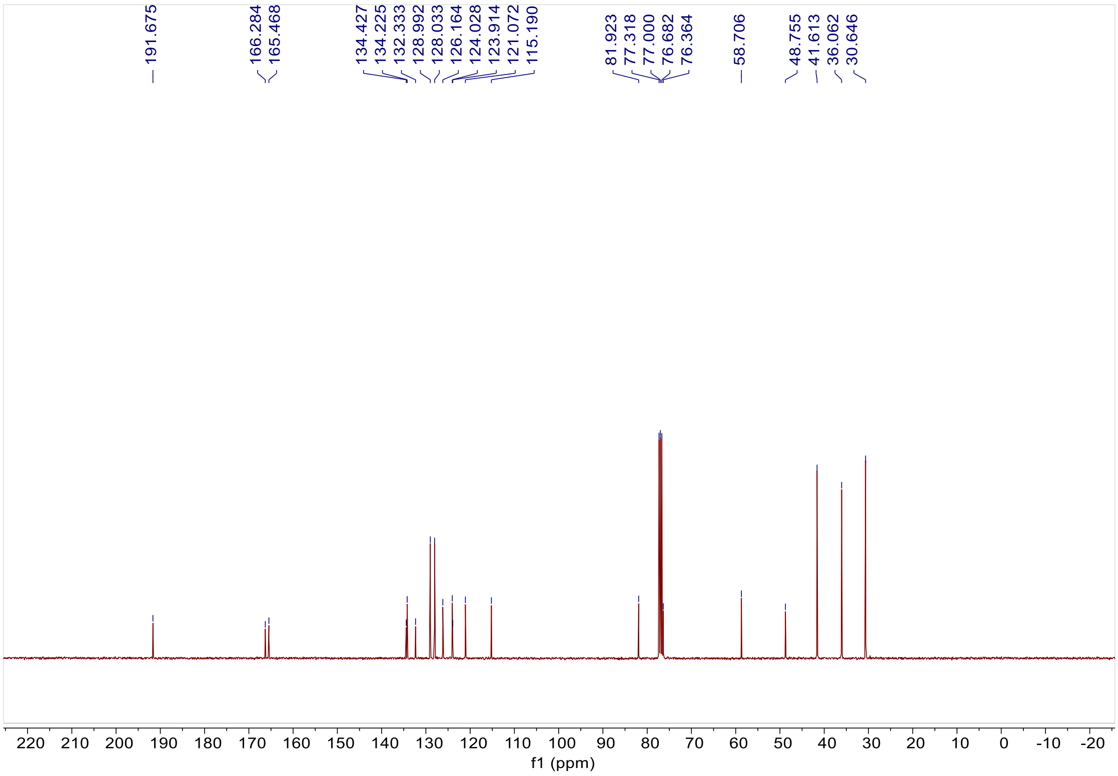

^{1}H NMR (400 MHz) Spectrum of 4k in $CDCl_3$

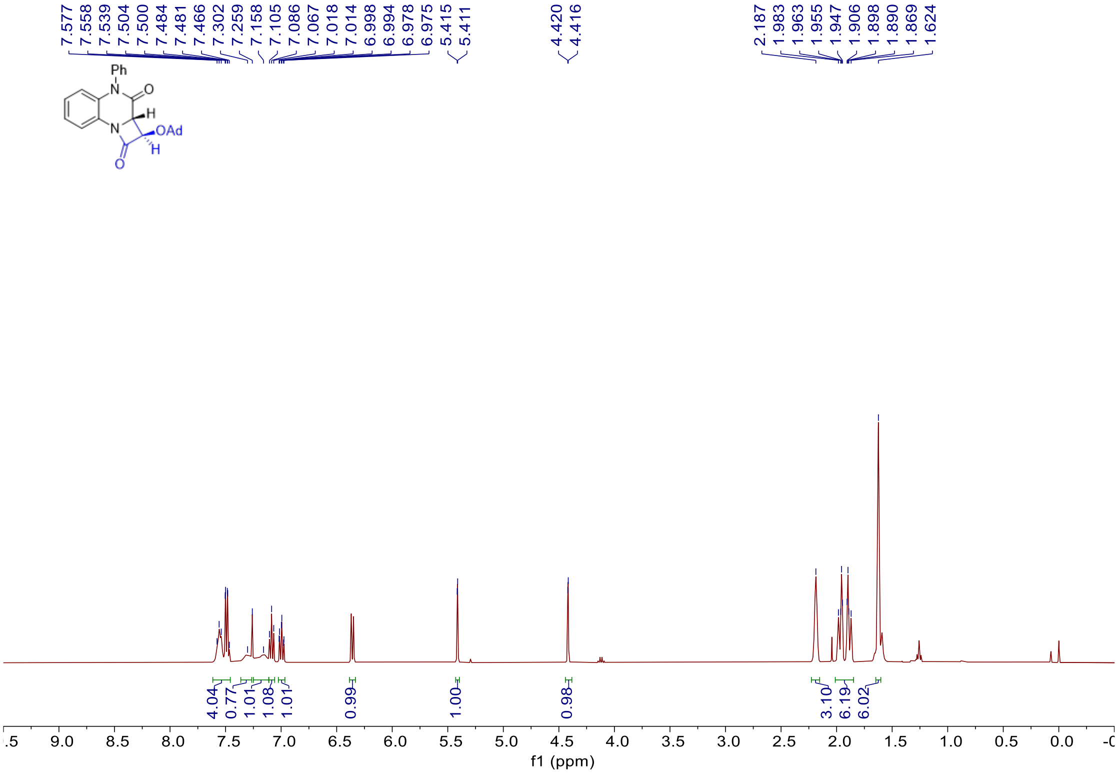

^{13}C NMR (100 MHz) Spectrum of 4k in $CDCl_3$

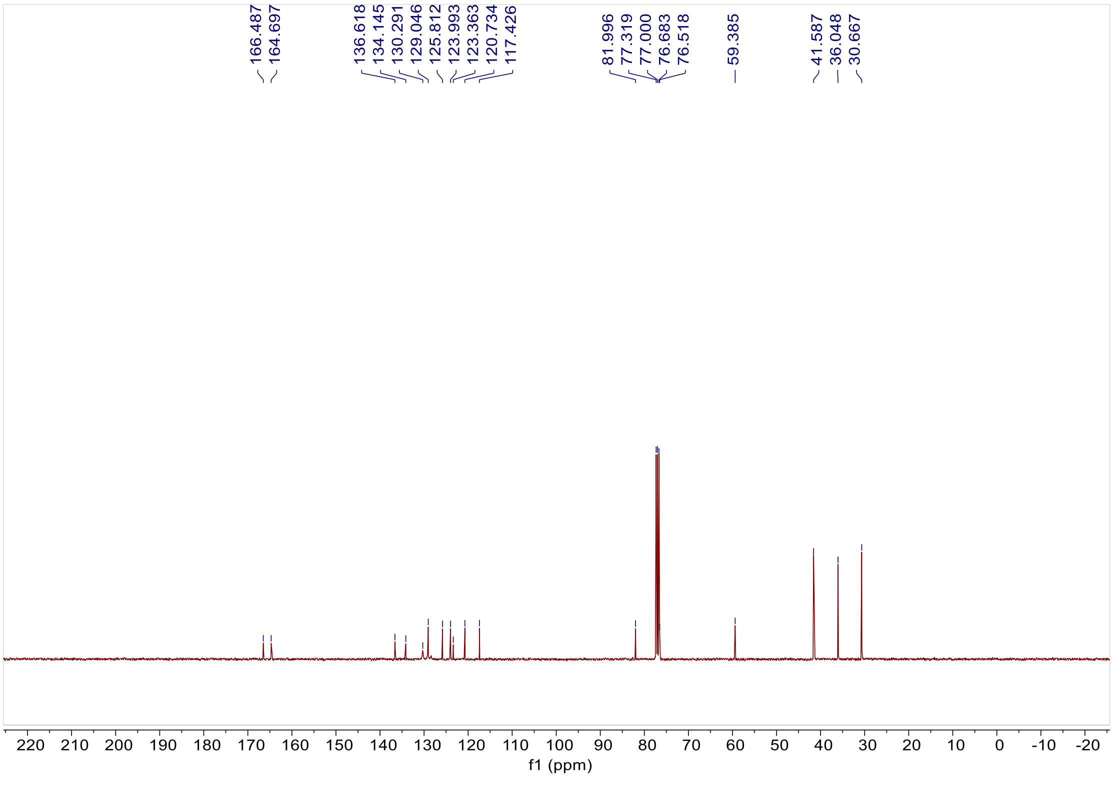

^{1}H NMR (400 MHz) Spectrum of 4l in $CDCl_3$

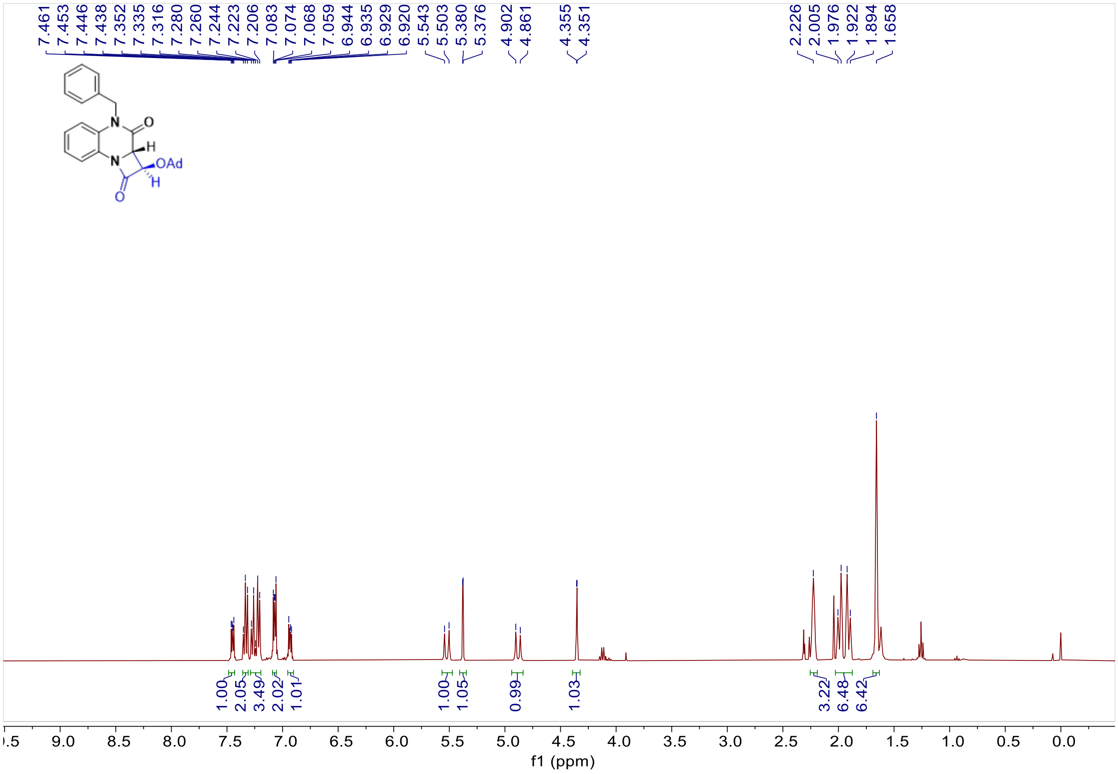

^{13}C NMR (100 MHz) Spectrum of 4l in $CDCl_3$

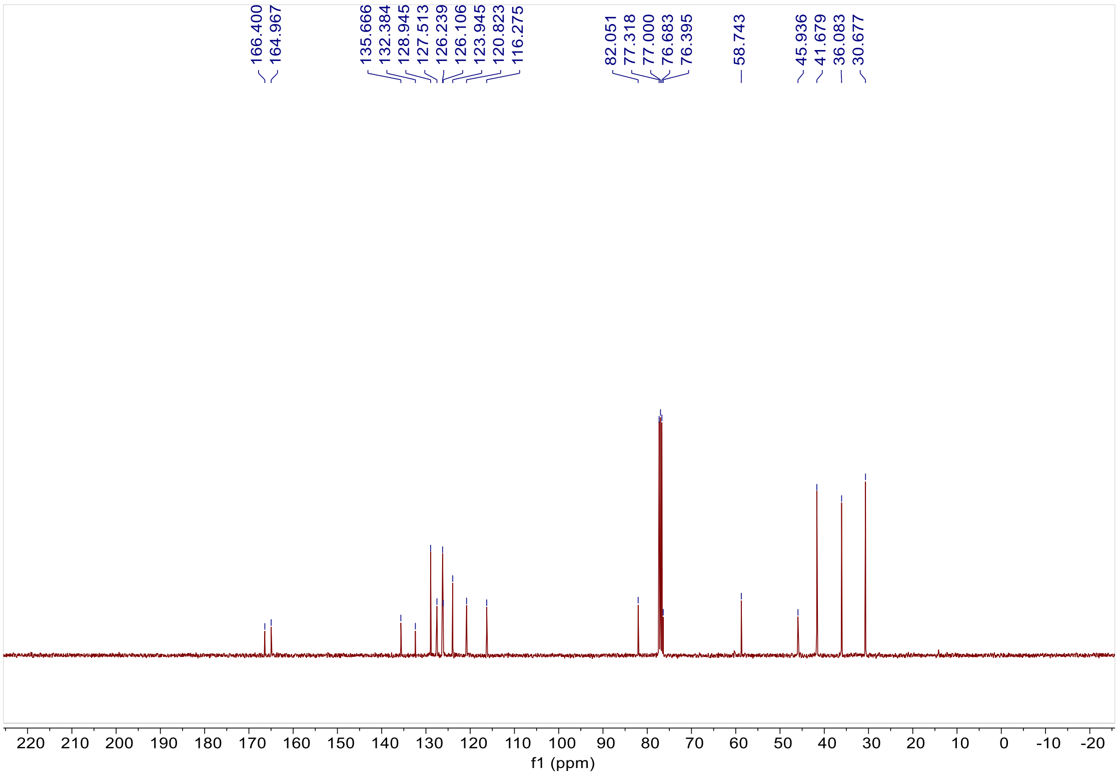

^{1}H NMR (400 MHz) Spectrum of 4m in $CDCl_3$

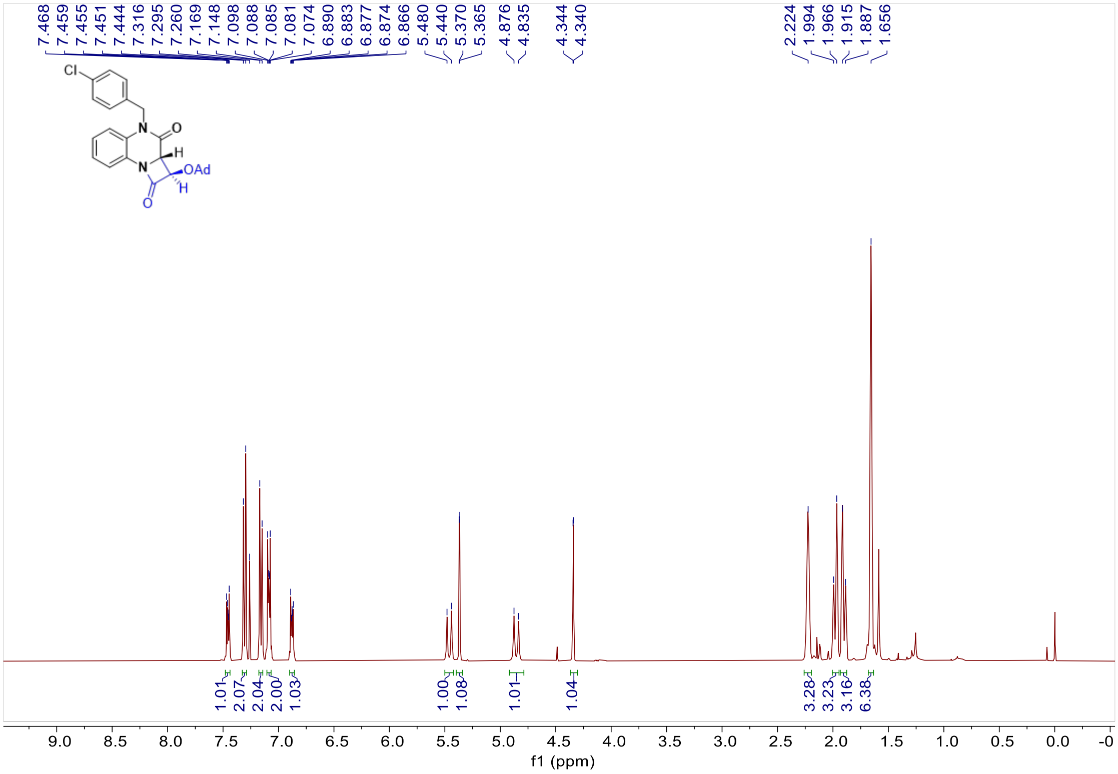

^{13}C NMR (100 MHz) Spectrum of 4m in $CDCl_3$

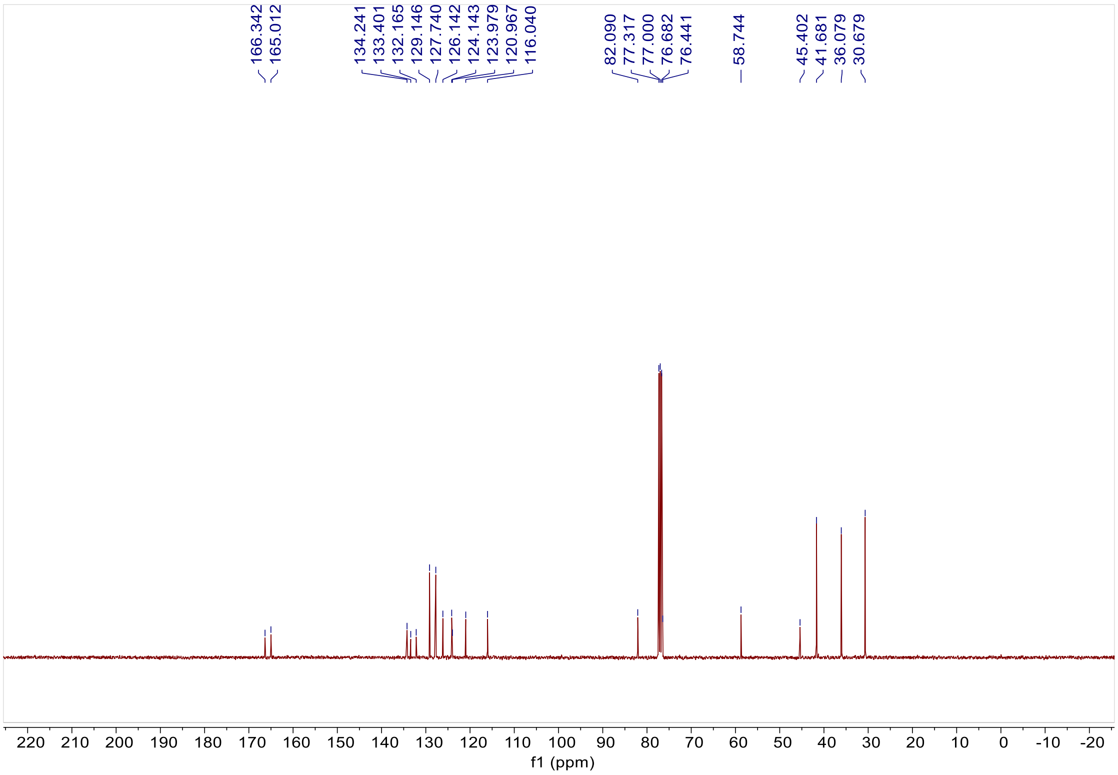

^{1}H NMR (400 MHz) Spectrum of 4n in $CDCl_3$

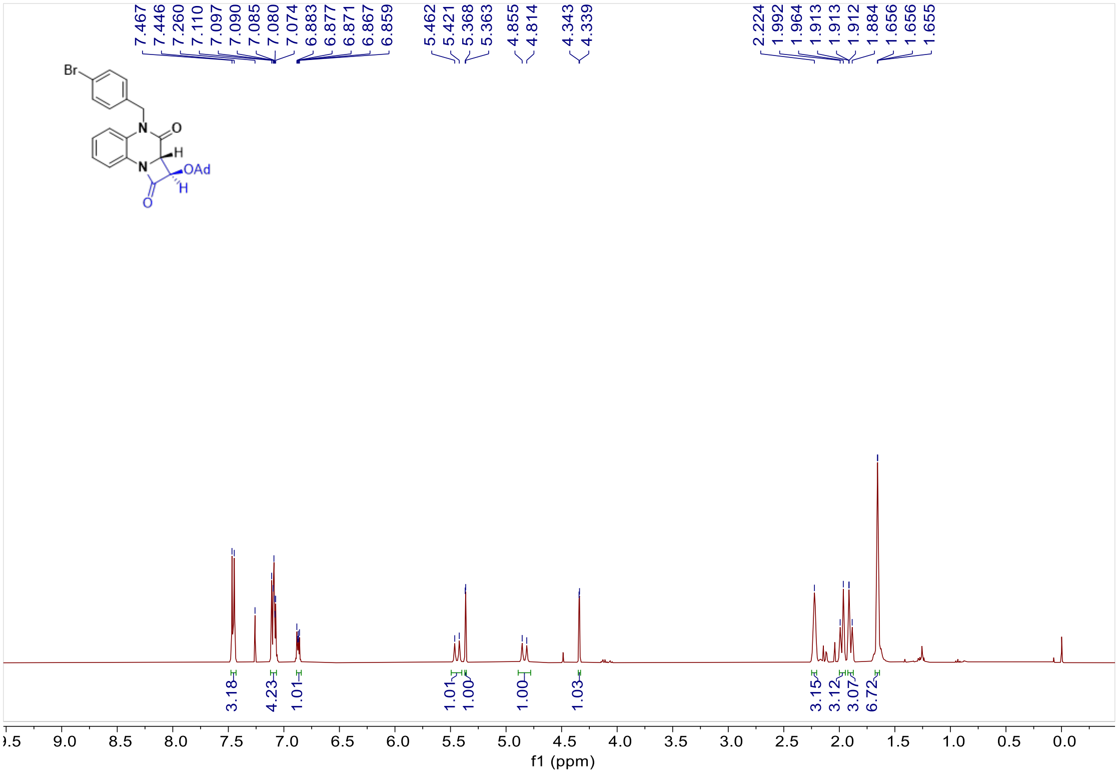

^{13}C NMR (100 MHz) Spectrum of 4n in $CDCl_3$

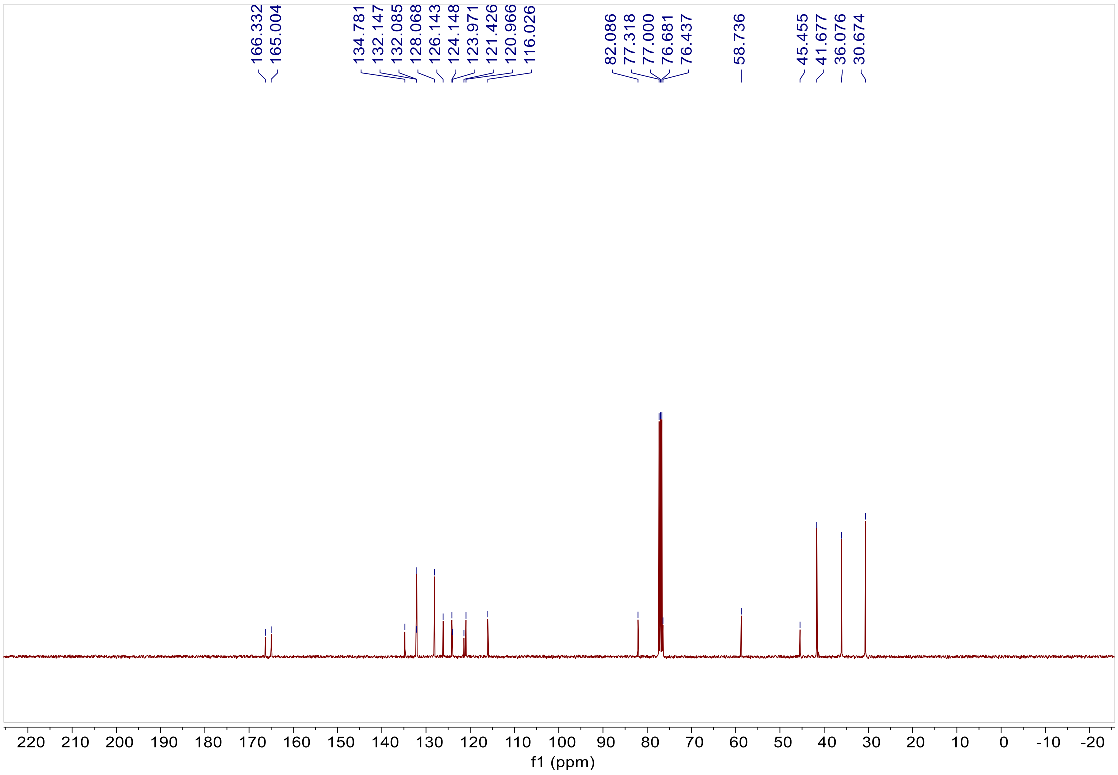

^{1}H NMR (400 MHz) Spectrum of 4o in $CDCl_3$

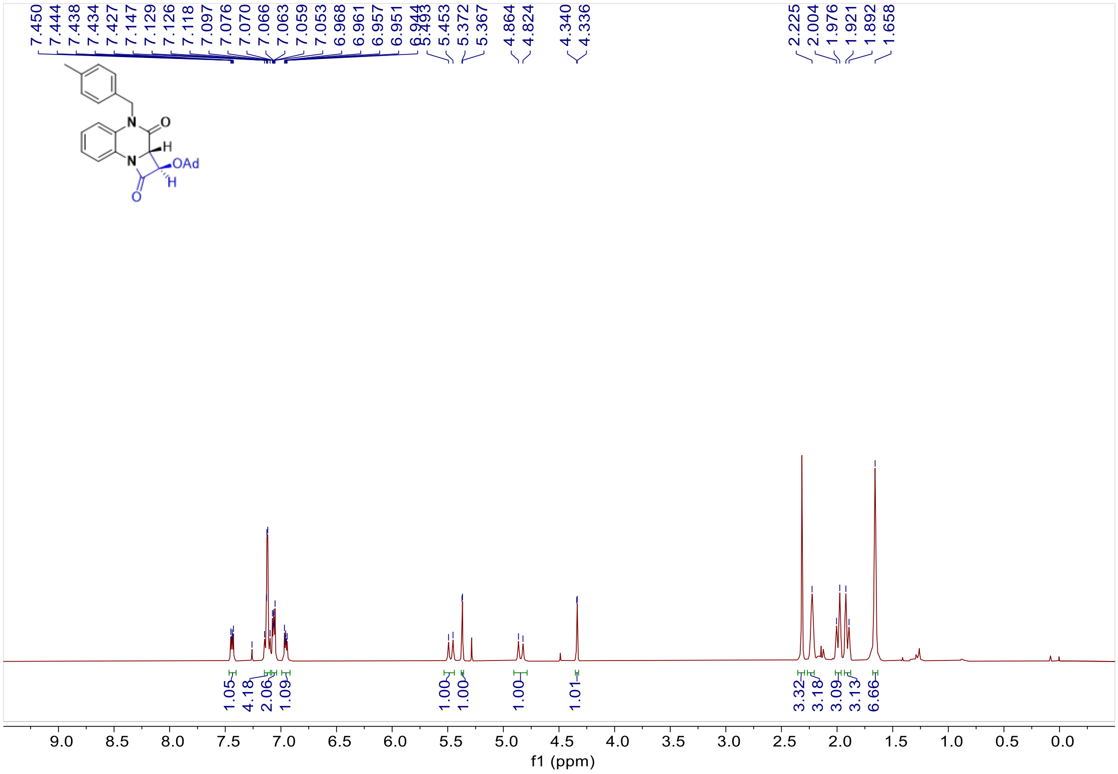

^{13}C NMR (100 MHz) Spectrum of 4o in $CDCl_3$

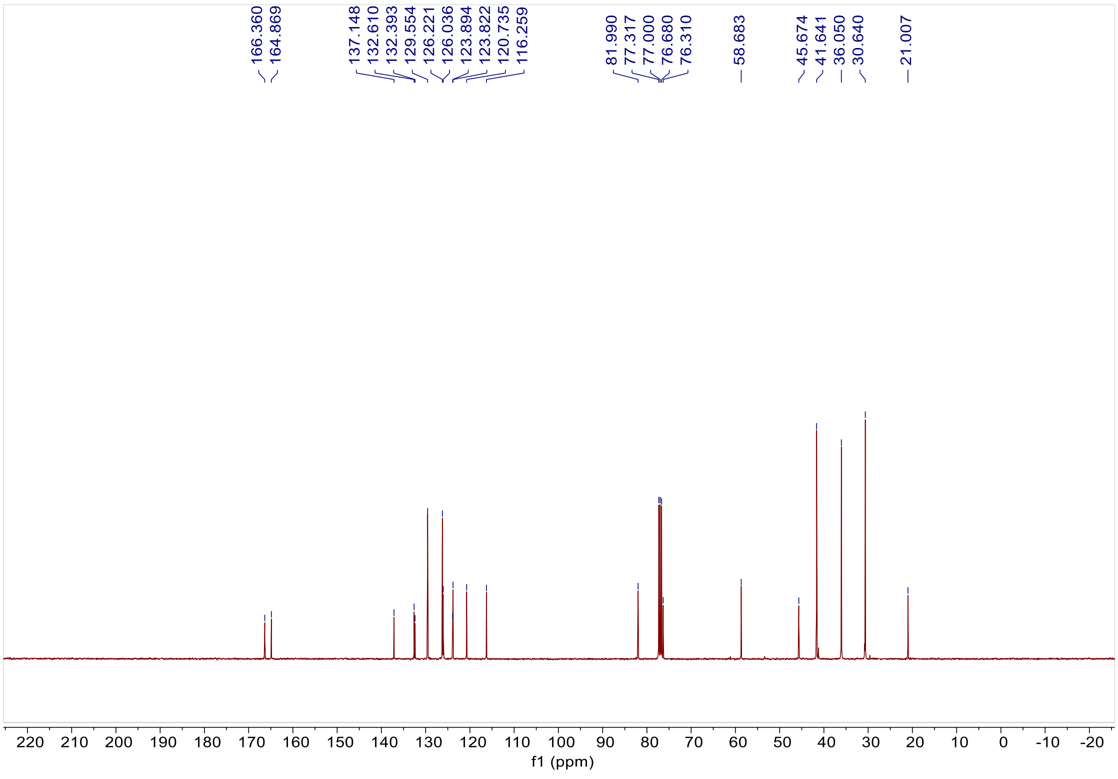

^{1}H NMR (400 MHz) Spectrum of 4p in $CDCl_3$

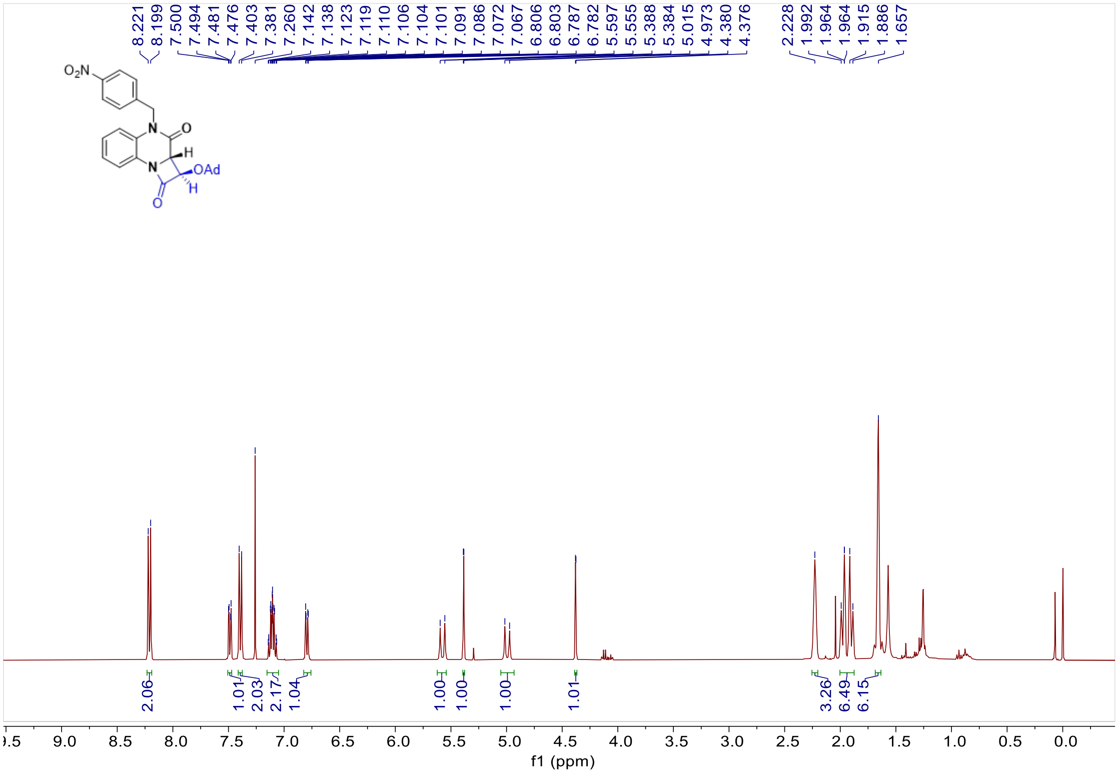

^{13}C NMR (100 MHz) Spectrum of 4p in $CDCl_3$

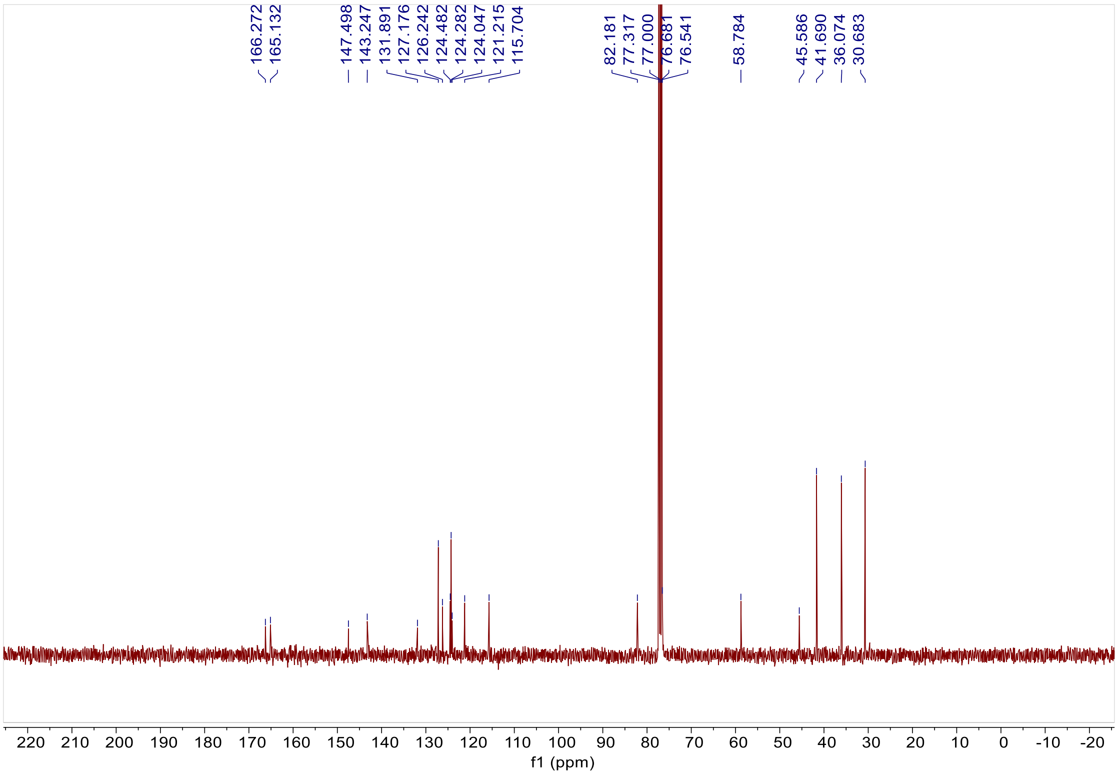

^{1}H NMR (400 MHz) Spectrum of 4q in CDCl$_3$

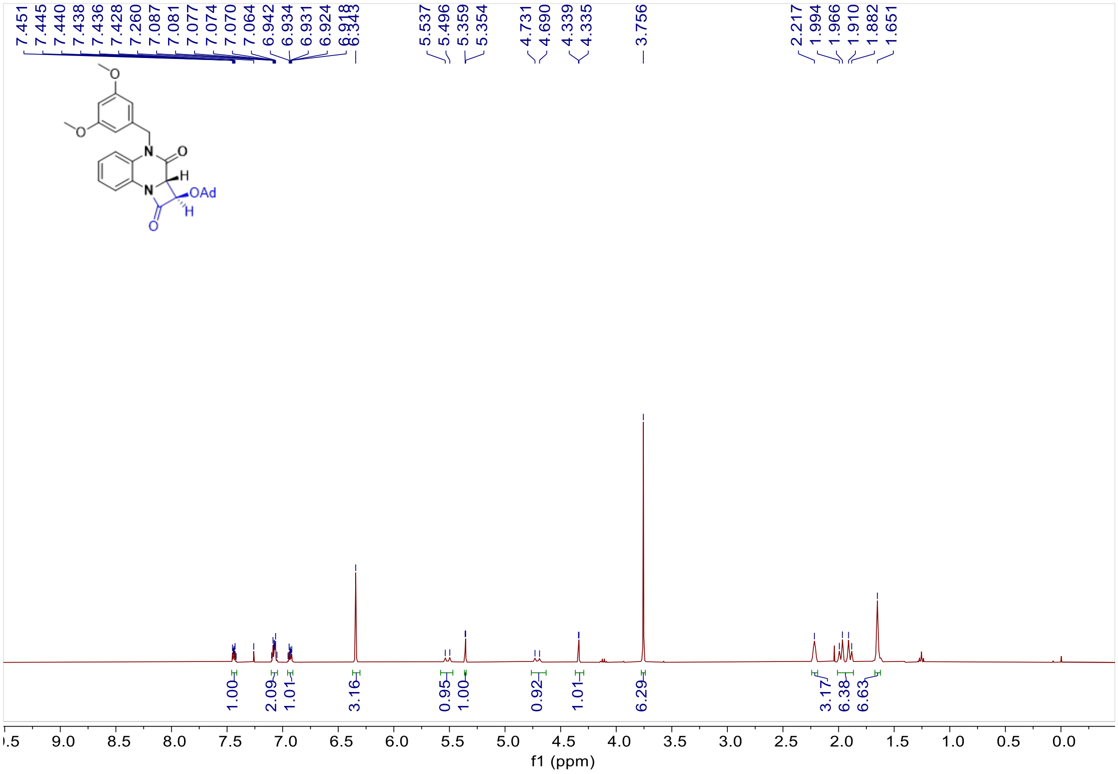

^{13}C NMR (100 MHz) Spectrum of 4q in CDCl$_3$

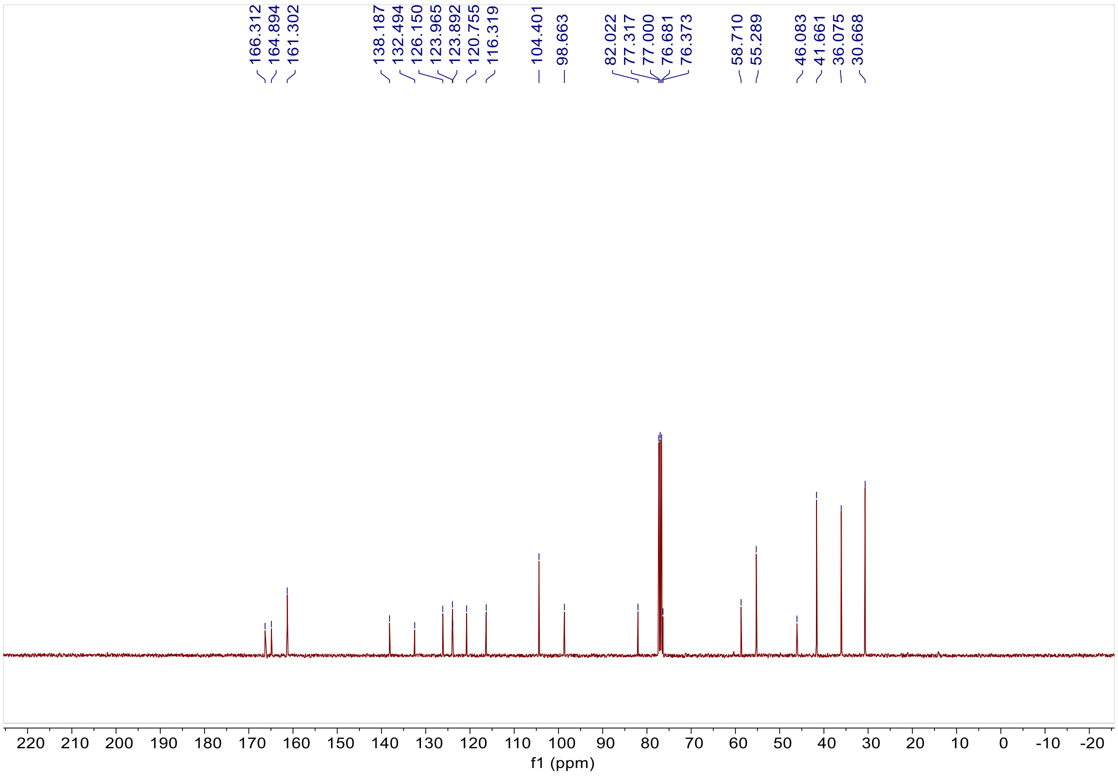

^{1}H NMR (400 MHz) Spectrum of 4r in $CDCl_3$

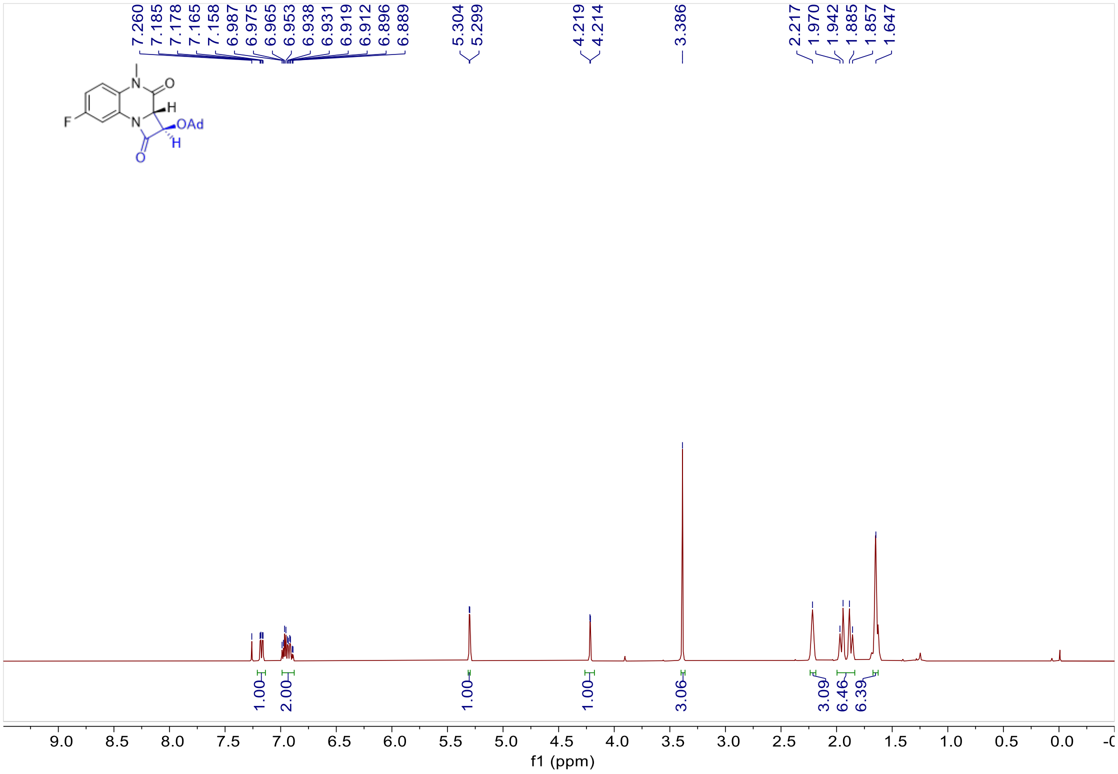

^{13}C NMR (100 MHz) Spectrum of 4r in $CDCl_3$

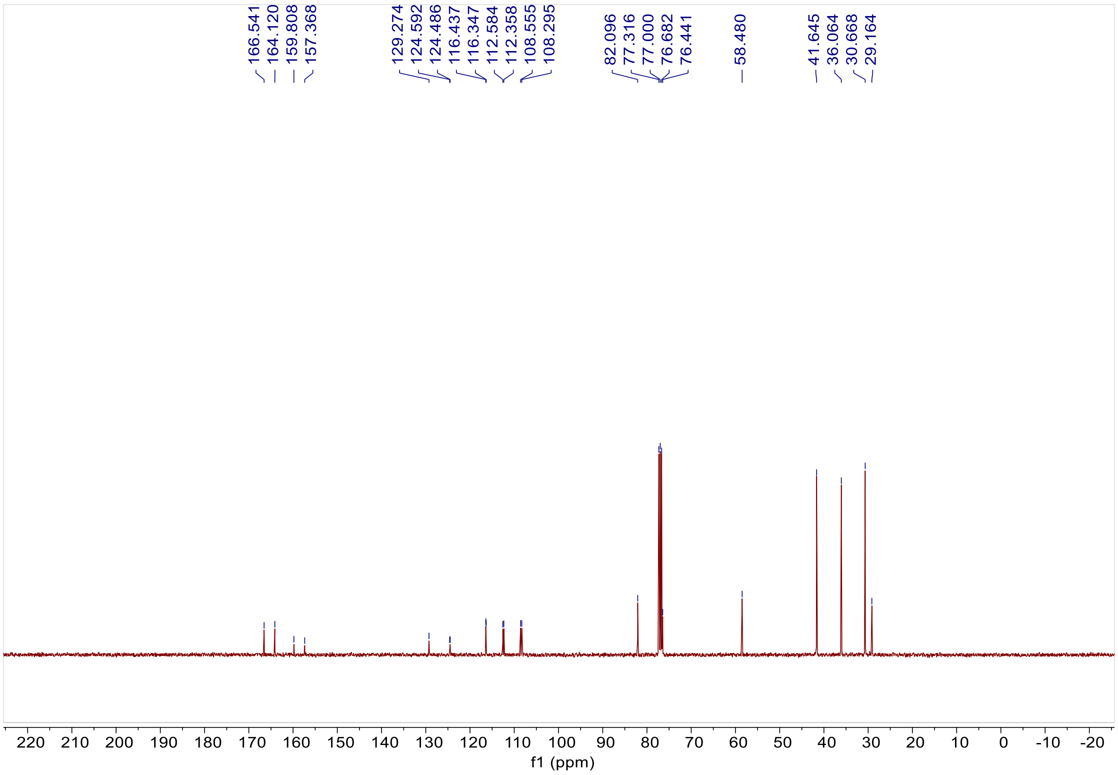

^{19}F NMR (375 MHz) Spectrum of 4q in $CDCl_3$

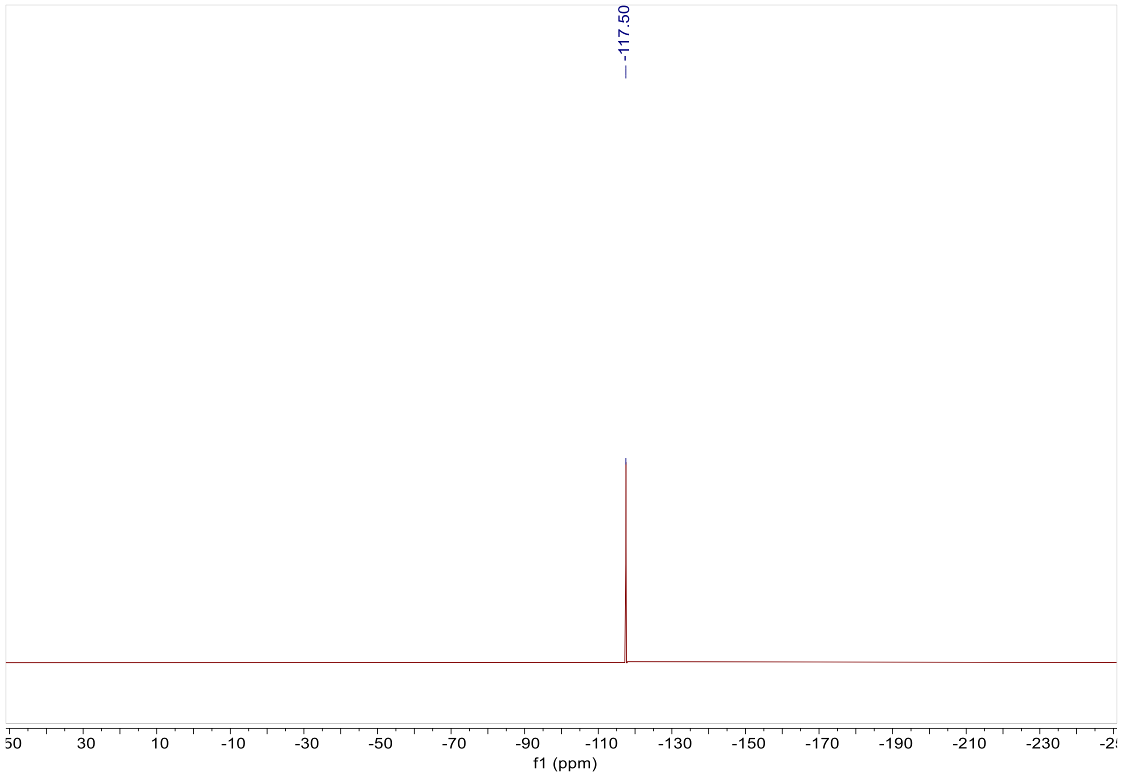

^{1}H NMR (400 MHz) Spectrum of 4s in $CDCl_3$

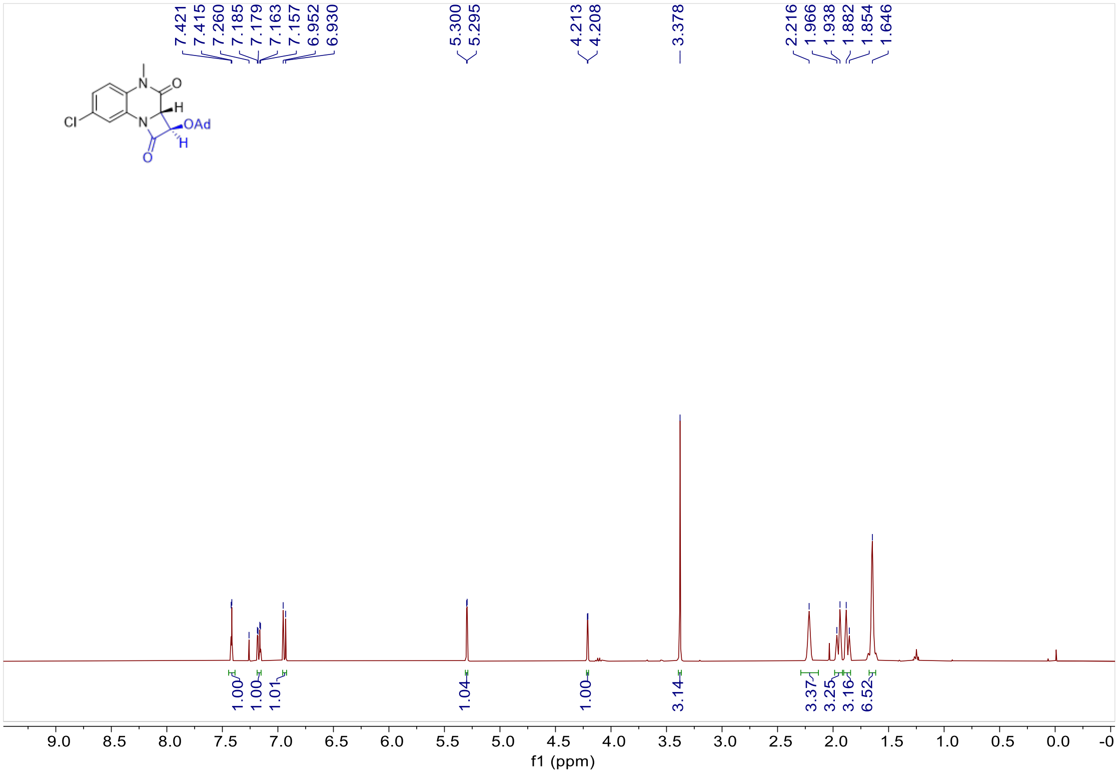

^{13}C NMR (100 MHz) Spectrum of 4s in $CDCl_3$

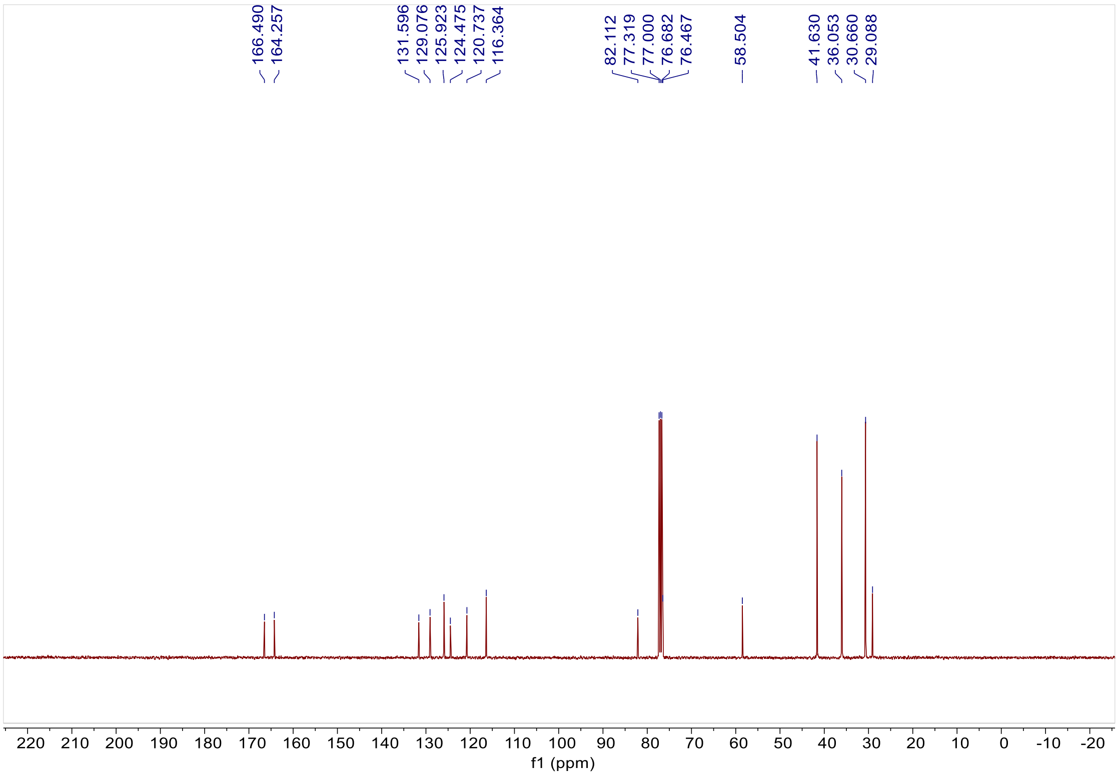

^{1}H NMR (400 MHz) Spectrum of 4t in CDCl$_3$

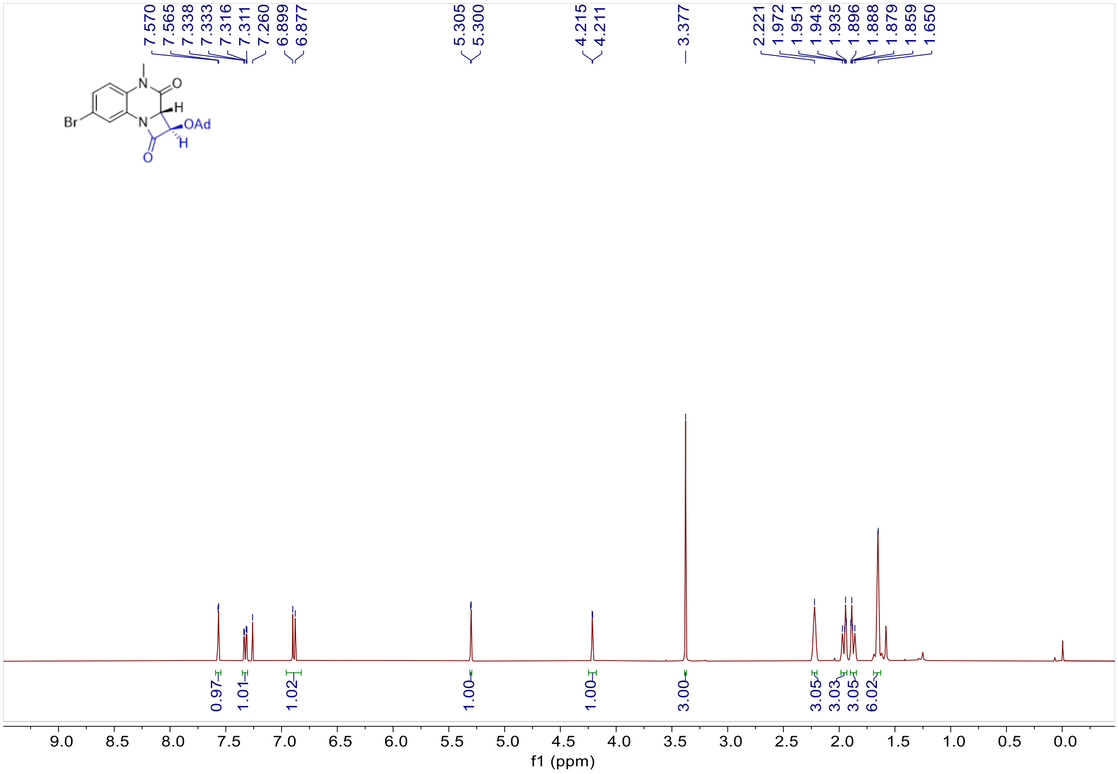

^{13}C NMR (100 MHz) Spectrum of 4t in CDCl$_3$

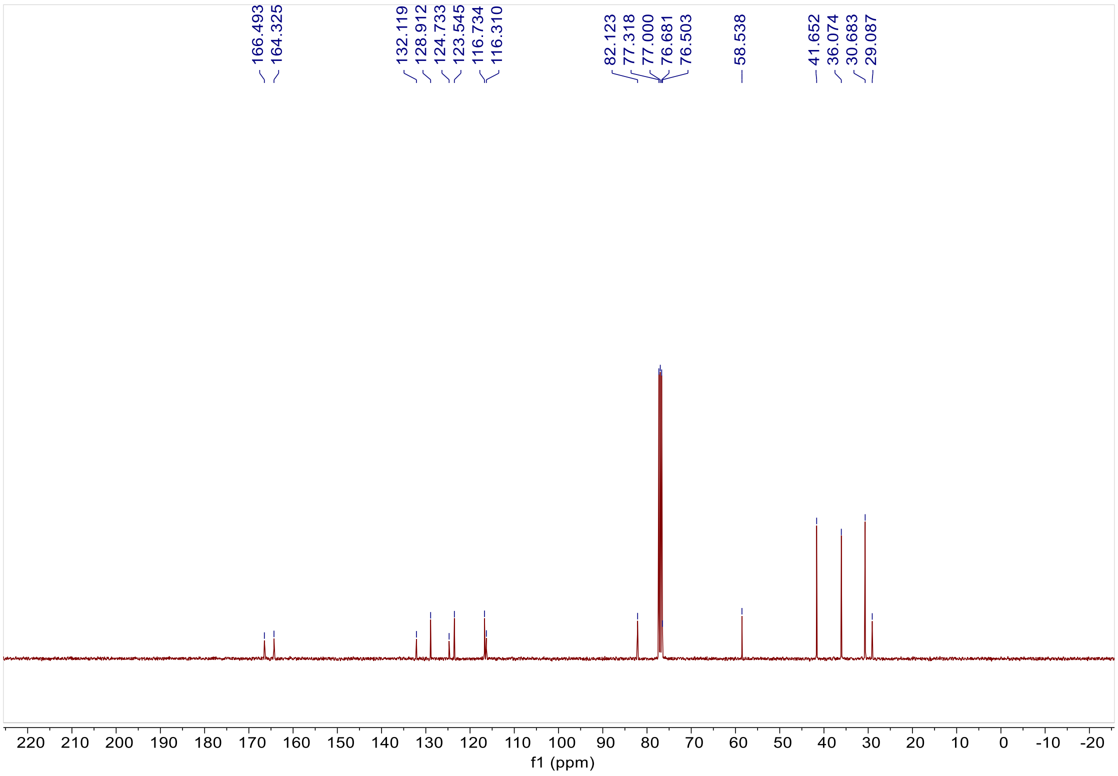

^{1}H NMR (400 MHz) Spectrum of 4u in $CDCl_3$

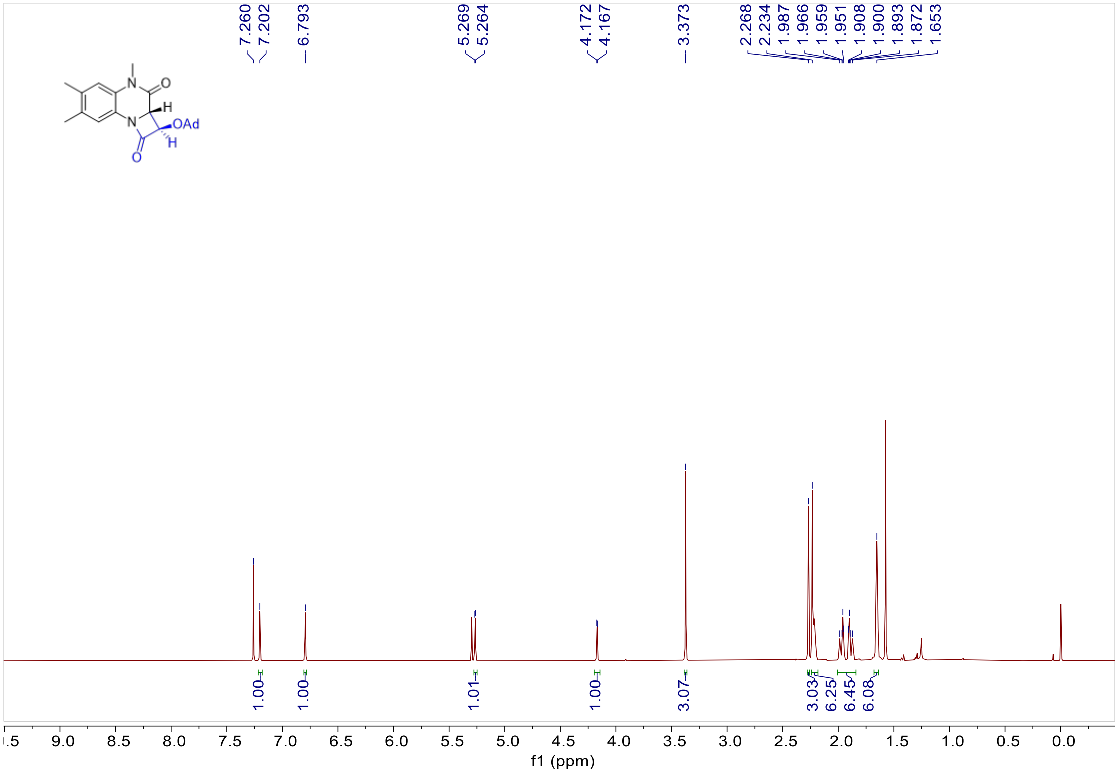

^{13}C NMR (100 MHz) Spectrum of 4u in $CDCl_3$

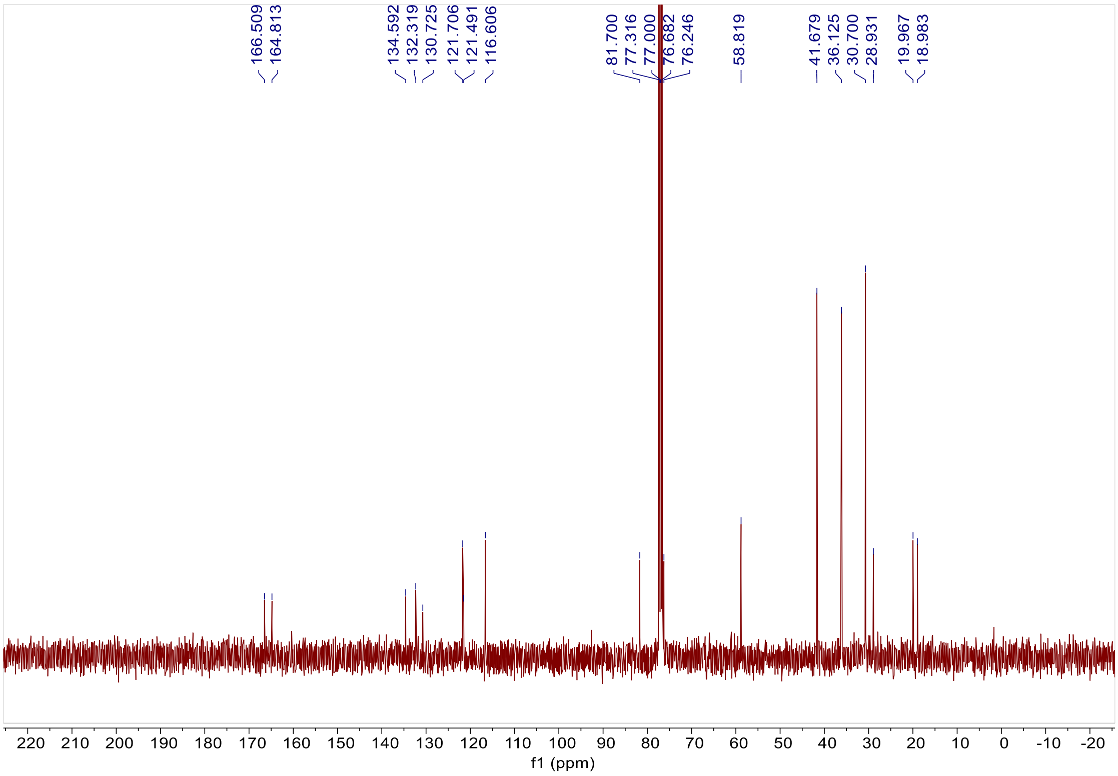

^{1}H NMR (400 MHz) Spectrum of 4v in $CDCl_3$

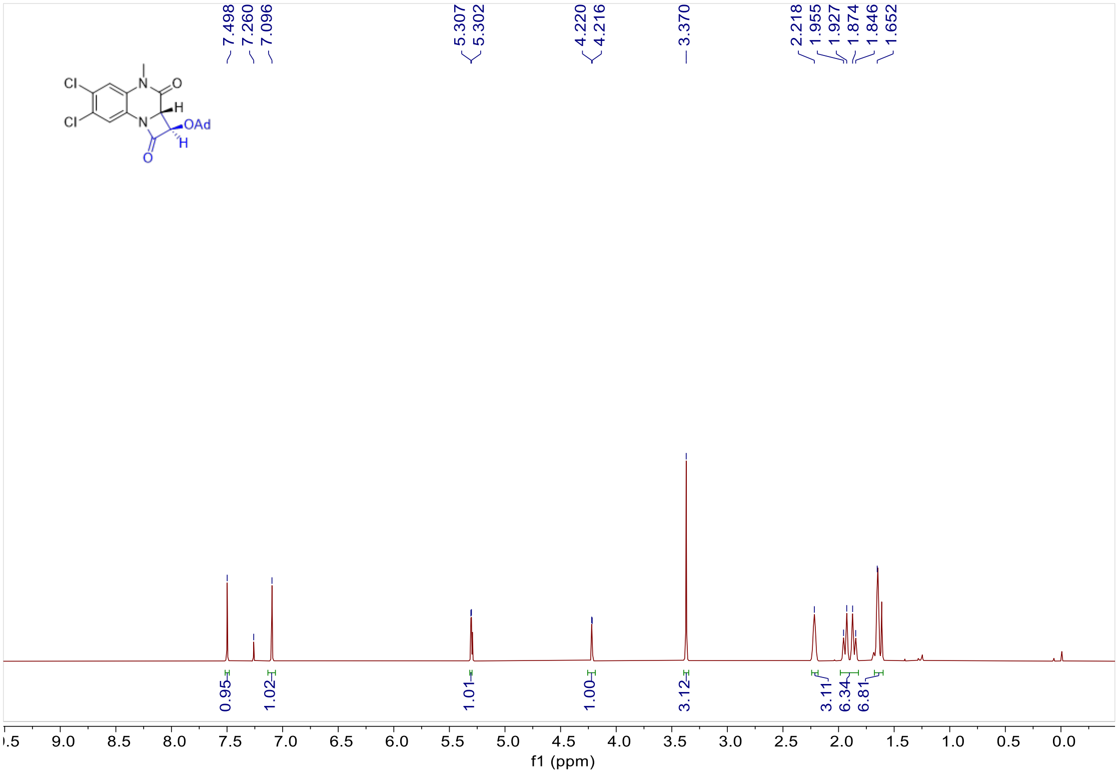

^{13}C NMR (100 MHz) Spectrum of 4v in $CDCl_3$

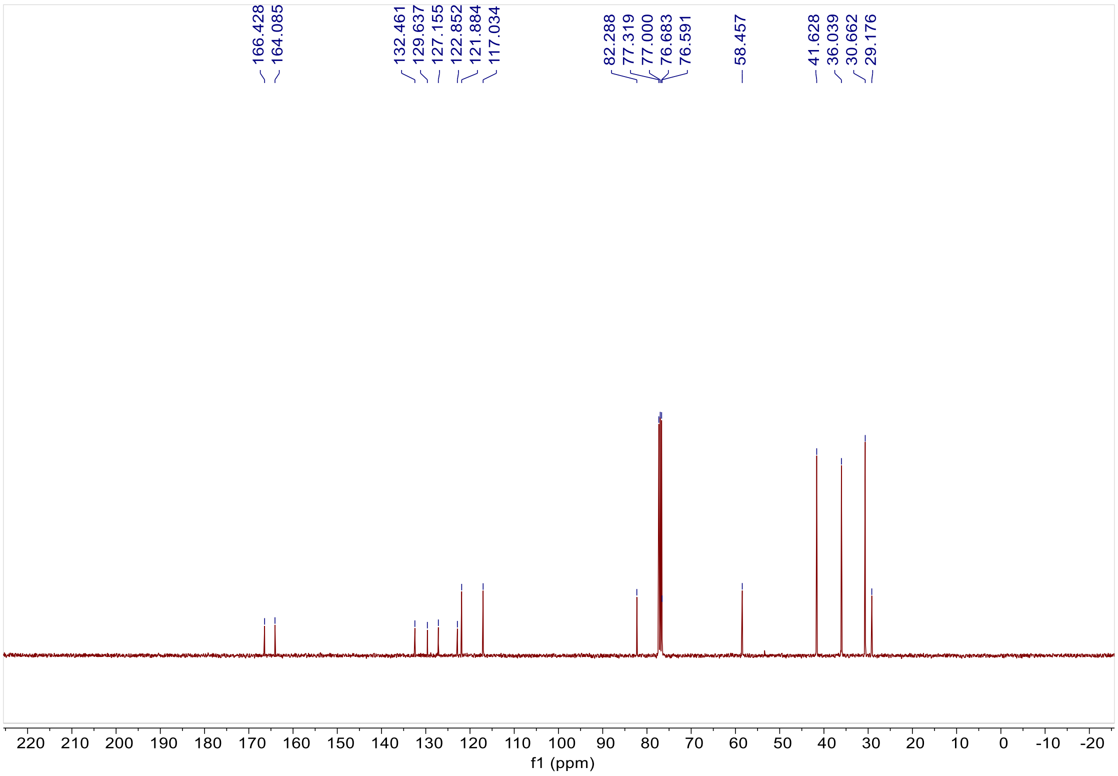

^{1}H NMR (400 MHz) Spectrum of 6a in CDCl$_3$

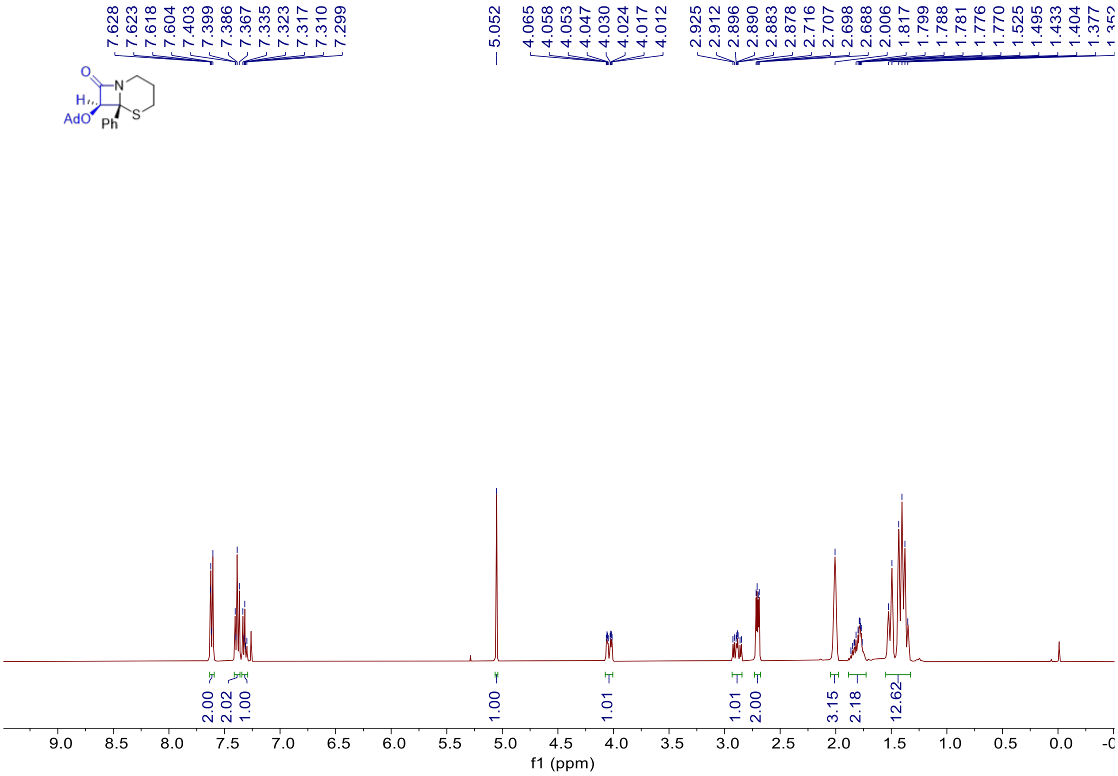

^{13}C NMR (100 MHz) Spectrum of 6a in CDCl$_3$

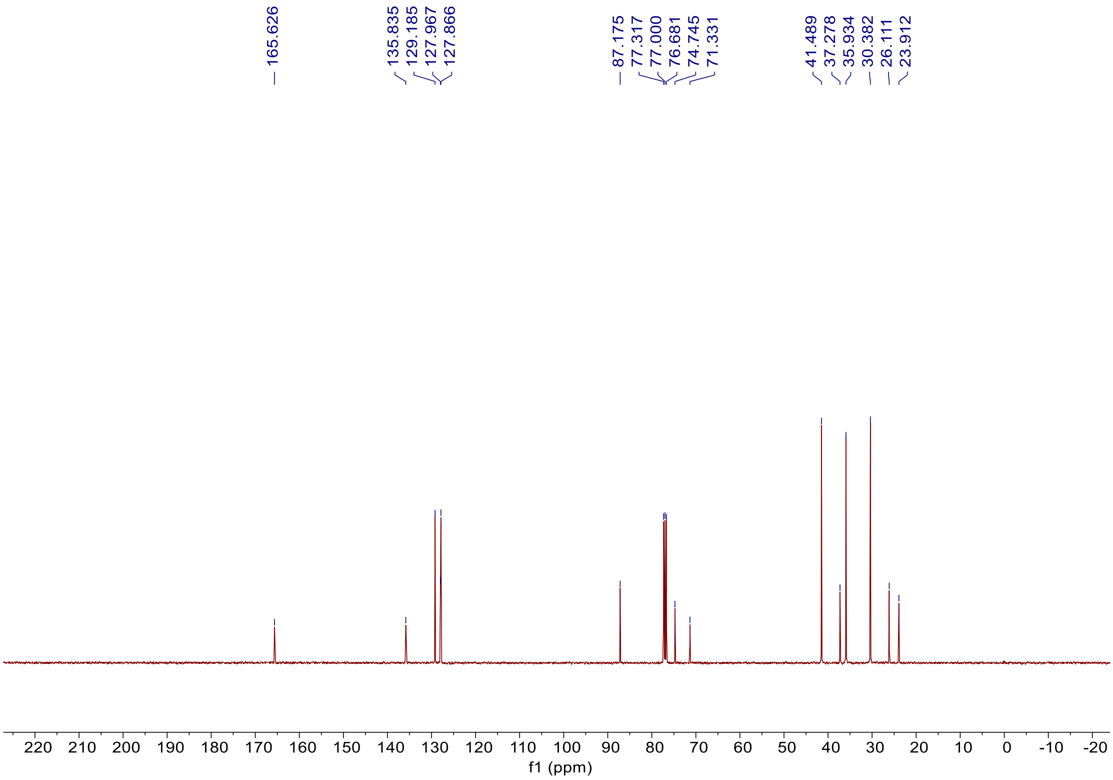

^{1}H NMR (600 MHz) Spectrum of 6b in CDCl$_3$

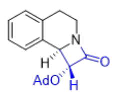

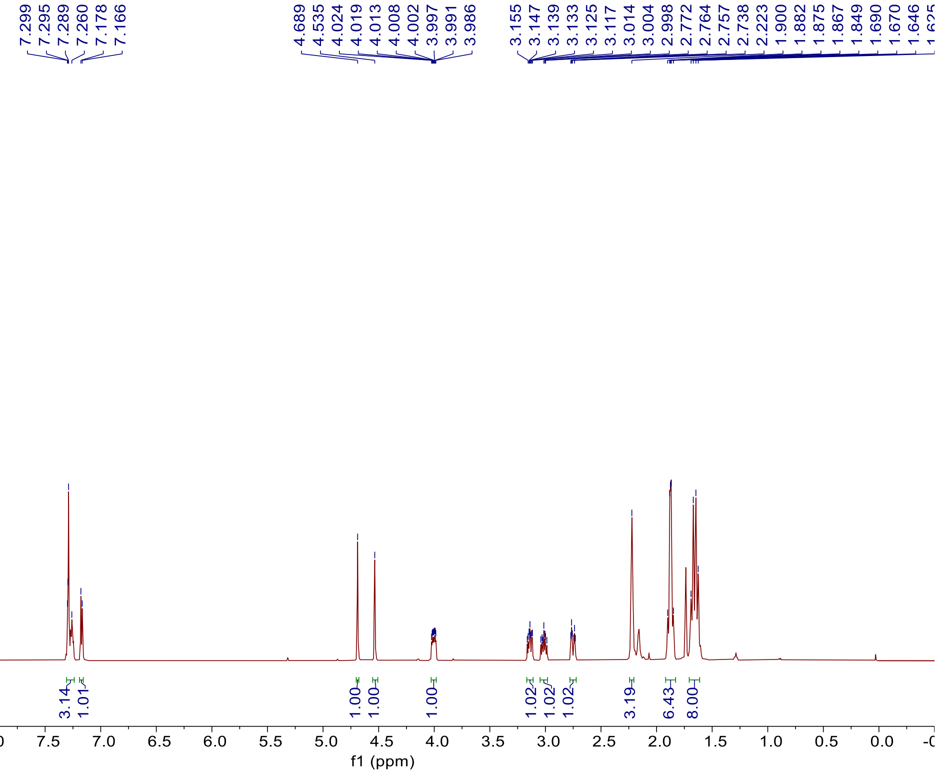

^{13}C NMR (150 MHz) Spectrum of 6b in CDCl$_3$

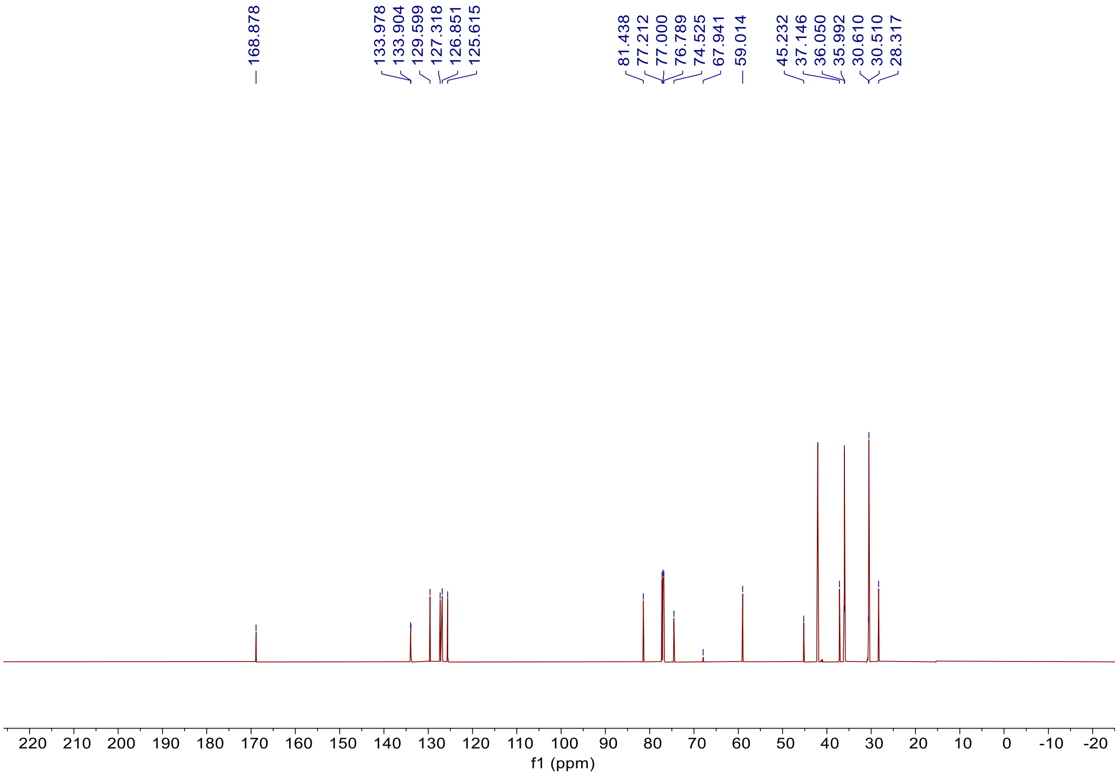

^{1}H NMR (400 MHz) Spectrum of 6c in CDCl$_3$

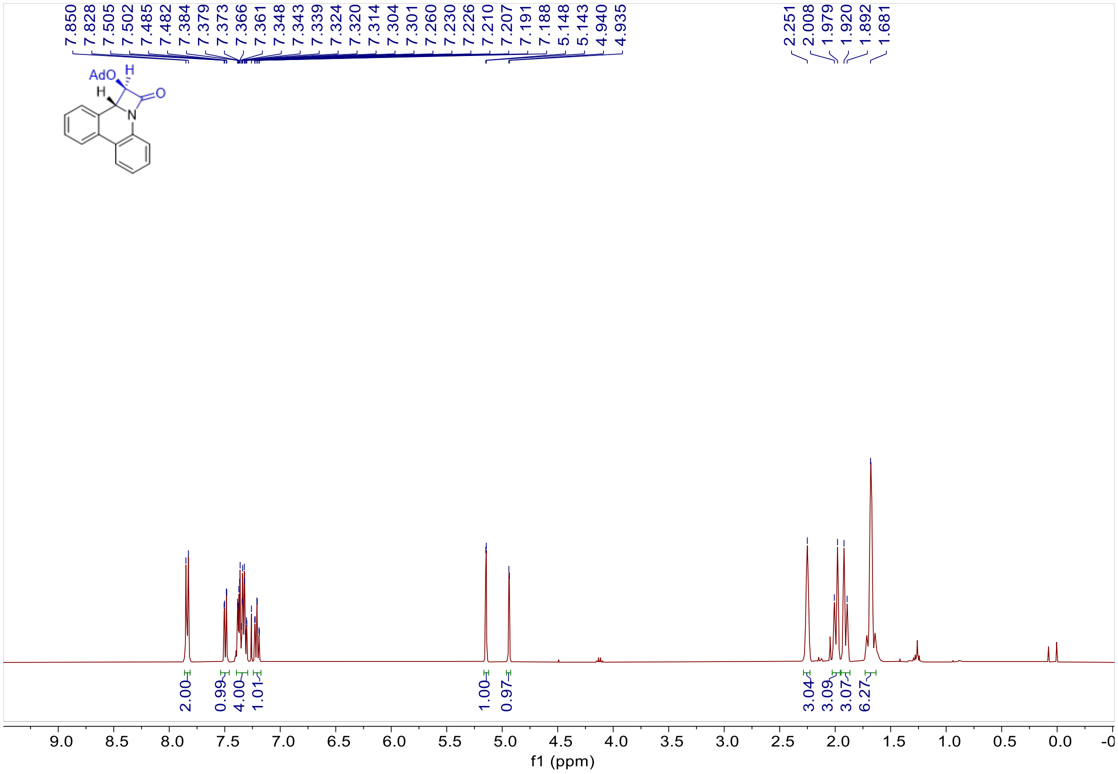

^{13}C NMR (100 MHz) Spectrum of 6c in CDCl$_3$

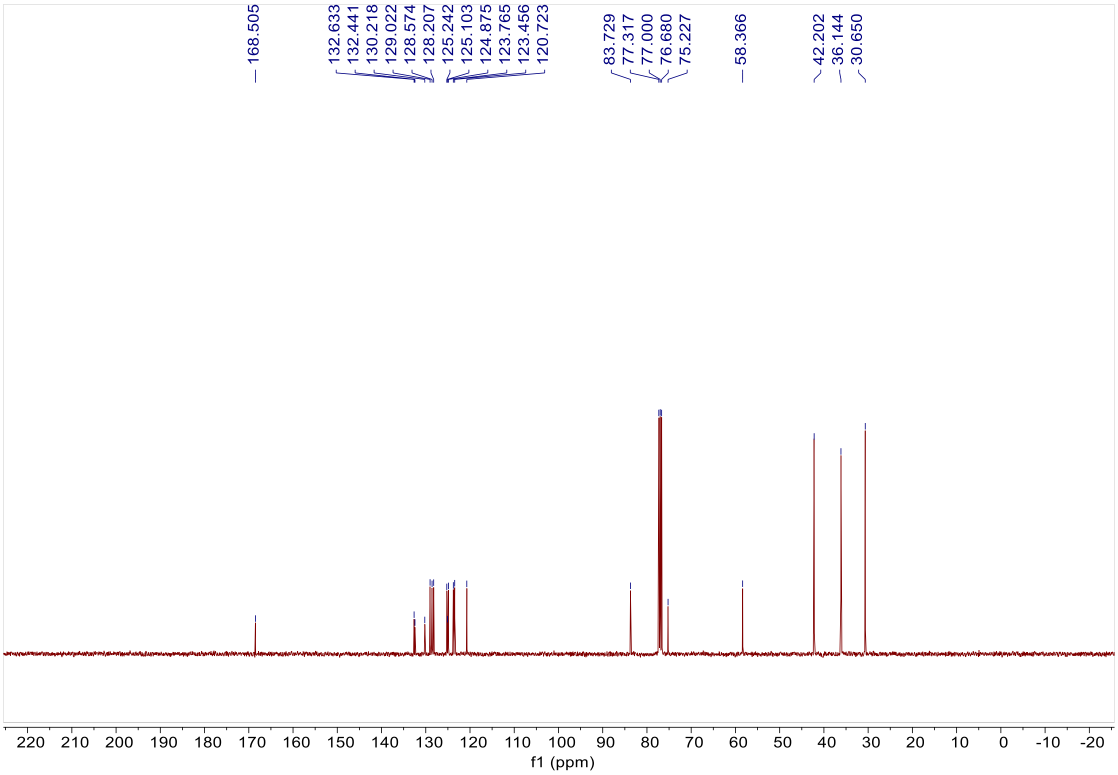

^{1}H NMR (400 MHz) Spectrum of 6d in $CDCl_3$

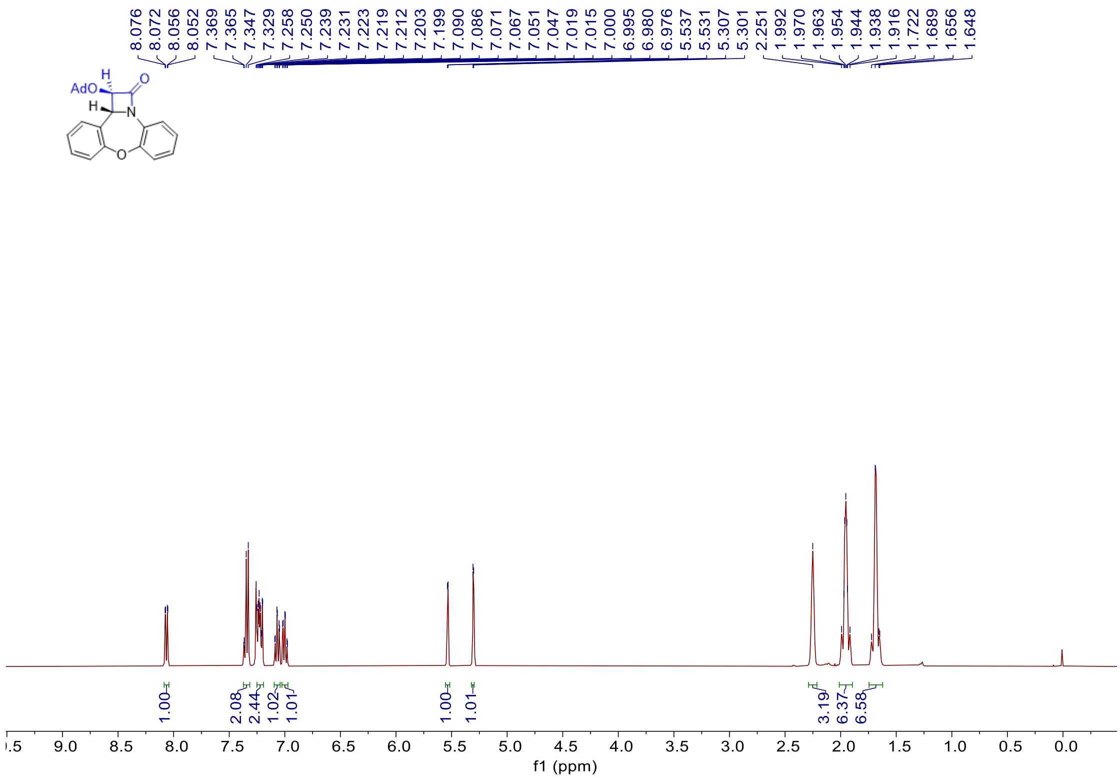

^{13}C NMR (100 MHz) Spectrum of 6d in $CDCl_3$

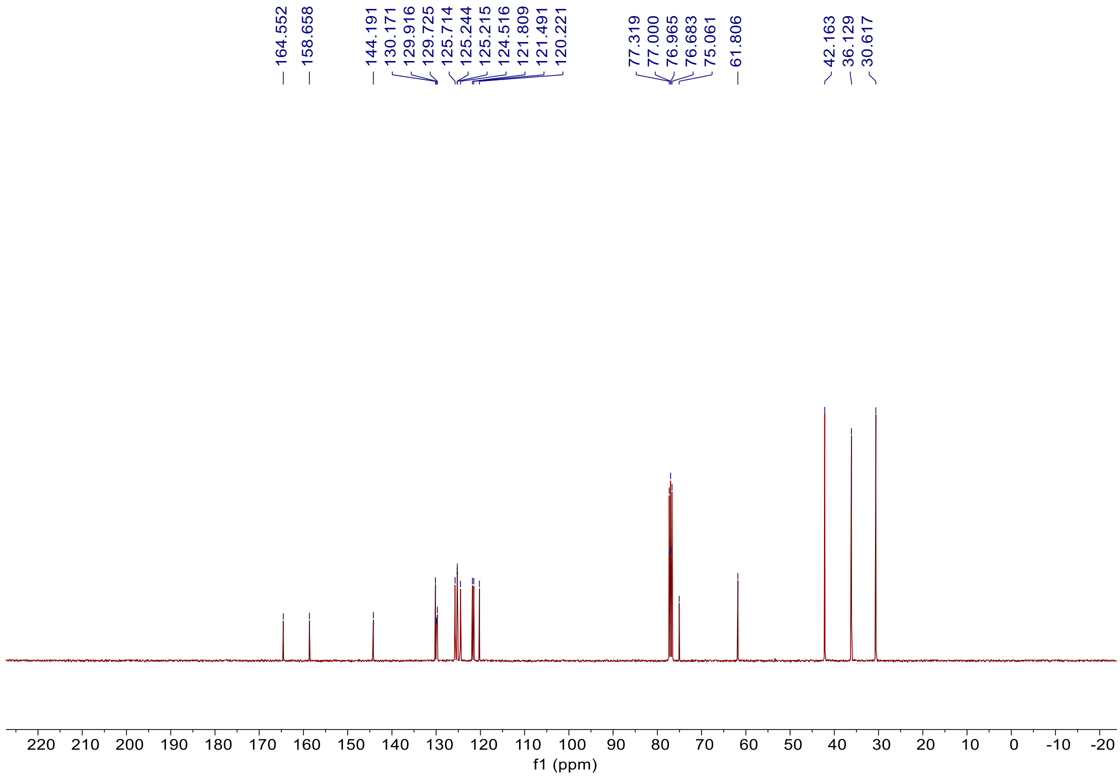

^{1}H NMR (600 MHz) Spectrum of 6e in $CDCl_3$

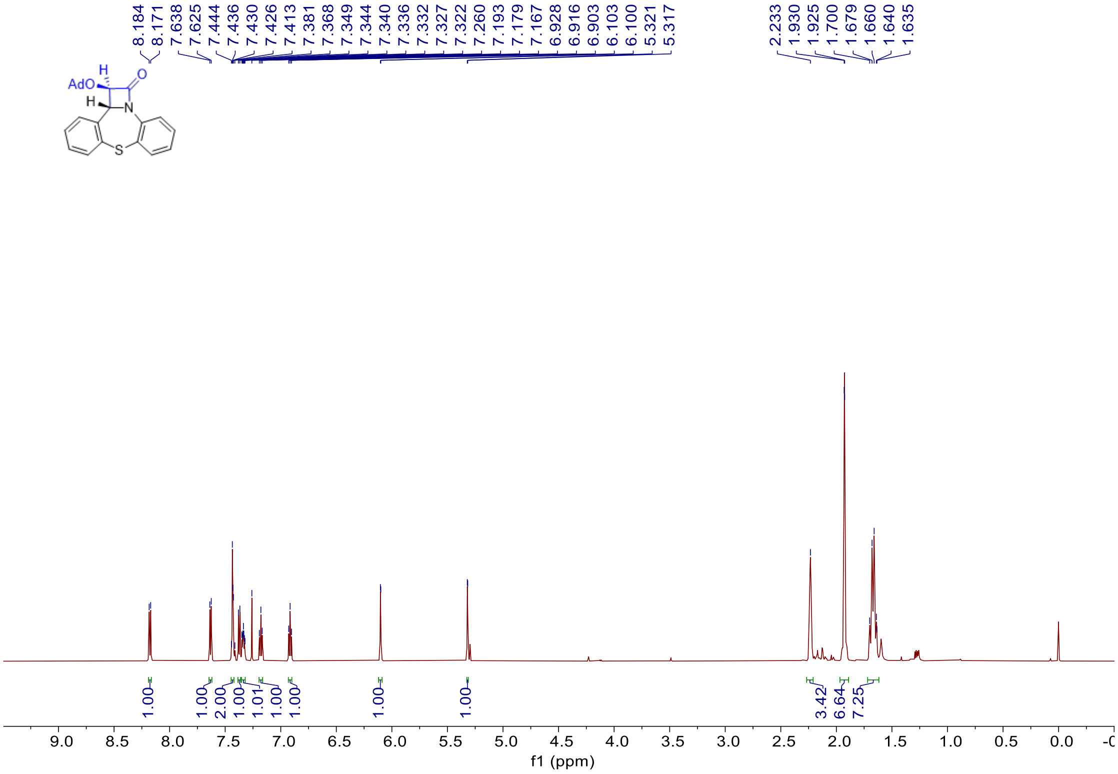

^{13}C NMR (150 MHz) Spectrum of 6e in $CDCl_3$

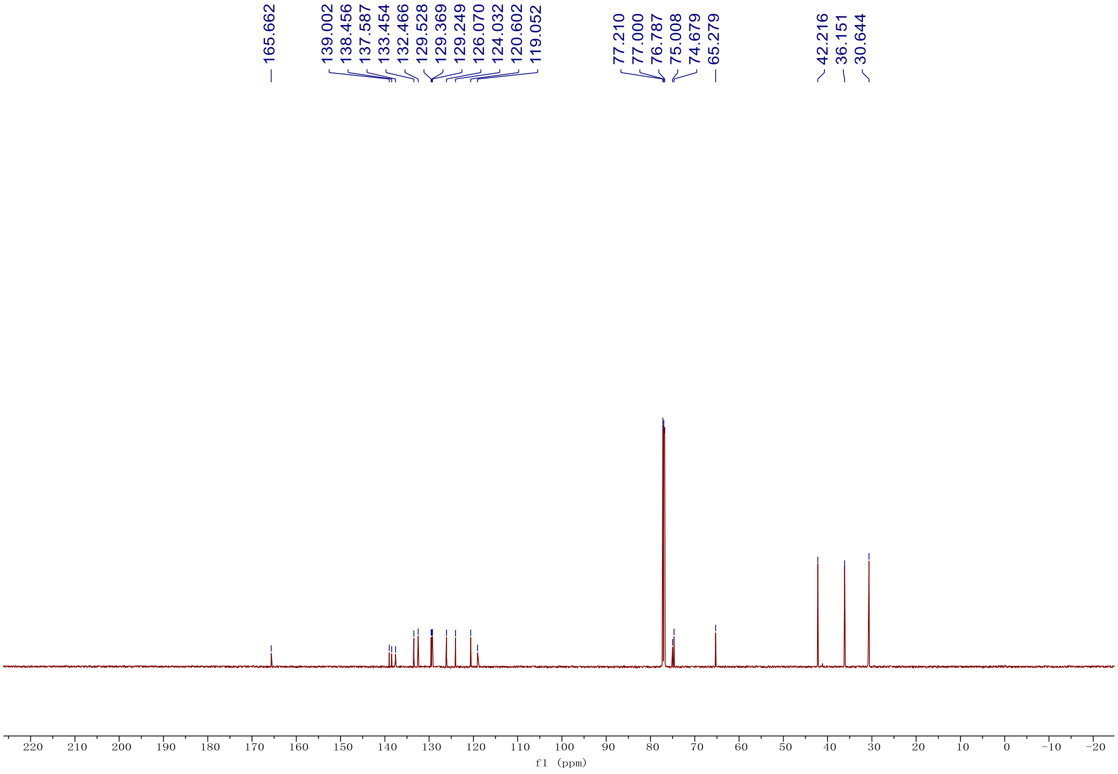

^{1}H NMR (600 MHz) Spectrum of 6f in CD_3CN

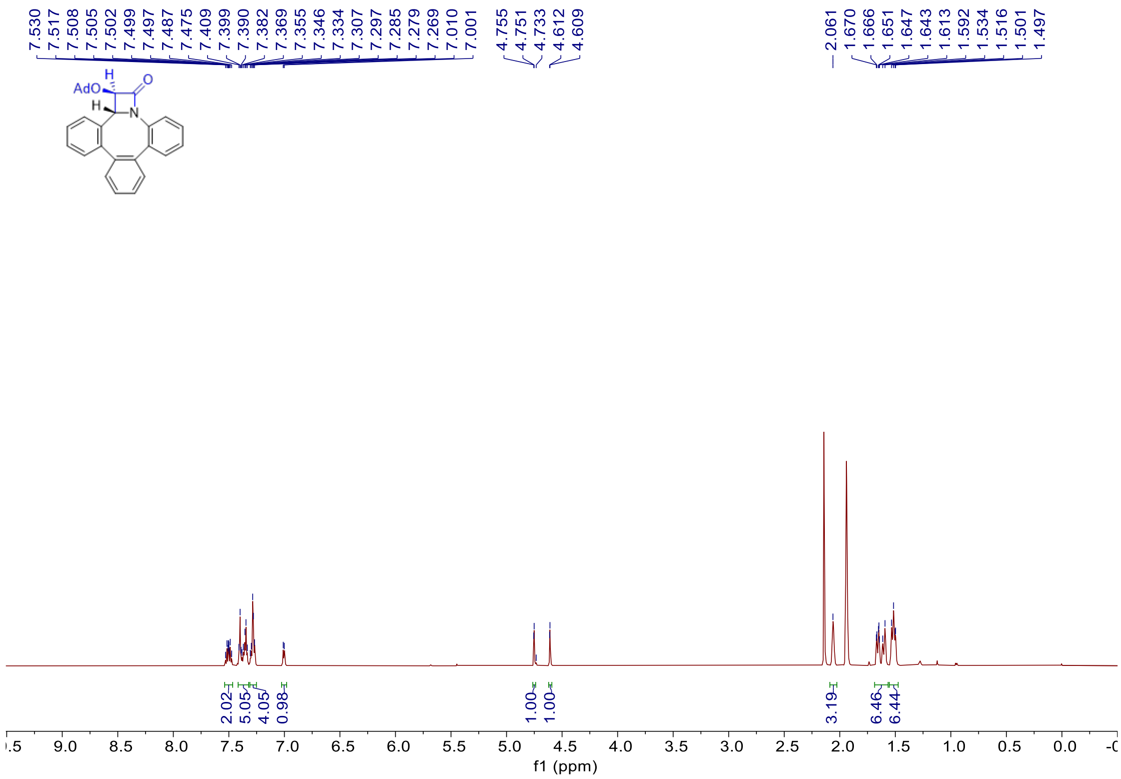

^{13}C NMR (150 MHz) Spectrum of 6f in CD_3CN

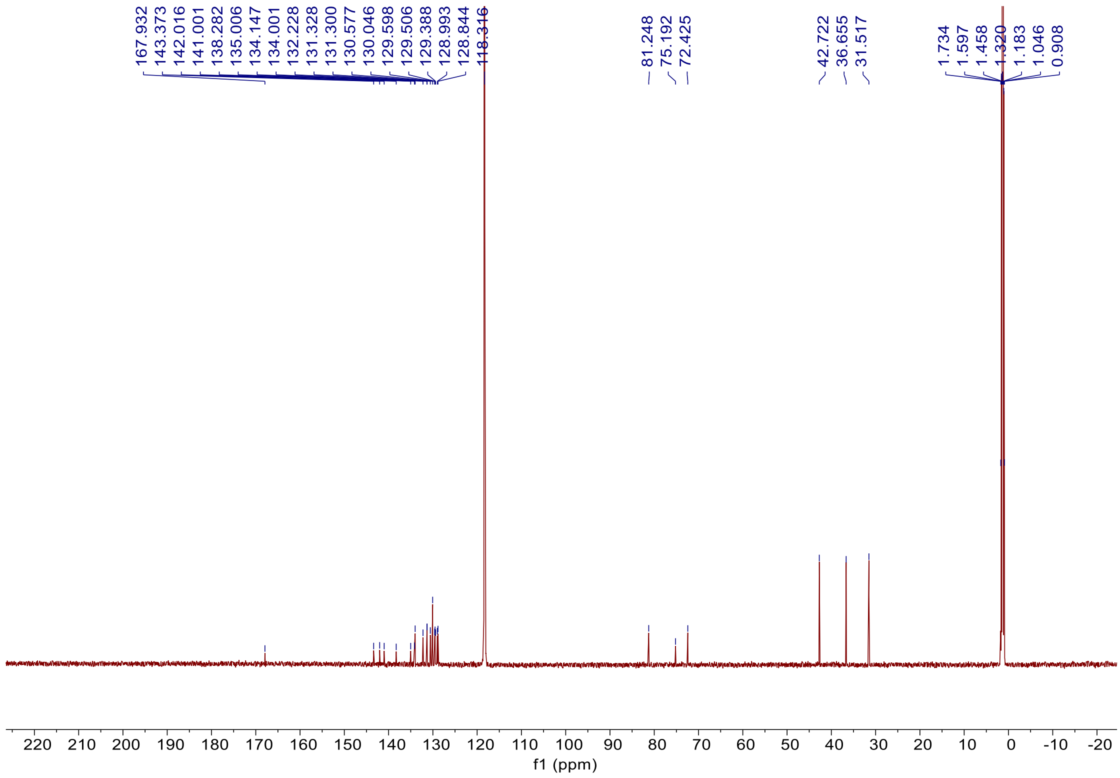

^{1}H NMR (400 MHz) Spectrum of 6g in CDCl$_3$

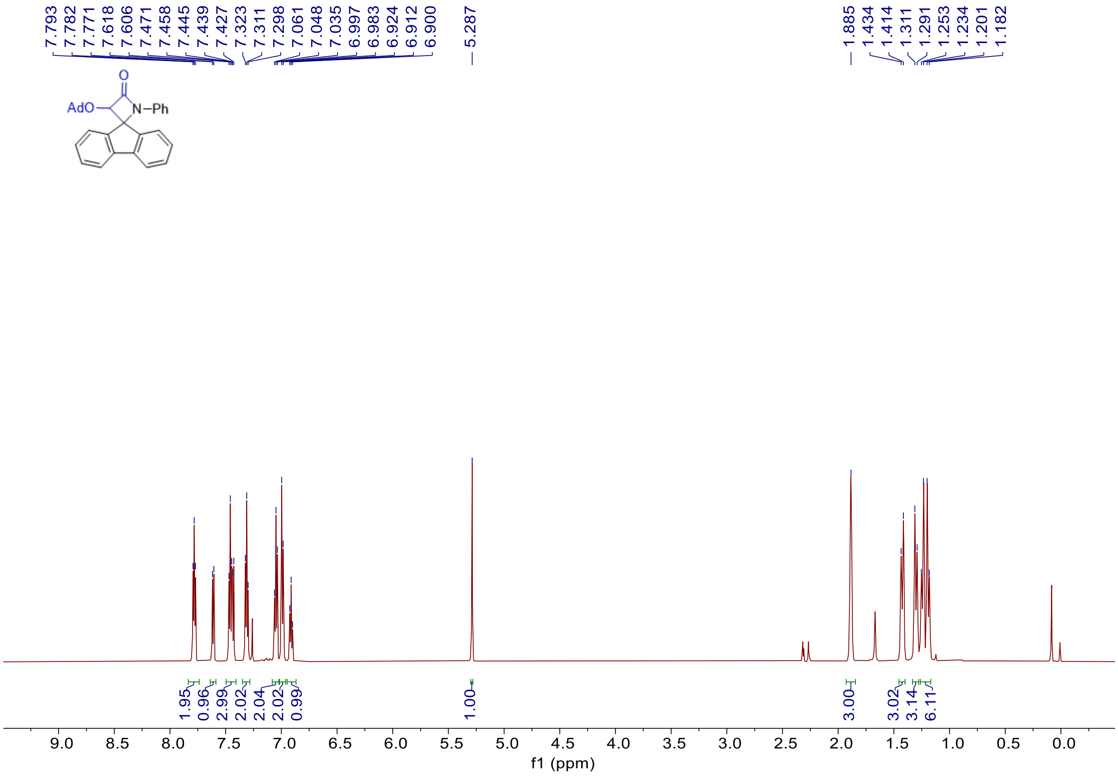

^{13}C NMR (100 MHz) Spectrum of 6g in CDCl$_3$

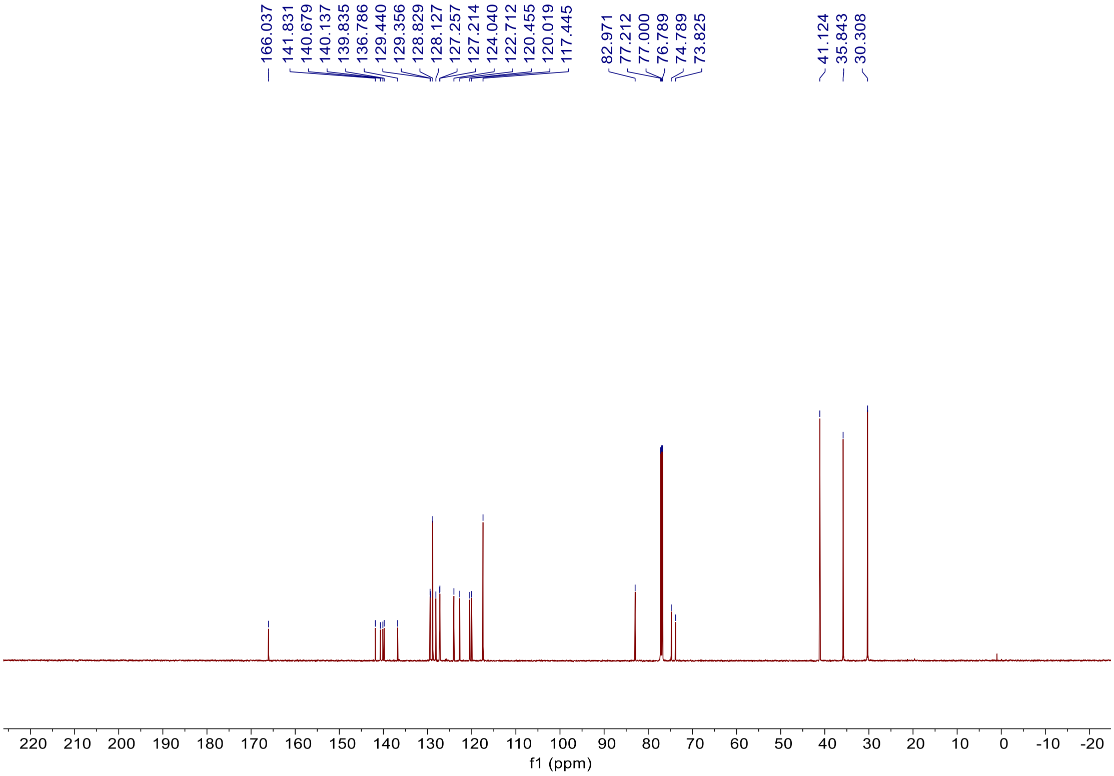

^{1}H NMR (400 MHz) Spectrum of 6h in $CDCl_3$

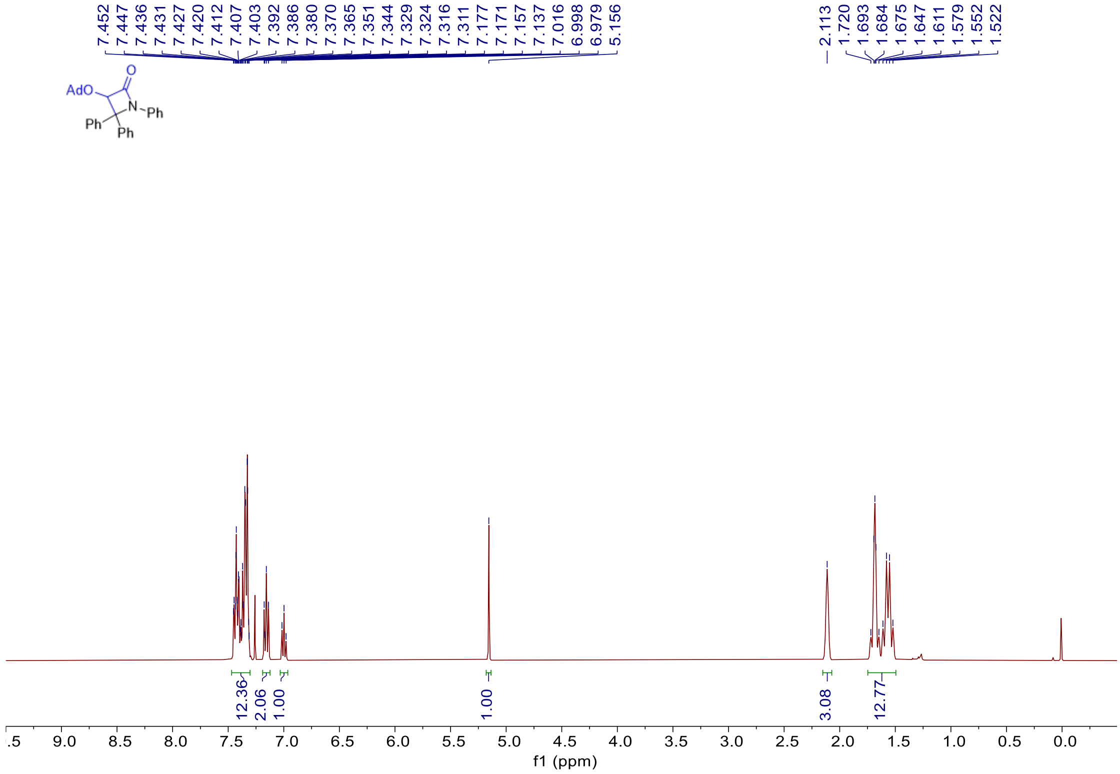

^{13}C NMR (100 MHz) Spectrum of 6h in $CDCl_3$

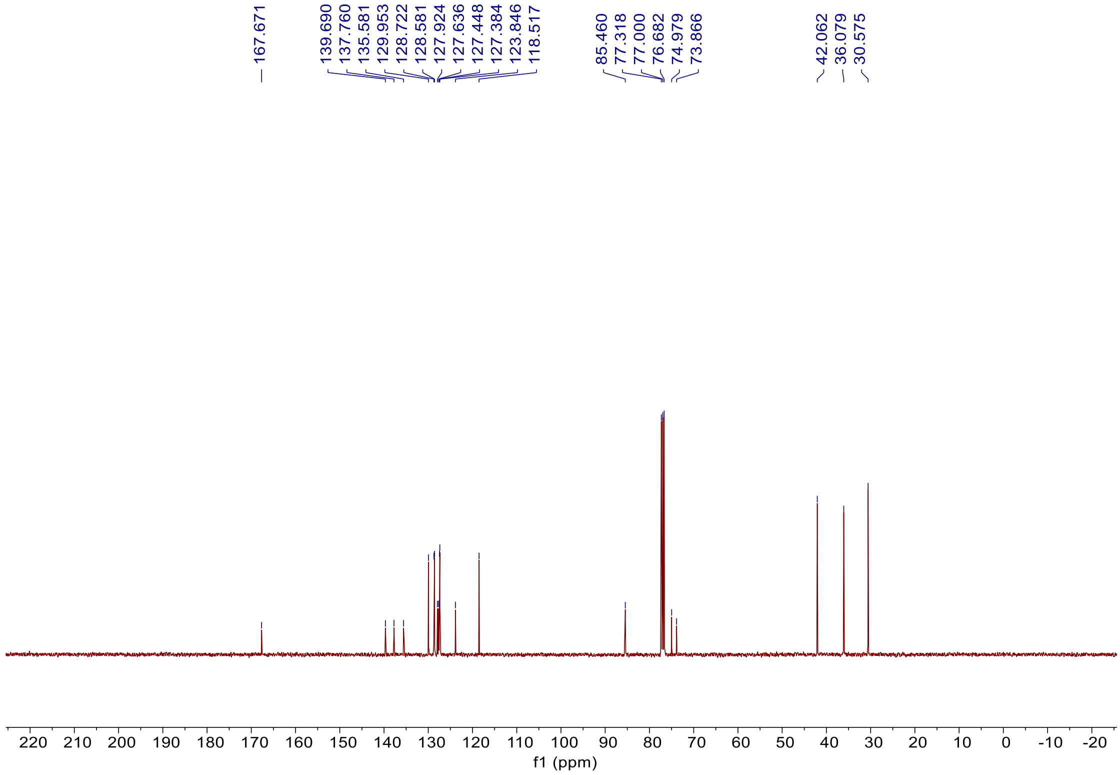

^{1}H NMR (400 MHz) Spectrum of 6i in CDCl$_3$

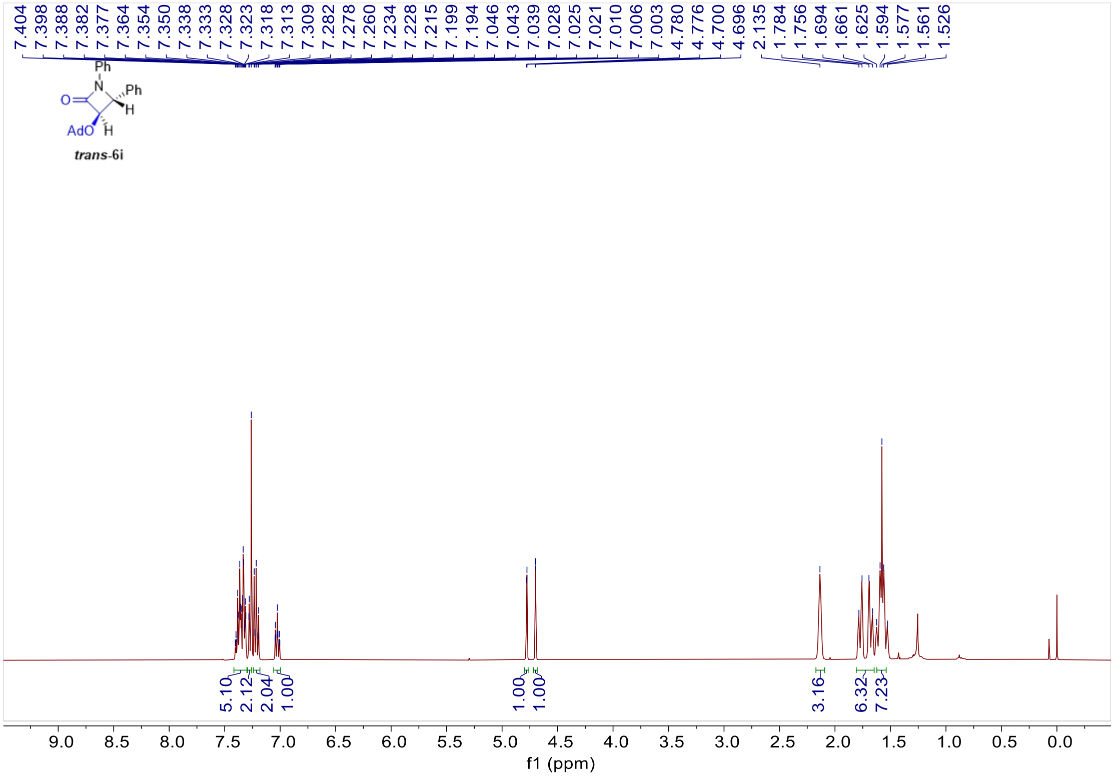

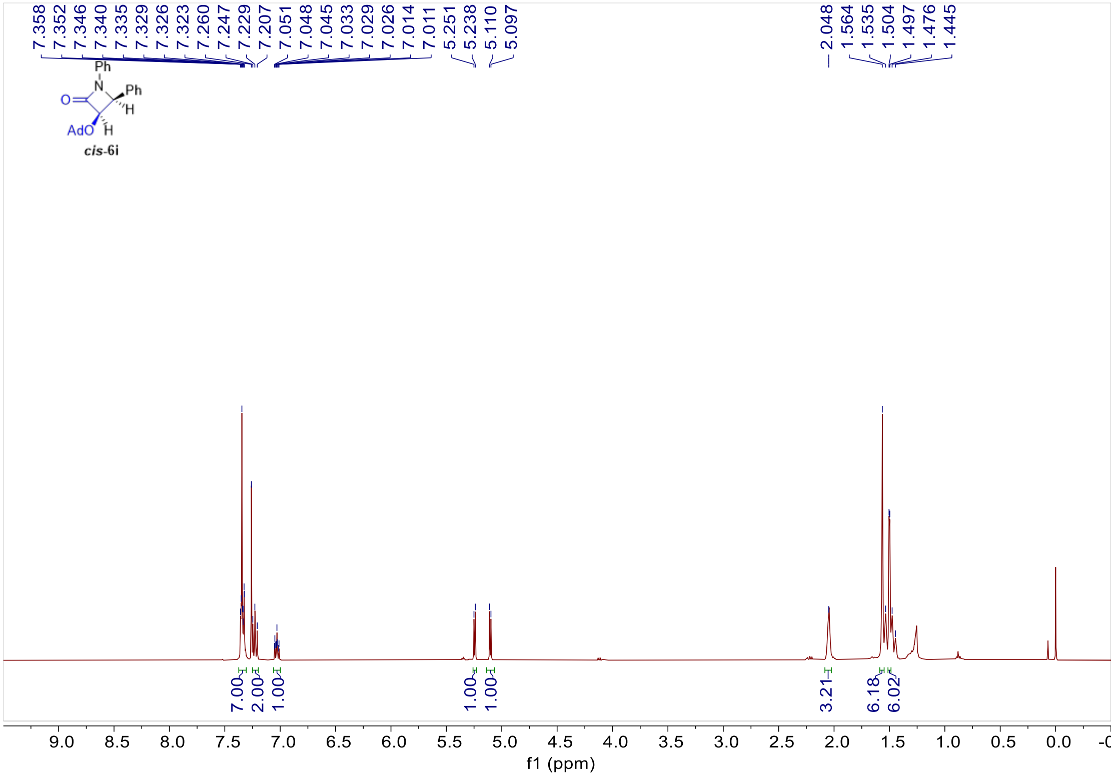

Partial crude ^{1}H NMR (400 MHz) Spectrum of 6i in $CDCl_3$ (*trans*: *cis* = 2: 1)

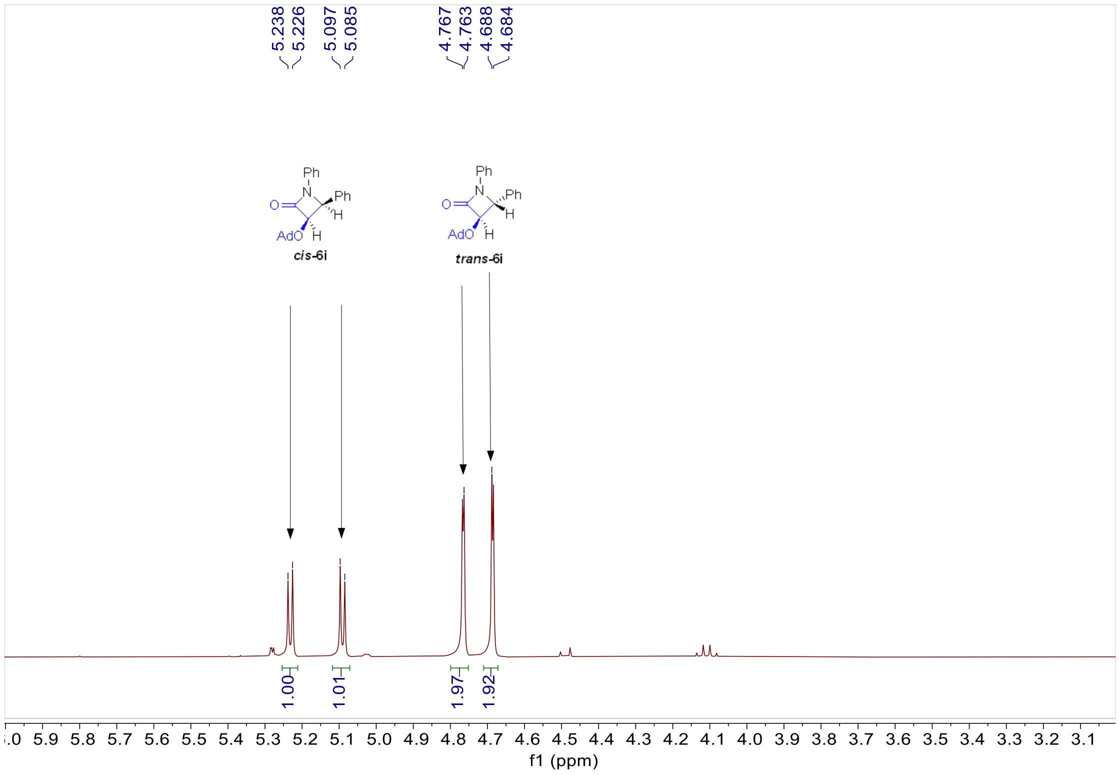

^{13}C NMR (100 MHz) Spectrum of 6i in $CDCl_3$

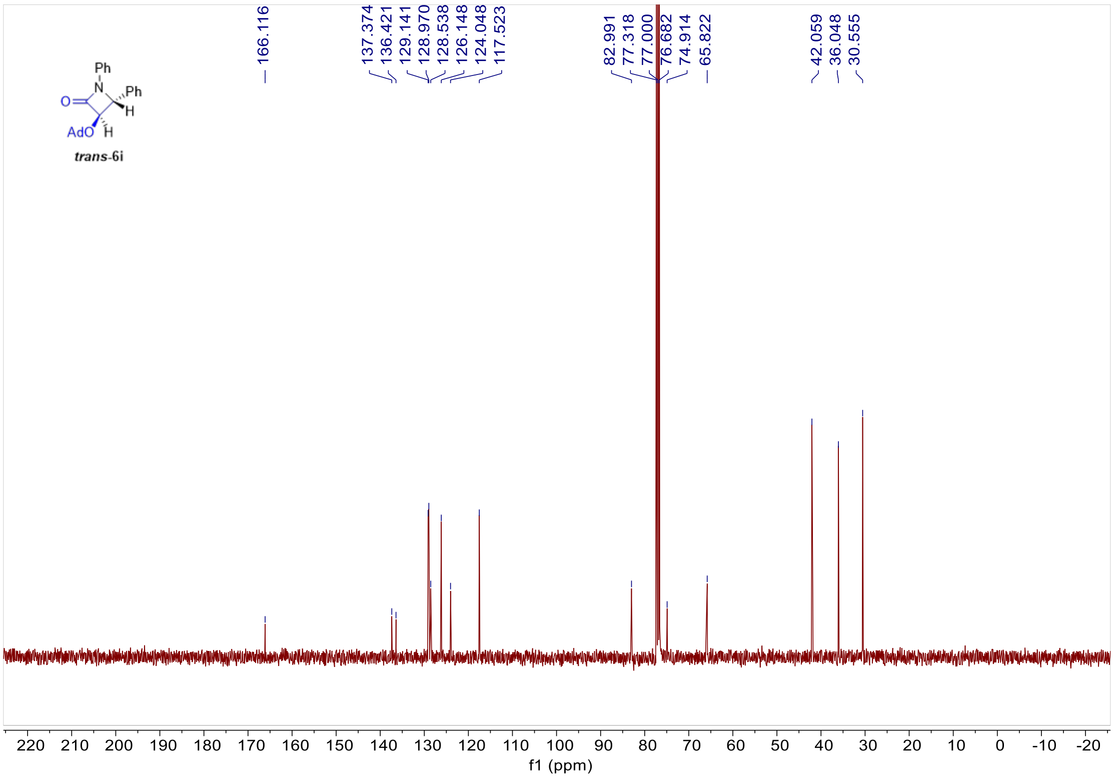

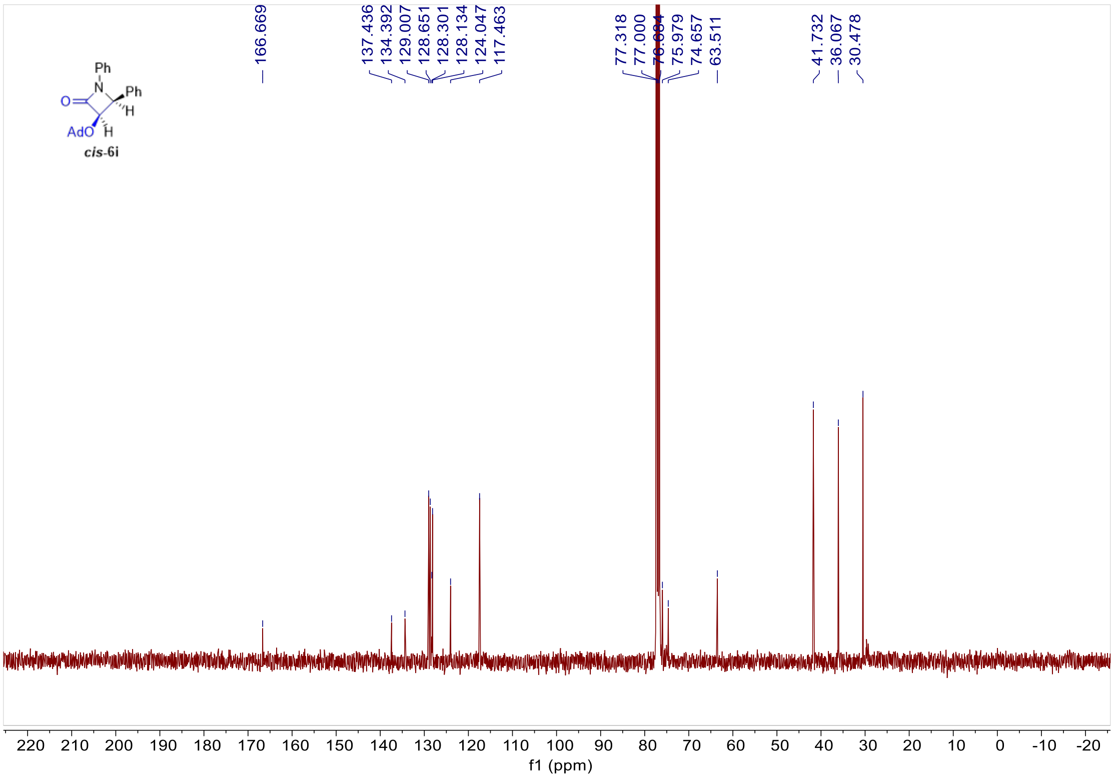

^{1}H NMR (400 MHz) Spectrum of 6j in $CDCl_3$

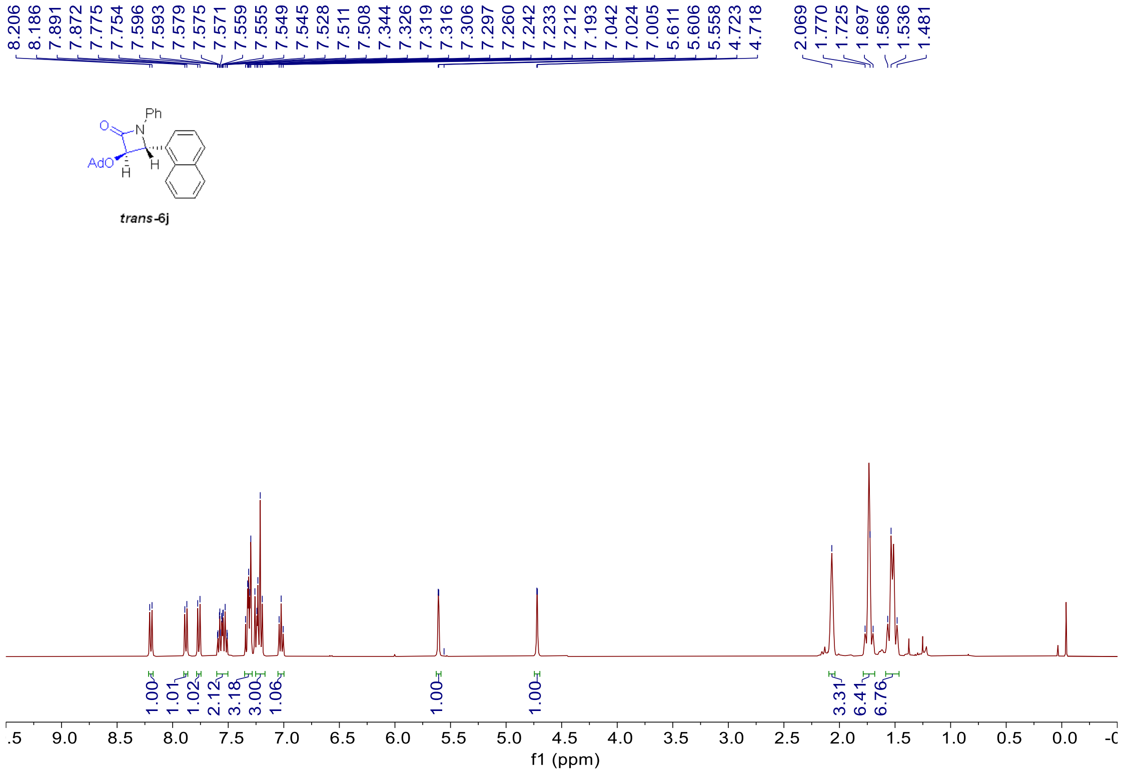

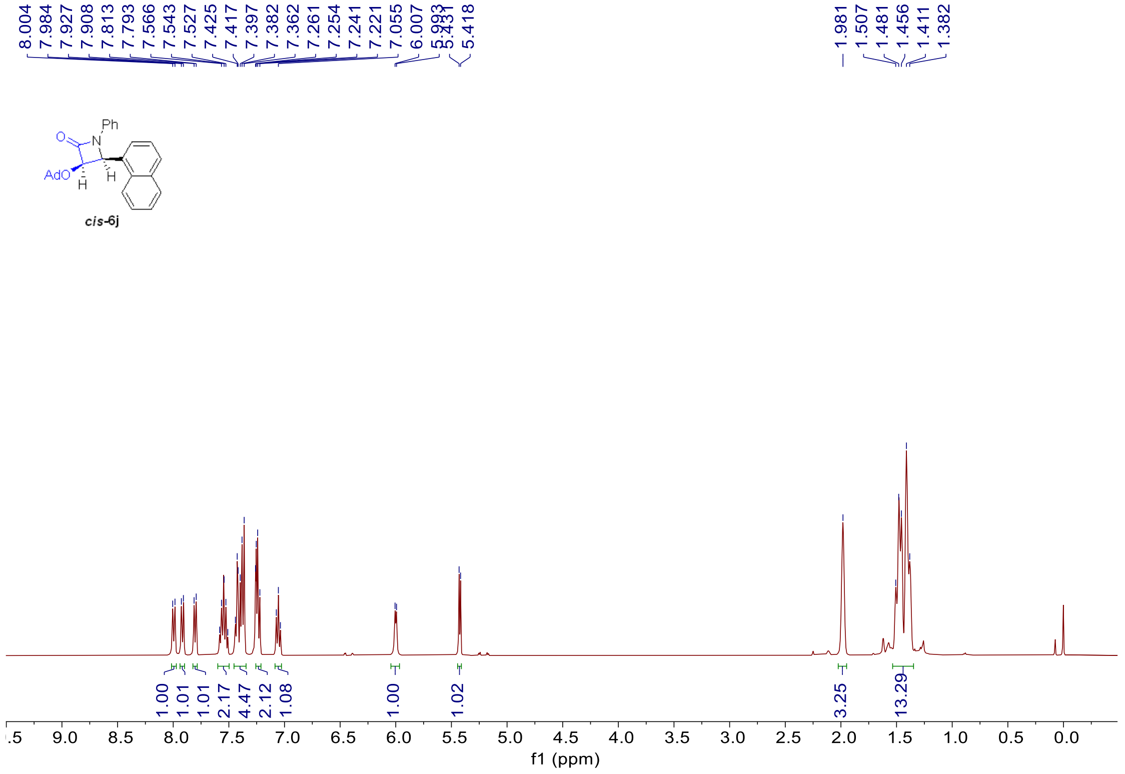

Partial crude ^{1}H NMR (400 MHz) Spectrum of 6j in $CDCl_3$ (*trans*: *cis* = 1: 1.3)

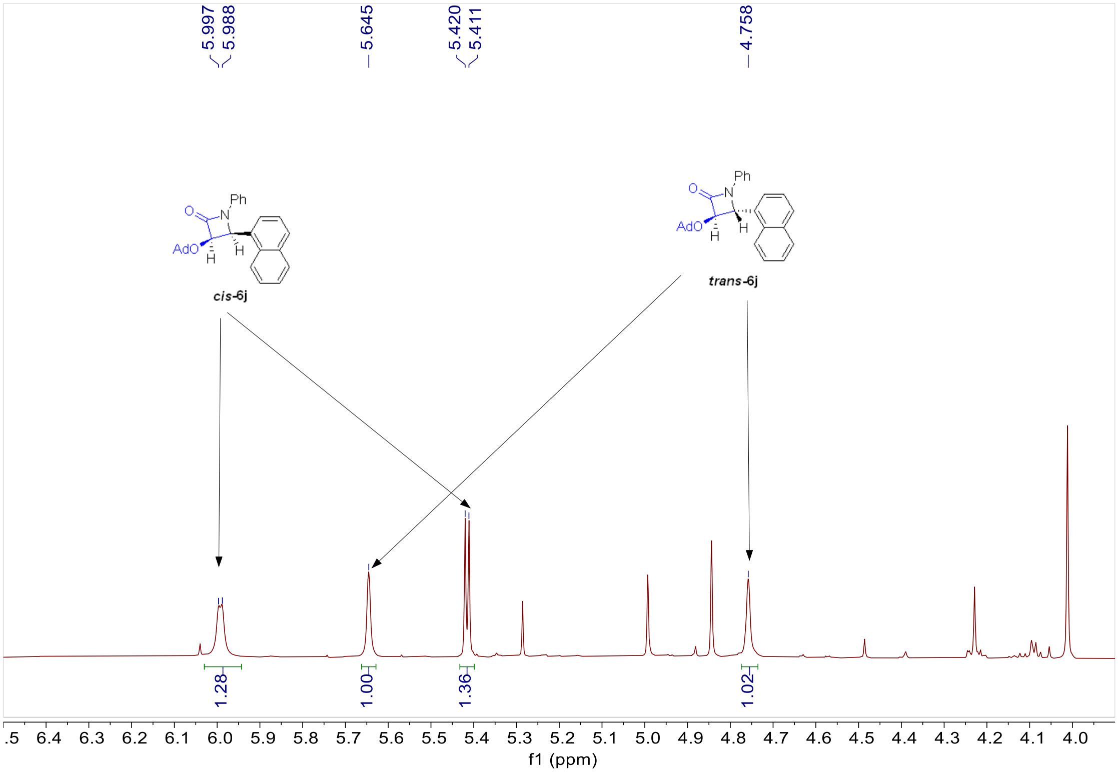

^{13}C NMR (100 MHz) Spectrum of 6j in $CDCl_3$

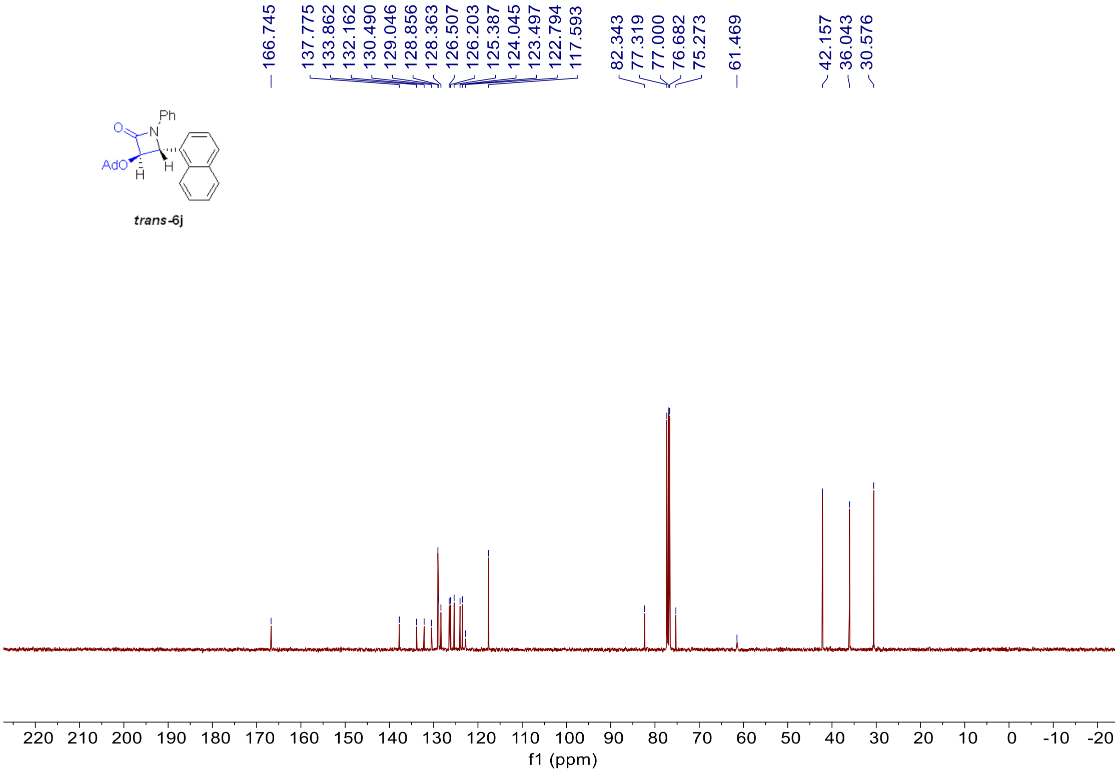

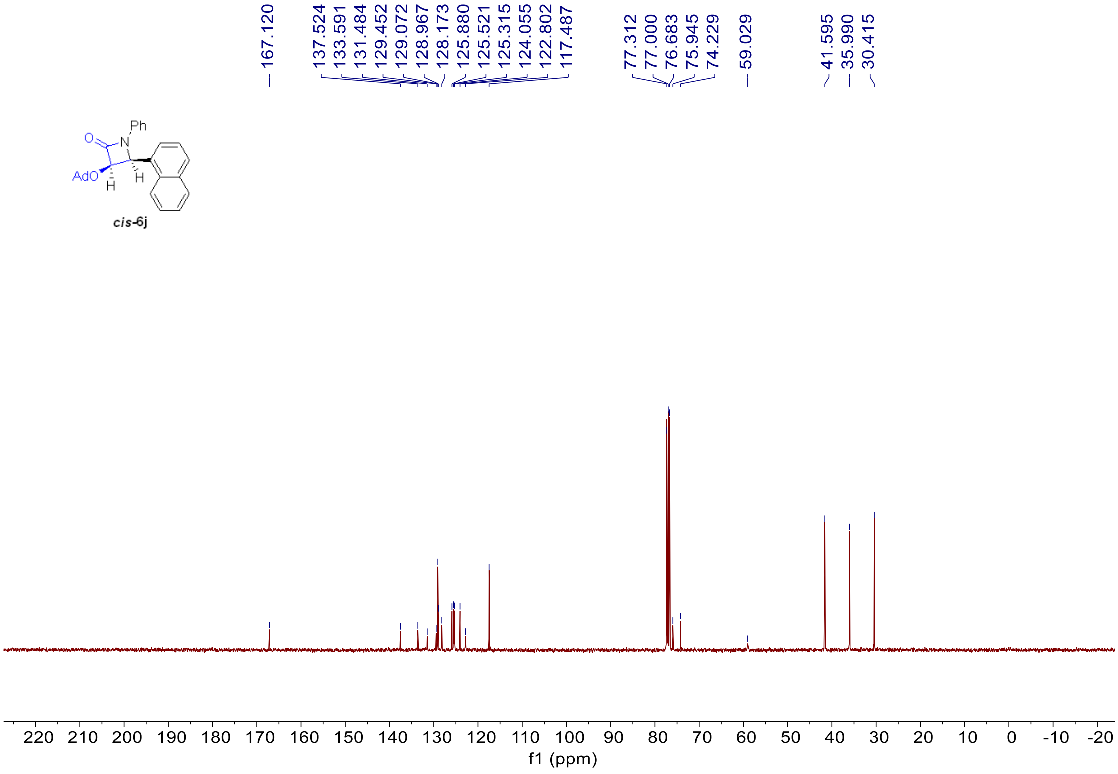

^{1}H NMR (400 MHz) Spectrum of 6k in $CDCl_3$

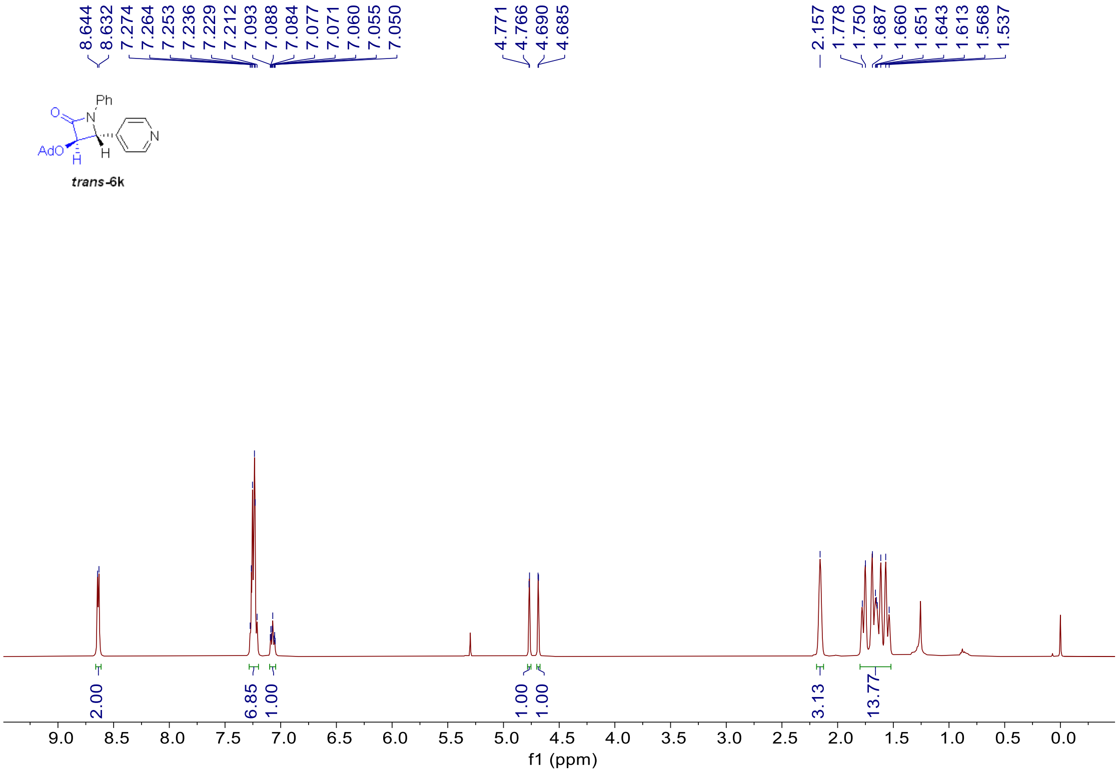

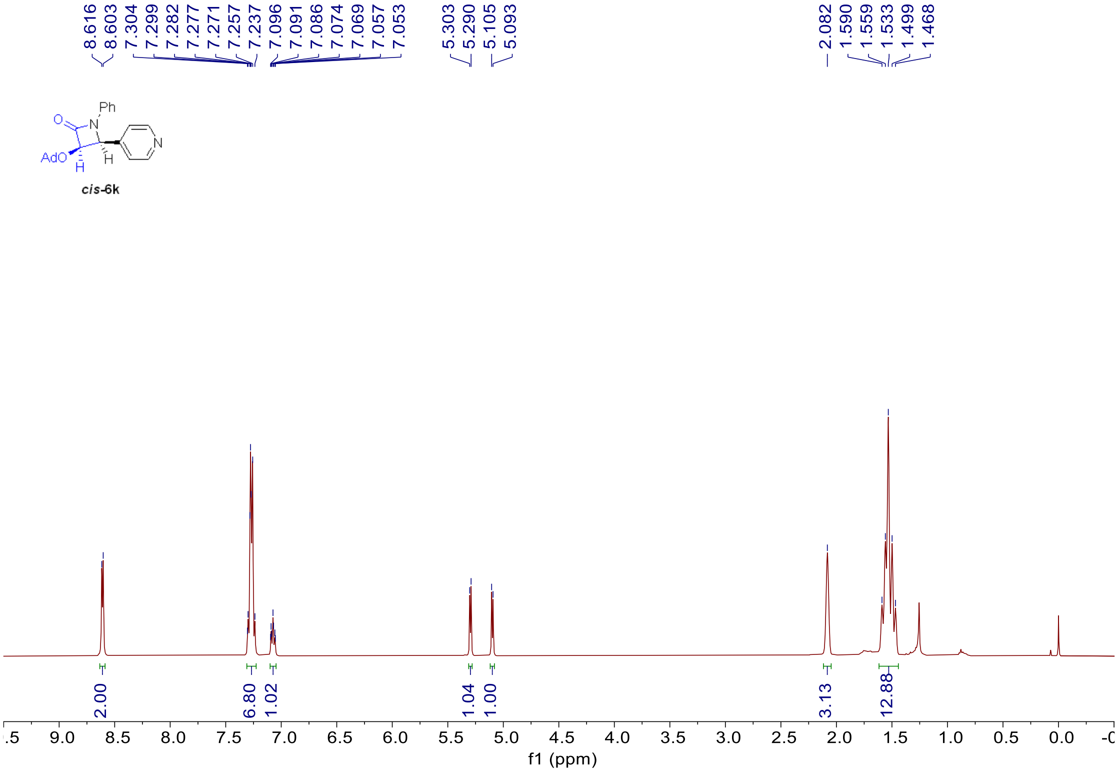

Partial crude ^{1}H NMR (400 MHz) Spectrum of 6k in $CDCl_3$ (*trans*: *cis* = 1.2: 1)

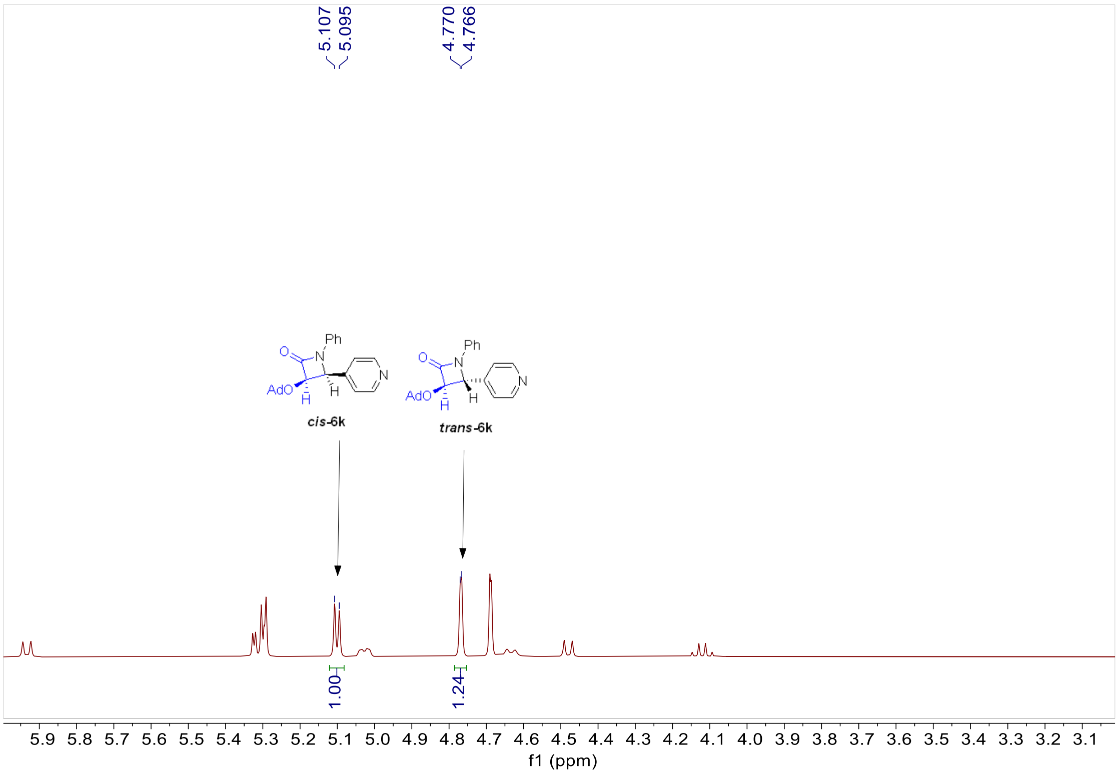

^{13}C NMR (100 MHz) Spectrum of 6k in $CDCl_3$

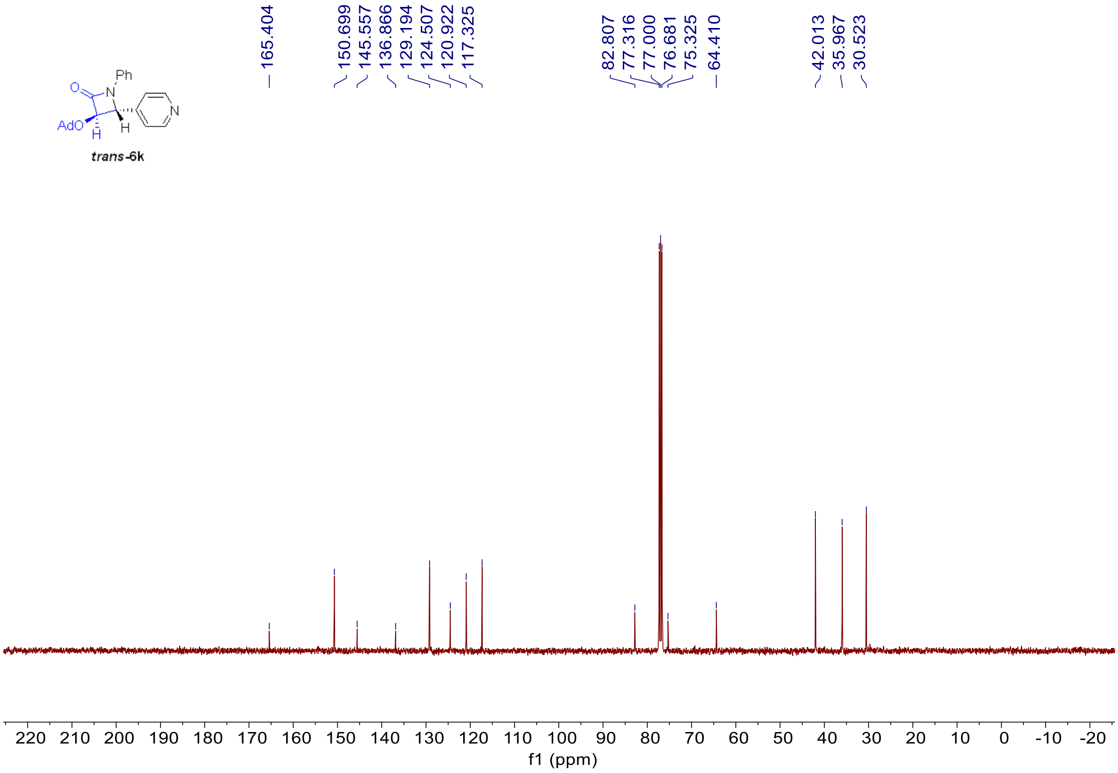

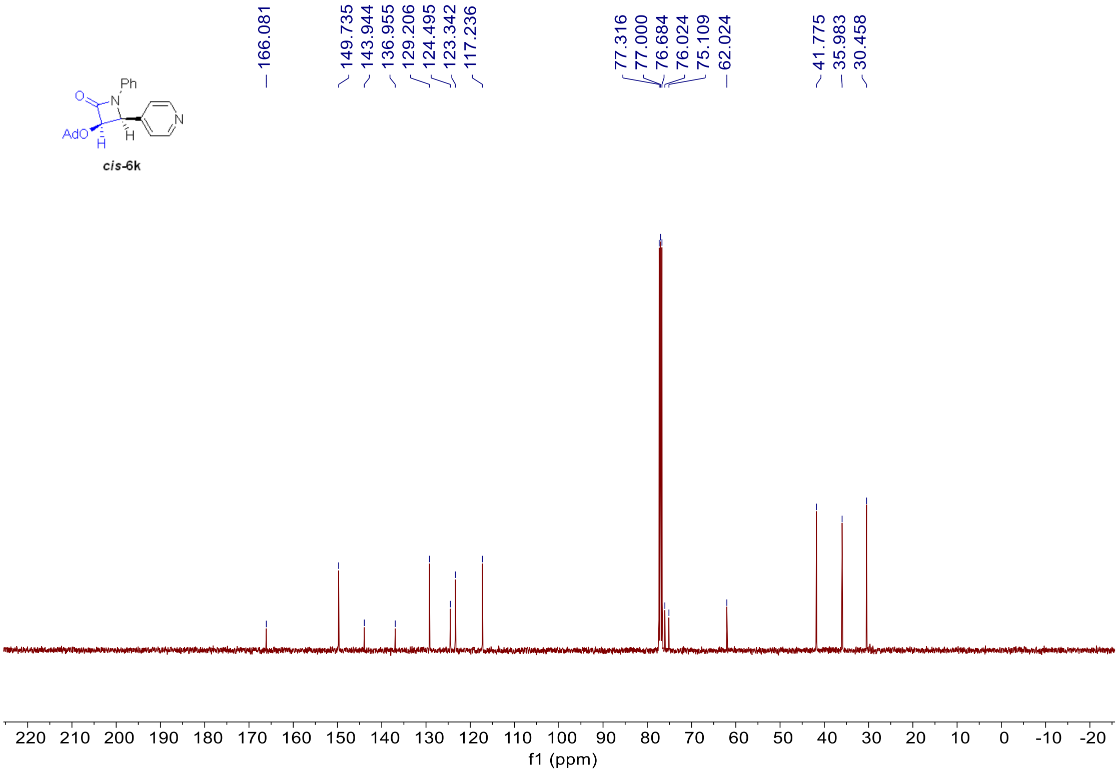

^{1}H NMR (400 MHz) Spectrum of 6l in $CDCl_3$

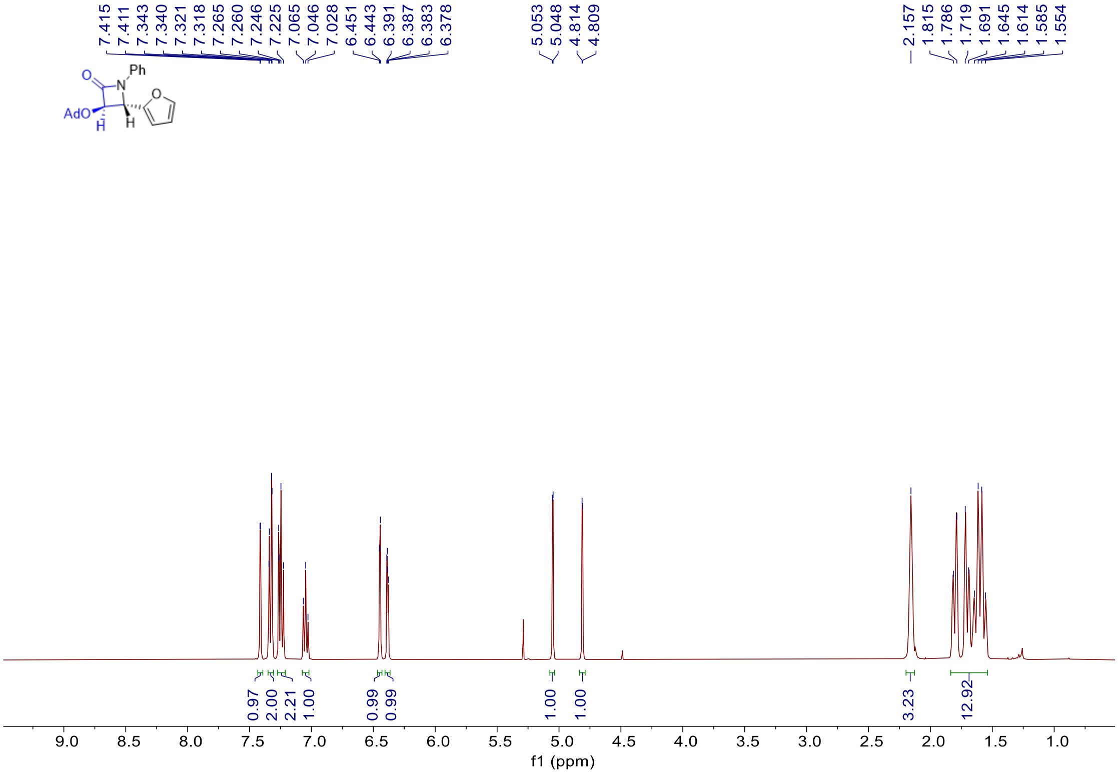

^{13}C NMR (100 MHz) Spectrum of 6l in $CDCl_3$

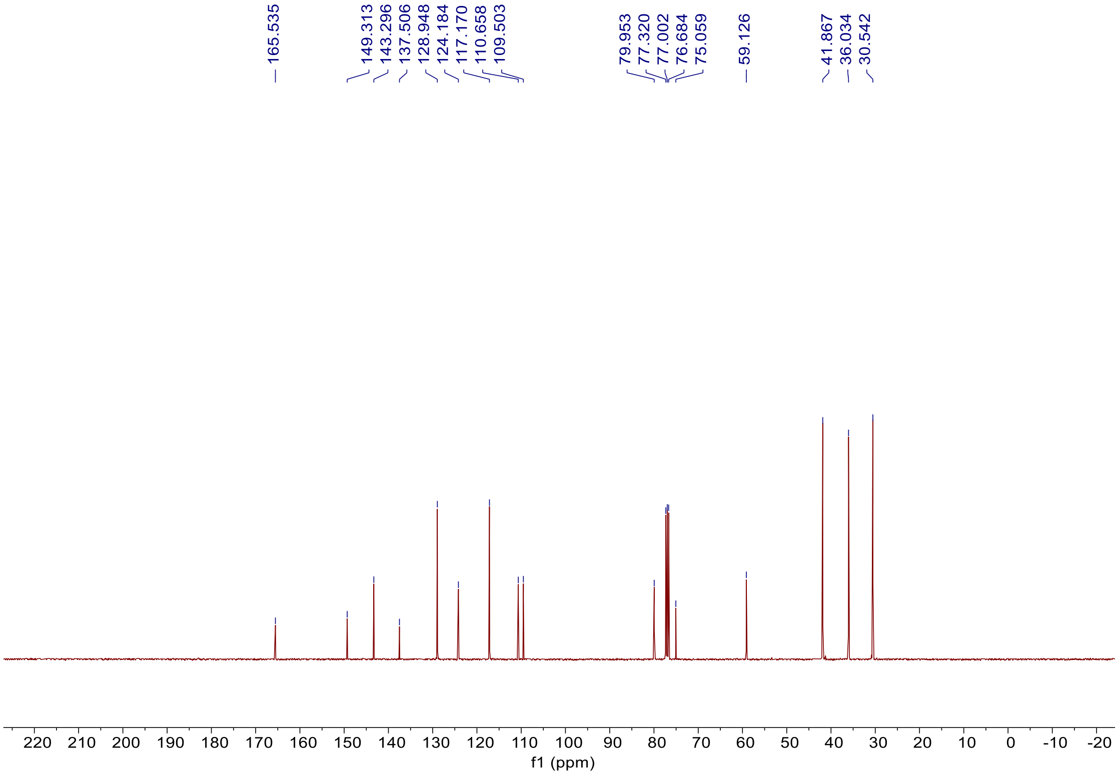

Partial crude ^{1}H NMR (400 MHz) Spectrum of 6l in $CDCl_3$ (*trans*: *cis* = 7: 1)

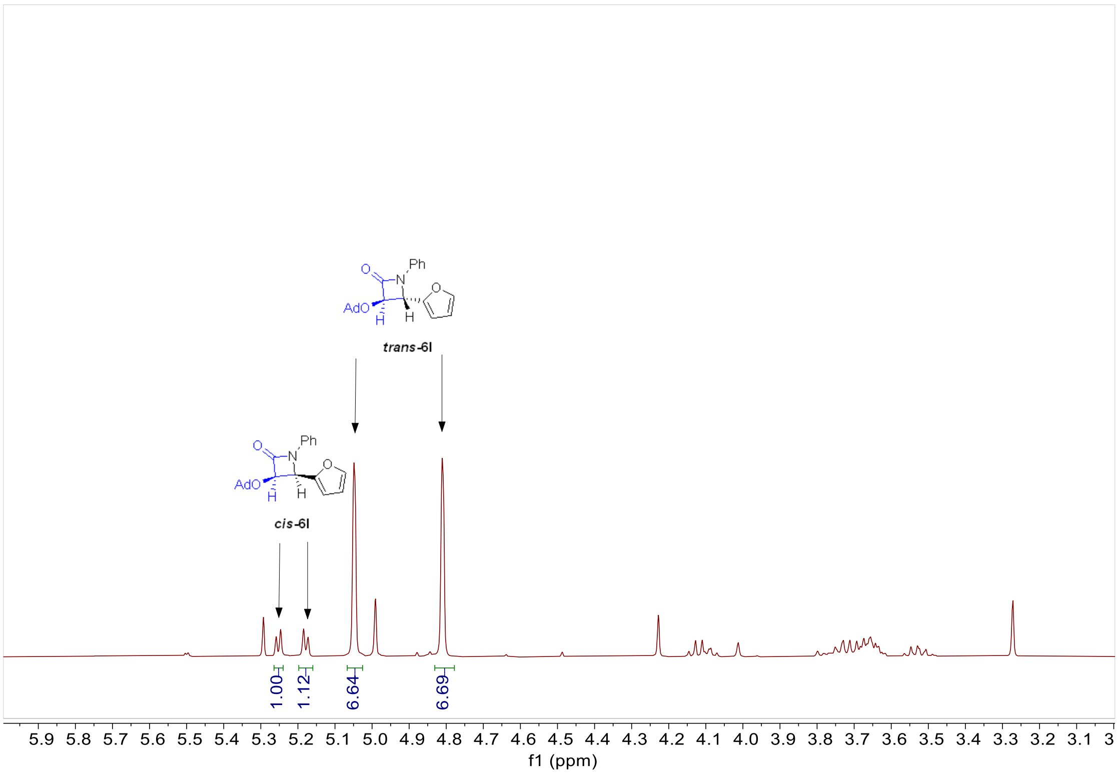

^{1}H NMR (600 MHz) Spectrum of 6m in CDCl$_3$

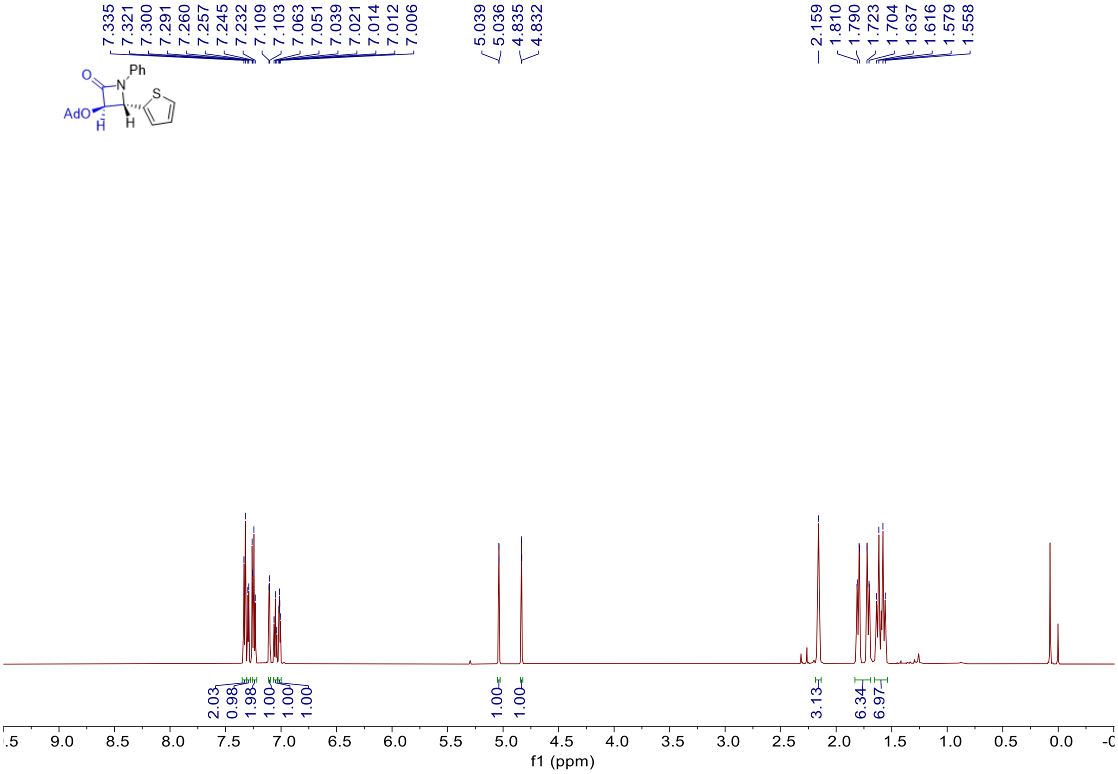

^{13}C NMR (150 MHz) Spectrum of 6m in CDCl$_3$

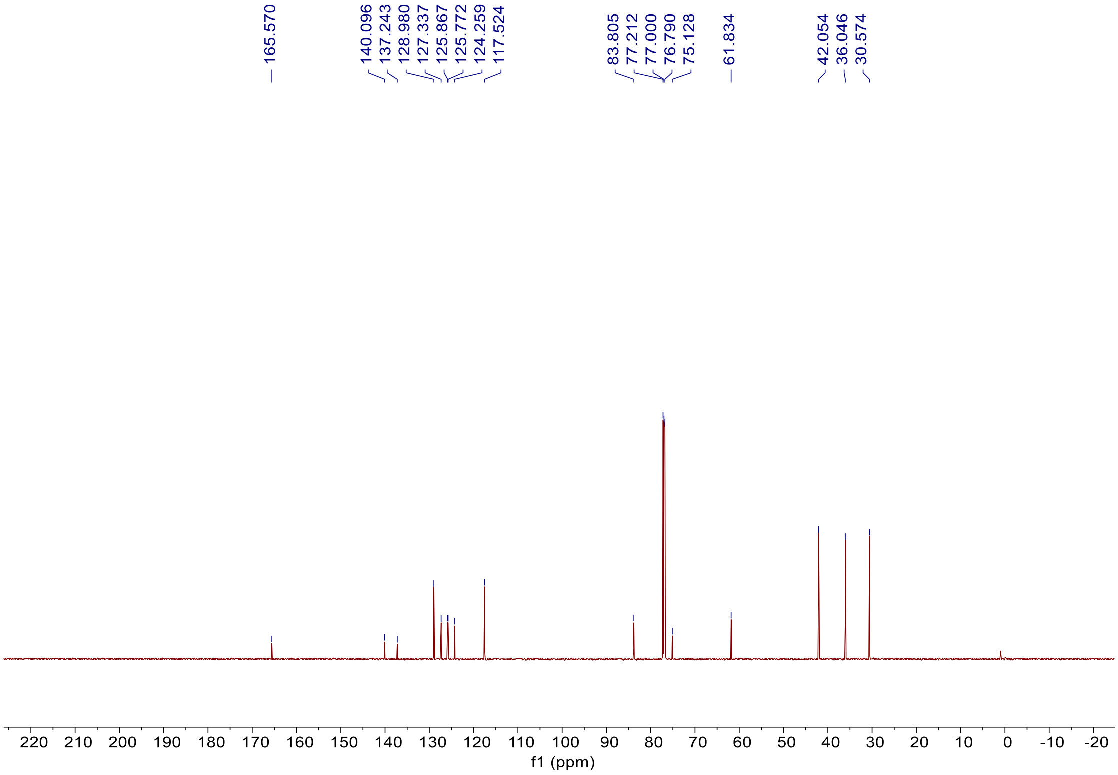

Partial crude ^1H NMR (400 MHz) Spectrum of 6m in $CDCl_3$ (*cis*-6m was not observed, according to the coupling constant)

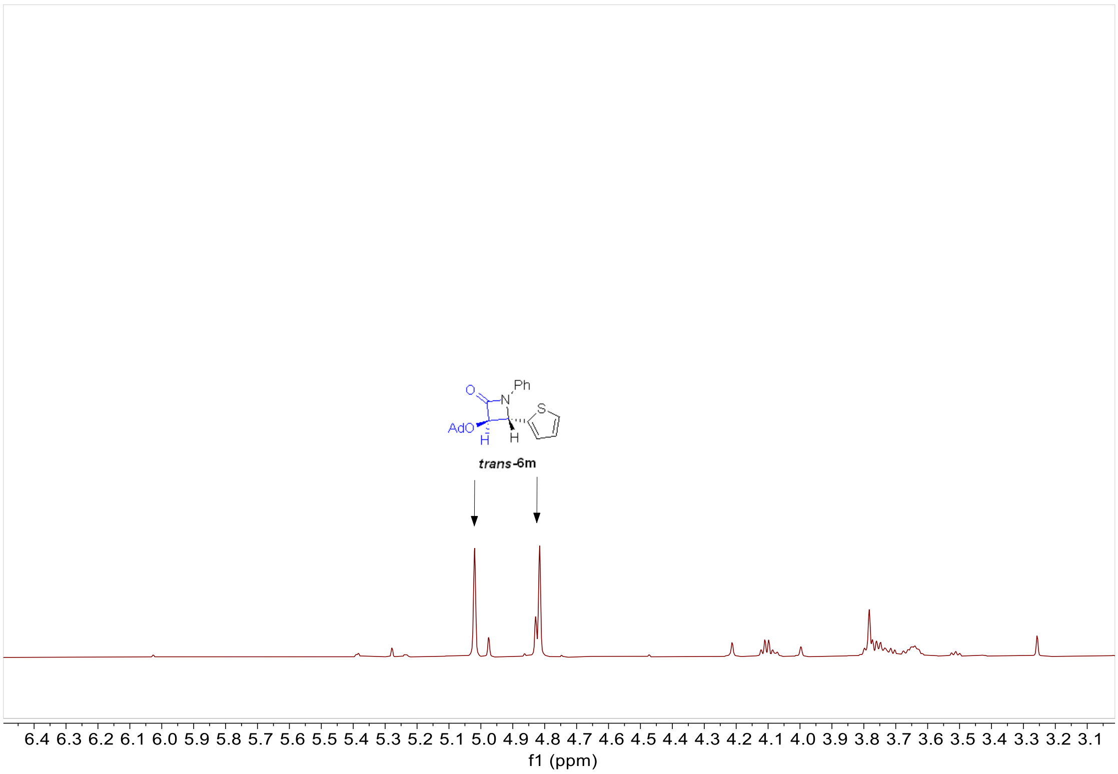

^{1}H NMR (400 MHz) Spectrum of 6n in $CDCl_3$

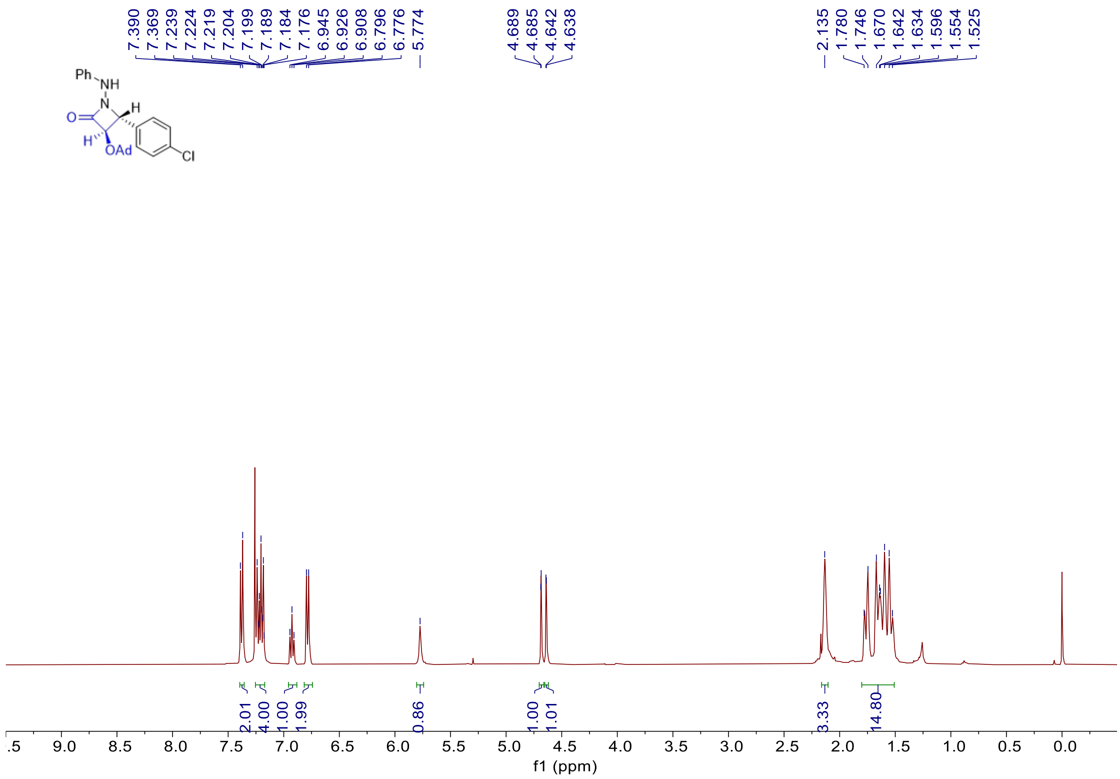

^{13}C NMR (100 MHz) Spectrum of 6n in $CDCl_3$

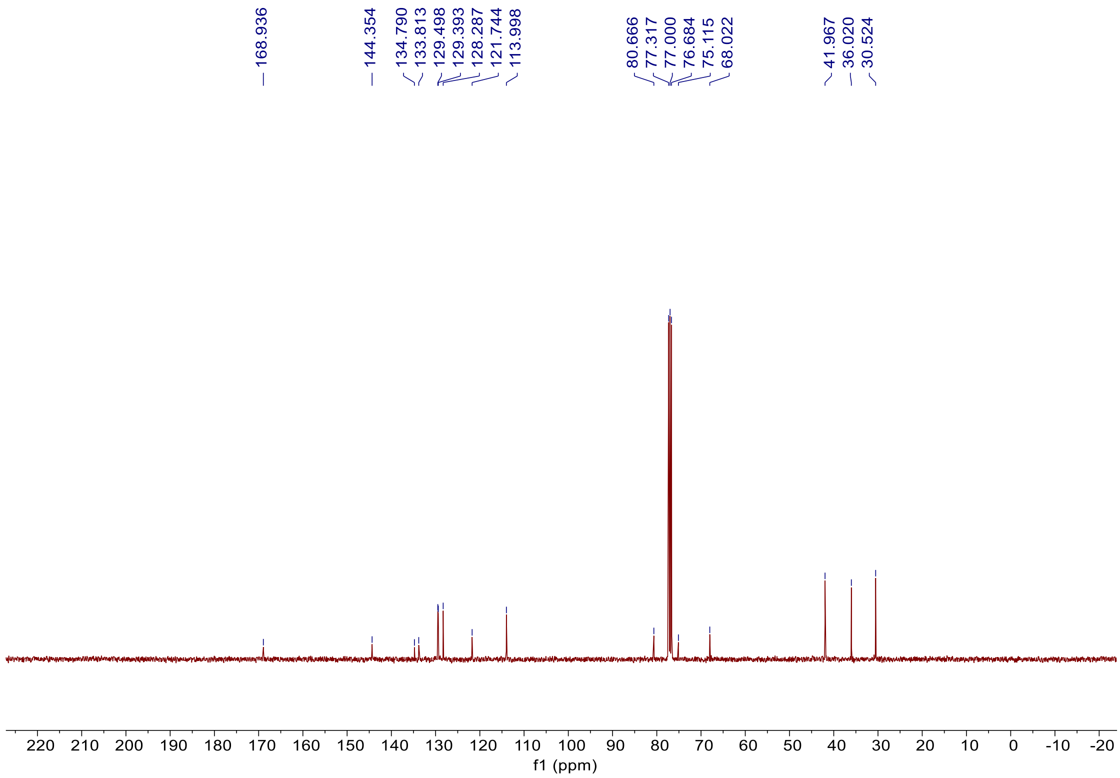

^{1}H NMR (400 MHz) Spectrum of 7 in $CDCl_3$

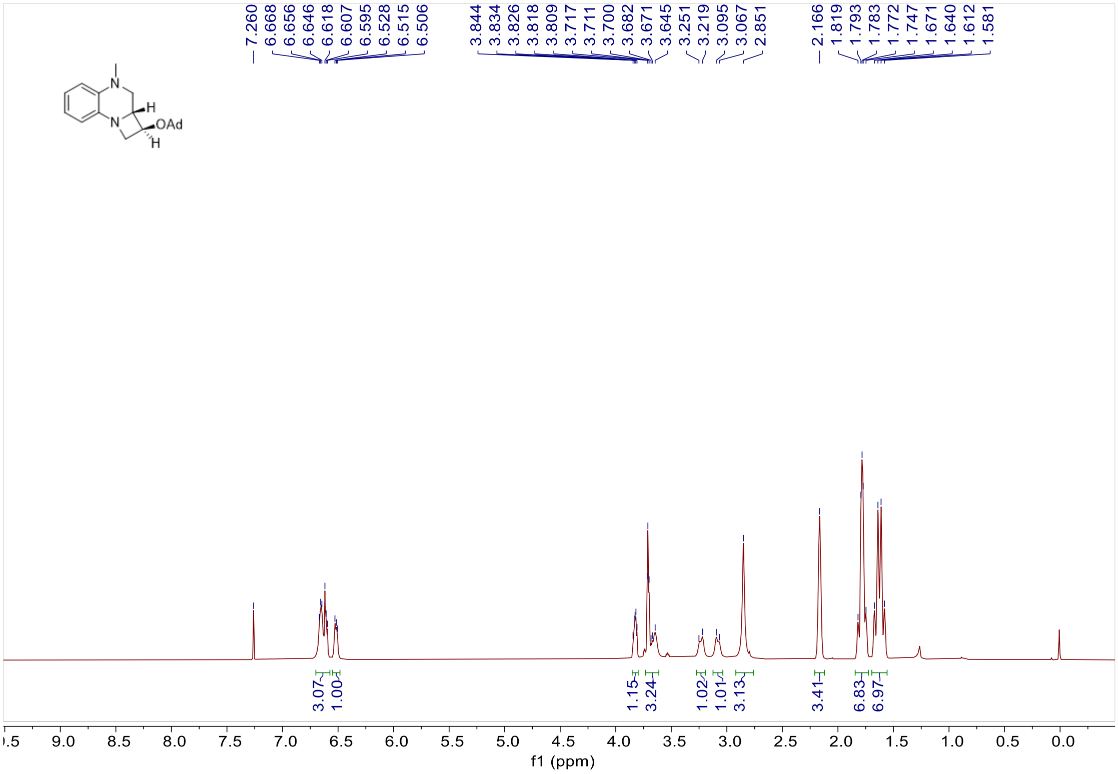

^{13}C NMR (100 MHz) Spectrum of 7 in $CDCl_3$

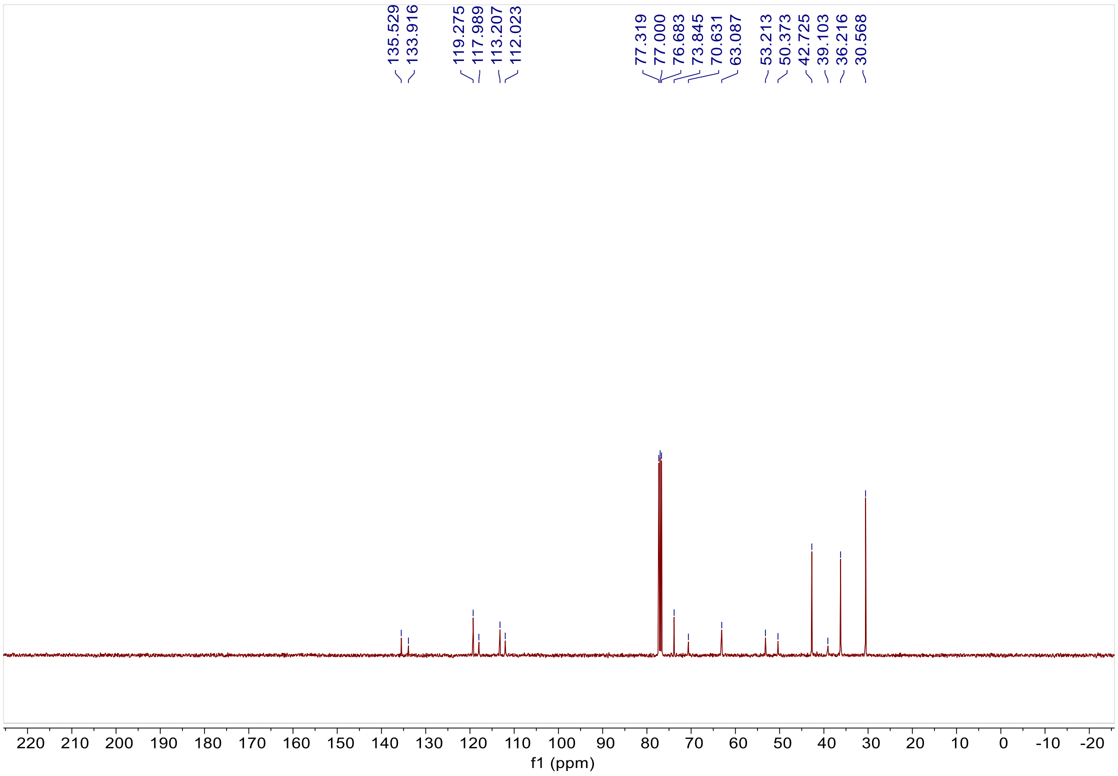

^{1}H NMR (600 MHz) Spectrum of 8 in $CDCl_3$

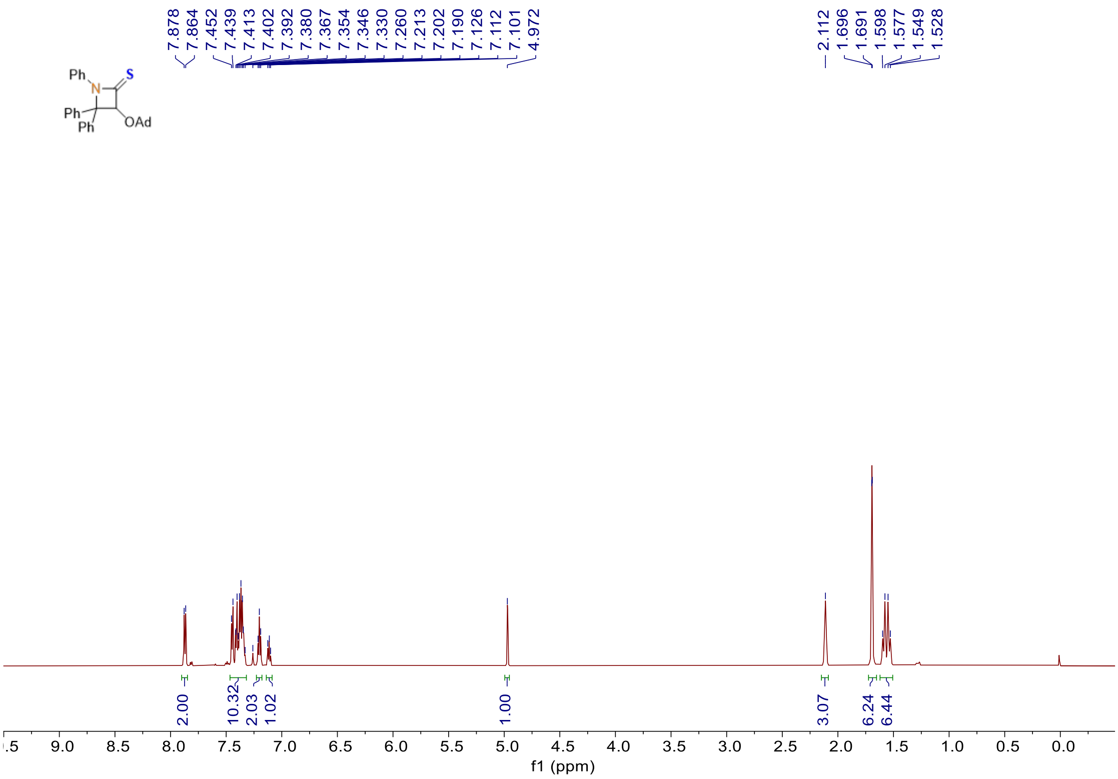

^{13}C NMR (150 MHz) Spectrum of 8 in $CDCl_3$

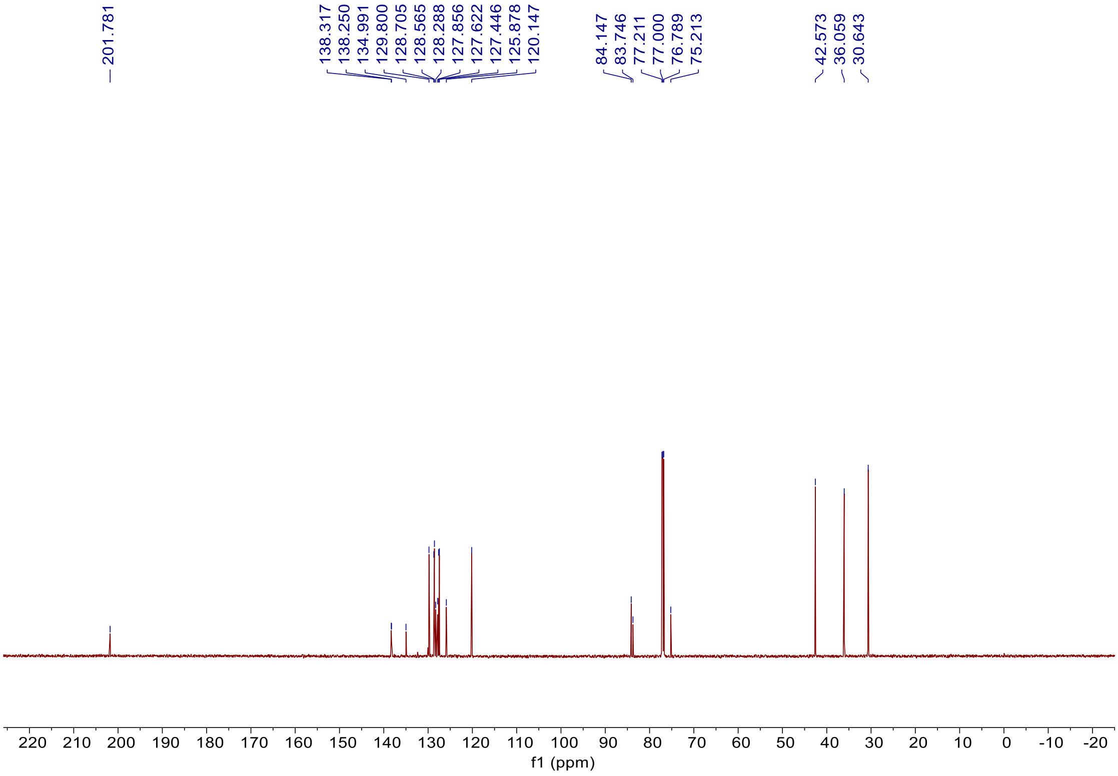

^{1}H NMR (400 MHz) Spectrum of 3a-D1 in $CDCl_3$

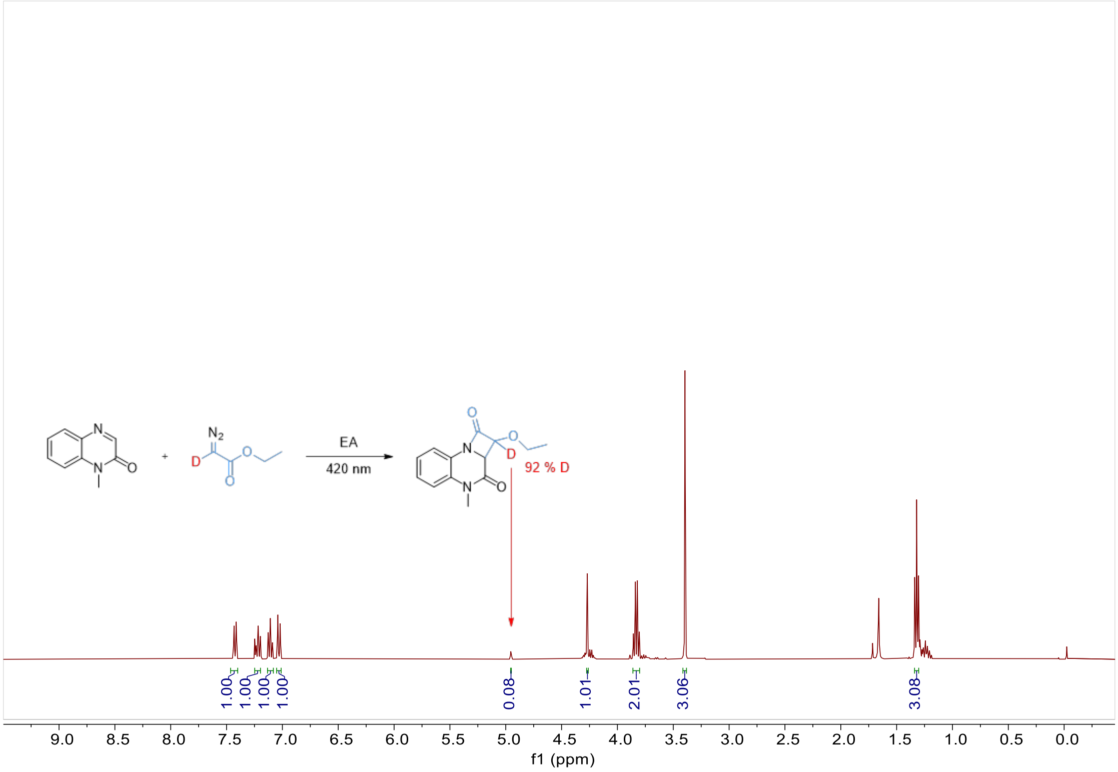

^{1}H NMR (400 MHz) Spectrum of 2a-D1 in $CDCl_3$

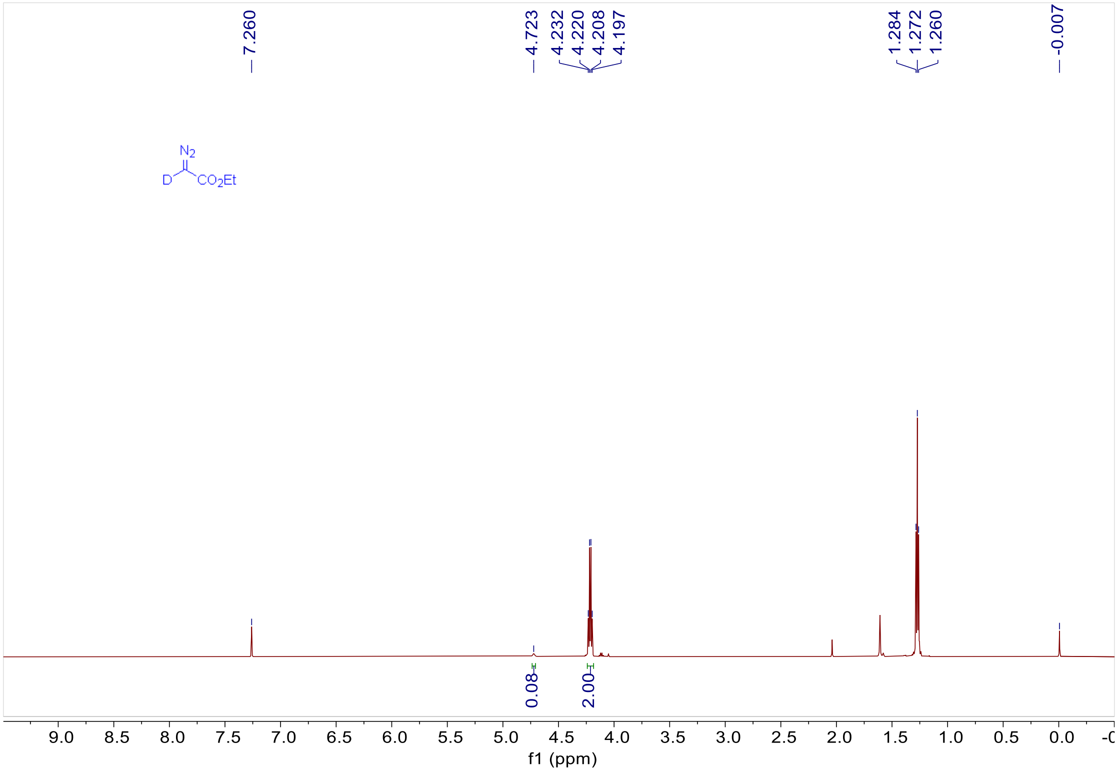

^{1}H NMR (600 MHz) Spectrum of 10 in $CDCl_3$

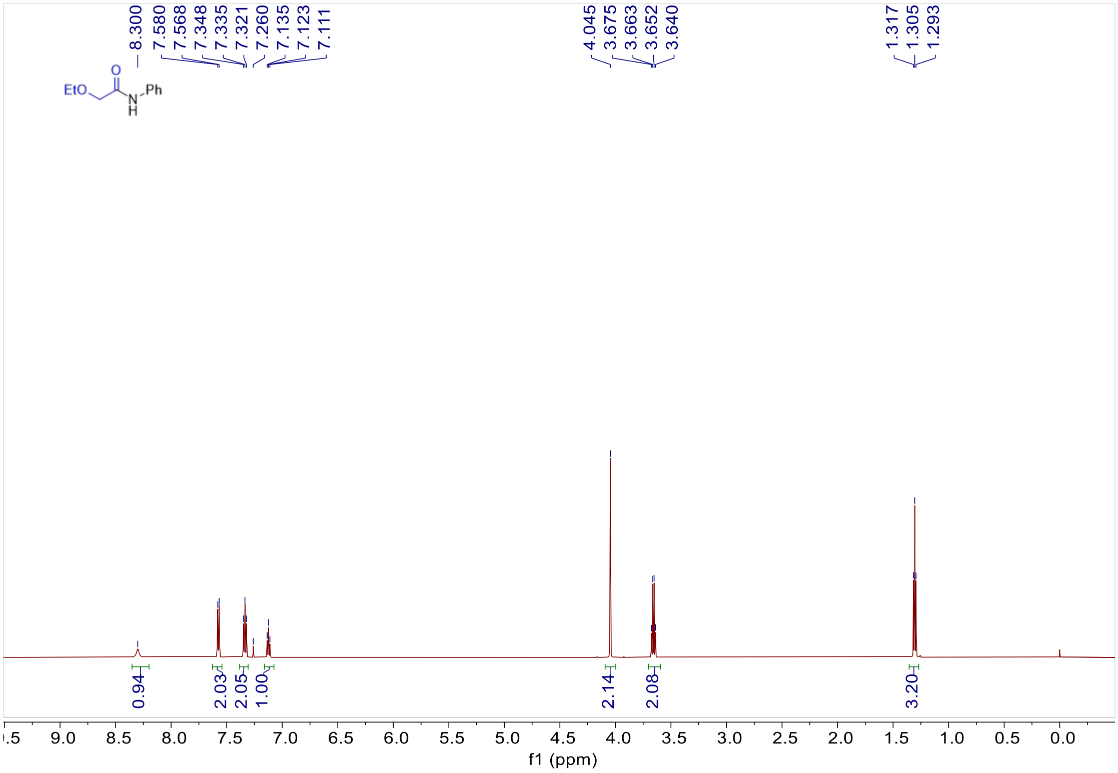

^{13}C NMR (150 MHz) Spectrum of 10 in $CDCl_3$

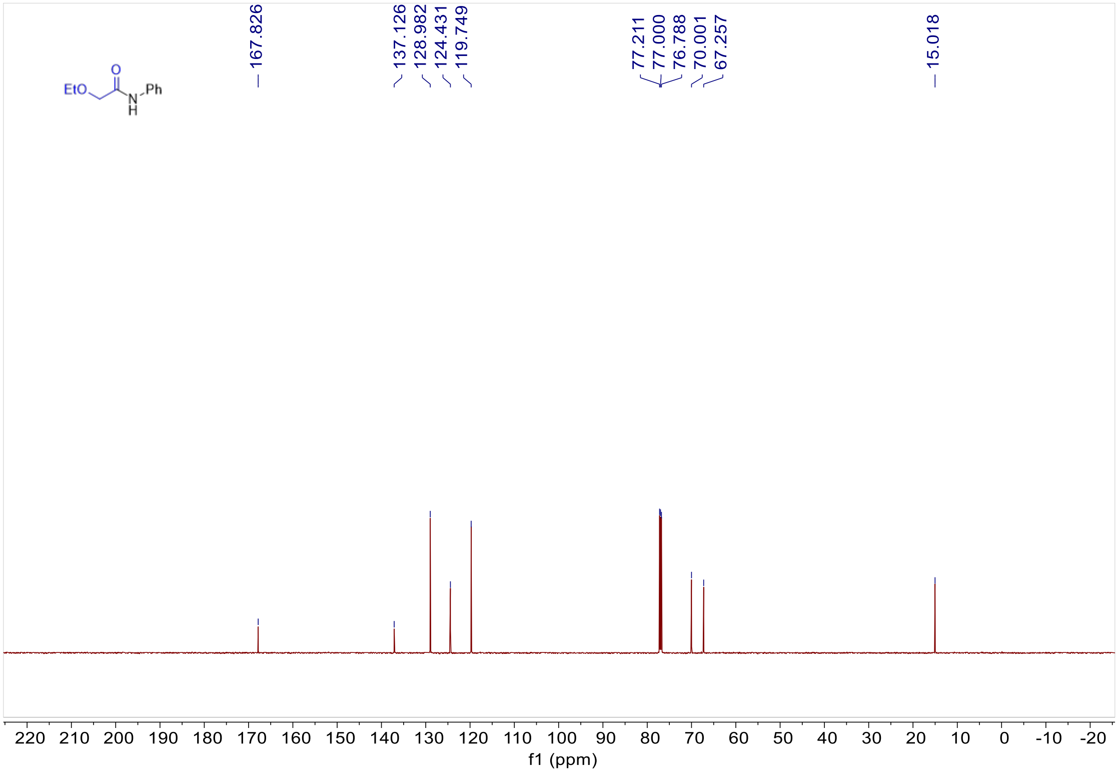

^{1}H NMR (600 MHz) Spectrum of 11 in $CDCl_3$

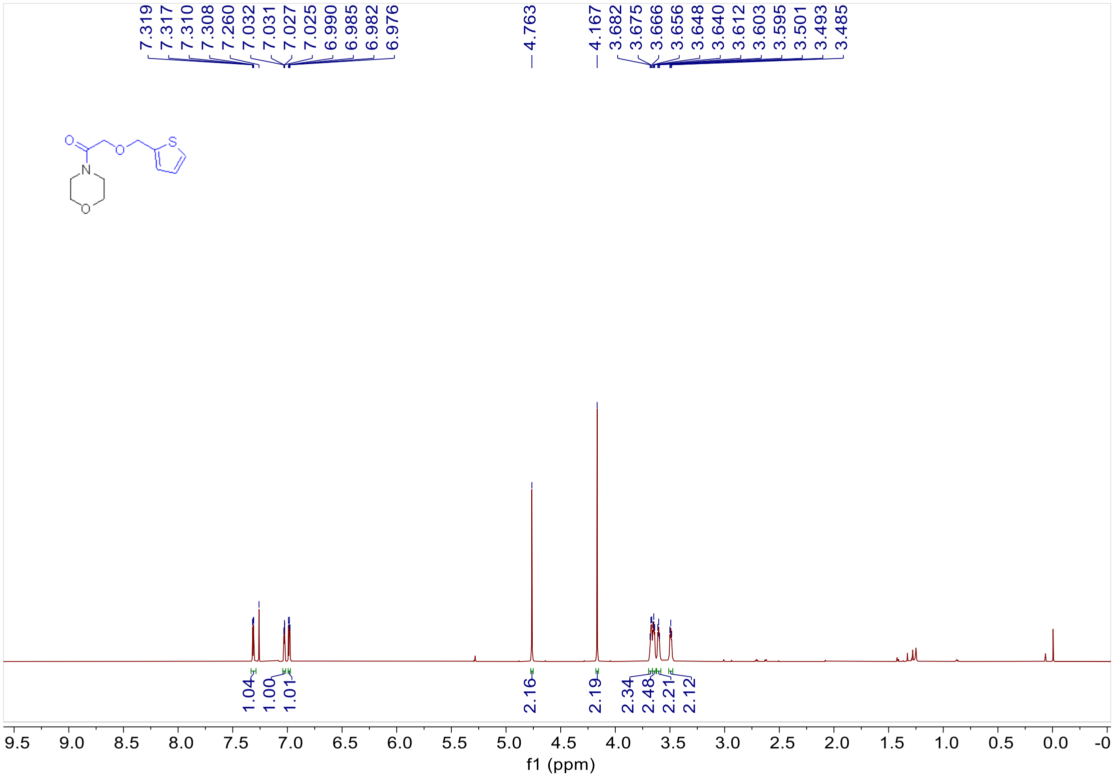

^{13}C NMR (150 MHz) Spectrum of 11 in $CDCl_3$

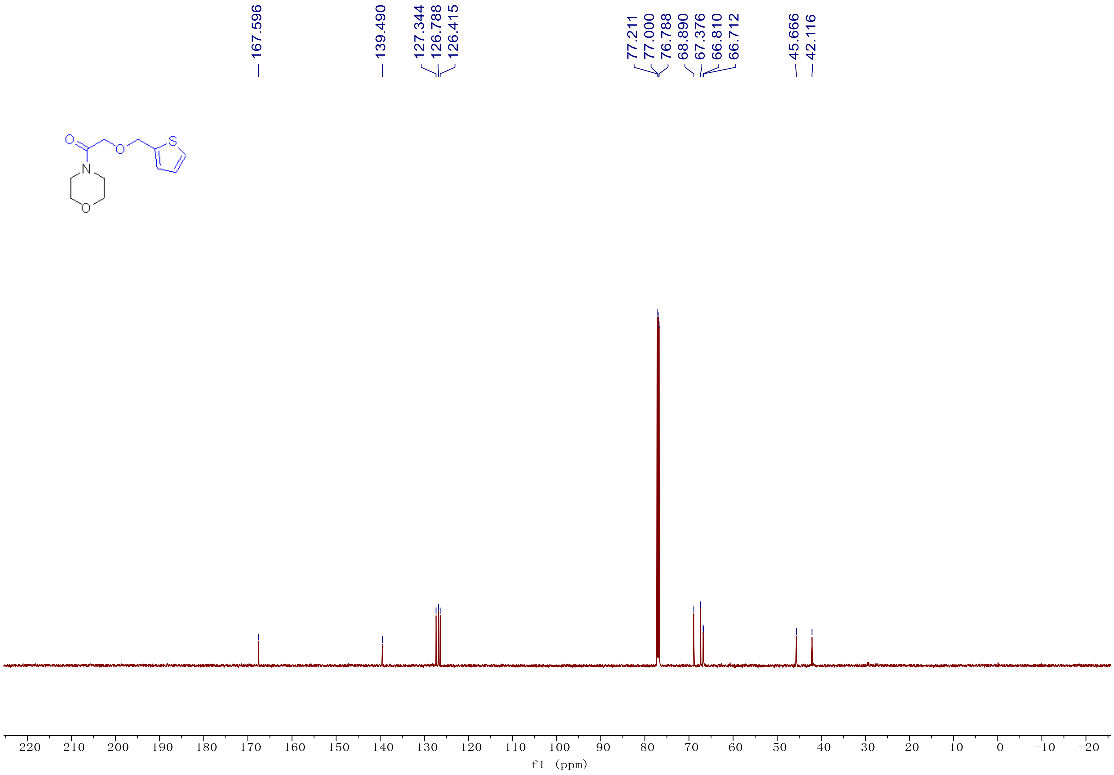

^{1}H NMR (600 MHz) Spectrum of 12 in CDCl$_3$

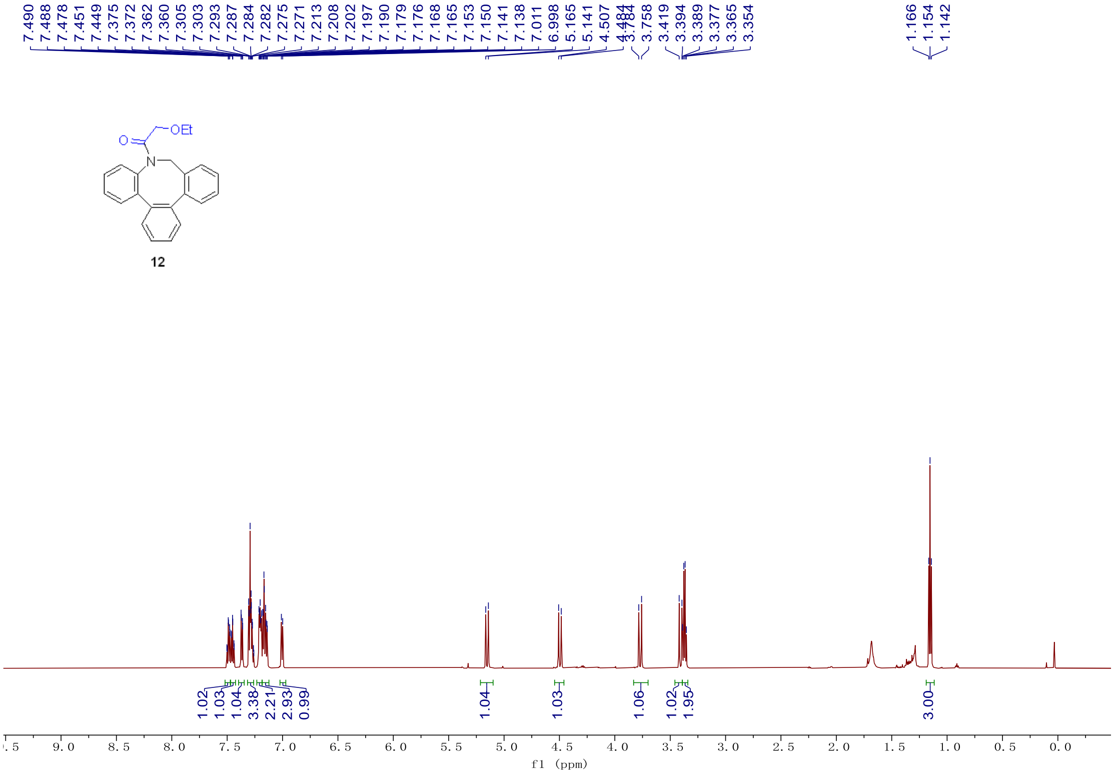

^{13}C NMR (150 MHz) Spectrum of 12 in CDCl$_3$

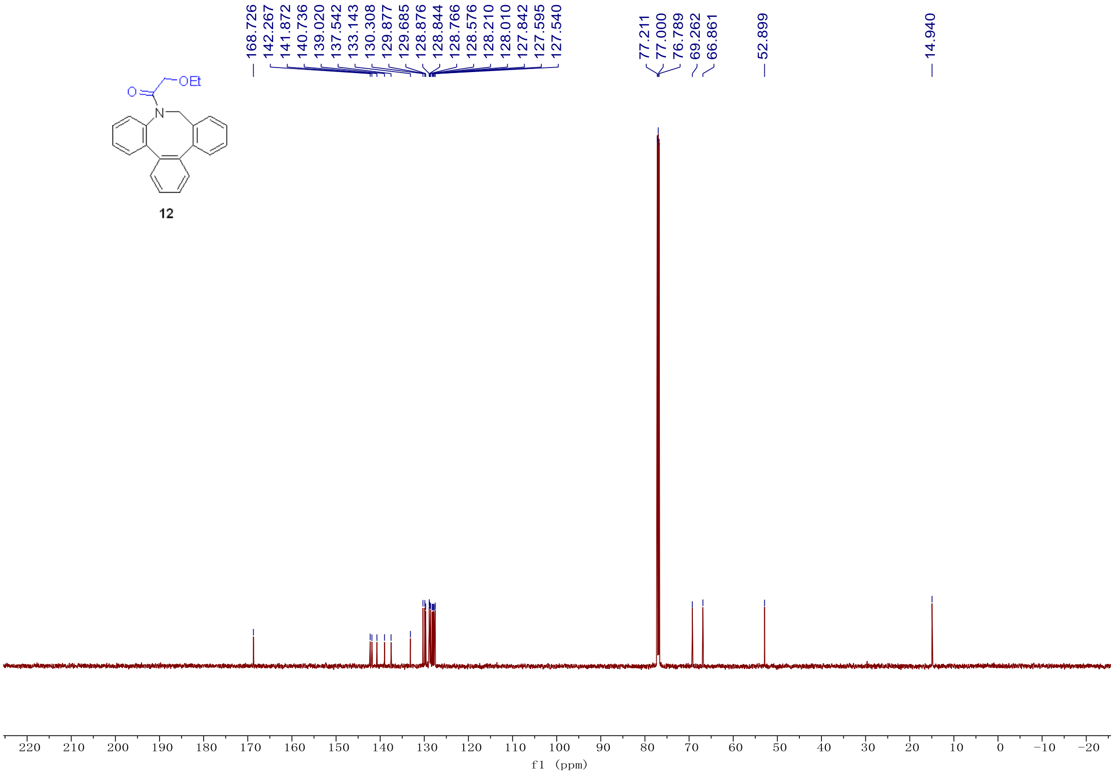

^{1}H NMR (400 MHz) Spectrum of 14 in $CDCl_3$

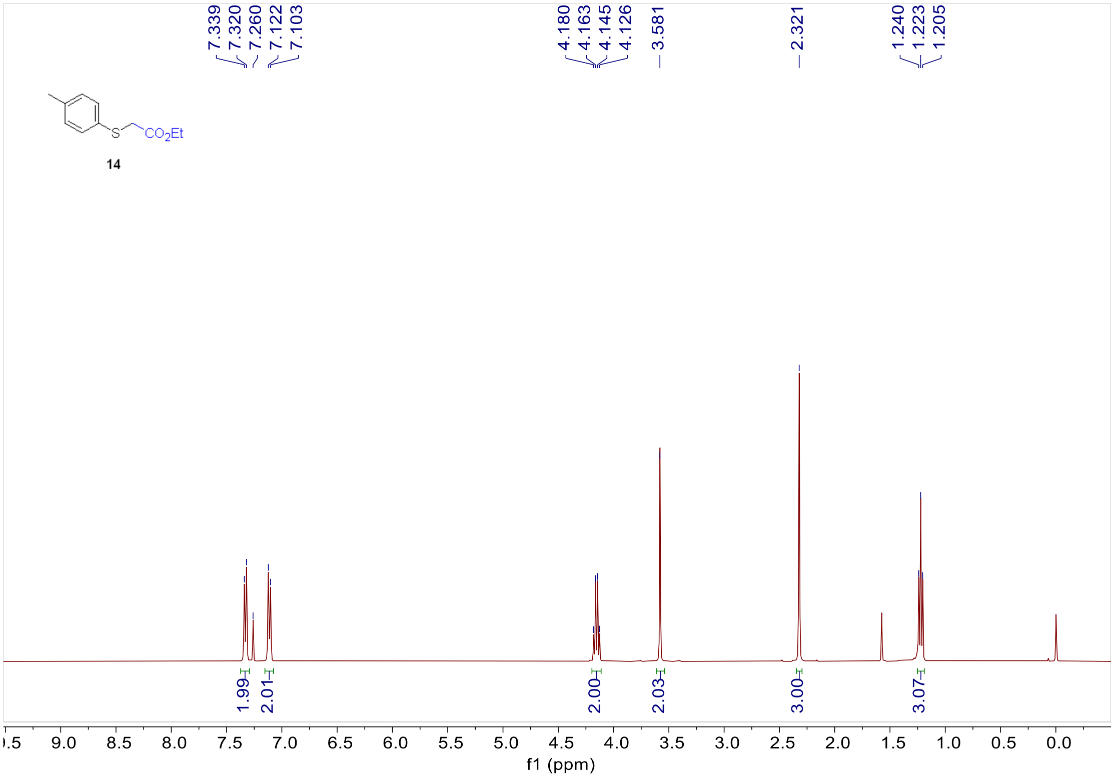

^{13}C NMR (100 MHz) Spectrum of 14 in $CDCl_3$

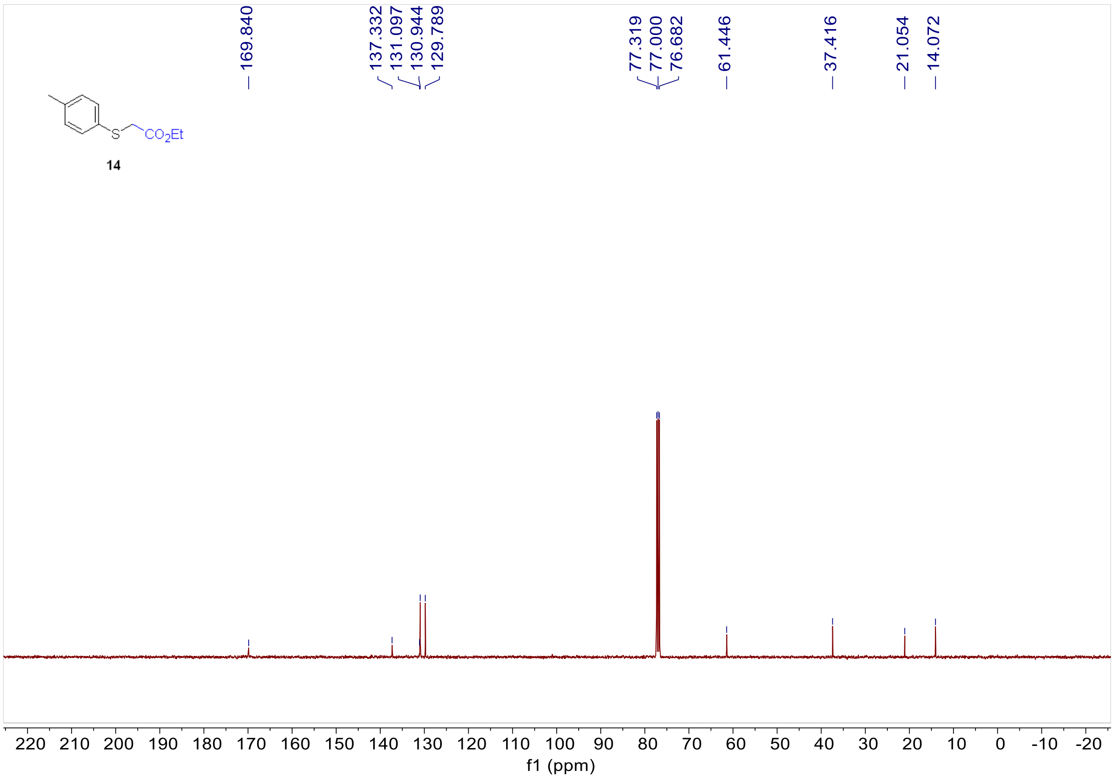

^{1}H NMR (600 MHz) Spectrum of 15 in CDCl$_3$

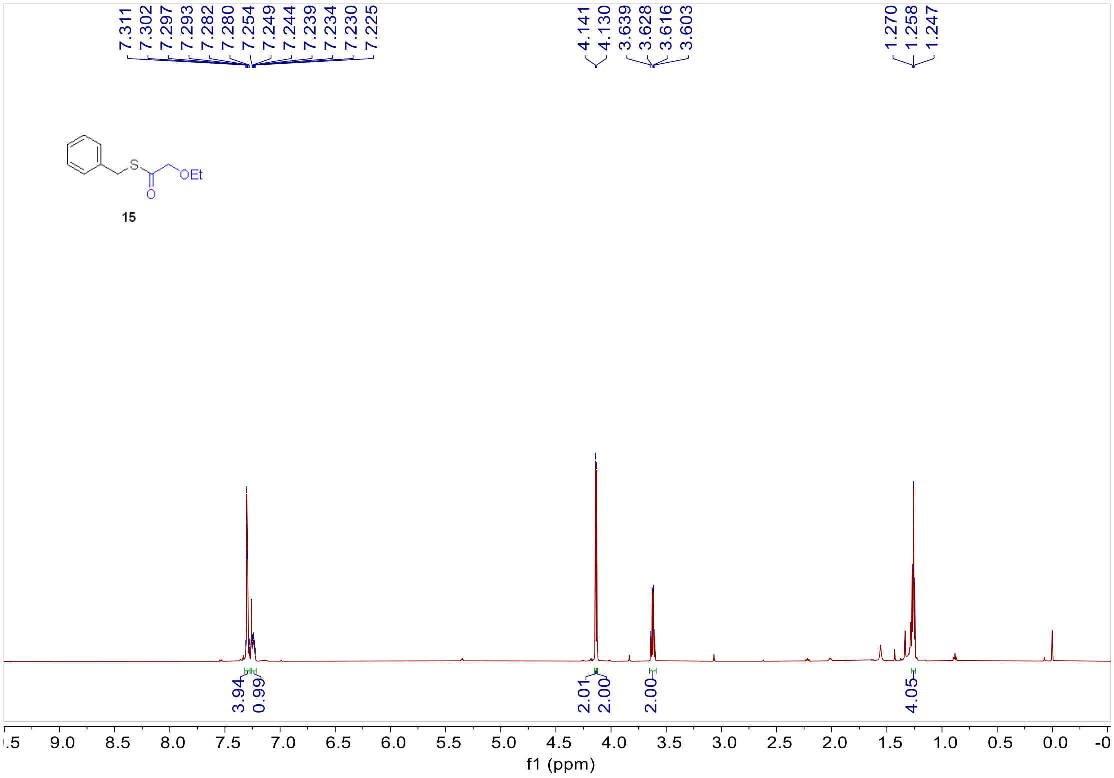

^{13}C NMR (150 MHz) Spectrum of 15 in CDCl$_3$

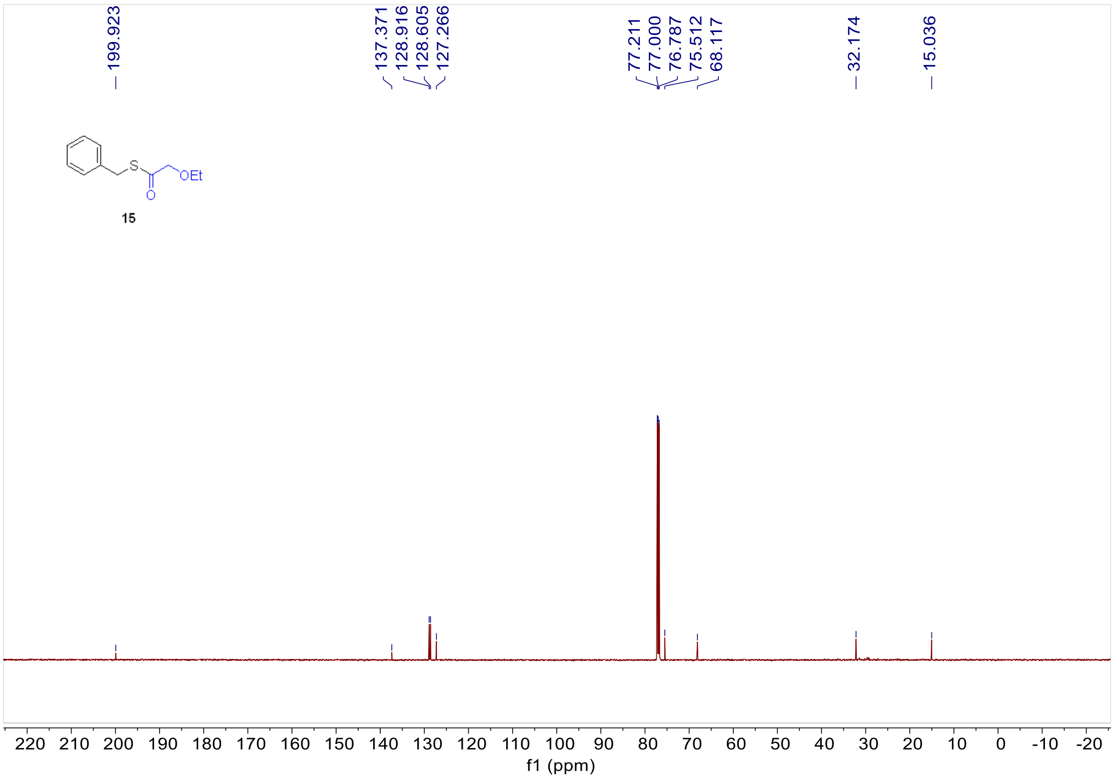

Supplement: SC-017-D5SC08263C-s001 [file SC-017-D5SC08263C-s001.pdf]
